# Molecular Diversity from Longipinenes of *Santolina viscosa* Lag. through Acid Catalysis: Biocidal Activity

**DOI:** 10.3390/biom14070780

**Published:** 2024-06-30

**Authors:** Irene Torres-García, José F. Quílez del Moral, Alejandro F. Barrero, Azucena González-Coloma, María Fe Andrés, José L. López-Pérez, Miriam Álvarez-Corral, Ignacio Rodríguez-García, Manuel Muñoz-Dorado

**Affiliations:** 1 Organic Chemistry, University of Almería, ceiA3, 04120 Almería, Spain; itg491@ual.es (I.T.-G.); malvarez@ual.es (M.Á.-C.); irodrigu@ual.es (I.R.-G.); 2 Organic Chemistry, Institute of Biotechnology, University of Granada, 18071 Granada, Spain; jfquilez@ugr.es (J.F.Q.d.M.); afbarre@ugr.es (A.F.B.); 3 Institute of Agricultural Sciences—CCMA, CSIC, 28006 Madrid, Spain; azu@ica.csic.es (A.G.-C.); mafay@ica.csic.es (M.F.A.); 4 Pharmaceutical Chemistry, CIETUS, IBSAL, University of Salamanca, 37007 Salamanca, Spain; lopez@usal.es; 5 CIPFAR. Department of Pharmacology, Faculty of Medicine, University of Panamá. Ave. Octavio Mendez Pereira, 3366 Panama City, Panama

**Keywords:** natural product, *Santolina viscosa*, Δ^9^-longipinenes, molecular rearrangement, ring-opening and bioactivity

## Abstract

The search for new compounds with biocidal potential was carried out, focusing on the longipinenes **1**–**7** from the plant species *Santolina viscosa* Lag. Compounds **1**, **2**, and **5** showed remarkable molecular diversity when treated in acidic reaction conditions. Protonic, Lewis, and heterogeneous compounds were used in the treatment. Three main models of reaction have been observed: isomerization of the double bond (**8**–**10**); rearrangements to longibornane-based skeleton (**11**–**15**) and ring-opening to himachalane-based skeleton (**16**–**18**). *Seco*longibornane aldehydes **23** and **24** were obtained after epoxide opening under the same reaction conditions. The elucidation of the structures of the new compounds was carried out using spectroscopic data and was supported by computational theoretical calculations of ^13^C NMR spectra. Additionally, high-resolution mass spectrometry and single-crystal X-ray diffraction analysis were employed for certain compounds. Natural longipinenes **4**–**7**, methyl esters **1**–**3** of corresponding natural carboxylic acids and the isomerized and derivatives compounds **8**–**19** exhibit moderate to high insecticidal activity against *R. padi* and *M. persicae* insects. Longipinene **5** shows potent inhibition against the root growth of the plants *L. perenne* and *L. sativa*, as well as compound **2** on the leaves of *L. perenne*. Furthermore, significant ixocidal and nematicidal activity was found for this latter compound.

## 1. Introduction

Due to the excessive use of chemical pesticides, the accumulation of their residues and metabolites in the environment poses risks to human health and diminishes biodiversity in agricultural areas [1]. In fact, recently, the EU has regulated the widely used herbicide glyphosate due to its possible carcinogenic properties [2]. Our interest in the preparation of sesquiterpenes arises from the possibility of discovering new molecules with biocidal properties that are more efficient and less harmful to the environment than those currently employed.

**Figure 1:**
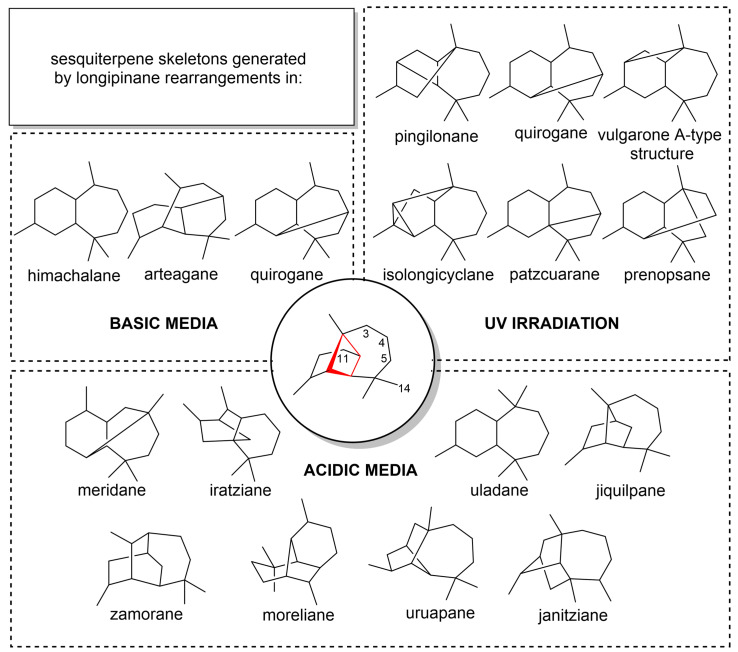
Sesquiterpene skeletons generated by longipinane rearrangements in acidic, alkaline and photochemical conditions.

Longipinenes exhibit a distinct tendency for molecular rearrangements, consequently generating great molecular diversity. This phenomenon has been observed in longipinanes isolated from the genus *Stevia*, such as rastevione [3]. Generally, these compounds have the C3, C4, and C5 positions oxidized with acyloxy and/or hydroxyl groups, in addition to a carbonyl group present at C11 that is conjugated in most compounds. The C14 position can also be functionalized but is less common in nature [4]. The exploration of the chemistry of this unique tricyclic ring system has led to the discovery of a variety of hydrocarbon skeletons including moreliane [3], arteagane [5,6], uruapane [7], jiquilpane [8], meridane [9,10], quirogane [11], among others, through Wagner-Meerwein rearrangements or Michel retro reactions. Alternatively, skeletons such as pingilonane [12] or patzcuarane [13] have seen synthesized photochemically (Figure 1). The location of functional groups on the longipinane skeleton is crucial to direct the formation of new compounds by releasing the four-membered ring strain under acidic [3,7,8,9,14,15,16,17,18] and alkaline [5,6,11,15] conditions, or through UV irradiation [12,13,19]. Most of these studies have been conducted by Dr. Joseph Nathan’s team, considered a pioneer in the field of longipinane chemistry.

This type of skeleton has also been identified in compounds isolated from plant species suchas as *Santolina viscosa* Lag., an endemic plant of southwestern Spain [20,21]. This work reports the chemical behavior under acidic conditions of the major longipinenes obtained from extracts of *Santolina viscosa* Lag.

## 2. Results and Discussion

**Figure 2:**
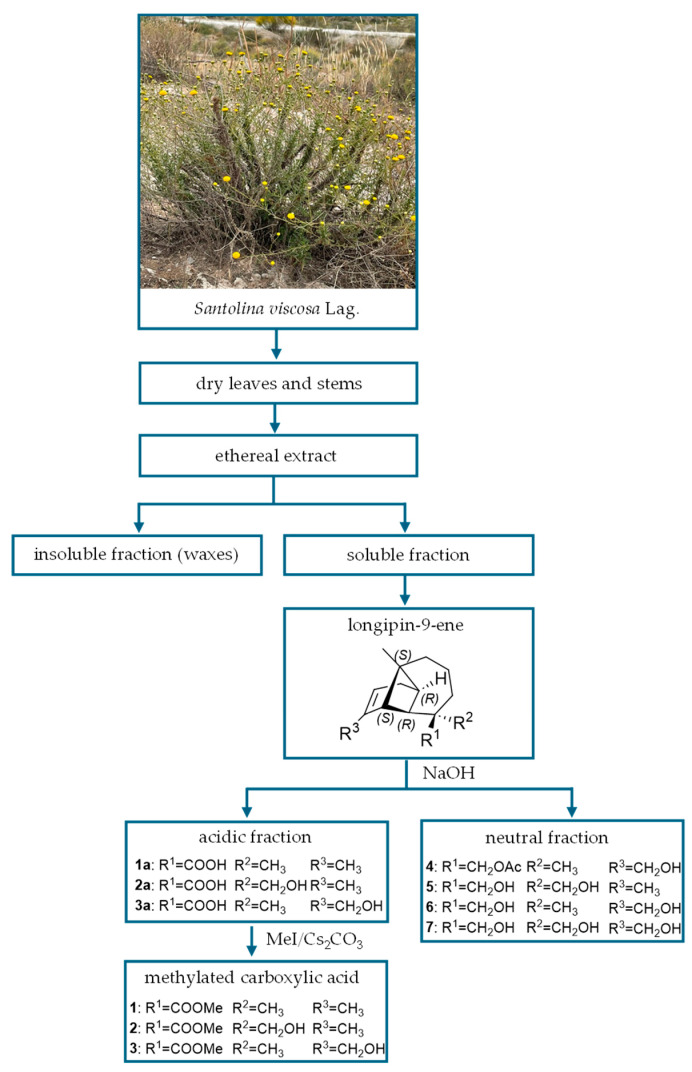
The major Δ^9^-longipinenes isolated from *Santolina viscosa* Lag.

*Santolina viscosa* Lag. was collected in Los Yesares de Tabernas (Almería, Spain). Dry leaves and stems (approximately 700 g) were extracted by immersion in diethyl ether at room temperature for 24 h. The crude extract (approximately 70 g) was enriched in longipin-9-enes, being seven the major compounds (Figure 2). Fractionation by RRE with alkaline aqueous eases separation of three are carboxylic acids (**1a**–**3a**), subsequently methylated (**1**–**3**), and also, four are compounds exclusively with hydroxyl groups (**4**–**7**). Thus, this plant constitutes an excellent molecular factory toward isolation of large amounts of these compounds in pure form, except for **3**, which was obtained as an inseparable mixture with its isomer **2**. The Δ^9^-longipinenes isolated in this study turned out to be the same compounds isolated from this same species that Barrero et al. described between 1994 [20] and 2000 [21]. 

**Reactions.** Each one of the longipinenes **1**, **2**, and **4**–**7** were treated in different acidic media. Heterogeneous catalysts such as zeolite Y-CBV720 and silicotungstic acid hydrate (H_6_SiW_12_O_41_), Lewis acids such as InCl_3_, or homogeneous protic acids such as HSO_3_F were employed. The experimental procedures are described in the Supplementary Materials.

**Figure 3:**
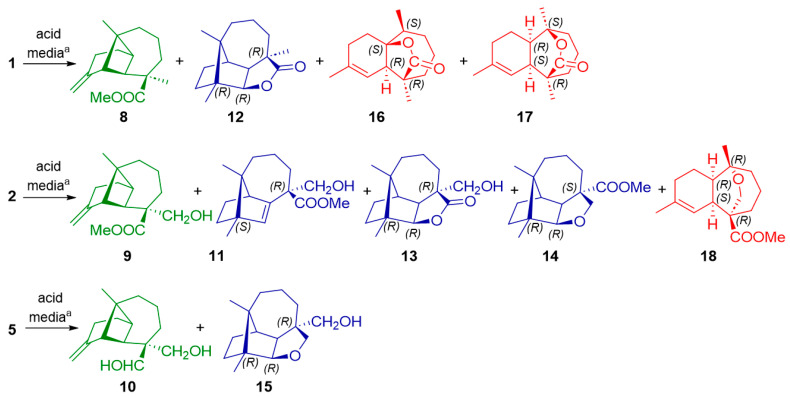
New compounds obtained through treatment in acid medium of longipinanes **1**, **2** and **5**. ^a^ For details of the compound isolated and the corresponding yield, see Table 1.

Reaction of the sesquiterpenes bearing hydroxyl groups in the allylic position C12 (**4**, **6**, and **7**) resulted in an irresolvable mixture of compounds after treatment. However, longipinenes **1**, **2** and **5** provided several products which are easily isolated (Figure 3). For example, **1** in the presence of the zeolite Y-CBV 720 catalyst led to the formation of compounds **8**, **12**, **16** and **17** (Figure 3, Table 1). In these reactions, three classes of interesting transformations in the original longipinene skeleton were observed: the simplest reaction was the double bond isomerization (**8**–**10**) in which the longipinene structure was maintained. In the other transformation class, the original skeleton was rearranged by expanding the four-membered ring to a new five-membered ring. The newly structures have a longibornane-based skeleton (**11**–**15**). In third place, the opening of the four-membered ring occurred, forming structures with a himachalane-based skeleton (**16**–**18**). Interestingly, functional groups in starting products played a pivotal role in the formation of these classes of compounds, yielding lactones from methyl esters and ethers from alcohols in tetra- and tricyclic systems (Figure 3).

In Table 1, the yields of all transformations are summarized. The widest array of compounds obtained from longipinene **1** occurred when treating this compound with zeolite Y-CBV720 (Table 1). However, treatment with H_6_SiW_12_O_41_ is more selective towards the formation of himachalanes. Longipinene **2** is more versatile due to the presence of hydroxyl and ester groups in its structure. While the use of zeolite Y-CB720 favors the formation of **9**, the use of H_6_SiW_12_O_41_ forms longibornane as the major compound, and the use of HSO_3_F directs the reaction towards the formation of himachalane **III**. Treatment with InCl_3_ allows more compounds to be obtained but renders the reaction less selective. The reaction of longipinene **5** with InCl_3_ is regiospecific towards the formation of longibornane **II**.

**Scheme 1:**
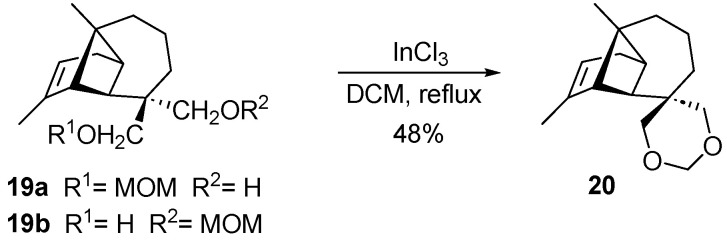
Formation of **20** from the isomeric mixture **19a**–**19b**.

On the one hand, when the isomeric mixture **19a**–**19b** obtained from the partial protection of the hydroxyl groups with MOM was treated with InCl_3_, compound **20** was formed. The formation of a new six-membered ring is justified by the nucleophilic attack of the hydroxyl group on the methylene of MOM, causing the loss of the protecting group in the form of a leaving group (Scheme 1).

**Scheme 2:**
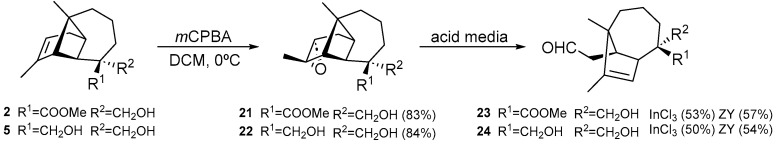
Obtaining *seco*longibornane aldehydes (**23**–**24**) through the epoxide ring opening (**21**–**22**) with various acidic compounds.

On the other hand, longipinenes **2** and **5** were treated with *m*CPBA to stereoselectively yield the corresponding epoxides (**21**–**22**). Epoxide ring opening on **21**–**22** was attempted under acidic conditions using InCl_3_ and zeolite Y-CBV720. Two new rearranged compounds were obtained with a skeleton known [22], the *seco*longibornane aldehyde (**23**–**24**) (Scheme 2).

**Structural elucidation.** The structures of the new compounds were elucidated through their spectroscopic data. A 600 MHz nuclear magnetic resonance (NMR) spectrometer was utilized, conducting both 1D (^1^H, ^13^C, DEPT135) and 2D (HSQC, HMBC, COSY, NOESY, TOCSY) experiments (Supplementary Materials). These structures were corroborated by computational theoretical calculations of ^13^C NMR spectra. Additionally, high-resolution mass spectrometry (TOF MS ES+) was applied to certain samples, and single-crystal X-ray diffraction analysis was performed on compound **13**.

The HRESIMS data of longipinene **9** establish their molecular formula as C_16_H_24_O_3_ with an [M+H]^+^ ion at *m/z* 265.1797 (calculated as 265.1804 for C_16_H_24_O_3_H^+^). The ^1^H NMR data show two characteristic singlets of an exocyclic system at δ 4.49 and 4.72 (H12). These protons correlate with a secondary carbon at δ 166.5 (C9) on the HSQC spectrum. The spectroscopic data obtained suggest the preservation of the original longipinane structure. For compound **10**, the NMR data are like **9**, except for a second primary alcohol at position 15 (^1^H NMR: δ 3.55 (s); ^13^C NMR: δ 70.6 (CH_2_OH). Generally, endocyclic bonds are more stable in polycyclic systems; however, in this type of structure, due to the highly strained tricyclic system, the exocyclic double bond seems more favorable.

The ^1^H NMR spectrum of **11** shows the most deshielded signal at δ 5.18 (m, H8) belonging to a trisubstituted double bond; two diastereotopic oxygenated protons (δ 3.74, d, *J* = 11.4 Hz, H14a; δ 3.67, d, *J* = 11.4 Hz, H14b) geminal to an ester group (δ 3.60, s, COOCH_3_); and two methyl groups as singlets at δ 0.99 and 0.90 (H12 and H13). The O-H stretching observed in the IR spectrum at 3465 cm^−1^ as well as the C=O stretching at 1720 cm^−1^ confirm the presence of the hydroxyl group and the ester group in the structure. The signals of C7 (δ 154.0, C) and C8 (δ 112.20, CH) in ^13^C NMR spectra corroborate the presence of an endocyclic bond. A clear correlation of C12-H13 was observed in the HMBC spectrum. That suggested a rearrangement through the expansion of the four-membered ring to a new five-membered ring. Thus, this sesquiterpene presents a longibornane skeleton. 

In the ^1^H NMR spectra of **12**, the signal at δ 4.24 (d, *J* = 7.8 Hz, H8) of an oxygenated methine proton coupled to another methine proton at δ 2.19 (d, *J* = 7.8 Hz, H7) is observed, along with three methyl groups as singlets at δ 1.44, 0.97, and 0.80 (H14, H12, H13). The most characteristic signal of the IR spectrum corresponds to the C=O stretching at 1748 cm^−1^, characteristic of a lactone group. In the HMBC spectrum, the correlation between the acyl group quaternary carbon C15 (δ 185.4) and proton H8 can be observed, confirming the structure of lactone C15-O-C8, and the correlation of C12-H13 confirms the expansion of the four-membered ring to a five-membered ring. The new rearranged compound is a tetracyclic lactone with a longibornane-based skeleton. 

**Figure 4:**
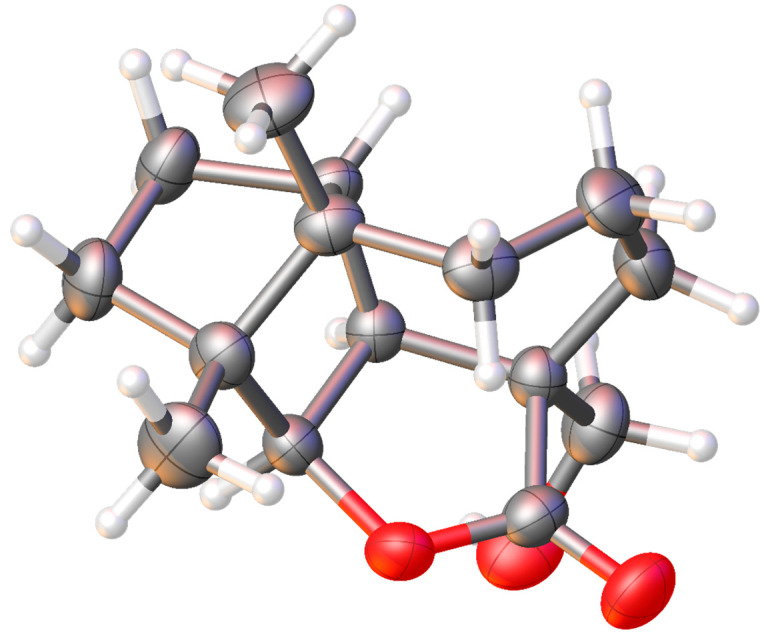
X-ray structure of longibornane **13**.

The formation structure of longibornane **13** has similar spectroscopic characteristics as **12**, except for a primary hydroxyl group (δ 3.55, d, *J* = 8.4 Hz, H14a); δ 3.33 (d, *J* = 10.2 Hz, H14b) geminal to the acyl group. The O-H stretching observed in the infrared spectrum at 3399 cm^−1^ and the C=O stretching at 1730 cm^−1^ confirm the presence of the hydroxyl group and lactone group in the structure. The HRESIMS data of **13** establish their molecular composition as C_15_H_22_O_3_ with an [M+H]^+^ ion at *m/z* 251.1648 (calculated as 251.1647 for C_15_H_22_O_3_H^+^). It was obtained as colorless crystals and subjected to X-ray diffraction (Figure 4). Crystal structure and least squares refinement were performed with ShelX [23,24,25]. Its absolute configuration was established by calculating the Flack parameter (Supplementary Materials). Thus, its structure and stereochemistry are confirmed. Crystallographic data have been deposited with the Cambridge Structural Data Centre as supplementary publication number CCDC 2363257.

The molecular composition of **14** was established as C_16_H_24_O_3_ with an [M + H]^+^ ion at *m/z* 265.1790 (calculated as 265.1804 for C_16_H_24_O_3_H^+^). Their spectroscopic data are in accord with a longibornane backbone. The O-H stretching is not observed in the infrared spectrum. In the ^1^H NMR spectrum, signals of protons attached to oxygenated carbons are observed at δ 3.75 (1H, d, *J* = 1.2 Hz, H8) and δ 3.73 (2H, m, H14), an ester group with its methyl group appearing as a singlet at δ 3.61 (s, COOCH_3_), and two methyl groups at δ 0.91 (s, H12) and δ 0.78 (s, H13). In the ^13^C NMR spectrum, the CH oxygenated C8 signal is deshielded at δ 91.7. These spectral features support the formation of a cyclic ether. The spectroscopic ^1^H NMR data of longibornane **15** are similar, except for a primary hydroxyl group at position 14 (δ 3.44 (d, *J* = 10.6 Hz, H14a); δ 3.39 (d, *J* = 10.6 Hz, H14b); O-H: ν 3373 cm^−1^).

The ^1^H NMR spectrum of compound **16** reveals signals for a vinylic proton and methyl group at δ 5.15 (s, H8) and δ 1.65 (s, H12). A doublet peak integrating for three protons at δ 1.02 (d, *J* = 7.2 Hz, H13) indicates opening of the strained four-membered ring and another singlet peak was observed at δ 1.09 belonging to the H14 methyl group. In the TOCSY spectrum, the correlation of the H8 proton with the H7, H10 and H11 protons can be observed. The ^13^C NMR data, along with a DEPT experiment, show the signal of a carbonyl group of cyclic ester at δ 183.4 and a quaternary oxygenated carbon at δ 85.4, indicative of lactonization. This cyclization occurs between positions C15-C1, as observed in the HMBC data by correlation of C1 with H2. The most characteristic signal of the IR spectrum corresponds to the C=O stretching at 1760 cm^−1^, characteristic of a lactone group. Compounds **16** and **17** have the same himachalane backbone structure, but the acyl group cyclization occurs at different positions. Lactone **17** is formed at positions C15-C2. This is evidenced in the ^1^H NMR spectra where the methyl group protons H13 appear as a singlet at δ 1.37 and in the HMBC data where the correlation between methyl group H13 and oxygenated carbon C2 (δ 83.8) is observed.

The molecular composition of C_16_H_24_O_3_ for **18** was obtained from HRESIMS, which showed an [M + H]^+^ ion at *m/z* 265.1795 (calculated as 265.1804 for C_16_H_24_O_3_H^+^). The O-H stretching is not observed in the infrared spectrum. The ^1^H NMR spectrum exhibits signals for a vinylic proton and methyl group at δ 5.25 (s, H8) and δ 1.66 (m, H12), two methylene protons at δ 3.95 (dd, *J* = 10.2, 1.8 Hz, H14a) and δ 3.81 (1H, d, *J* = 10.2 Hz, H14b) bearing the oxygen atom of the ether bridge, and an ester group with its methyl group singlet appearing at δ 1.14 (s). The correlation observed in the HMBC spectrum between H13 (δ 1.14, s) and C2 (δ 76.5), as well as H14, confirms the C13-C2-O-C14 linkage. The resulting tricyclic ether compound has a himachalane backbone.

In the ^1^H NMR spectrum of **20**, signals are observed for a vinylic proton and a methyl group at δ 5.16 (s, H10) and δ 1.61 (d, *J* = 1.8 Hz, H12), two doubly oxygenated methylene protons at δ 4.75 (d, *J* = 6.6 Hz, H16a) and δ 4.69 (d, *J* = 6.6 Hz, H16b), and two oxygenated methylene groups at δ 3.56 (dd, *J* = 33.6, 10.8 Hz, -CH_2_O-) and 3.46 (dd, *J* = 33.6, 10.8 Hz, -CH_2_O-). In the ^13^C NMR data, C16 appears at δ 94.3, showing a correlation in the HMBC data with the -CH_2_O- protons. Furthermore, O-H stretching is not observed in the infrared spectrum, indicating the formation of the 1,3-dioxane structure.

The acid treatment of epoxides **21** and **22** resulted in the formation of the aldehydes *seco*longibornane [22] (**23**–**24**). In the ^1^H NMR spectrum of **23**, in addition to the characteristic signs of the skeleton, a primary hydroxyl group at δ 3.67 (d, *J* = 11.2 Hz, H14a) and δ 3.49 (d, *J* = 11.2 Hz, H14b) and an ester group with its methyl appearing at δ 3.61 (s) were observed. The HRESIMS data of **24** established their molecular composition as C_15_H_24_O_3_ with the ion [M + H]^+^ at *m/z* 253.1795 (calculated as 253.1804 for C_15_H_24_O_3_H^+^). Its ^1^H, ^13^C NMR and IR spectra show similarities with those of **23**, but in C15, a second primary hydroxyl group is observed at δ 3.55 (dd, *J* = 10.8, 1.8 Hz, H15a) and δ 3.44 (d, *J* = 10.8 Hz, H15b).

**Computational calculations.** The correct structure of these different structural proposals was verified through computational chemistry. Their respective theoretical spectroscopic data were obtained and compared with experimental data. For this purpose, Spartan’20 was chosen as it has implemented an appropriate protocol for calculating ^13^C NMR chemical shifts in conformationally flexible molecules such as those under study. This protocol comprises a multi-step procedure to accurately determine Boltzmann weights, starting with a systematic conformational search using MMFF molecular mechanics and ending with a series of large basis set density functional theory energy calculations and empirically corrected small basis set density functional theory chemical shift calculations, each multiplied by its respective weight to produce a Boltzmann-averaged ^13^C NMR spectrum [26]. The details of this protocol are presented in the Supplementary Materials. In this protocol, the solvent has not been considered either in determining the conformational distribution or in calculating the chemical shifts of each of the conformers since the empirical correction scheme implemented in Spartan’20 has been parameterized based on experimental NMR data.

**Figure 5:**
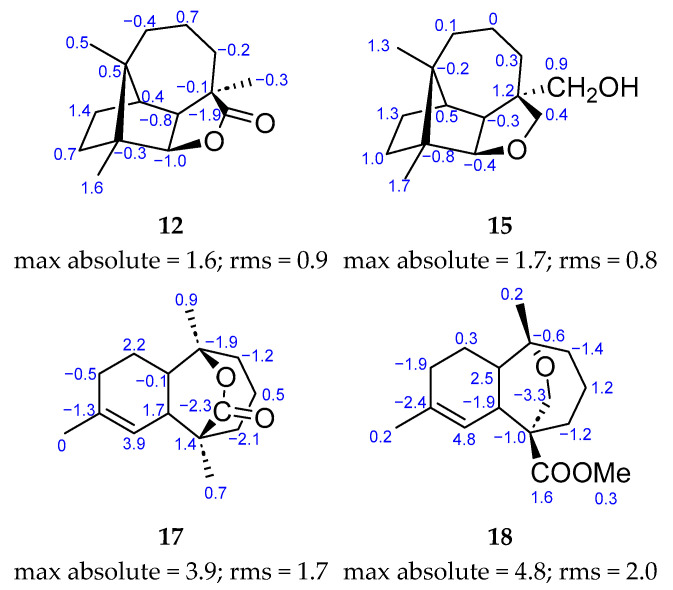
Confirmed structures for **12**, **15**, **17** and **18**. Each carbon shows the difference between the calculated and its experimental (Table 2) ^13^C NMR chemical shift. Underneath each structure are the terms max absolute and rms.

The structures of compounds **12**, **15**, **17**, and **18** are shown in Figure 5 along with the deviations of the calculated chemical shift values from the experimentally obtained values for each carbon (calculated value—experimental value). The largest of these values is shown at the bottom of each structure as a maximum absolute term, alongside the rms (root mean square) value. Both the maximum absolute value and the rms value are acceptable for the four compounds, thus indicating an appropriate fit. This confirms the proposed structures for **12**, **15**, **17**, and **18** (Table 2).

For compound **16**, the formation of two possible stereoisomers (**16**: *2S* and **16a**: *2R*) was studied (Table 3). After performing computational calculations on both compounds, we concluded that **16** is the correct structure. Although there are no significant differences between the experimental and calculated ^13^C NMR chemical shifts for both compounds, structure **16** fits the data in a slightly better manner, and it is also 8 kcal/mol more stable than **16a**.

The elucidation of molecule **14** was controversial. Initially, for **14a**, derived from **2**, the same configuration *R* for C6 was proposed. However, computational data showed high deviations for C6, C7, and C9 (Table 3). The exchange in the configuration at C6 (**14**) showed a maximum absolute value of 1.8 and a rms of 0.9 in the results obtained through computational calculations. An excellent correlation was obtained for cyclic ether **14-(6*S*)** between the values of experimental and computational ^13^C NMR chemical shifts. This confirms structure **14** as the obtained compound.

**Scheme 3:**
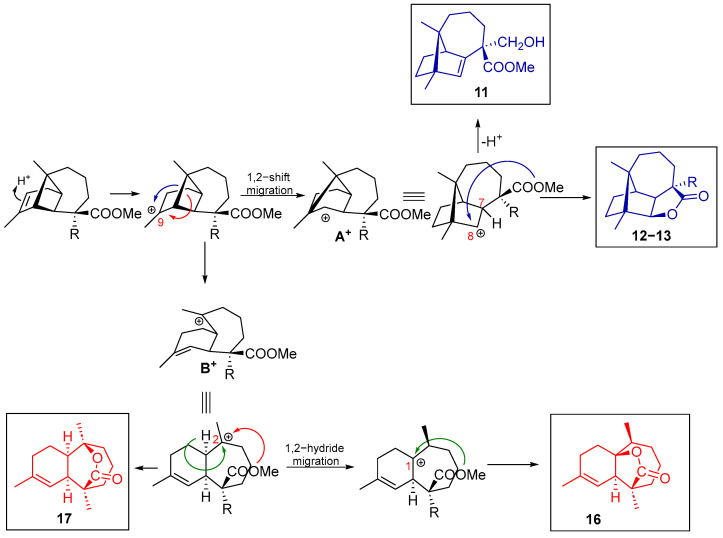
Proposed mechanism for the formation of longibornane **11** and sesquiterpene lactones **12**, **13**, **16,** and **17**.

**Mechanistic discussion.** The reaction mechanism of these compounds begins with the attack of the π electrons of the double bond towards the acid catalyst present in the medium. This generates a carbocation at the C9 position, which induces Wagner–Meerwein 1,2 migration of the C2–C8 antiperiplanar bond to form the C2-C9 bond, resulting in the formation of the cationic intermediate A+. The intermediate B^+^ can also be generated by opening the cyclobutane ring (Scheme 3).

From the intermediate A^+^, upon loss of the proton at C7, the Δ^7^ double bond of longibornene **11** is formed. The formation of longibornanes **12**–**13** occurs from the carbocation at position C8 prone to form a lactone ring by the attack of the ester group at C15 with concomitant Me elimination thanks to the anion from the catalyst. Furthermore, the generation of the B^+^ intermediate has two possible alternatives: 1,2 hydride migration followed by the subsequent attack of the ester group to form a γ-butyrolactone with a himachalane-based skeleton (**16**), or the direct attack of the ester group on the carbocation at C2, generating a δ-valerolactone with a himachalane-based skeleton (**17**).

**Scheme 4:**
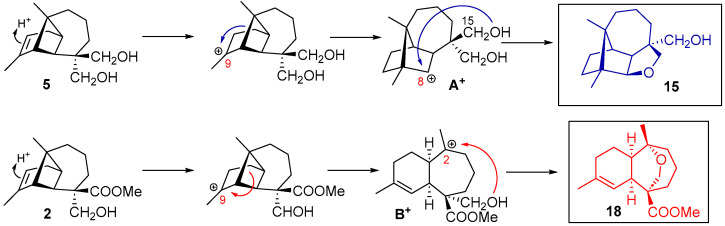
Cyclic ether sesquiterpenes from longipinenes **5** and **2**.

**Scheme 5:**
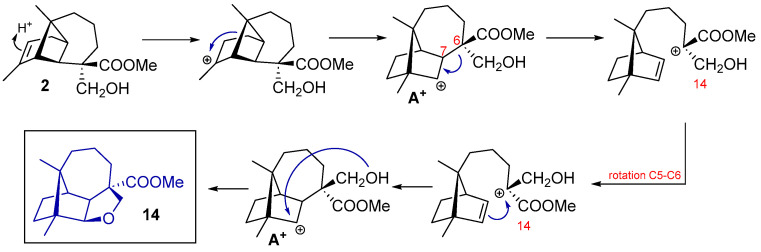
Molecular cyclic ether rearrangement **14** from longipinene **2**.

The formation of cyclic ethers follows a similar mechanism as that proposed to the lactones. Compounds **15** and **18** are formed through intermediates A^+^ and B^+^, respectively, with the attack of the hydroxyl group on the generated carbocation. The hydroxyl group located at C15 is responsible for cyclization through the front side of the molecule at **15** and the hydroxyl group located at C14 is responsible for cyclization through the back side of the molecule at **18** (Scheme 4). From natural longipinene **2**, the formation of a cyclic ether with a longibornane-based skeleton also occurs (Scheme 5). After confirming the structure **14** through computational calculations, which involve the exchange between substituents C14 and C15, a mechanism is proposed to explain the formation of this longibornane. In this case, the breaking of C6–C7 bond in intermediate A^+^ origines a carbocation at C6. At this point, a rotation of the C5–C6 carbons occurs, positioning the hydroxyl group at C15, where intermediate A^+^ is regenerated and then attacked by the hydroxyl group to form **14** (Scheme 5). 

**Biocidal activity of *Santolina viscosa* extracts.** The extracts of *S. viscosa* (ether E; neutral fraction NF, acidic fraction AF, esterified acid fraction AEF) were tested for their crop protection effects, including antifeedant assays against insect pests (*Spodoptera littoralis*, *Rhopalosiphum padi* and *Myzus persicae*), nematicidal activity against the root-knot nematode *Meloidogyne javanica* and the phytotoxic activity against *Lolium perenne* and *Lactuca sativa.* The details of these assays are presented in the Supplementary Materials [27,28,29,30,31,32].

All the extracts exhibited significant antifeedant activity against the insects tested (Table 4). *S. littoralis* was moderately affected by NF (EC_50_ of 41 μg/cm^2^) and AEF (EC_50_ of 32 μg/cm^2^) and *M. persicae* by E, NF (EC_50_ of 19 μg/cm^2^), and AEF with a strong effect (EC_50_ of 3 μg/cm^2^), while *R. padi* was only affected by AEF (EC_50_ of 14 μg/cm^2^). Therefore, the esterified acid fraction is enriched in insect antifeedant compounds. None of these extracts were active against the nematode *M. javanica (mortality* values between 5 and 34%). 

**Figure 6:**
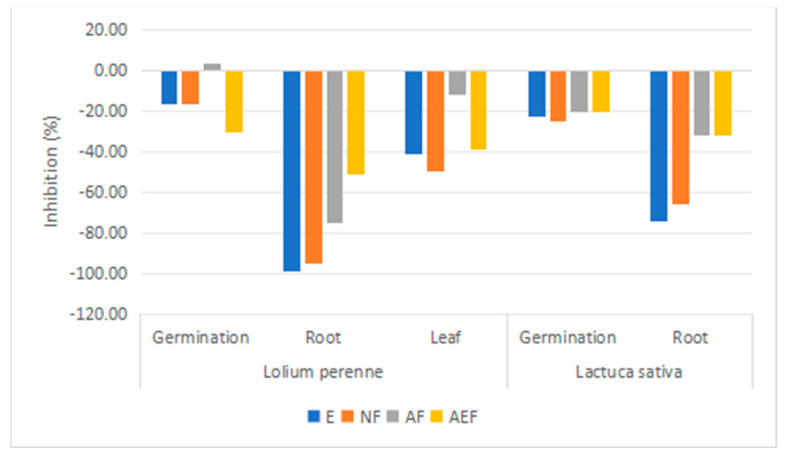
Phytotoxic effects of *S. viscosa* extracts (ether, E; neutral fraction, NF; acidic fraction, AF; acid esterified fraction, AEF) on *Lolium perenne* and *Lactuca sativa* (germination and root-leaf growth) expressed as percent relative to the control.

The *S. viscosa* extracts were tested (0.4 mg/mL) for their phytotoxic effects against *L. perenne* and *L. sativa* seeds (Figure 6). Specifically, the extracts ether E, neutral fraction NF and acidic fraction AF inhibited the root growth of *L. perenne* (99, 94, and 75% respect to the control). *L. sativa* root growth was less affected by extracts E and NF (74 and 65 %) than *L. perenne*. These results indicate the potential allelopathic effects of *S. viscosa* and the presence of herbicidal components mostly in the ether extract (E) and neutral fraction (NF).

**Biocidal activity of longipinenes and the synthetic derivatives.** The structural diversity obtained from the longipinenes isolated from *S. viscosa* has enabled a comprehensive study in the search for new sustainable biocidal agents. The effects of the isolated longipinenes (**1**–**7**) were evaluated, along with the synthesized compounds (**8**,**9**,**12**,**13**,**15**,**17**–**19** and **22**–**24**) for antifeedant, nematicidal phytotoxic, and ixodicidal effects, as previously performed for the extracts. 

None of the pure compounds tested (**1**–**9**, **12**–**15**, **17**–**19**, **22**–**24**) were antifeedant against the cotton leafworm *S. littoralis* in contrast to the results found for the extracts, therefore suggesting synergistic effects for these mixtures. However, interesting results were obtained against the aphids (*R. padi* and *M. persicae*) (Table 5). The longipinenes (**1**–**7**) were all antifeedant to *M. persicae,* with EC_50_ values ranging between 7 and 22 μg/cm^2^, with **3** being the most effective. Compound **3** also had the highest effect against *R. padi* (EC_50_ of 8 μg/cm^2^) compared to ester **5**, which showed moderate activity (EC_50_ of 21 μg/cm^2^) or the inactive triol **7**. Among the newly synthetic compounds (**8**–**24**), a lower proportion (6 of 11) was antifeedant compared to the natural ones (7 of 7). The migration of the double bond from C9-C10 (**2**) to C9–C12 (**9**) decreased the activity on *M. persicae* (EC_50_ of 12 and >50 μg/cm^2^ respectively). However, the presence of the lactone group in **12** considerably increased the antifeedant activity against both aphids compared to **1** (EC_50_ of 5–7 and 22–50 μg/cm^2^ respectively). However, when the lactone presented a free alcohol, as in **13**, the effects dropped abruptly compared to **12** (EC_50_ of >50 and 5–8 μg/cm^2^ respectively). The position of the lactone (**12**: C8 or **17**: C2) resulted in similar effects, making these compounds very promising against both insects (EC_50_ of 5–8 and 8–12 μg/cm^2^ respectively). The cyclical ether **18** exhibited stronger activity against *M. persicae* than *R. padi* (EC_50_ of 8 and 18 μg/cm^2^ respectively). Similarly, **15** was also more effective for *M. persicae* but had overall activity lower than **18**. The low EC_50_ value obtained for isomers **19a**/**19b** makes this mixture a very potent antifeedant against *M. persicae* and possibly to *R. padi* (EC_50_ of <0.78 μg/cm^2^ for *M. persicae* and not calculated for *R. padi*), whereas for epoxide **22**, aldehydes **23**–**24** showed no antifeedant effects against these two species. 

When tested against the root-knot nematode *M. javanica*, only product **2** was nematicidal with lethal doses LC_50-90_ of 0.151–0.351 μg/μL, indicating a strong structural dependency of this effect.

**Figure 7:**
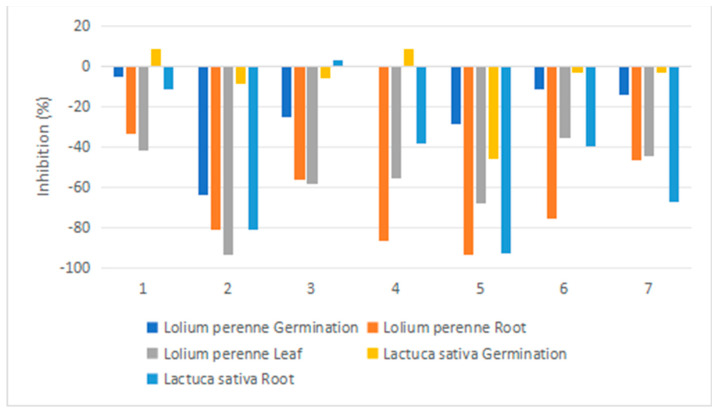
Phytotoxicity of natural products **1**–**7** against the plant species *Lolium perenne* L. and *Latuca sativa* at a concentration of 0.2 mg/mL (measurement: % of control).

The effect of the compounds **1**–**7** on *L. perenne* and *L. sativa* are shown in Figure 7. Compound **2** strongly inhibited the germination of *L. perenne* (81% inhibition) over the course of 168 h of experimentation. Most of the tested compounds inhibited root and leaf growth in both species, with **5** being a potent inhibitor of root growth of both species (93% inhibition), **2** inhibiting the roots of both species (81% inhibition) and the leaves of *L. perenne* (93% inhibition), and **4** selectively inhibiting *L. perenne* roots (86%). Therefore, these compounds are promising leads for the production of effective new herbicides.

**Figure 8:**
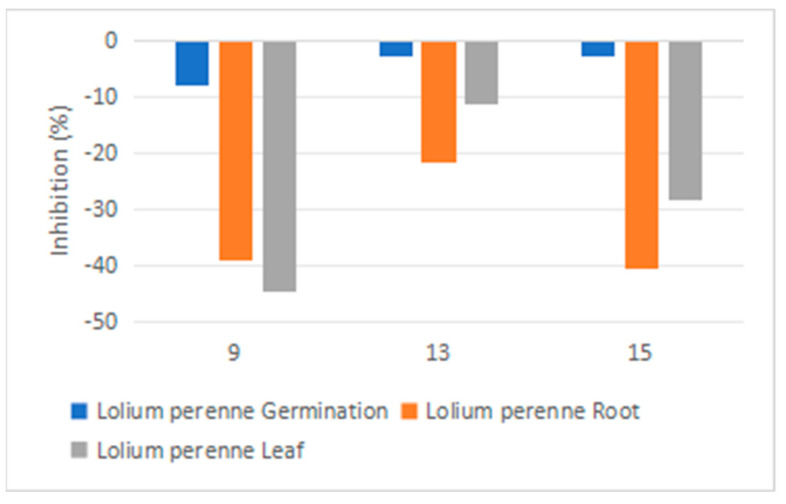
Phytotoxicity of compounds **9, 13, 15** against *Lolium perenne* at a concentration of 0.2 mg/mL (measurement: % of control).

Due to the limited amounts of synthetic compounds (**8**–**24**) available, only a few phytotoxicity tests could be conducted. Compounds **8**, **9**, **12**, **13**, **17** and **18** were tested against *L. sativa* and none of them showed phytotoxic effects. Compounds **9**, **13** and **15** were tested against *L. perenne (*Figure 8), with small effects on root and leaf growth (39%, 21% and 40% root and 44%, 11% and 28% leaf inhibitions).

## 3. Conclusions

Properly functionalized longipinanes are highly useful as starting compounds for forming other carbocyclic skeletons that can foster the development of new synthetic strategies. It can be concluded that the placement of hydroxyl and ester groups on the skeleton of longipinenes isolated from *Santolina viscosa Lag.* significantly influences the outcome of various transformations induced by treatment in acidic media. Consequently, three models of reaction have been observed: isomerization of the double bond; rearrangements to lactones/cyclic ethers with a longibornane-based skeleton; and ring opening to form lactones/cyclic ethers with a himachalane-based skeleton. The structures of the newly formed compounds were corroborated through computational calculations, and in some cases, their use was necessary to confirm the structure. Additionally, potential new insecticides could be appreciated such as compounds **4**–**7** and the isomeric mixture **19a**/**19b** or, alternatively, natural longipinene **2** as an excellent insecticide, nematicide, and herbicide due to its high efficacy shown in vitro.

## Figures and Tables

**Table 1:** Yields of the compounds obtained from longipinenes **1**, **2** and **5**.

Acid Catalyst	Longipinene 1	Longipinene 2	Longipinene 5
zeolite Y-CBV720	**8** * 12 (10%) **16** (5%) **17** (7%)	**9** (30%) **18** *	**10** (5%) **5** *
H_6_SiW_12_O_41_	**12** (18%) **17** *	**13** (38%) **18** *	**10** (4%) **15** *
InCl_3_	**8** * 16 (4%) **17** (4%)	**9** (14%) **11** (4%) **13** (3%) **14** (24%) **18** (26%)	**15** (36%)
HSO_3_F	**8** * 12 (19%) **17** (10%)	**9** (16%) **13** (15%) **18** (29%)	**15** *

* Estimation by NMR or only traces of crude oil.

**Table 2:** The calculated and experimental ^13^C NMR chemical shift (**δ**) for compounds **12**, **15**, **17** y **18**.

C	12		15		17		18	
	^13^C NMR Exp.	^13^C NMR Theory	^13^C NMR Exp.	^13^C NMR Theory	^13^C NMR Exp.	^13^C NMR Theory	^13^C NMR Exp.	^13^C NMR Theory
**1**	44.0	44.4	44.1	44.6	41.3	41.2	36.8	39.3
**2**	49.4	49.9	50.9	50.7	83.8	81.9	76.5	75.9
**3**	27.9	27.5	34.9	35.0	34.6	33.4	37.5	36.1
**4**	20.0	20.7	22.5	22.5	20.5	21.0	19.4	20.6
**5**	33.4	33.2	31.3	31.6	31.7	29.6	31.2	30.0
**6**	44.1	44.0	48.5	49.7	44.4	45.8	48.9	47.9
**7**	54.9	54.1	55.0	54.7	39.0	40.7	40.5	38.6
**8**	88.6	87.6	92.1	91.7	119.1	123.0	121.6	126.4
**9**	49.9	49.6	50.0	49.2	138.2	136.9	136.8	134.4
**10**	32.7	33.4	33.8	34.8	29.0	28.5	28.9	27.0
**11**	27.1	28.5	28.6	29.9	23.7	25.9	24.1	24.4
**12**	11.0	12.6	11.1	12.8	24.2	24.2	24.0	24.2
**13**	21.1	21.6	23.7	25.0	29.6	30.5	30.4	30.6
**14**	29.7	29.4	70.3	71.2	24.4	25.1	71.0	67.7
**15**	185.3	183.4	75.9	76.3	178.5	176.2	175.9	177.5
**16**	-	-	-	-	-	-	52.2	52.5

**Table 3:** Comparison of experimental ^13^C NMR data with computational data for epimers **14/14a** and **16/16a**.

**C**		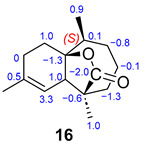 max absolute = 3.3; rms = 1.3	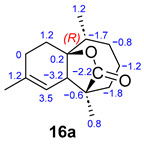 max absolute = 3.5; rms = 1.7		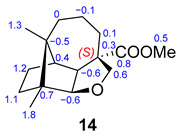 max absolute = 1.8; rms = 0.9	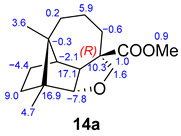 max absolute = 17.1; rms = 7.8
	**^13^C NMR** **Exp**	**^13^C NMR** **Theory**	**^13^C NMR** **Theory**	**^13^C NMR** **Exp.**	**^13^C NMR** **Theory**	**^13^C NMR** **Theory**
**1**	85.4	84.1	85.6	44.2	44.6	42.1
**2**	42.4	42.5	40.7	51.3	50.8	51.0
**3**	32.4	31.6	31.6	35.2	35.2	35.4
**4**	22.0	21.9	20.8	22.7	22.6	28.6
**5**	41.7	40.4	39.9	32.7	32.8	26.7
**6**	48.4	47.8	47.8	55.0	55.3	65.3
**7**	45.2	46.2	42.0	55.1	54.5	72.2
**8**	119.6	122.9	123.1	91.7	91.1	83.8
**9**	135.7	136.2	136.9	48.6	49.3	65.5
**10**	24.7	24.7	24.7	33.7	34.8	42.7
**11**	31.8	32.8	33.0	28.5	29.7	24.1
**12**	-	-	-	10.9	12.7	15.6
**13**	16.2	17.1	17.4	23.8	25.1	27.4
**14**	22.5	23.5	23.3	176.7	177.5	76.1
**15**	183.4	181.4	181.2	74.5	75.1	177.7
**16**	**-**	**-**	**-**	52.0	52.5	52.9

**Table 4:** Antifeedant effects of *S. viscosa* extracts (ether, E; neutral fraction, NF; acidic fraction, AF; acid esterified fraction, AEF) against sixth instar larvae (L6) of *S. littoralis* and adults of *M. persicae* and *R. padi.* Data are expressed as %FI / SI and efficient dose (EC_50_: concentration required to produce 50% inhibition in settling/feeding and 95% lower–upper confidence limits).

Extract	μg/cm^2^	*S. littoralis*	*M. persicae*	*R. padi*
E	%FI/SI	23.1 ± 7.6	71.78 ± 6.87	48.25 ± 6.76
	EC_50_	>100	19.8 (11.4–34.3)	>100
NF	%FI/SI	78.2 ± 12.1	73.98 ± 6.95	60.9 ± 6.8
	EC_50_	40.7 (24.8–67.2)	19.5 (11.0–34.5)	≈100
AF	%FI/SI	42.1 ± 10.5	53.47 ± 8.57	59.7 ± 6.3
	EC_50_	>100	≈100	≈100
AEF	%FI/SI	86.2 ± 8.7	80.8 ± 5.8	81.5 ± 5.8
	EC_50_	32.2 (17.7–58.6)	2.8 (1.2–6.3)	13.7 (7.8–24.3)

**Table 5:** Antifeedant effects (%SI/FI) and effective concentrations (EC_50_: concentration required to produce 50% inhibition in settlement/feeding and 95% confidence limits (lower, upper)) of pure compounds evaluated against adults of *R. padi* and *M. persicae*.

Compound	μg/cm^2^	*S. littoralis*	*M. persicae*	*R. padi*
**1**	%FI/SI	18.0 ± 7.4	73.5 ± 7.5	63.2 ± 5.6
	EC_50_	>50	22.3 (16.0–31.2)	≈50
**2**	%FI/SI	39.7 ± 15.1	83.06 ± 8.14	62.56 ± 7.6
	EC_50_	>50	11.8 (8.2–17.0)	≈50
**3**	%FI/SI	29.9 ± 13.3	86.3 ± 6.02	78.7 ± 5.7
	EC_50_	>50	6.7 (3.7–12.2)	8.0 (2.7–23.4)
**4**	%FI/SI	23.3 ± 5.7	85.8 ± 3.2	66 ± 6
	EC_50_	>50	11.5 (7.0–18.6)	>50
**5**	%FI/SI	41.6 ± 18.9	70.5 ± 9.2	69.3 ± 5.5
	EC_50_	>50	11.2 (6.1–20.7)	21.5 (16.2–28.5)
**6**	%FI/SI	27.9 ± 8.3	74.7 ± 6.9	5.06 ± 5.6
	EC_50_	>50	14.7 (8.3–25.7)	≈50
**7**	%FI/SI	29.0 ± 16.1	72.0 ± 7.2	35.5 ± 7.7
	EC_50_	>50	11.6 (6.4–21.1)	>50
**8**	%FI/SI	50.4 ± 14.8	85.9 ± 3.3	73 ± 5
	EC_50_	≈50	17.1 (12.7–22.9)	25–50
**9**	%FI/SI	11.3 ± 7.1	36.5 ± 7.1	57.5 ± 5.8
	EC_50_	>50	>50	>50
**12**	%FI/SI	49.2 ± 13.6	85.5 ± 6.7	97 ± 1
	EC_50_	≈50	5.5 (5.4–5.6)	7.6 (7.5–7.6)
**13**	%FI/SI	14.9 ± 7.4	47.9 ± 7.9	30.5 ± 6.7
	EC_50_	>50	>50	>50
**15**	%FI/SI	27.4 ± 11.7	85.9 ± 4.8	56.1 ± 8.2
	EC_50_	>50	12.5–25.0	≈50
**17**	%FI/SI	19.2 ± 10.5	81.6 ± 6.8	74.3.8 ± 7.0 *
	EC_50_	>50	8.1 (8.1–8.2)	11.8 (9.3–27.2)
**18**	%FI/SI	39 ± 10	86.5 ± 6.7	75.9 ± 6.0
	EC_50_	>50	7.7 (5.3–11.1)	17.7 (11.5–27.2)
**19/19a**	%FI/SI	58.7 ± 11.3	87.8 ± 3.2	83.2 ± 3.5
	EC_50_	≈50	<0.78	Nc
**22**	%FI/SI	37.9 ± 16.3	56.9 ± 10.4	24.8 ± 6.3
	EC_50_	>50	>50	>50
**23**	%FI/SI	28.6 ± 13.2	31.8 ± 6.6	31.0 ± 6.9
	EC_50_	>50	>50	>50
**24**	%FI/SI	30.3 ± 8.0	33.3 ± 9.5	35.2 ± 6.4
	EC_50_	>50	>50	>50

* Tested at 25 μg/cm^2^. Nc, not calculated.

## Data Availability

Data applicable on request.

## Supplementary Materials

The following supporting information can be downloaded at: https://www.mdpi.com/article/10.3390/biom14070780/s1. Experimental section (general remarks; computational chemistry calculations; biocidal activity; plant material; extraction and isolation; experimental procedures; characterization of molecules **9**–**24**), NMR / IR spectra and crystal data and structure refinement for **13**.
