# Supplementary material for: Molecular Diversity from Longipinenes of Santolina viscosa Lag. through Acid Catalysis: Biocidal Activity
Source: Biomolecules. 2024 Jun 30;14(7):780. doi: 10.3390/biom14070780 (PMC11274961; doi:10.3390/biom14070780)
Supplement: Supplementary file 1 [file biomolecules-14-00780-s001.zip › biomolecules-3047339-supplementary.pdf]

biomolecules

MDPI

Supplementary Materials

Molecular Diversity from Longipinenes of *Santolina viscosa* Lag. through Acid Catalysis: Biocidal Activity

Irene Torres-García[1], José F. Quílez del Moral[2], Alejandro F. Barrero[2], Azucena González-Coloma[3], María Fe Andrés[3], José L. López-Pérez[4,5], Miriam Álvarez-Corral[1], Ignacio Rodríguez-García[1] and Manuel Muñoz-Dorado[1]*

1 Organic Chemistry, University of Almería, ceiA3, 04120 Almería, Spain; itg491@ual.es; malvarez@ual.es; irodrigu@ual.es
2 Organic Chemistry, Institute of Biotechnology, University of Granada, 18071Granada, Spain; jfquilez@ugr. es; afbarre@ugr.es
3 Institute of Agricultural Sciences – CCMA, CSIC, 28006 Madrid, Spain; azu@ica.csic.es; mafay@ica.csic.es
4 Pharmaceutical Chemistry, CIETUS, IBSAL, University of Salamanca, 37007 Salamanca, Spain; lopez@usal.es
5 CIPFAR. Department of Pharmacology, Faculty of Medicine, University of Panamá. Ave. Octavio Mendez Pereira, 3366 Panamá City, Panamá
* Correspondence: mdorado@ual.es

EXPERIMENTAL SECTION 2
General Remarks 2
Computational Chemistry Calculations 2
Biocidal Activity 2
Plant Material 3
Extraction and Isolation 3
Experimental Procedures 4
Characterization of Molecules **9-24** 5
NMR AND IR SPECTRA 10
CRYSTAL DATA AND STRUCTURE REFINEMENT FOR 13 121

EXPERIMENTAL SECTION

General Remarks

NMR spectra were recorded on Bruker Nanobay Avance III HD 600 MHz spectrometer (magnetic field: 14.09 Teslas; probes: QCI-P CryoProbe™ (5mm quad probe with reverse sensing) ^{1}H, ^{13}C, ^{31}P, ^{15}N; gradients in the Z axis; thermostatted automatic changer). Proton-decoupled ^{13}C NMR and DEPT-135 were measured in all cases. HMBC, edited HSQC, COSY, TOCSY and NOESY experiments were used for the complete signal assignation. Chemical shifts (δ) are expressed in ppm and coupling constants (*J*) in hertz (Hz). Chemical shifts are reported using $CDCl_3$ as internal reference. IR Spectra were recorded on a Bruker Alpha spectrometer. Mass spectra were recorded on a Waters Xevo by LC-QTof-MS by electrospray ionization. X-ray structure analyses were performed on a Bruker AXS Smart APEX diffractometer. Using Olex2 [23], the structure was solved with the SHELXT [24] structure solution program using Intrinsic Phasing and refined with the SHELXL [25] refinement package using Least Squares minimization. All reactions were monitored by thin-layer chromatography (TLC) carried out on 0.2 mm DC-Fertigfolien Alugram® XtraSil G/UV254 silica gel plates. The TLC plates were visualized with UV light and 7% phosphomolybdic acid/heat. Flash chromatography was performed on silica gel 60 (0.04–0.06 mm) using different eluents depending on the compound to be separated. Commercially available chemicals such as silicotungstic acid hydrate, indium chloride and fluorosulfonic acid were obtained from Aldrich Chemical Co., Alfa Aesar used as received. Zeolite Y-CBV720 was supplied by the "Instituto Universitario Mixto de Tecnología Química (ITQ), Universidad de Valencia". Reactions were performed under nitrogen atmosphere, using oven-dried glassware in all cases. Additionally, anhydrous CH_2Cl_2 was distilled from CaH_2 under a nitrogen atmosphere.

Computational Chemistry Calculations

Computational calculations were performed with Spartan'20 (Wavefunction Inc., Irvine, CA, USA). To obtain the ^{13}C NMR data by computational calculation, the automated protocol implemented in Spartan'20 has been followed [26]. This protocol consists of five steps: (1) systematic conformational search using MMFF molecular mechanics, eliminating duplicate conformers and those with energy 40 kJ/mol above the global minimum, (2) geometric calculation using HF/3-21G, also eliminating duplicate conformers and those with energy higher than 40 kJ/mol above the global minimum; (3) energy calculation with the ωB97X-D/6-31G* model and removal of conformers above 15 kJ/mol with respect to the global minimum, (4) geometric calculation with the ωB97X-D/6-31G* model and removal of conformers with energies higher than 10 kJ/mol from that of the global minimum, (5) energy calculation with the ωB97X-V/6-311+G(2df,2p)[6-311G*], and finally (6) the NMR calculation (following calculation of Boltzmann weights for conformationally flexible molecules) using the ωB97X-D/6-31G* method that has been corrected empirically based on the comparison of calculated and experimental ^{13}C shifts for ~2000 rigid molecules. These corrections are on the order of 1−3 ppm.

Biocidal Activity

S. littoralis, *M. persicae* and *R. padi* colonies were maintained at ICA-CSIC, reared on an artificial diet, bell pepper (*Capsicum annuum*) and barley (*Hordeum vulgare*) plants, respectively, and kept at 22 ± 1ºC and >70% RH, with a photoperiod of 16:8 h (L:D) in a custom-made walk-in growth chamber. The bioassays were conducted as described [27]. The upper surface of *C. annuum* and *H. vulgare* leaf disks or fragments (1.0 cm^2) were treated with 10 µL of the test substance. The products were tested at dose of 5 and 2.5 mg/mL. A total of 5 to 7 Petri dishes or 20 ventilated square plastic boxes (2 × 2 cm) with 2 sixth-instar *S. littoralis* larvae (> 24 h after molting) or 10 apterous aphid adults (24–48 h old) each were allowed to feed in a growth chamber (until 75% larval consumption of control disks or 24 h for aphids, environmental conditions as above). Each experiment was repeated 2–3 times. Feeding inhibition or aphid settling was calculated by measuring the disk surface consumption (digitalized with https://imagej.nih.gov/ij/)[28] or by counting the number of aphids on each leaf fragment. Feeding/settling inhibition (%FI or %SI) was calculated as % FI/SI = [1−(T/C) x 100], where T and C represent feeding/settling on treated and control leaf disks, respectively. The antifeedant effects (% FI/SI) were analyzed for significance by the nonparametric Wilcoxon paired signed-rank test comparing the consumption/settling between the treatment and control leaf disks. Compounds with an FI/SI > 60% were further tested in a dose-response experiment (1:2 serial dilutions to cover a range of activities between 100 and <50% feeding inhibition with a minimum

of 3 doses) to calculate their effective dose LC_{50} (dose to give a 50% settling reduction) from linear regression analysis (% FI/SI on Log-dose, STATGRAPHICS Centurion XVI, version 16.1.02).

Experiments for phytotoxic evaluation were conducted with *Lactuca sativa*, and *Lolium perenne* seeds (40 seeds/test) in 12-well microplates, as described previously [29]. The compounds were tested at concentrations of 5 mg/mL. Juglone (97%; Sigma) was included as positive control (0.1 mg/mL), resulting in 100% germination inhibition. Germination was monitored for six (*L. sativa*) or seven days *(L. perenne*), and the root length (25 plants randomly selected and digitalized) was measured (ImageJ, http//rsb.info.nih.gov./ij/) at the end of the experiment. A nonparametric analysis of variance (ANOVA) was performed on root/leaf length data.

Hyalomma lusitanicum engorged female ticks were collected in central Spain (Finca La Garganta, Ciudad Real) from their host (deer) and *Rhipicephalus bursa* adults were collected from the vegetation. Adults of *R. bursa* were fed in vitro to obtain engorged females [30]. Both engorged females were maintained at 22–24 ºC and 70% RH until oviposition and egg hatching. Resulting larvae (4–6 weeks old) were used for the bioassays. Briefly, 50 µL of test solution were added to 25 mg of powdered cellulose at different concentrations (initial concentration of 10 mg/mL) and the solvent was evaporated. For each test, three replicates with 20 larvae each were used. Dead ticks were counted after 24 h of contact with the treated cellulose at the environmental conditions described, using a binocular magnifying glass. The larvicidal activity data are presented as percent mortality corrected according to Schneider–Orelli's formula. Effective lethal doses (LD50) were calculated by Probit Analysis (5 serial dilutions, STATGRAPHICS Centurion XVI, version 16.1.02, Statgraphics Technologies, Inc., P.O. Box 134, The Plains, Virginia 20198, USA).

A *Meloidogyne javanica* population maintained on *Lycopersicon esculentum* plants (var. Marmande) in pot cultures at 25 ± 1ºC with 70% relative humidity was used in this work. Egg masses of *M. javanica* were hand-picked from infected tomato roots. Second stage juveniles (J2) were obtained from hatched eggs by incubating egg masses in a water suspension at 25ºC for 24 h. Bioassays were performed in 96-well plates (BD Falcon, Plants 2022, 11, 418 8 of 9 San Jose, CA, USA) as described by Andrés *et al.* [31]. Compounds were dissolved in water with a 5% DMSO-Tween solution (0.5% Tween 20 in DMSO), and 5 µL of this solution was added to 95 µL of water containing 90–100 nematodes to obtain an initial concentration of 1 mg/mL per well. Treatments were replicated 4 times. As a control, 4 wells were filled with 95 µL of solvent. The plates were covered to prevent evaporation and were maintained in the dark at 25 ◦C. After 72 h, the dead J2 were counted under a binocular microscope. The nematicidal activity data were presented as percent dead J2s and corrected according to Schneider-Orelli's formula [32].

Plant Material

Santolina viscosa Lag. (Compositae) was collected in Los Yesares (Tabernas), Almería, Spain, in July 2020. It was identified in "Departamento de Biología Vegetal, Universidad de Almería".

Extraction and Isolation

The air-dried material (658 g) was extracted by immersion in Et_2O for 24h, yielding in 70 g of extract. The ethereal extract was dissolved in 175 mL of hot chloroform and 875 mL of boiling methanol was slowly added. The solution was allowed to cool to room temperature and then further cooled to -4 ºC for 4 days. Vacuum filtration produced 2 g of solid residue (waxes) and 60 g of dewaxed extract. A portion of 34 g was defatted, dissolved in Et_2O, and extracted with 1N NaOH solution to yield 22 g of a neutral fraction. The aqueous phase was acidified with 2N HCl (pH 2) and extracted with Et_2O (x3), obtaining 11 g of dewaxed acidic extract. **Acidic fraction:** a portion of 5 g of the acidic extract was esterified witg $CsCO_3$ (13 g) and CH_3I (3 mL) in DMF (60 mL), resulting in 4g of esterified acidic extract. The acid-esterified fraction underwent column chromatography on silica gel using a hexane/AcOEt mixture. Three main compounds (**1**-**3**) were obtained. Fraction 1 [hexane/AcOEt, (7:3)] consisted in 500 mg of **1** and fraction 2, a mixture of 1 g of **2** and **3**. F2 was rechromatographed [hexane/AcOEt, (8:2)] to obtain 500 mg of **2** and 300 mg of a mixture **2** and **3** (2:1). **Neutral fraction:** a portion of 3 g of the neutral fraction underwent column chromatography on silica gel using hexane/AcOEt/MeOH mixtures of increasing polarity as eluents. Four main compounds (**4**-**7**) with longipin-9-ene structure were isolated. Fraction 1 [hexane/AcOEt, (7:3)] consisted in 402 mg of **4**. Fraction 2 [hexane/AcOEt, (6:4)] consisted in 907 mg of **5**. Fraction 3 [hexane/AcOEt, (6:4)] consisted in 460 mg of **6**. The most polar compound in the extract (**7**) was obtained using MeOH as eluent (503 mg). The isolated longipinenes **1**-**7** match those described by Barrero *et al.* [20,21].

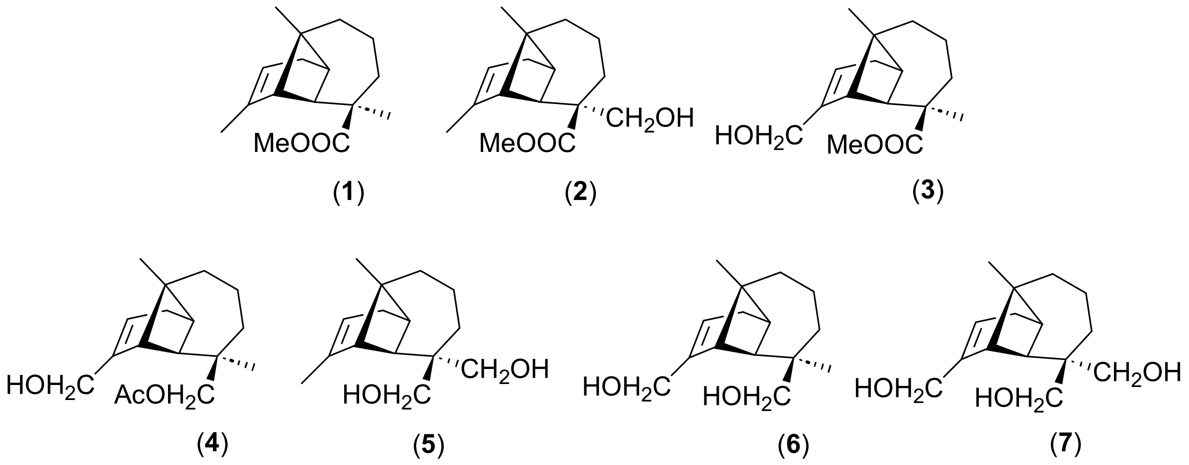

Figure I. Seven main compounds with Δ^9-longipinene structures (**1**-**7**) of which longipinene acids were isolated as methyl esters (**1**-**3**).

Experimental procedures

General procedure for treatment of longipinenes 1, 2 and 5 with zeolite Y-CBV720

Under N_2 atmosphere, zeolite Y-CBV720 (50% by weight of the starting material) was added to a solution of longipinene (1 eq.) in anhydrous CH_2Cl_2 (10 mL/mmol). The mixture was stirred overnight at reflux. The solvent was removed under reduced pressure and the crude reaction mixture was passed through a short plug of silica gel and celite (4:1), which was washed with CH_2Cl_2. Finally, the resulting crude from each reaction was concentrated under reduced pressure and purified through flash silica gel column chromatography (hexane/EtOAc mixtures) to yield pure products **9**, **10**, **12**, **16** and **17**. Yields are reported in **Table 1**.

General procedure for treatment of longipinenes 1, 2 and 5 with silicotungstic acid hydrate ($H_6SiW_{12}O_{41}$)

Under N_2 atmosphere, $H_6SiW_{12}O_{41}$ (0.05 eq.) was added to a solution of longipinene (1 eq.) in anhydrous CH_2Cl_2 (10 mL/mmol). The mixture was stirred overnight at room temperature. A solution of Et_3N (0.25 mL/mmol) in ethyl acetate (0.06 mL/mmol) was added, and the mixture was diluted with water. The aqueous phase was extracted with ethyl acetate (x3). The combined organic layer was dried over anhydrous $MgS0_4$ and the solvent was removed. In this manner, products **10**, **12** and **13** were obtained after purification through flash silica gel column chromatography (hexane/EtOAc mixtures) from their respective processes. Yields are reported in **Table 1**.

General procedure for treatment of natural longipinenes 1, 2 and 5 with indium chloride ($InCl_3$)

Under N_2 atmosphere, $InCl_3$ (0.2 eq.) was added to a solution of longipinene (1 eq.) in anhydrous CH_2Cl_2 (0.03 mM) was added. The mixture was stirred overnight at reflux. The solvent was removed under reduced pressure and crude reaction mixture was passed through a short plug of silica gel and celite (4:1), which was washed with CH_2Cl_2. Finally, the resulting crude from each reaction was concentrated under reduced pressure and purified through flash silica gel column chromatography. Products **9**, **11** and **13**-**18** were obtained and their yields are reported in **Table 1.**

General procedure for treatment of natural longipinenes 1, 2 and 5 with fluorosulfonic acid (HSO_3F)

Under N_2 atmosphere, HSO_3F (1 eq.) was added to a solution of longipinene (1 eq.) in anhydrous nitropropano:CH_2Cl_2 (12:1) (10 mL/mmol) at -78ºC. The mixture was stirred at -78ºC for 30 min. A solution of Et_3N (0.25 mL/mmol) in ethyl acetate (0.06 mL/mmol) was added, and the mixture was diluted with water. The aqueous phase was extracted with ethyl acetate (x3). The combined organic layer was dried over anhydrous $MgS0_4$ and the solvent was removed. Products **9**, **12**, **13**, **17** and **18** were obtained from each reaction and purified through flash silica gel column chromatography (hexane/EtOAc mixtures). Yields are reported in **Table 1**.

Protection of hydroxyl groups with MOM from longipinene 5

Under N_2 atmosphere, DIPEA (0.62 g, 4.8 mmol, 4 eq.) and MOMBr (0.40 g, 2.88 mmol, 2.4 eq) was added to a solution of **5** (0.29 g, 1.20 mmol, 1 eq.) in anhydrous CH_2Cl_2 (6 mL) at 0ºC. The mixture was stirred at room temperature for 3h. The reaction mixture was diluted with water and extracted with ethyl acetate (x3). The combined organic layer was washed with brine and dried over anhydrous $MgS0_4$. The resulting crude was concentrated under reduced pressure and purified through flash silica gel column chromatography using a hexane/AcOEt mixture (8:2) as the solvent. An isomeric mixture with partially protected hydroxyl groups (**19a**/**19b**) (0.06 g, 0.24 mmol, 20%) and compound **5** with fully protected hydroxyl groups (0.23 g, 0.72 mmol, 60%) were obtained as colorless syrup.

Treatment of the isomeric mixture 19a/19b with indium chloride ($InCl_3$)

Under N_2 atmosphere, $InCl_3$ (0.01 g, 0.04 mmol, 0.2 eq.) was added to a solution of **19a**/**19b** (0.06 g, 0.21 mmol, 1 eq.) in anhydrous CH_2Cl_2 (9 mL). The mixture was stirred overnight at reflux. The solvent was removed under reduced pressure, and the crude reaction mixture was passed through a short plug of silica gel and celite (4:1), which was washed with CH_2Cl_2. Finally, the resulting crude was concentrated under reduced pressure and purified through flash silica gel column chromatography using a hexane/AcOEt mixture (8:2) as the solvent. Compound **20** was obtained as a colorless syrup (0.04 g, 0.16 mmol, 76%).

Epoxidation reaction of longipinenes 2 and 5

Under N_2 atmosphere, *m*CPBA (2 eq.) was added to a solution of longipinene (1 eq.) in anhydrous CH_2Cl_2 (20 mL/mmol). The mixture was stirred at 0ºC for 2h. To the reaction mixture, 10% $Na_2S_2O_3$, saturated $NaHCO_3$, and ethyl acetate were added and stirred for 30 min. The organic layer was washed with saturated $NaHCO_3$ and brine and dried over anhydrous $MgS0_4$. From longipinene **2**, epoxide **21** was obtained as colorless syrup (0.17g, 0.61 mmol, 82%). From longipinene **5**, epoxide **22** was obtained as colorless syrup (1.02 g, 4.04 mmol, 84%).

Treatment of epoxides 21 and 22 with indium chloride ($InCl_3$)

Under N_2 atmosphere, $InCl_3$ (0.2 eq.) was added to a solution of epoxide (1 eq.) in anhydrous CH_2Cl_2 (0.03 mM) at 0ºC. The mixture was stirred for 1h. The solvent was removed under reduced pressure, and the crude reaction mixture was passed through a short plug of silica gel and celite (4:1), which was washed with CH_2Cl_2. Finally, the resulting crude was concentrated under reduced pressure and purified through flash silica gel column chromatography using a hexane/AcOEt mixture as the solvent. From epoxide **21**, *seco*longibornene aldehyde **23** was obtained (0.12g, 0.44 mmol, 53%). From epoxide **22**, *seco*longibornene aldehyde **24** was obtained (0.21g, 0.83 mmol, 51%).

Treatment of epoxides with zeolite Y-CBV720

Under N_2 atmosphere, zeolite Y-CBV-720 (50% by weight of the starting material) was added to a solution of longipinene (1 eq.) in anhydrous CH_2Cl_2 (10 mL/mmol). The mixture was stirred for 5h at room temperature. The solvent was removed under reduced pressure and the crude reaction mixture was passed through a short plug of silica gel and celite (4:1), which was washed with CH_2Cl_2. Finally, the resulting crude from each reaction was concentrated under reduced pressure and purified through flash silica gel column chromatography (hexane/EtOAc mixtures). From epoxide **21**, *seco*longibornene aldehyde **23** was obtained (0.13g, 0.49 mmol, 57%). From epoxide **22**, *seco*longibornene aldehyde **24** was obtained (0.22g, 0.88 mmol, 54%).

Characterization of molecules 9-24

Methyl 14-hydroxylongipin-9(12)-en-15-oate (9). From longipinene **2**. IR (ATR) ν (cm^{-1}) 3494, 2938, 1707, 1436, 1195, 1059, 889, 840. ^1H NMR (600 MHz, $CDCl_3$) δ (ppm) 4.72 (1H, s, H-12a), 4.49 (1H, s, H-12b), 3.68 (2H, m, H-14), 3.64 (3H, s, $COOCH_3$), 2.35 (1H, d, *J*= 4.8 Hz, H-8), 2.09 (1H, d, *J*= 13.2 Hz, H-5a), 2.05 (1H, m, H-1), 1.80 (1H, s, H-7), 1.70 (1H, m, H-10a), 1.67 (1H, m, H-3a), 1.64 (1H, m, H-11a), 1.58 (1H, m, H-3b), 1.56 (1H, d, *J*= 14.4 Hz, H-5b), 1.52 (1H, m, H-4a), 1.44 (1H, m, H-4b), 1.38 (1H, m, H-11b), 1.10 (1H, ddd, *J*= 12.0, 9.0, 5.8 Hz, H-10b), 0.95 (3H, s, H-13). $^{13}C\{^1H\}$ NMR (151 MHz, $CDCl_3$) δ (ppm) 177.1 (C, C-15), 166.5 (C, C-9), 100.0 (CH_2, C-12), 66.6 (CH_2, C-14), 53.4 (CH, C-7), 52.0 (C, C-6), 51.7 (CH_3, $COOCH_3$), 49.3 (CH, C-8), 45.4 (CH, C-1), 44.2 (C, C-2), 42.8 (CH_2, C-3), 29.8 (CH_3, C-13), 29.1 (CH_2, C-10), 25.2 (CH_2, C-11), 24.9 (CH_2, C-5), 20.0 (CH_2, C-4). HRESIMS *m/z* 265.1797 $[M + H]^+$ calcd for $[C_{16}H_{24}O_3 + H]^+$ 265.1804.

Longipin-9(12)-ene-14,15-diol (10). From longipinene **5.** IR (ATR) ν (cm^{-1}) 3333, 2924, 1454, 1376, 1032, 951, 872, 817. ^{1}H NMR (600 MHz, $CDCl_3$) δ (ppm) 4.73 (1H, s, H-12a), 4.46 (1H, s, H-12b), 3.58 (2H, m, H-14), 3.55 (2H, s, H-15), 2.56 (1H, d, *J*= 4.8 Hz, H-8), 2.06 (1H, d, *J*= 3.6 Hz, H-1), 1.72 (1H, m, H-10a), 1.65 (1H, m, H-11a), 1.59 (1H, s, H-7), 1.56 (1H, m, H-3b), 1.45 (1H, m, H-4a), 1.43 (1H, m, H-4b), 1.39 (1H, m, H-11b), 1.32 (1H, m, H-5b), 1.14 (1H, ddd, *J*= 12.0, 9.0, 5.8 Hz, H-10b), 0.97 (3H, s, H-13). ^{13}C{^{1}H} NMR (151 MHz, $CDCl_3$) δ (ppm) 167.1 (C, C-9), 99.6 (CH_2, C-12), 70.6 (CH_2, C-15), 69.8 (CH_2, C-14), 52.5 (CH, C-7), 47.2 (CH, C-8), 45.5 (CH, C-1), 44.4 (C, C-2), 42.9 (CH_2, C-3), 41.4 (C, C-6), 29.9 (CH_3, C-13), 29.6 (CH_2, C-10), 27.0 (CH_2, C-5), 25.2 (CH_2, C-11), 20.1 (CH_2, C-4).

Methyl 14-hydroxylongiborn-7(8)-en-15-oate (11). From longipinene **2.** IR (ATR) ν (cm^{-1}) 3465, 1720, 1435, 1263, 1197, 1056. ^{1}H NMR (600 MHz, $CDCl_3$) δ (ppm) 5.18 (1H, m, H-8), 3.74 (1H, d, *J*= 11.4 Hz, H-14a), 3.67 (1H, d, *J*= 11.4 Hz, H-14b), 3.60 (3H, s, $COOCH_3$), 2.22 (1H, m, H-10a), 1.99 (1H, m, H-10b), 1.79 (1H, m, H-5a), 1.74 (1H, br s, H-1), 1.72 (1H, m, H-4a), 1.66 (1H, m, H-3a), 1.53 (1H, m, H-11a), 1.50 (1H, m, H-11b), 1.44 (1H, m, H-3b), 1.33 (1H, ddd, *J*= 13.2, 10.8, 6.6 Hz, H-5b), 1.11 (1H, m, H-4b), 0.99 (3H, s, H-12), 0.90 (3H, s, H-13). ^{13}C{^{1}H} NMR (151 MHz, $CDCl_3$) δ (ppm) 176.4 (C, C-15), 154.0 (C, C-7), 112.2 (CH, C-8), 67.9 (CH_2, C-14), 51.8 (C, C-6), 51.3 (CH_3, $COOCH_3$), 49.9 (C, C9), 46.5 (CH, C1), 41.3 (C, C2), 40.3 (CH_2, C-11), 29.3 (CH_2, C-4), 29.1 (CH_3, C-13), 25.5 (CH_2, C-5), 25.4 (CH_3, C-12), 24.2 (CH_2, C-3), 23.2 (CH_2, C-10).

Longibornan-15,8-olide (12). From longipinene **1.** IR (ATR) ν (cm^{-1}) 2926, 1748, 1449, 1376, 1102, 1009, 732, 556. ^{1}H NMR (600 MHz, $CDCl_3$) δ (ppm) 4.24 (1H, d, *J*= 7.8 Hz, H-8), 2.19 (1H, d, *J*= 7.8 Hz, H-7), 2.01 (1H, ddd, *J*= 16.2, 11.4, 4.8 Hz, H-5a), 1.94 (1H, d, *J*= 4.8 Hz, H-1), 1.80 (1H, ddd, *J*= 16.9, 13.2, 4.2 Hz, H-3a), 1.71 (1H, m, H-11a), 1.66 (1H, m, H-5b), 1.63 (2H, m, H-4), 1.46 (1H, m, H-10a), 1.14 (3H,s, H-14), 1.00 (1H, m, H-10b), 1.00 (1H, m, H-11b), 0.97 (3H, s, H-12), 0.92 (1H, dt, *J*= 16.9, 4.2 Hz, H-3b), 0.80 (3H, s, H-13). ^{13}C{^{1}H} NMR (151 MHz, $CDCl_3$) δ (ppm) 185.3 (C, C-15), 88.6 (CH, C-8), 54.9 (CH, C-7), 49.9 (C, C-9), 49.4 (C, C-2), 44.1 (C, C-6), 44.0 (CH, C-1), 33.4 (CH_2, C-5), 32.7 (CH_2, C-10), 29.7 (CH_3, C-14), 27.9 (CH_2, C-3), 27.1 (CH_2, C-11), 21.1 (CH_3, C-13), 20.0 (CH_2, C-4), 11.0 (CH_3, C-12).

14-Hydroxylongibornan-15,8-olide (13). From longipinene **2.** IR (ATR) ν (cm^{-1}) 3399, 2958, 1730, 1451, 1377, 1323, 1196, 1072, 1031, 972, 561, 449. ^{1}H NMR (600 MHz, $CDCl_3$) δ (ppm) 4.24 (1H, d, *J*= 8.4 Hz, H-8), 3.55 (1H, d, *J*= 8.4 Hz, H-14a), 3.33 (1H, d, *J*= 10.2 Hz, H-14b), 2.45 (1H, d, *J*= 8.4 Hz, H-7), 1.98 (1H, m, H-5a), 1.91 (1H, d, *J*= 4.2 Hz, H-1), 1.77 (1H, m, H-3a), 1.71 (1H, m, H-11a), 1.65 (1H, m, H-4a), 1.58 (1H, m, H-5b), 1.47 (1H, dt, *J*= 11.4, 3.0 Hz, H-10a), 1.07 (1H, m, H-11b), 1.02 (1H, m, H-10b), 0.96 (3H, s, H-12), 0.93 (1H, m, H-3b), 0.80 (3H, s, H-13). ^{13}C{^{1}H} NMR (151 MHz, $CDCl_3$) δ (ppm) 184.2 (C, C-15), 89.8 (CH, C-8), 69.8 (CH_2, C-14), 50.8 (C, C-6), 50.2 (CH, C-7), 50.2 (C, C-2), 49.3 (C, C9), 43.7 (CH, C-1), 32.7 (CH_2, C-10), 28.1 (CH_2, C-3), 27.7 (CH_2, C-5), 27.0 (CH_2, C-11), 21.0 (CH_3, C-13), 19.4 (CH_2, C-4), 11.0 (CH_3, C-12). HRESIMS *m/z* 251.1648 $[M + H]^+$ calcd for $[C_{15}H_{22}O_3 + H]^+$ 251.1647. Crystal Data for $C_{15}H_{22}O_3$ (M =250.32 g/mol): orthorhombic, space group $P2_12_12_1$ (no. 19), a = 8.1459(2) Å, b = 12.0831(4) Å, c = 13.4903(4) Å, V = 1327.82(7) Å^3, Z = 4, T = 155.70 K, μ(CuKα) = 0.685 mm^{-1}, *Dcalc* = 1.252 g/cm^3, 16026 reflections measured (12.694º≤ 2Θ ≤ 143.86º), 2585 unique (R_{int} = 0.0402, R_{sigma} = 0.0282) which were used in all calculations. The final R_1 was 0.0341 (I > 2σ(I)) and wR_2 was 0.0927.

Methyl 8,15-bisepoxylongibornan-14-oate (14). From longipinene **2.** IR (ATR) ν (cm^{-1}) 2950, 2879, 1729, 1451, 1218, 1087, 1010. ^{1}H NMR (600 MHz, $CDCl_3$) δ (ppm) 3.75 (1H, d, *J*= 1.2 Hz, H-8), 3.73 (2H, m, H-14), 3.61 (3H, s, $COOCH_3$), 2.38 (1H, d, *J*= 8.4 Hz, H-7), 1.94 (1H, d, *J*= 4.2 Hz, H-1), 1.83 (1H, m, H-4a), 1.74 (2H, m, H-5), 1.70 (1H, m, H-11a), 1.59 (1H, ddd, *J*= 15.0, 5.4, 1.8 Hz, H-3a), 1.47 (1H, m, H-4b), 1.39 (1H, m, H-10a), 1.36 (1H, m, H-3b), 1.00 (1H, ddd, *J*= 13.2, 9.6, 4.2 Hz, H-11b), 0.94 (1H, ddd, *J*= 12.6, 9.0, 3.6 Hz, H-10b), 0.91 (3H, s, H-12), 0.78 (3H, s, H-13). ^{13}C{^{1}H} NMR (151 MHz, $CDCl_3$) δ (ppm) 176.7 (C, C-15), 91.7 (CH, C-8), 74.5 (CH_2, C-14), 55.1 (CH, C-7), 55.0 (C, C-6), 52.0 (CH_3, $COOCH_3$), 51.3 (C, C-2), 48.6 (C, C-9), 44.2 (CH, C-1), 35.2 (CH_2, C-3), 33.7 (CH_2, C-10), 32.7 (CH_2, C-5), 28.5 (CH_2, C-11), 23.8 (CH_3, C-13), 22.7 (CH_2, C-4), 10.9 (CH_3, C-12). HRESIMS *m/z* 265.1790 $[M + H]^+$ calcd for $[C_{16}H_{24}O_3 + H]^+$ 265.1804.

8,15-Bisepoxylongibornan-14-ol (15). From longipinene **5.** IR (ATR) ν (cm^{-1}) 3373, 2927, 2866, 1450, 1226, 1043, 1001, 96,7 624. ^{1}H NMR (600 MHz, $CDCl_3$) δ (ppm) 3.71 (1H, d, *J*= 8.8 Hz, H-8), 3.61 (1H, d, *J*= 8.8 Hz, H-15a), 3.44 (1H, d, *J*= 10.6 Hz, H-14a), 3.39 (1H, d, *J*= 10.6 Hz, H-14b), 3.36 (1H, dd, *J*= 8.8, 2.3 Hz, H-15b), 1.87 (1H, d, *J*= 8.8 Hz, H-7), 1.81 (1H, m, H-4a), 1.76 (1H, d, *J*= 4.2 Hz, H-1), 1.69 (1H, m, H-11a), 1.65 (1H, m, H-3a), 1.54 (1H, m, H-5a), 1.48 (1H, m, H-4b), 1.40 (1H, dt, *J*= 13.1, 2.3 Hz, H-5b), 1.36 (1H, m, H-10a), 1.30 (1H, ddd, *J*= 15.2, 10.5, 5.9 Hz, H-3b), 0.94 (1H, m, H-11b), 0.91 (3H, s, H-12), 0.89 (1H, m, H-10b), 0.76 (3H, s, H-13). ^{13}C{^{1}H} NMR (151 MHz, $CDCl_3$) δ (ppm) 92.1 (CH, C-8), 75.9

(CH_2, C-15), 70.3 (CH_2, C-14), 55.0 (CH, C-7), 50.9 (C, C-2), 50.0 (C, C-9), 48.5 (C, C-6), 44.1 (CH, C-1), 34.9 (CH_2, C-3), 33.8 (CH_2, C-10), 31.3 (CH_2, C-5), 28.6 (CH_2, C-11), 23.7 (CH_3, C-13), 22.5 (CH_2, C-4), 11.1 (CH_3, C-12).

Himachal-8(9)-en-15,1-olide (16). From longipinene **1.** IR (ATR) ν (cm^{-1}) 2921, 1760, 1452, 1377, 1210, 1183, 1113, 948. ^{1}H NMR (600 MHz, $CDCl_3$) δ (ppm) 5.15 (1H, br s, H-8), 2.79 (1H, br s, H-7), 2.30 (1H, ddd, *J*= 13.9, 4.6, 2.2 Hz, H-11a), 2.01 (1H, br t, *J*= 15.3 Hz, H-10a), 1.87 (1H, m, H-5a), 1.72 (1H, m, H-2), 1.71 (1H, m, H-3a), 1.69 (1H, m, H-10b), 1.65 (3H, br s, H-12), 1.58 (2H, m, H-4), 1.57 (1H, m, H-5b), 1.40 (1H, quin, *J*= 8.3 Hz, H-3b), 1.32 (1H, dt, *J*= 13.1, 5.0 Hz, H-11b), 1.09 (3H, s, H-14), 1.02 (3H, d, *J*= 7.2 Hz, H-13). $^{13}C\{^1H\}$ NMR (151 MHz, $CDCl_3$) δ (ppm) 183.4 (C, C-15), 135.7 (C, C-9), 119.6 (CH, C-8), 85.4 (C, C-1), 48.4 (C, C-6), 45.2 (CH, C-7), 42.4 (CH, C-2), 41.7 (CH_2, C-5), 32.4 (CH_2, C-3), 31.8 (CH_2, C-11), 24.7 (CH_2, C-10), 23.8 (CH_3, C-12), 22.5 (CH_3, C-14), 22.0 (CH_2, C-4), 16.2 (CH_3, C-13).

Himachal-8(9)-en-15,2-olide (17). From longipinene **1.** IR (ATR) ν (cm^{-1}) 2932, 1733, 1453, 1378, 1274, 1210, 1164, 1086, 965. ^{1}H NMR (600 MHz, $CDCl_3$) δ (ppm) 5.37 (1H, s, H-8), 2.33 (1H, d, *J*= 12.0 Hz, H-7), 2.24 (1H, dt, *J*= 12.0, 6.6 Hz, H-1), 1.96 (1H, dt, *J*= 15.6, 3.6 Hz, H-10a), 1.88 (1H, m, H-10b), 1.83 (1H, m, H-3a), 1.77 (1H, m, H-4a), 1.73 (3H, s, H-12), 1.71 (1H, m, H-3b), 1.68 (1H, m, H-11a), 1.64 (1H, m, H-5a), 1.48 (1H, m, H-5b), 1.40 (1H, m, H-4b), 1.37 (3H, s, H-13), 1.28 (1H, dd, *J*= 12.0, 4.8 Hz, H-11b), 1.23 (3H, s, H-14). $^{13}C\{^1H\}$ NMR (151 MHz, $CDCl_3$) δ (ppm) 178.5 (C, C-15), 138.2 (C, C-9), 119.1 (CH, C-8), 83.8 (C, C-2), 44.4 (C, C-6), 41.3 (CH, C-1), 39.0 (CH, C-7), 34.6 (CH_2, C-3), 31.7 (CH_2, C-5), 29.6 (CH_3, C-13), 29.0 (CH_2, C-10), 24.4 (CH_3, C-14), 24.2 (CH_3, C-12), 23.7 (CH_2, C-11), 20.5 (CH_2, C-4).

Methyl 2,14-bisepoxyhimachal-8(9)-en-15-oate (18). From longipinene **2**. IR (ATR) ν (cm^{-1}) 2927, 2876, 1726, 1439, 1372, 1217, 1037, 835. ^{1}H NMR (600 MHz, $CDCl_3$) δ (ppm) 5.25 (1H, br s, H-8), 3.95 (1H, dd, *J*= 10.2, 1.8 Hz, H-14a), 3.81 (1H, d, *J*= 10.2 Hz, H-14b), 3.62 (3H, s, $COOCH_3$), 2.85 (1H, d, *J*= 10.8 Hz, H-1), 2.17 (1H, dd, *J*= 10.8, 4.5 Hz, H-7), 2.13 (1H, m, H-5a), 1.88 (1H, m, H-5b), 1.87 (1H, m, H-10a), 1.83 (1H, m, H-10b), 1.76 (1H, m, H-3a), 1.66 (3H, s, H-12), 1.63 (1H, m, H-4a), 1.56 (1H, m, H-3b), 1.50 (1H, m, H-4b), 1.22 (2H, m, H-11), 1.14 (3H, s, H-13). $^{13}C\{^1H\}$ NMR (151 MHz, $CDCl_3$) δ (ppm) 175.9 (C, C-15), 136.8 (C, C-9), 121.6 (CH, C-8), 76.5 (C, C-2), 71.0 (CH_2, C-14), 52.2 (CH_3, $COOCH_3$), 48.9 (C, C-6), 40.5 (CH, C-7), 37.5 (CH_2, C-3), 36.8 (CH, C-1), 31.2 (CH_2, C-5), 30.4 (CH_3, C-13), 28.9 (CH_2, C-10), 24.1 (CH_2, C-11), 24.0 (CH_3, C-12), 19.4 (CH_2, C-4). HRESIMS *m/z* 265.1795 $[M + H]^+$ calcd for $[C_{16}H_{24}O_3 + H]^+$ 265.1804.

15-(Methoxymethoxy)longinin-9(10)-en-14-ol (19a) / 14-(methoxymethoxy)longinin-9(10)-en-15-ol (19b). IR (ATR) ν (cm^{-1}) 3448, 2915, 2837, 1738, 1442, 1373, 1213, 1147, 1108, 1038. ^{1}H NMR (300 MHz, $CDCl_3$) δ (ppm) 5.21 (2H, s, H-10), 4.62 (2H, s, OCH_2O), 4.60 (2H, s, OCH_2O), 3.48 (4H, m, CH_2O), 3.52 (4H, m, CH_2OH), 3.37 (3H, s, OCH_3), 3.36 (3H, s, OCH_3), 2.25 (4H, m), 2.14 (2H, m), 2.03 (2H, m), 1.80 (2H, br s), 1.67 (14H, m), 1.43 (4H, m), 0.86 (6H, s, H-13). $^{13}C\{1H\}$ NMR (75 MHz, $CDCl_3$) δ (ppm) 147.3-147.1 (C, C9), 117.6-117.3 (CH, C10), 96.8-96.7 (CH_2, OCH_2O), 72.9-72.8 (CH_2, CH_2O), 67.6 (CH_2, CH_2OH), 55.4-55.3 (CH_3, OCH_3), 49.5 (CH), 45.7 (CH), 40.7 (CH_2), 40.7 (C), 40.3 (C), 39.4 (CH), 34.0 (CH_2), 29.0 (CH_2), 23.4 (CH_3, C13), 22.9-22.8 (CH_3, C12), 20.9 (CH_2).

Compound 20. IR (ATR) ν (cm^{-1}) 2918, 1454, 1174, 1155, 1036, 928. ^{1}H NMR (600 MHz, $CDCl_3$) δ (ppm) 5.16 (1H, br s, H-10), 4.75 (1H, d, *J*= 6.6 Hz, H-16a), 4.69 (1H, d, *J*= 6.6 Hz, H-16b), 3.56 (2H, dd, *J*= 33.6, 10.8 Hz, H14*), 3.46 (2H, dd, *J*= 33.6, 10.8 Hz, H15*), 2.21 (2H, m, H-11), 2.07 (1H, m, H-8), 1.94 (1H, d, *J*= 6.6 Hz, H-7), 1.87 (1H, br s, H-1), 1.61 (3H, d, *J*= 1.8 Hz, H-12), 1.58 (2H, m, H-3), 1.53 (2H, m, H-4), 1.52 (1H, m, H-5a), 1.42 (1H, m, H-5b), 0.80 (3H, s, H-13). $^{13}C\{^1H\}$ NMR (151 MHz, $CDCl_3$) δ (ppm) 147.0 (C, C-9), 117.6 (CH, C-10), 94.3 (CH_2, C-16), 74.3 (CH_2, C-14/15), 50.2 (CH, C-1), 45.5 (CH, C-7), 40.7 (CH_2, C-3), 39.2 (CH, C-8), 35.3 (C, C-6), 34.0 (CH_2, C-11), 31.2 (CH_2, C-5), 23.4 (CH_3, C-13), 22.9 (CH_3, C-12), 20.8 (CH_2, C-4). *Interchangeable signals.

Methyl 9,10 bisepoxy-14-hydroxylongipinan-15-oate (21). IR (ATR) ν (cm^{-1}) 3433, 2920, 1723, 1438, 1204, 1043, 837. ^{1}H NMR (300 MHz, CDCl3) δ (ppm) 3.61 (3H, s, $COOCH_3$), 3.48 (1H, d, *J*= 11.1 Hz, H-14a), 3.41 (1H, d, *J*= 11.1 Hz, H-14b), 3.00 (1H, da, *J*= 3.0 Hz, H-10), 2.38 (1H, s), 2.11 (1H, d, *J*= 5.7 Hz), 1.79 (5H, m), 1.48 (4H, m), 1.29 (3H, s, H-12), 0.86 (3H, s, H-13). $^{13}C\{^1H\}$ NMR (75 MHz, $CDCl_3$) δ (ppm) 176.7 (C, $COOCH_3$), 66.5 (CH2, C-14), 61.9 (C), 56.9 (CH, C-10), 51.8 (CH_3, $COOCH_3$), 51.1 (C), 44.1 (CH), 40.5 (CH2), 43.0 (C), 40.9 (CH), 39.1 (CH), 29.8 (CH2), 29.2 (CH2), 22.2 (CH3, C-12), 21.7 (CH2), 21.6 (CH3, C-13).

9,10-Bisepoxylongipinano-14,15-diol (22). IR (ATR) ν (cm^{-1}) 3376, 2919, 2817, 1455, 1437, 1378, 1278. ^{1}H NMR (300 MHz, CDCl3) δ (ppm) 3.53 (4H, m, H-14, H-15), 3.11 (1H, da, *J*= 3.9 Hz, H-10), 2.14 (1H, s), 2.03-1.91 (4H, m), 1.63 (4H, br s), 1.40 (3H, s, H-12), 1.44 (2H, br s), 0.96 (3H, s, H-13). $^{13}C\{^1H\}$ NMR (75 MHz, $CDCl_3$) δ (ppm) 67.8 (CH2, C-15*), 67.5

(CH2, C-14*), 62.2 (C), 57.7 (CH, C-10), 43.3 (C), 43.2 (CH), 40.9 (CH2), 40.3 (C), 40.0 (CH), 38.4 (CH), 29.3 (CH2), 28.9 (CH2), 22.3 (CH3, C-12), 21.8 (CH3, C-13), 20.9 (CH2). *Interchangeable signals.

Methyl 10-oxo-14-hydroxysecolongifol-8(9)-en-15-oate (23). IR (ATR) ν (cm^{-1}) 3444, 2945, 2912, 1717, 1439, 1216, 1058, 914, 842, 731. ^{1}H NMR (600 MHz, $CDCl_3$) δ (ppm) 9.68 (1H, s, H-10), 5.11 (1H, br t, H-8), 3.61 (3H, s, $COOCH_3$), 3.67 (1H, d, *J*= 11.2 Hz, H-14a), 3.49 (1H, d, *J*= 11.2 Hz, H-14b), 2.49 (2H, m, H-11), 2.39 (1H, m, H-1), 2.39 (1H, m, H-7), 2.08 (1H, m, H-5a), 1.53 (1H, m, H-5b), 1.51 (1H, m, H-3a), 1.51 (1H, m, H-4a), 1.47 (1H, m, H-4b), 1.42 (3H, s, H-12), 1.29 (1H, m, H-3b), 0.88 (3H, s, H-13). ^{13}C{^{1}H} NMR (151 MHz, $CDCl_3$) δ (ppm) 204.4 (CH, C-10), 175.9 (C, C-15), 144.9 (C, C-9), 125.4 (CH, C-8), 70.1 (CH_2, C-14), 54.2 (C, C-6), 52.0 (CH, C-7), 51.5 (CH_3, $COOCH_3$), 51.2 (C, C-2), 47.1 (CH_2, C-11), 42.5 (CH_2, C-3), 42.4 (CH, C-1), 30.7 (CH_2, C-5), 21.2 (CH_2, C-4), 21.1 (CH_3, C-13), 12.4 (CH_3, C-12).

14,15-Dihydroxysecolongifol-8(9)-en-10-al (24). IR (ATR) ν (cm^{-1}) 3333, 2916, 1715, 1438, 1375, 1019, 914, 836. ^{1}H NMR (600 MHz, $CDCl_3$) δ (ppm) 9.73 (1H, br s, H-10), 5.44 (1H, br s, H-8), 3.70 (1H, dd, *J*= 11.4, 1.8 Hz, H-14a), 3.55 (1H, dd, *J*= 10.8, 1.8 Hz, H-15a), 3.44 (1H, d, *J*= 10.8 Hz, H-15b), 3.41 (1H, d, *J*= 11.4 Hz, H-14b), 2.55 (1H, dd, *J*= 18.0, 3.6 Hz, H-11a), 2.50 (1H, ddd, *J*= 18.6, 10.8, 1.8 Hz, H-11b), 2.23 (1H, dd, *J*= 10.8, 3.6 Hz, H-1), 2.40 (1H, s, H-7), 1.52 (1H, dt, *J*= 13.8, 4.2 Hz, H-3a), 1.47 (3H, br s, H-12), 1.33 (2H,m, H-4), 1.25 (1H, ddd, *J*= 13.8, 11.4, 3.6 Hz, H-3b), 1.19 (1H,m, H-5a), 0.97 (1H, ddd, *J*= 13.8, 11.4, 1.2 Hz, H-5b), 0.90 (3H, s, H-13). ^{13}C{^{1}H} NMR (151 MHz, $CDCl_3$) δ (ppm) 205.0 (CH, C-10), 143.8 (C, C-9), 125.8 (CH, C-8), 71.2 (CH_2, C-14), 69.2 (CH_2, C-15), 50.7 (C, C-2), 48.1 (CH, C-7), 47.1 (CH_2, C-11), 44.0 (C, C-6), 42.5 (CH_2, C-3), 42.1 (CH, C-1), 31.3 (CH_2, C-5), 21.3 (CH_3, C-13), 20.4 (CH_2, C-4), 12.5 (CH_3, C-12). HRESIMS *m/z* 253.1795 $[M + H]^+$ calcd for $[C_{15}H_{24}O_3 + H]^+$ 253.1804.

References

1. European Comission. Pacto Verde: Propuestas Pioneras para Restaurar la Naturaleza en Europa de Aquí a 2050 y Reducir a la Mitad el uso de Plaguicidas de Aquí a 2030. Available online: https://ec.europa.eu/commission/presscorner/detail/es/ip_22_3746 (accessed on 12 July 2023).
2. European Comission. Glyphosate. Available online: https://food.ec.europa.eu/plants/pesticides/approval-active-substances/renewal-approval/glyphosate_en (accessed on 27 January 2024).
3. Roman, L.U.; Hernandez, J.D.; Del Rio, R.E.; Bucio, M.A.; Cerda-Garcia-Rojas, C.M.; Joseph-Nathan, P. Wagner-Meerwein Rearrangements of Longipinane Derivatives. *J. Org. Chem.* **1991**, *56*, 1938. https://doi.org/10.1021/jo00005a050.
4. Borgo, J.; Laurella, L.C.; Martini, F.; Catalán, C.A.N.; Sülsen, V.P. *Stevia* Genus: Phytochemistry and Biological Activities Update *Molecules* **2021**, *26*, 2733. https://doi.org/10.3390/molecules26092733.
5. Román, L.U.; Zepeda, L.G.; Morales, N.R.; Hernández, J.D.; Cerda-García-Rojas, C.M.; Joseph-Nathan, P. Molecular Rearrangement of Rastevione Mesylate into Arteagane Derivatives. *J. Nat. Prod.* **1995**, *58*, 1808. https://doi.org/10.1021/np50126a002.
6. Román, L.U.; Zepeda, L.G.; Morales, N.R.; Flores, S.; Hernández, J.D.; Cerda-García-Rojas, C.M.; Joseph-Nathan, P. Mechanistic Studies of the Longipinane to Arteagane Rearrangement. *J. Nat. Prod.* **1996**, *59*, 391. https://doi.org/10.1021/np960072x.
7. Cerda-García-Rojas, C.M.; Flores-Sandoval, C.A.; Román, L.U.; Hernández, J.D.; Joseph-Nathan, P. A Regioselective Wagner–Meerwein Rearrangement Directed towards the Six-Membered Ring of the Longipinane Skeleton. *Tetrahedron* **2002**, *58*, 1061. https://doi.org/10.1016/S0040-4020(01)01205-4.
8. Román, L.U.; Cerda-García-Rojas, C.M.; Guzmán, R.; Armenta, C.; Hernández, J.D.; Joseph-Nathan, P. Jiquilpane Hydrocarbon Skeleton Generated by Two Successive Wagner–Meerwein Rearrangements of Longipinane Derivatives. *J. Nat. Prod.* **2002**, *65*, 1540. https://doi.org/10.1021/np0201570.
9. Chacón-Morales, P.A.; Amaro-Luis, J.M. Meridane and Uladane, Two Unprecedented Sesquiterpene Skeletons Obtained by Wagner–Meerwein Rearrangements of Longipinane Derivatives. *Tetrahedron Lett.* **2016**, *57*, 2713. https://doi.org/10.1016/j.tetlet.2016.04.116.
10. Reddy, D.S.; Kutateladze, A.G. Computational Structure Revision of a Longipinane Derivative Meridane. *Tetrahedron Lett.* **2016**, *57*, 4727. https://doi.org/10.1016/j.tetlet.2016.09.030.
11. Román, L.U.; Rebeca Morales, N.; Hernández, J.D.; Cerda-García-Rojas, C.M.; Gerardo Zepeda, L.; Flores-Sandoval, C.A.; Joseph-Nathan, P. Generation of the New Quirogane Skeleton by a Vinylogous retro-Michael Type Rearrangement of Longipinene Derivatives. *Tetrahedron* **2001**, *57*, 7269. https://doi.org/10.1016/S0040-4020(01)00718-9.
12. Joseph-Nathan, P.; Meléndez-Rodríguez, M.; Cerda-García-Rojas, C.M.; Catalan, C.A.N. Photochemical Rearrangements of Highly Functionalized Longipinene Derivatives. *Tetrahedron Lett.* **1996**, *37*, 8093. https://doi.org/10.1016/0040-4039(96)01848-5.
13. Meléndez-Rodríguez, M.; Cerda-García-Rojas, C.M.; Joseph-Nathan, P. Quirogane, Prenopsane, and Patzcuarane Skeletons Obtained by Photochemically Induced Molecular Rearrangements of Longipinene Derivatives. *J. Nat. Prod.* **2002**, *65*, 1398. https://doi.org/10.1021/np020158s.

14. Román, L.U.; Hernández, J.D.; Cerda-García-Rojas, C.M.; Domínguez-López, R.M.; Joseph-Nathan, P. Molecular Rearrangements in the Longipinene Series. *J. Nat. Prod.* **1992**, *55*, 577. https://doi.org/10.1021/np50083a004.
15. Joseph-Nathan, P.; Cerda-Garcia-Rojas, C.M. Molecular Rearrangements in Longipinane Derivatives. *Pure & Appl. Chem.* **1994**, *66*, 2361. https://doi.org/10.1351/pac199466102361.
16. Chacón-Morales, P.A.; Amaro-Luis, J.M.; Kutateladze, A.G. Structure Determination and Mechanism of Formation of a *seco*-Moreliane Derivative Supported by Computational Analysis. *J. Nat. Prod.* **2017**, *80*, 1214. https://doi.org/10.1021/acs.jnatprod.7b00041.
17. Armenta-Salinas, C.; Guzmán-Mejía, R.; García-Gutiérrez, H.A.; Román-Marín, L.U.; Hernández-Hernández, J.D.; Cerda-García-Rojas, C.M.; Joseph-Nathan, P. Novel Sesquiterpene Skeletons by Multiple Wagner–Meerwein Rearrangements of a Longipinane-1,9-diol Derivative. *J. Nat. Prod.* **2019**, *82*, 3410. https://doi.org/10.1021/acs.jnatprod.9b00784.
18. Ruiz-Ferrer, C.; Román-Marín, L.U.; Hernández-Hernández, J.D.; Cerda-García-Rojas, C.M.; Joseph-Nathan, P. Novel Sesquiterpenoid Skeletons by Wagner–Meerwein Rearrangements of Longipinane-9,13-diol-1-one Derivatives. *J. Nat. Prod.* **2021**, *84*, 1087. https://doi.org/10.1021/acs.jnatprod.0c01160.
19. Meléndez-Rodríguez, M.; Cerda-García-Rojas, C.M.; Catalán, C.A.N.; Joseph-Nathan, P. Mechanistic Studies of the Photochemical Rearrangement of 1-Oxolongipin-2-ene Derivatives. *Tetrahedron* **2002**, *58*, 2331. https://doi.org/10.1016/S0040-4020(02)00121-7.
20. Barrero, A.F.; Herrador, M.M.; Molina, J.M.; Quílez, J.F.; Quirós, M. α-Longipinene Derivatives from *Santolina viscosa*. A Conformational Analysis of the Cycloheptane Ring. *J. Nat. Prod.* **1994**, *57*, 873. https://doi.org/10.1021/np50109a001.
21. Barrero, A.F.; Herrador, M.M.; Álvarez-Manzaneda, R.J.; Quirós, M.; Lara, A.; Quílez del Moral, J. Longipinene Derivatives from *Santolina viscosa*. *J. Nat. Prod.* **2000**, *63*, 587. https://doi.org/10.1021/np9904206.
22. Shastri, M.H.; Dev, S. Studies in Sesquiterpenes-LX[a,b] Reversion of Longipinane to Himachalane System: Revision of Structure of Isocentdarol. *Tetrahedron* **1992**, *48*, 4905.
23. Dolomanov, O. V.; Bourhis, L. J.; Gildea, R. J.; Howard, J. A. K.; Puschmann, H. OLEX2: A Complete Structure Solution, Refinement and Analysis Program. *J. Appl. Crystallogr.* **2009**, *42*, 339. https://doi.org/10.1107/S0021889808042726.
24. Sheldrick, G. M. SHELXT– Integrated Space-Group and Crystal-Structure Determination. *Acta Crystallogr. Sect A Foundations and Advances* **2015**, *71*, 3. https://doi.org/10.1107/S2053273314026370.
25. Sheldrick, G. M. Crystal Structure Refinement with SHELXL. *Acta Crystallogr. Sect C Structural Chemistry* **2015**, *71*, 3. https://doi.org/10.1107/S2053229614024218.
26. Hehre, W.; Klunzinger, P.; Deppmeier, B.; Driessen, A.; Uchida, N.; Hashimoto, M.; Fukushi, E.; Takata, Y., Efficient Protocol for Accurately Calculating ^{13}C Chemical Shifts of Conformationally Flexible Natural Products: Scope, Assessment, and Limitations. *J. Nat. Prod.* **2019,** *82*, 2299. https://doi.org/10.1021/acs.jnatprod.9b00603.
27. Navarro-Rocha, J.; Andrés, M. F.; Díaz, C. E.; Burillo, J.; González-Coloma, A. Composition and Biocidal Properties of Essential Oil from pre-Domesticated Spanish *Satureja Montana*. *Industrial Crops and Products* **2020**, *145*, 111958. https://doi.org/https://doi.org/10.1016/j.indcrop.2019.111958.
28. Rueden, C. T.; Schindelin, J.; Hiner, M. C.; DeZonia, B. E.; Walter, A. E.; Arena, E. T.; Eliceiri, K. W. ImageJ2: ImageJ for the Next Generation of Scientific Image Data. *BMC Bioinformatics* **2017**, *18*, 529. https://doi.org/10.1186/s12859-017-1934-z.
29. Santana, O.; Andrés, M. F.; Sanz, J.; Errahmani, N.; Abdeslam, L.; González-Coloma, A. Valorization of Essential Oils from Moroccan Aromatic Plants. *Nat. Prod. Commun.* **2014**, *9*, 1109. https://doi.org/10.1177/1934578X1400900812.
30. González, J.; Valcárcel, F.; Aguilar, A.; Olmeda, A. S. In vitro Feeding of *Hyalomma lusitanicum* Ticks on Artificial Membranes. *Exp. Appl. Acarol.* **2017**, *72*, 449. https://doi.org/10.1007/s10493-017-0167-1.
31. Andrés, M. F.; González-Coloma, A.; Muñoz, R.; De la Peña, F.; Julio, L. F.; Burillo, J. Nematicidal Potential of Hydrolates from the semi-Industrial Vapor-Pressure Extraction of Spanish Aromatic Plants. *Environ. Sci. Pollut. Res.* **2018**, *25*, 29834. https://doi.org/10.1007/s11356-017-9429-z.
32. Püntener, W. Manual for Field Trials in Plant Protection. *Ciba-Geigy Limited: Basel, Switzerland* **1981**, 205.

NMR AND IR SPECTRA

^{1}H NMR, DEPT 135, ^{13}C NMR, IR, HSQC, HMBC, COSY, TOCSY and NOESY of **9**

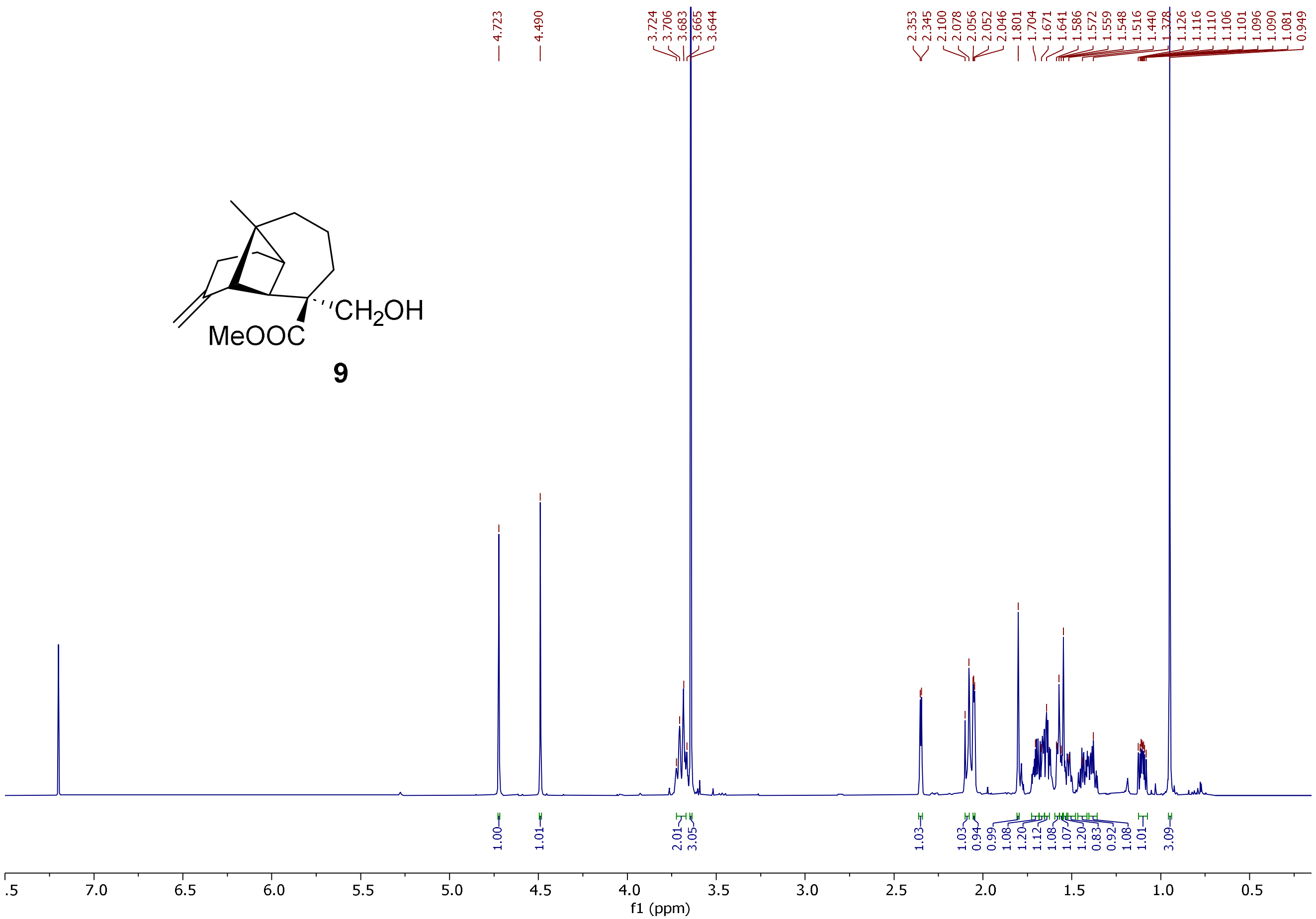

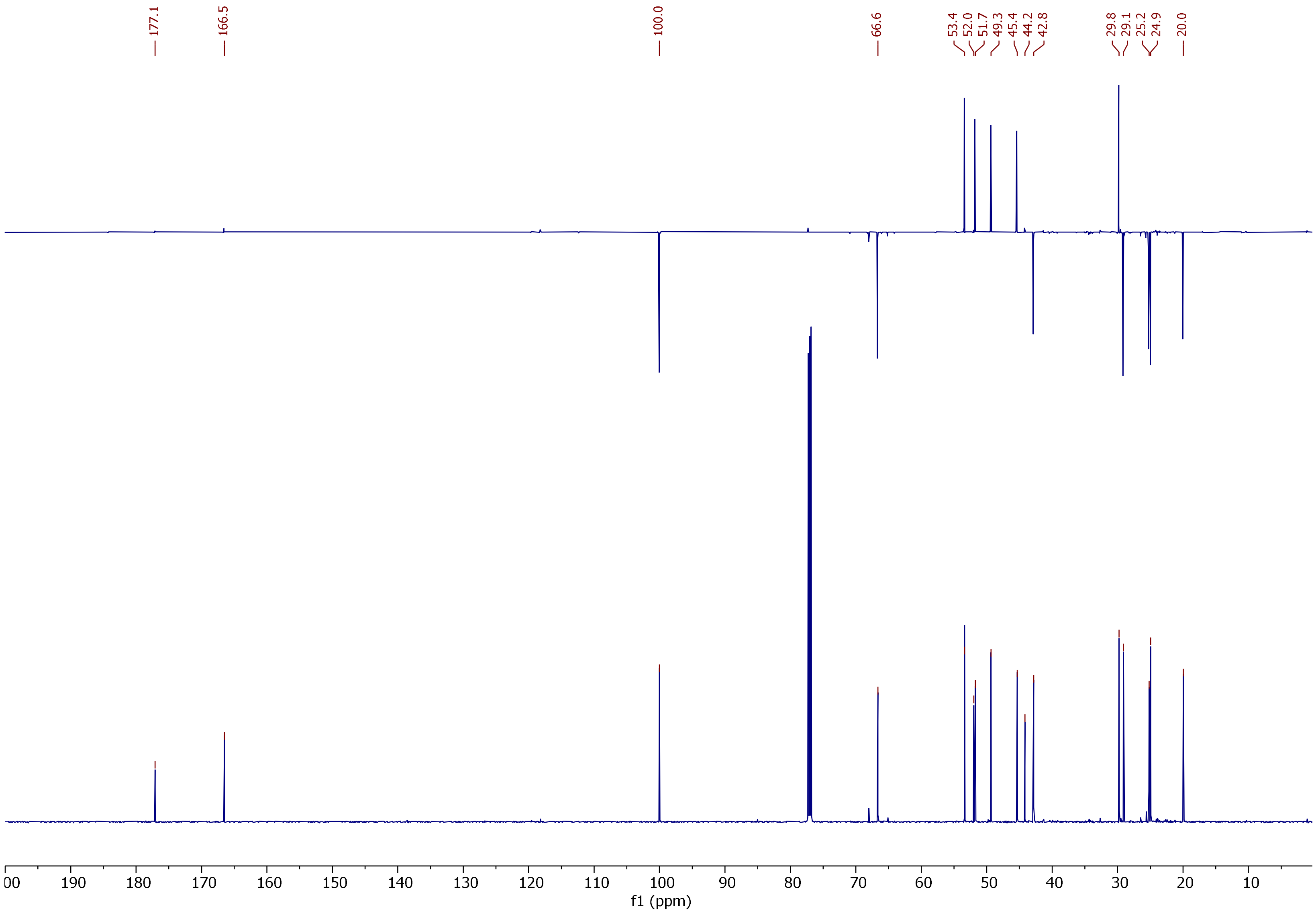
177.1
166.5
100.0
66.6
53.4
52.0
51.7
49.3
45.4
44.2
42.8
29.8
29.1
25.2
24.9
20.0
00
190
180
170
160
150
140
130
120
110
100
90
80
70
60
50
40
30
20
10
f1 (ppm)

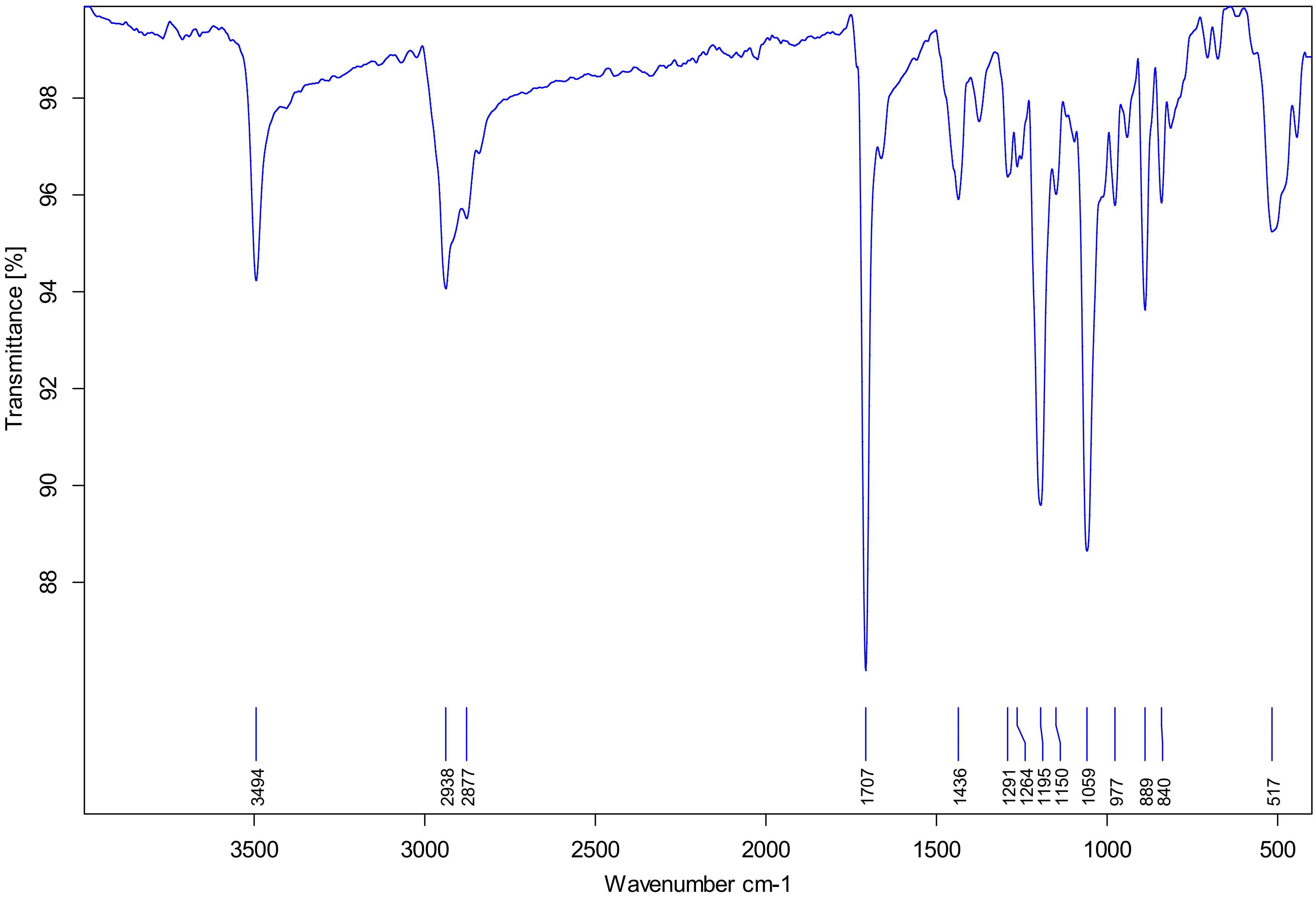

Transmittance [%]
98
96
94
92
90
88
3494
2938
2877
1707
1436
1291
1264
1195
1150
1059
977
889
840
517
3500
3000
2500
2000
1500
1000
500
Wavenumber cm-1

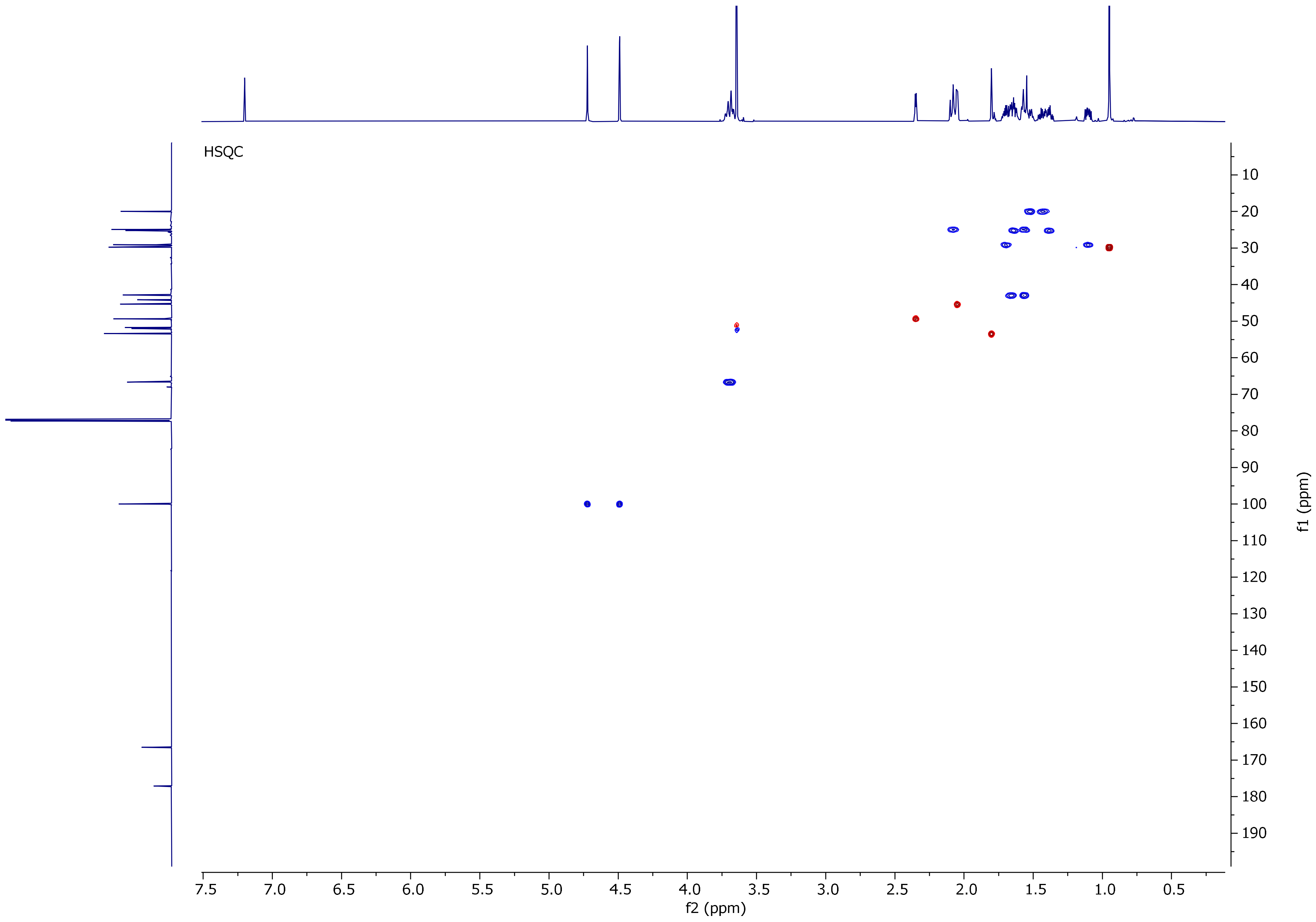
HSQC
7.5
7.0
6.5
6.0
5.5
5.0
4.5
4.0
3.5
3.0
2.5
2.0
1.5
1.0
0.5
f2 (ppm)
10
20
30
40
50
60
70
80
90
100
110
120
130
140
150
160
170
180
190
f1 (ppm)

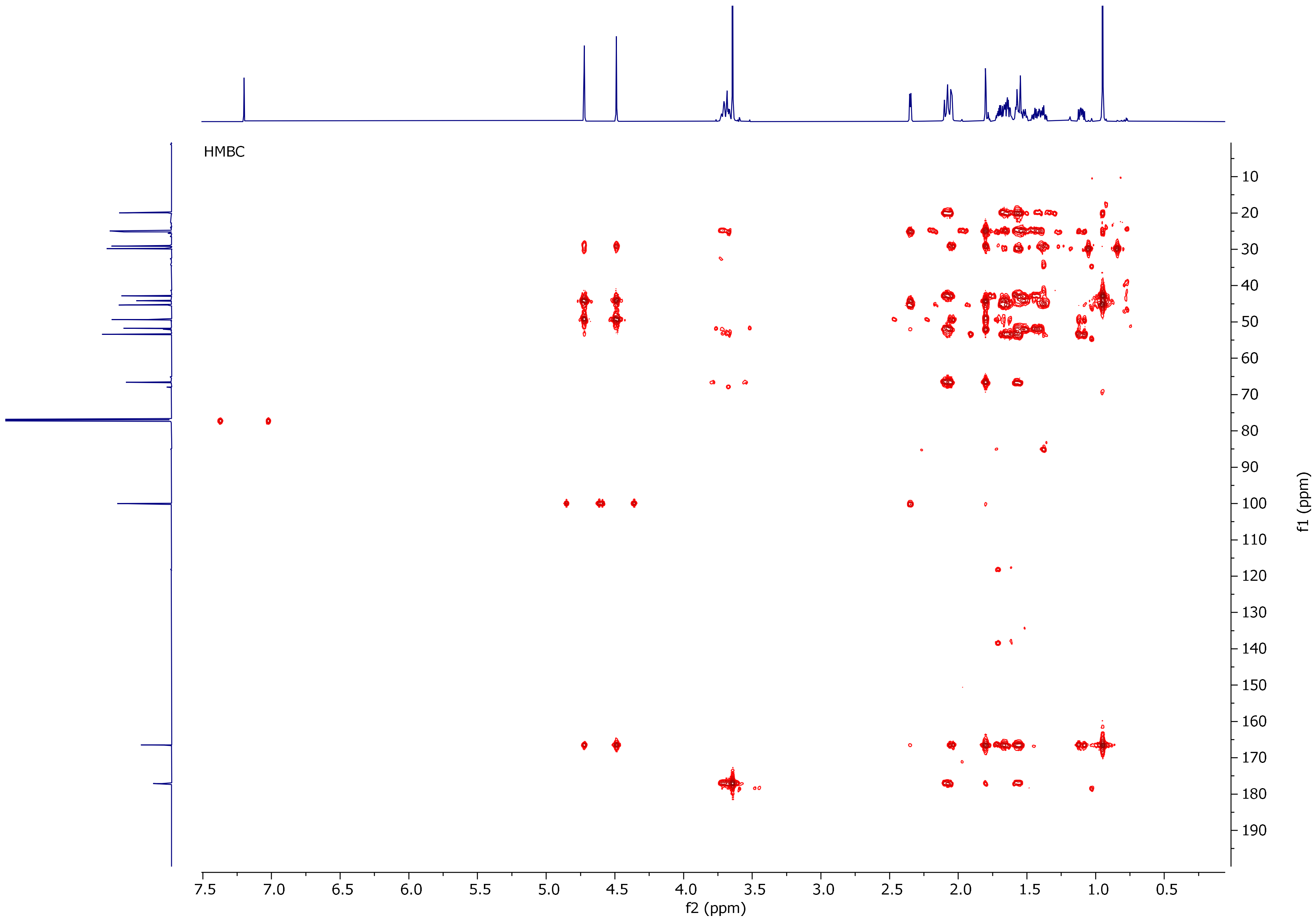
HMBC
f2 (ppm)
f1 (ppm)
7.5
7.0
6.5
6.0
5.5
5.0
4.5
4.0
3.5
3.0
2.5
2.0
1.5
1.0
0.5
10
20
30
40
50
60
70
80
90
100
110
120
130
140
150
160
170
180
190

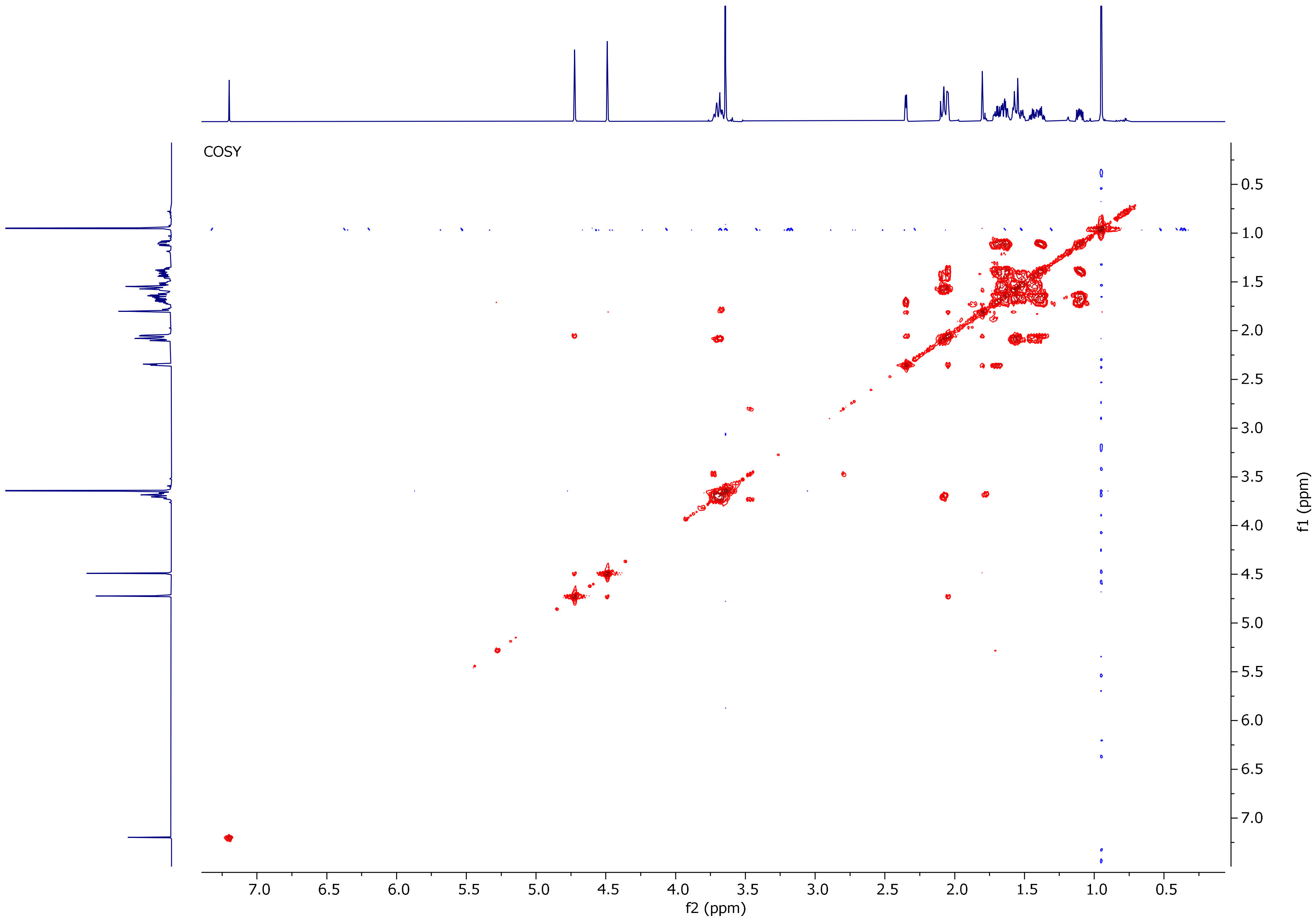

COSY
f2 (ppm)
f1 (ppm)

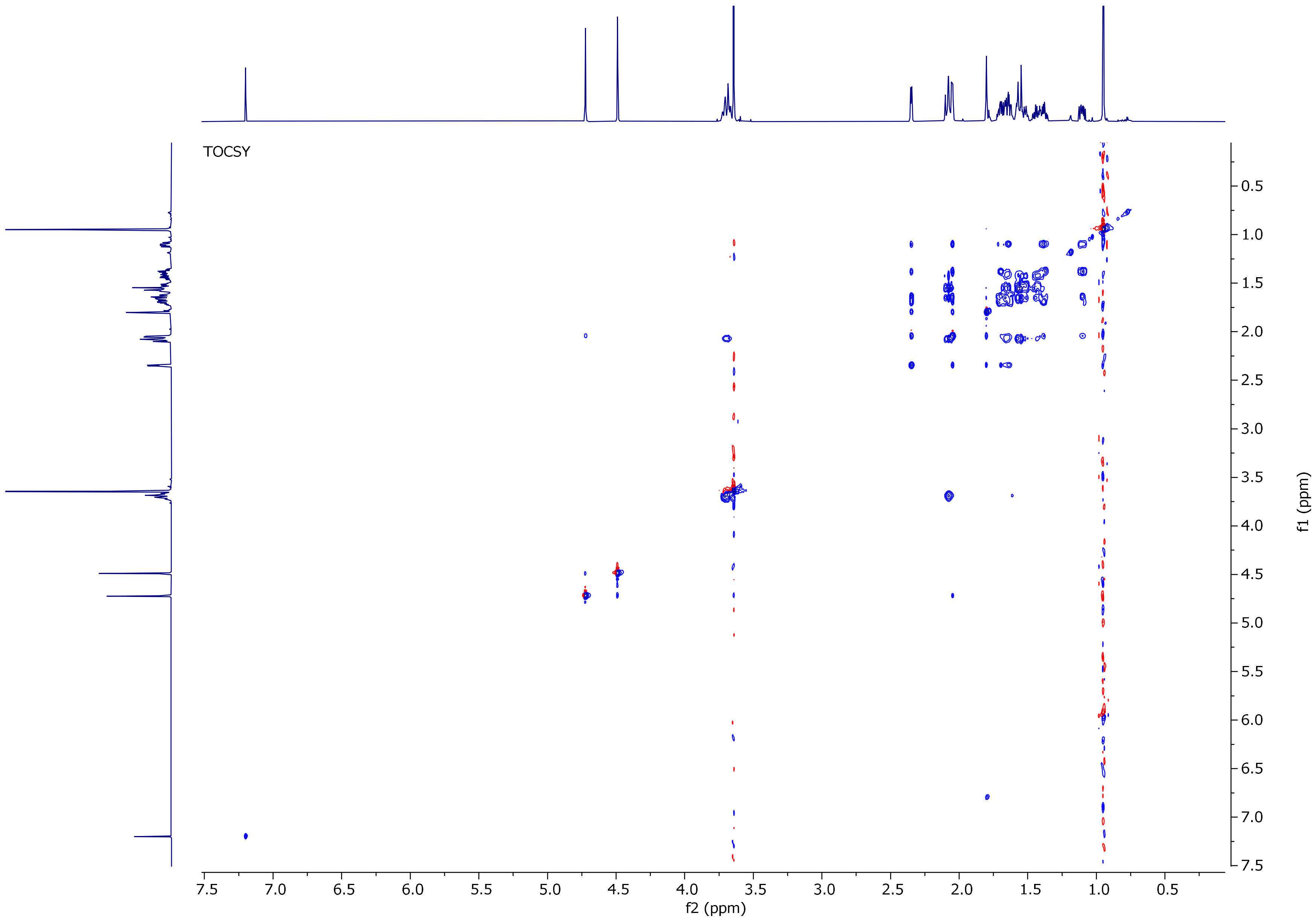
TOCSY
f2 (ppm)
f1 (ppm)
7.5
7.0
6.5
6.0
5.5
5.0
4.5
4.0
3.5
3.0
2.5
2.0
1.5
1.0
0.5

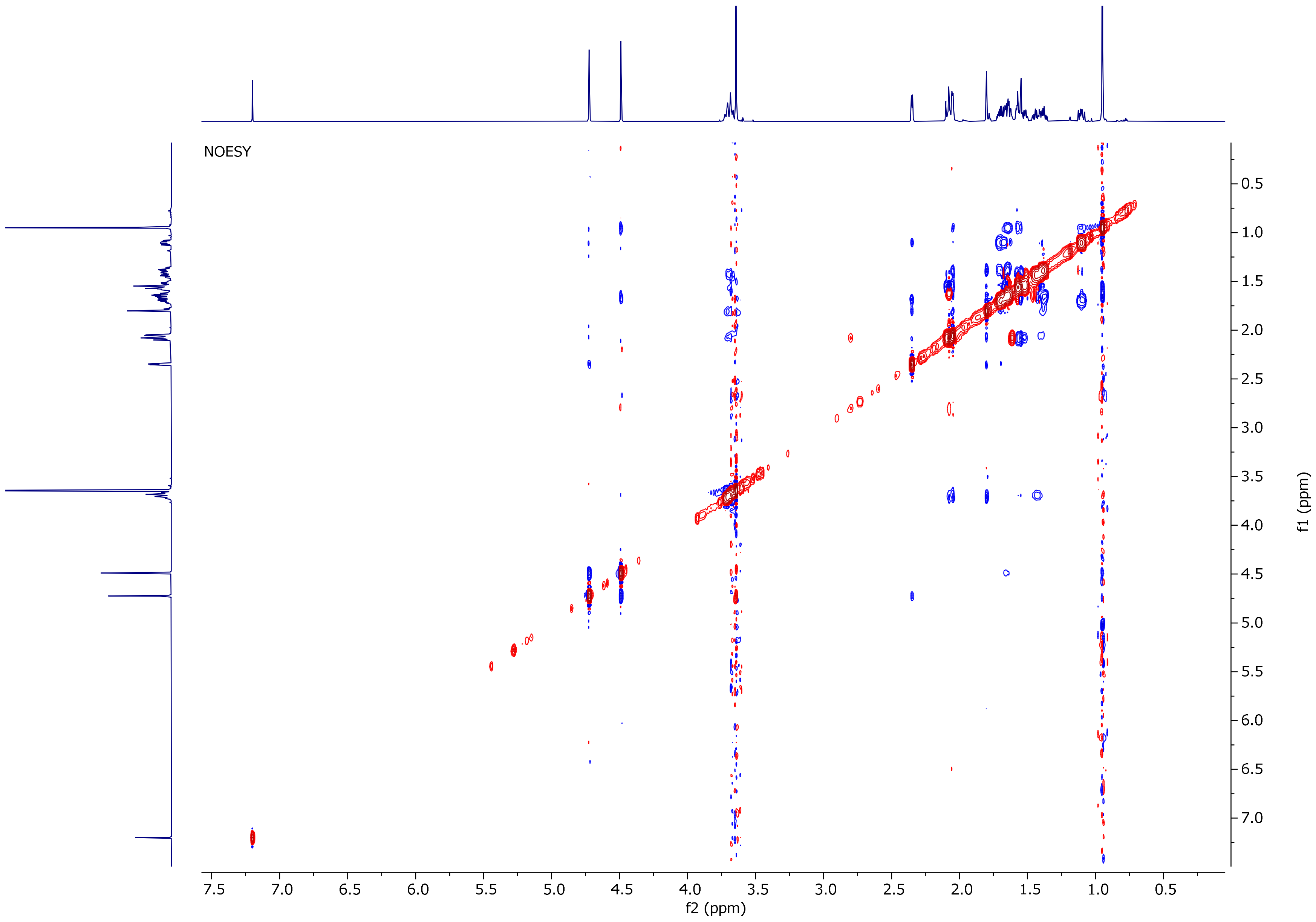
NOESY
f2 (ppm)
f1 (ppm)
7.5
7.0
6.5
6.0
5.5
5.0
4.5
4.0
3.5
3.0
2.5
2.0
1.5
1.0
0.5

^{1}H NMR, DEPT 135, ^{13}C NMR, IR, HSQC, HMBC, COSY, TOCSY and NOESY of **10**

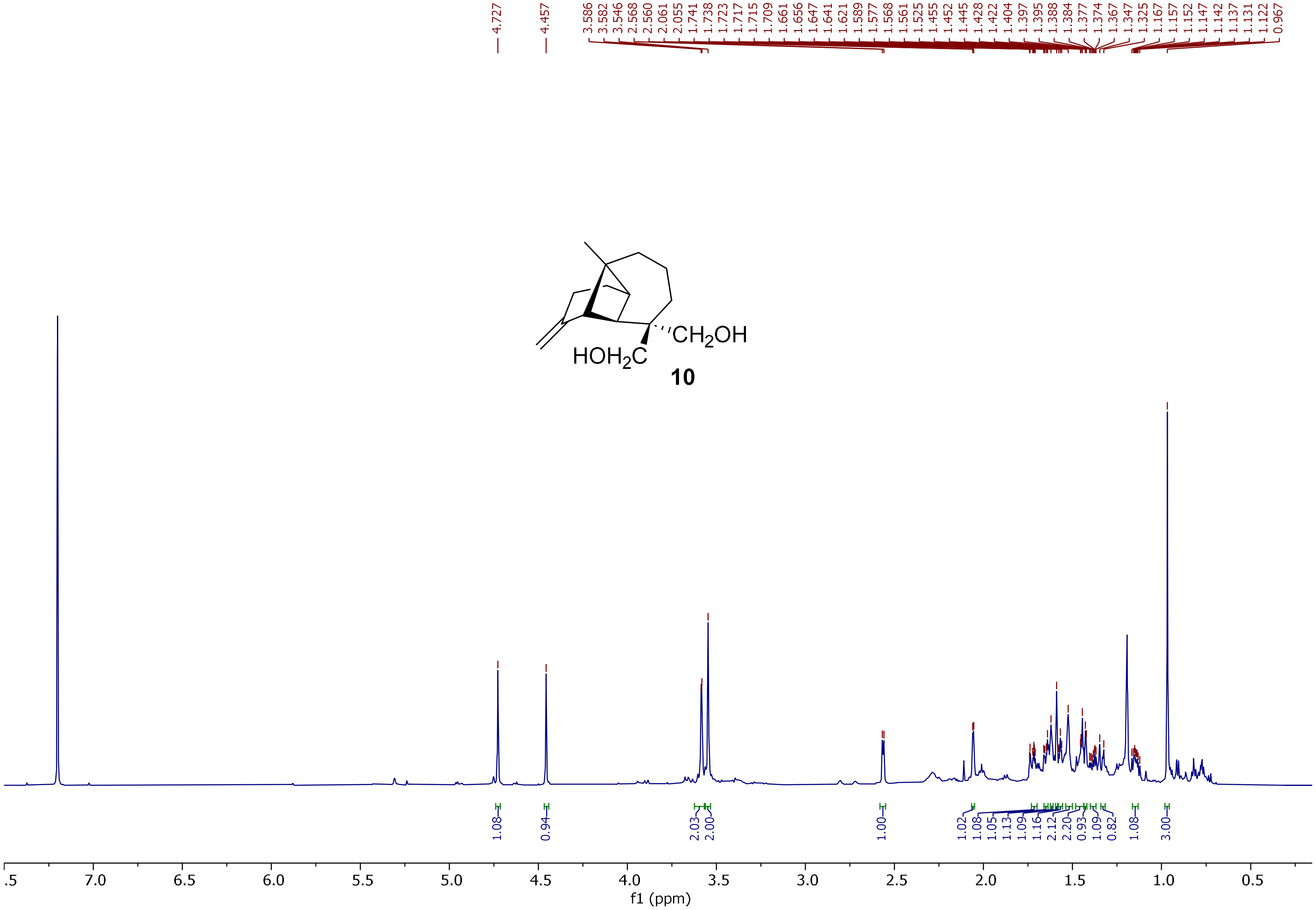

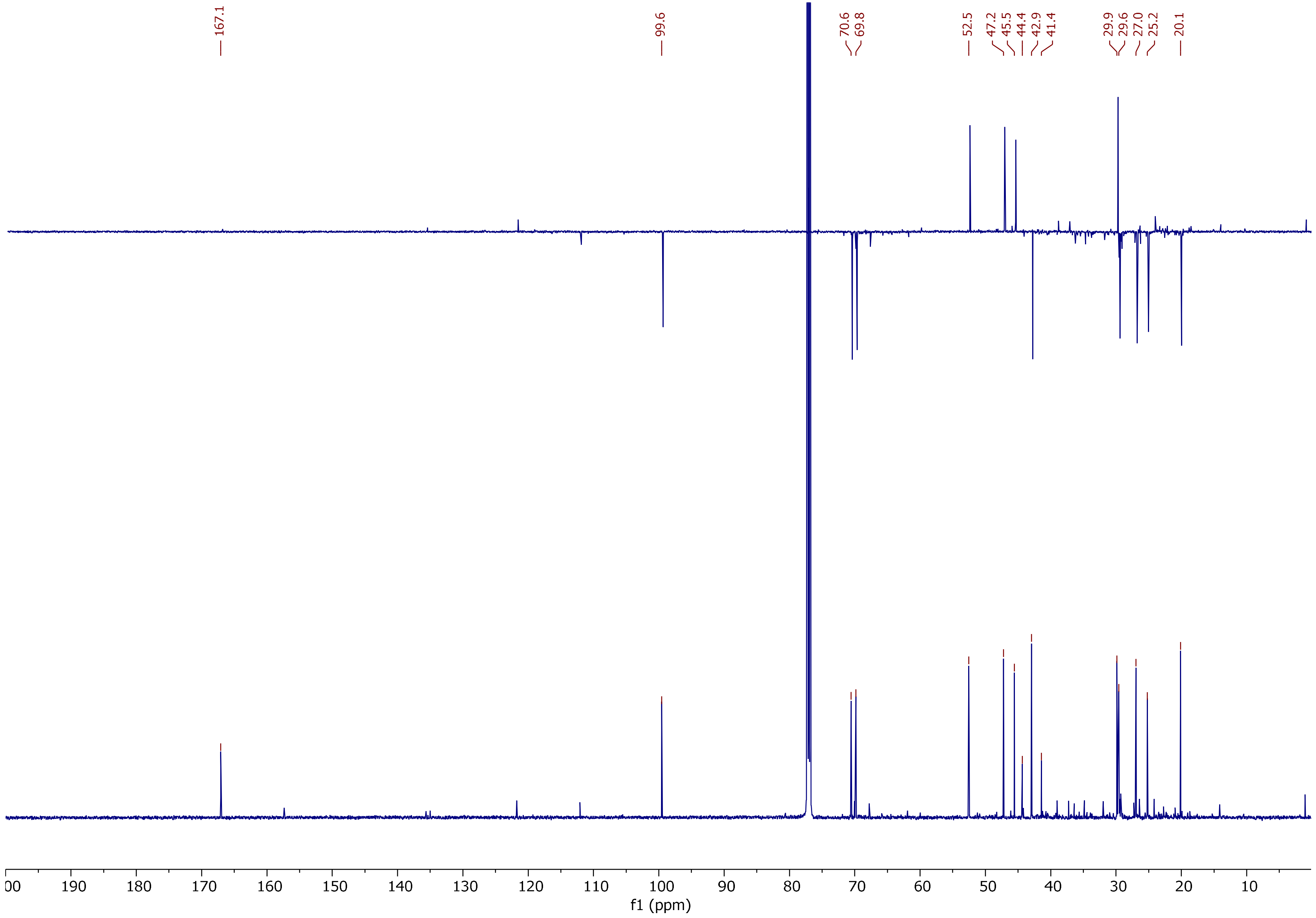

167.1
99.6
70.6
69.8
52.5
47.2
45.5
44.4
42.9
41.4
29.9
29.6
27.0
25.2
20.1
00
190
180
170
160
150
140
130
120
110
100
90
80
70
60
50
40
30
20
10
f1 (ppm)

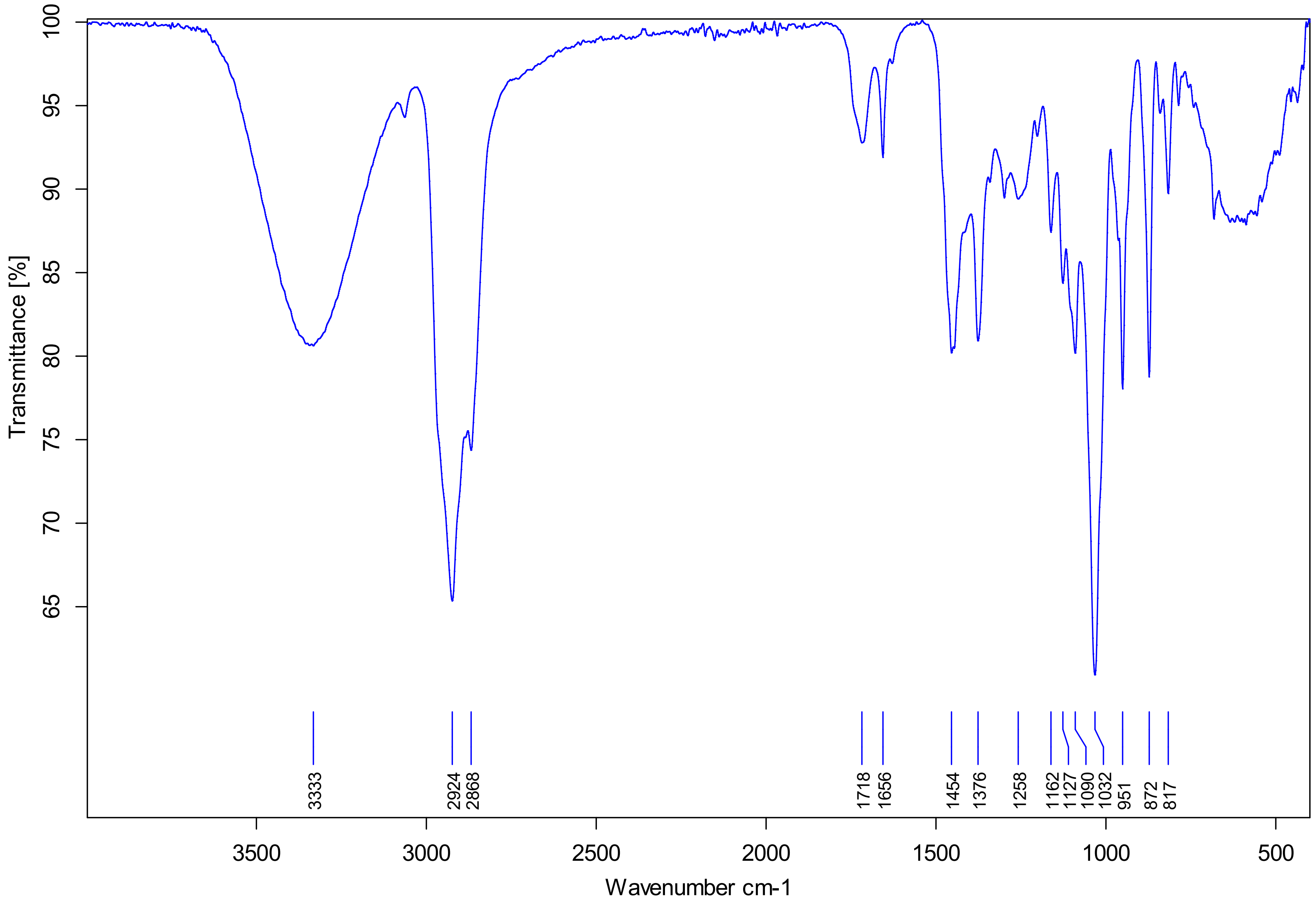

Transmittance [%]
100
95
90
85
80
75
70
65
3333
2924
2868
1718
1656
1454
1376
1258
1162
1127
1090
1032
951
872
817
3500
3000
2500
2000
1500
1000
500
Wavenumber cm-1

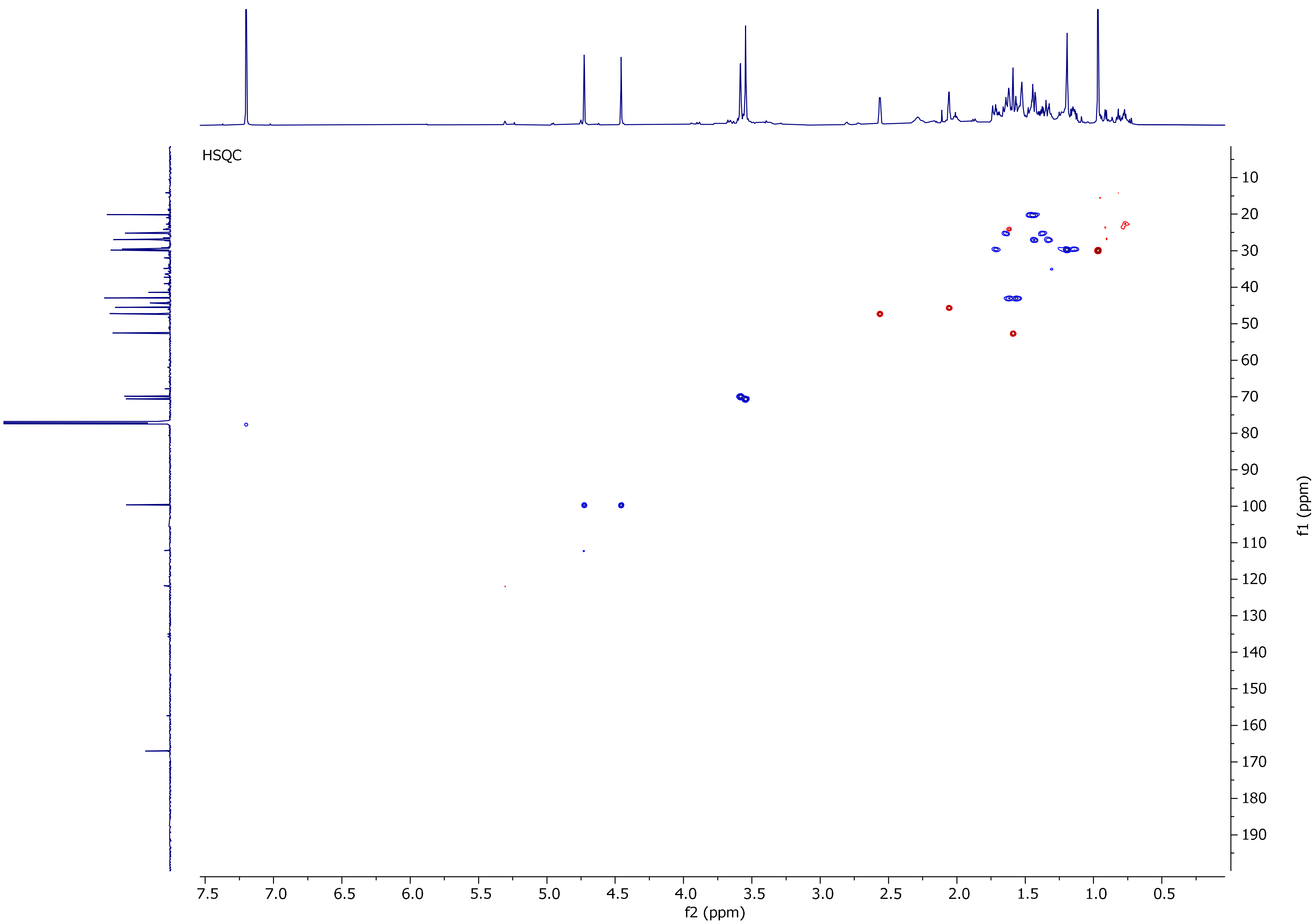
HSQC
f2 (ppm)
f1 (ppm)

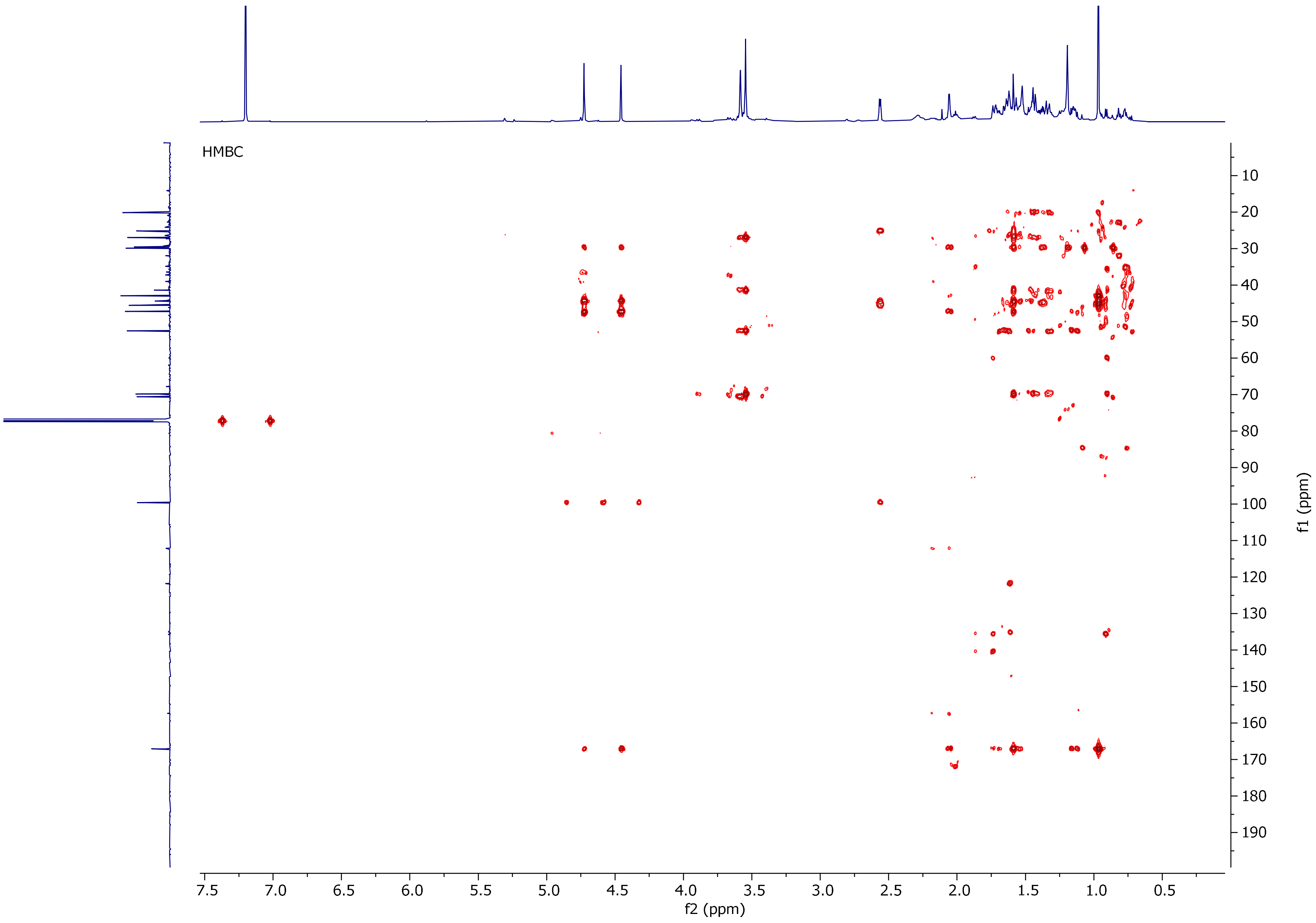
HMBC
7.5
7.0
6.5
6.0
5.5
5.0
4.5
4.0
3.5
3.0
2.5
2.0
1.5
1.0
0.5
f2 (ppm)
10
20
30
40
50
60
70
80
90
100
110
120
130
140
150
160
170
180
190
f1 (ppm)

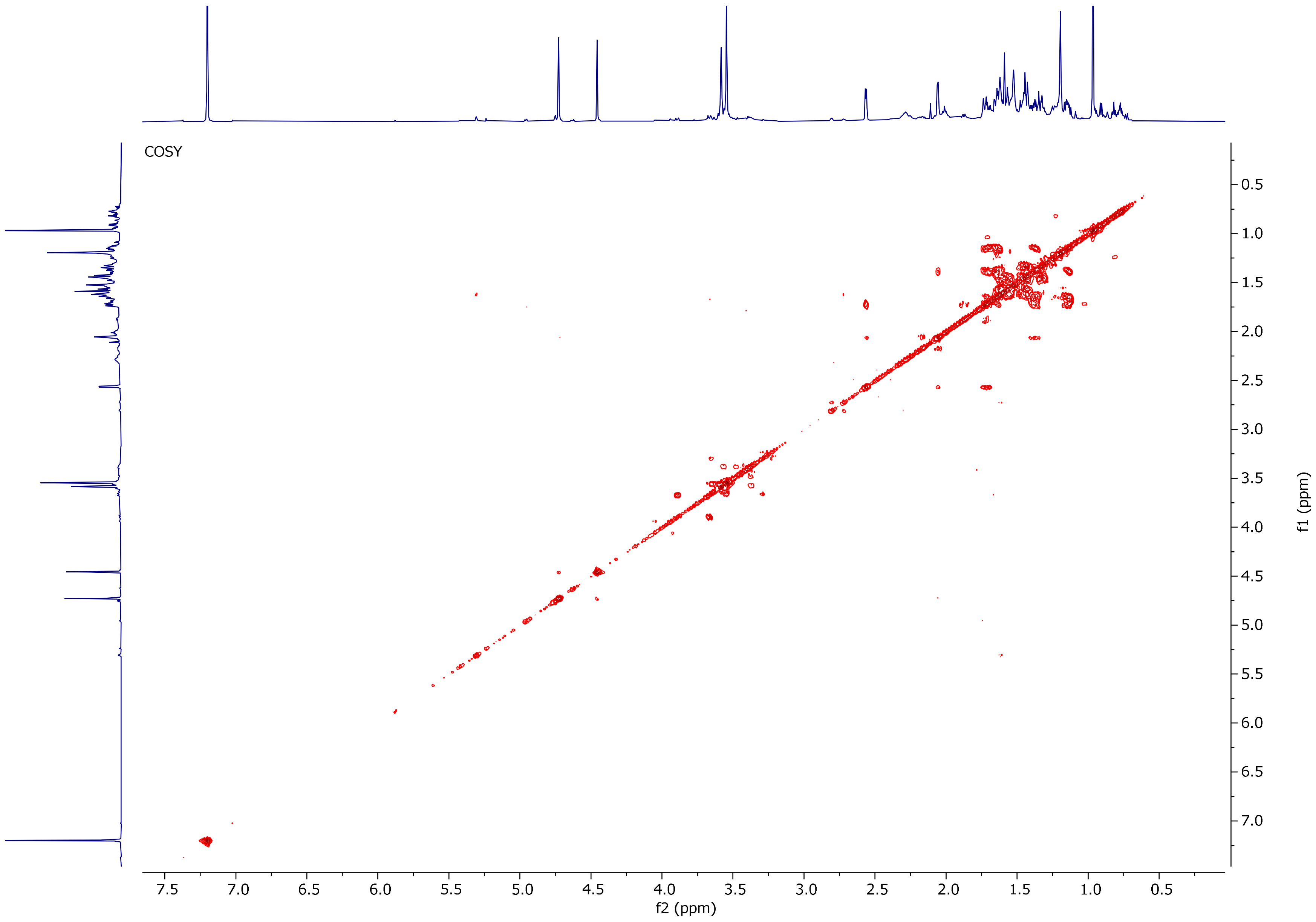
COSY
f2 (ppm)
f1 (ppm)

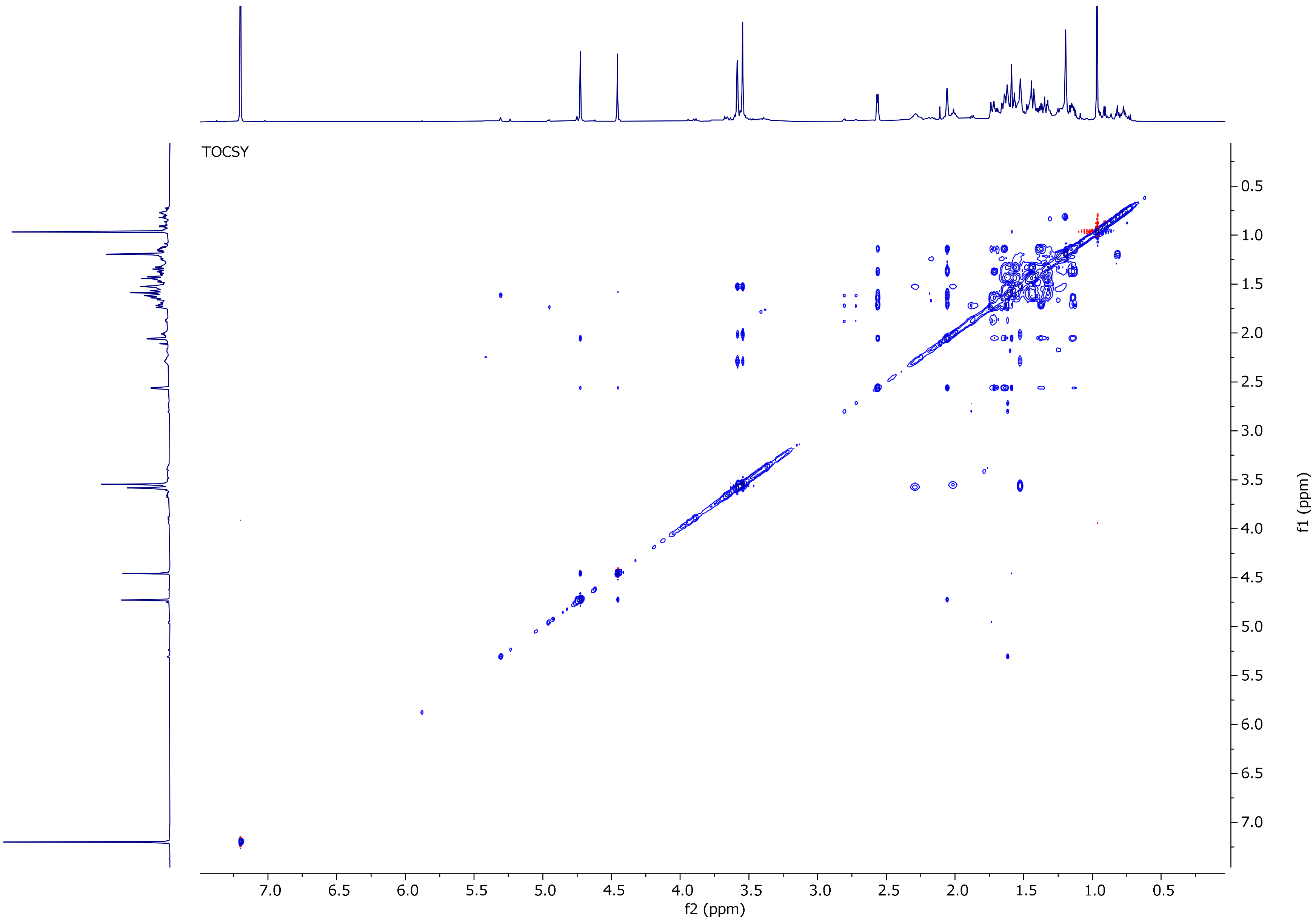
TOCSY
f2 (ppm)
f1 (ppm)

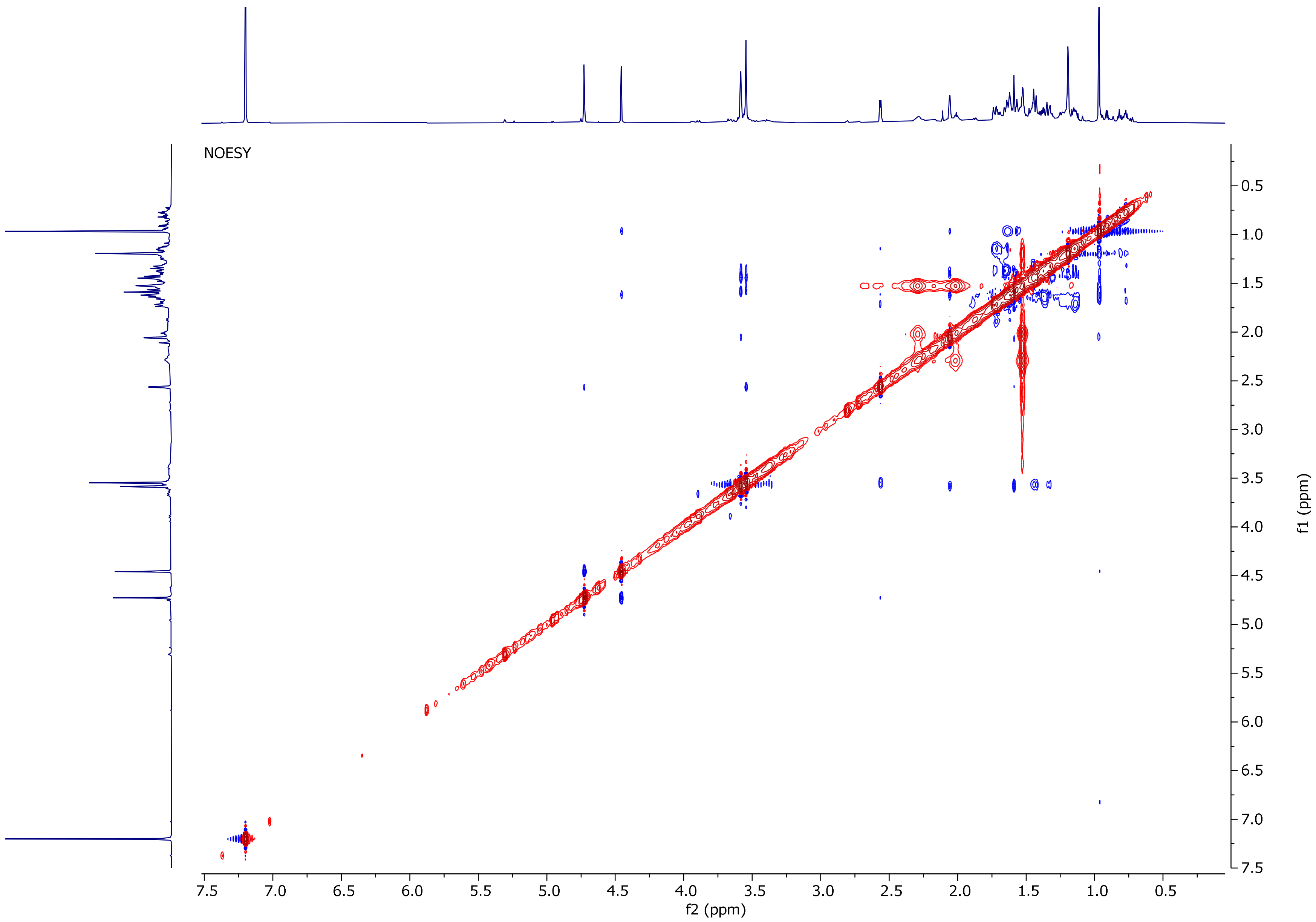
NOESY
f2 (ppm)
f1 (ppm)
7.5
7.0
6.5
6.0
5.5
5.0
4.5
4.0
3.5
3.0
2.5
2.0
1.5
1.0
0.5

^{1}H NMR, DEPT 135, ^{13}C NMR, IR, HSQC, HMBC, COSY, TOCSY and NOESY of **11**

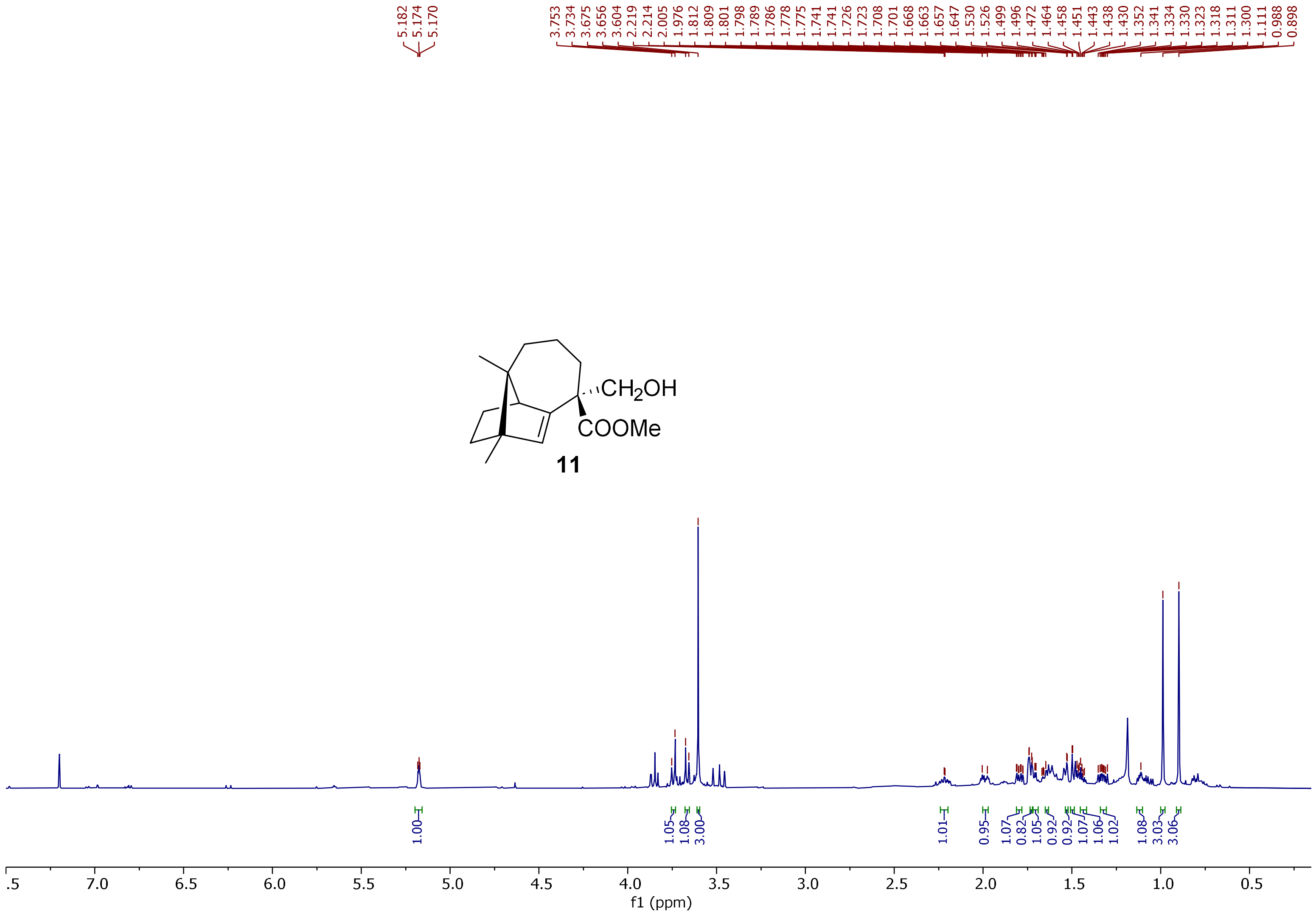

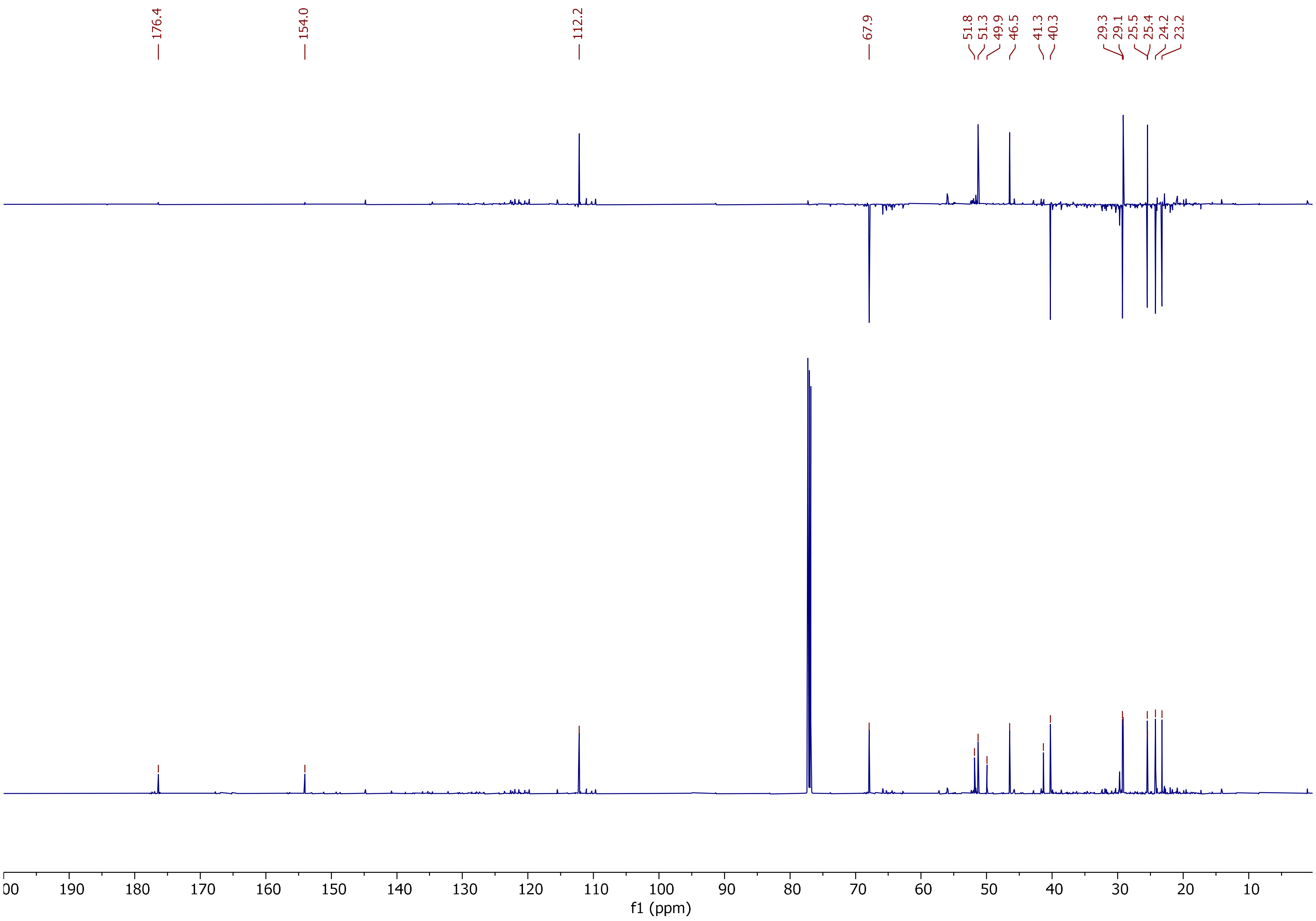
176.4
154.0
112.2
67.9
51.8
51.3
49.9
46.5
41.3
40.3
29.3
29.1
25.5
25.4
24.2
23.2
00
190
180
170
160
150
140
130
120
110
100
90
80
70
60
50
40
30
20
10
f1 (ppm)

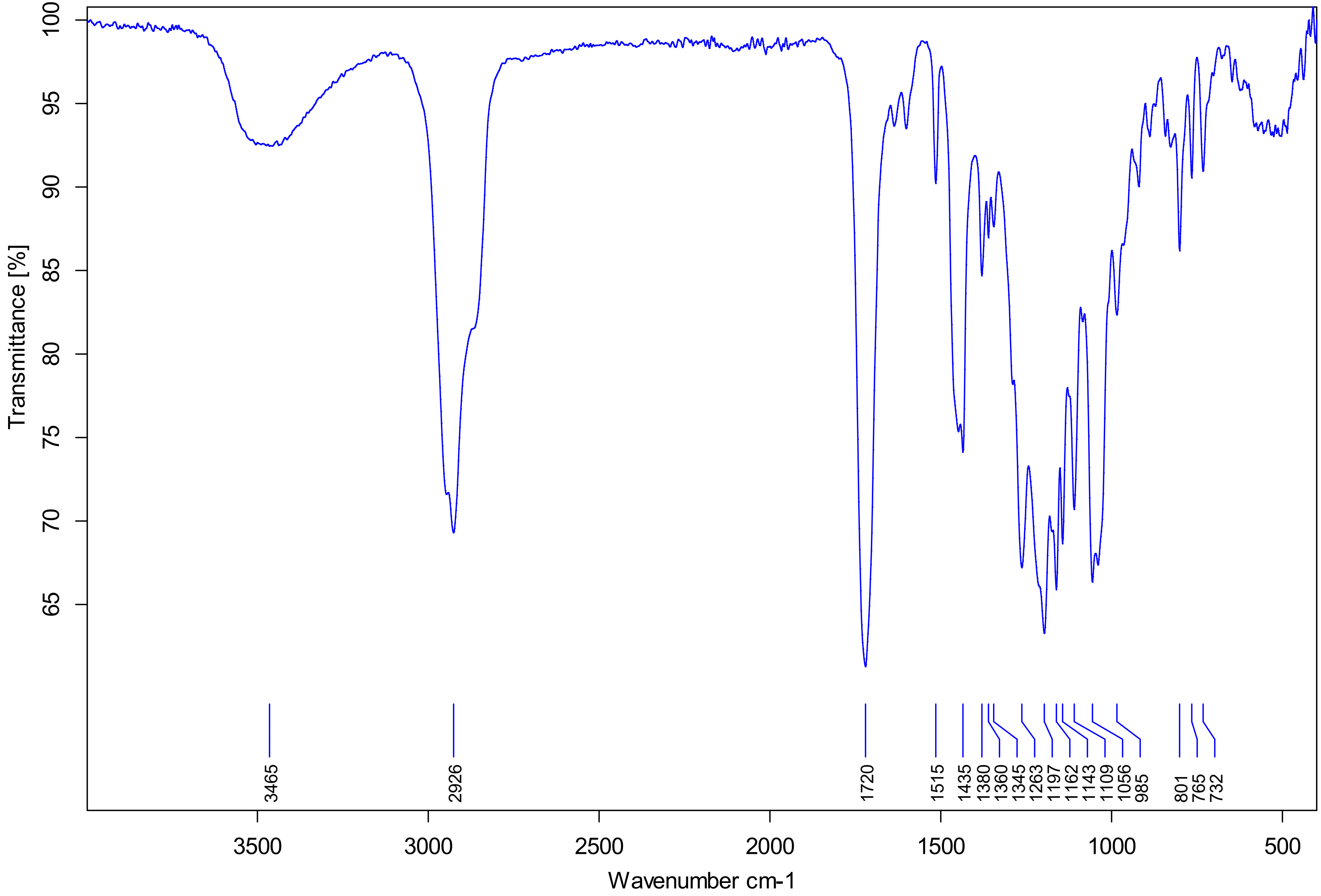

Transmittance [%]
100
95
90
85
80
75
70
65
3465
2926
1720
1515
1435
1380
1360
1345
1263
1197
1162
1143
1109
1056
985
801
765
732
3500
3000
2500
2000
1500
1000
500
Wavenumber cm-1

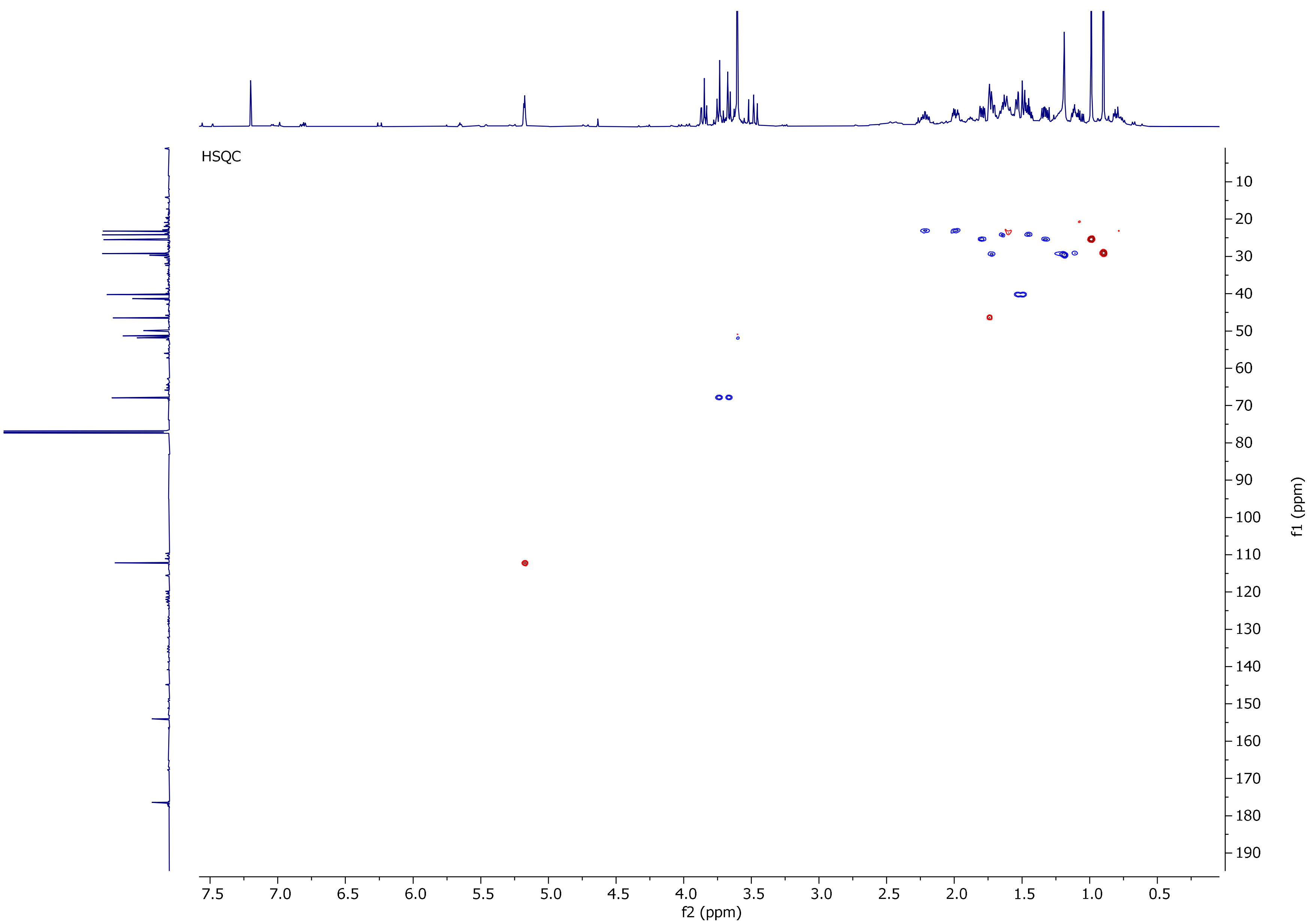
HSQC
f2 (ppm)
f1 (ppm)
7.5
7.0
6.5
6.0
5.5
5.0
4.5
4.0
3.5
3.0
2.5
2.0
1.5
1.0
0.5
10
20
30
40
50
60
70
80
90
100
110
120
130
140
150
160
170
180
190

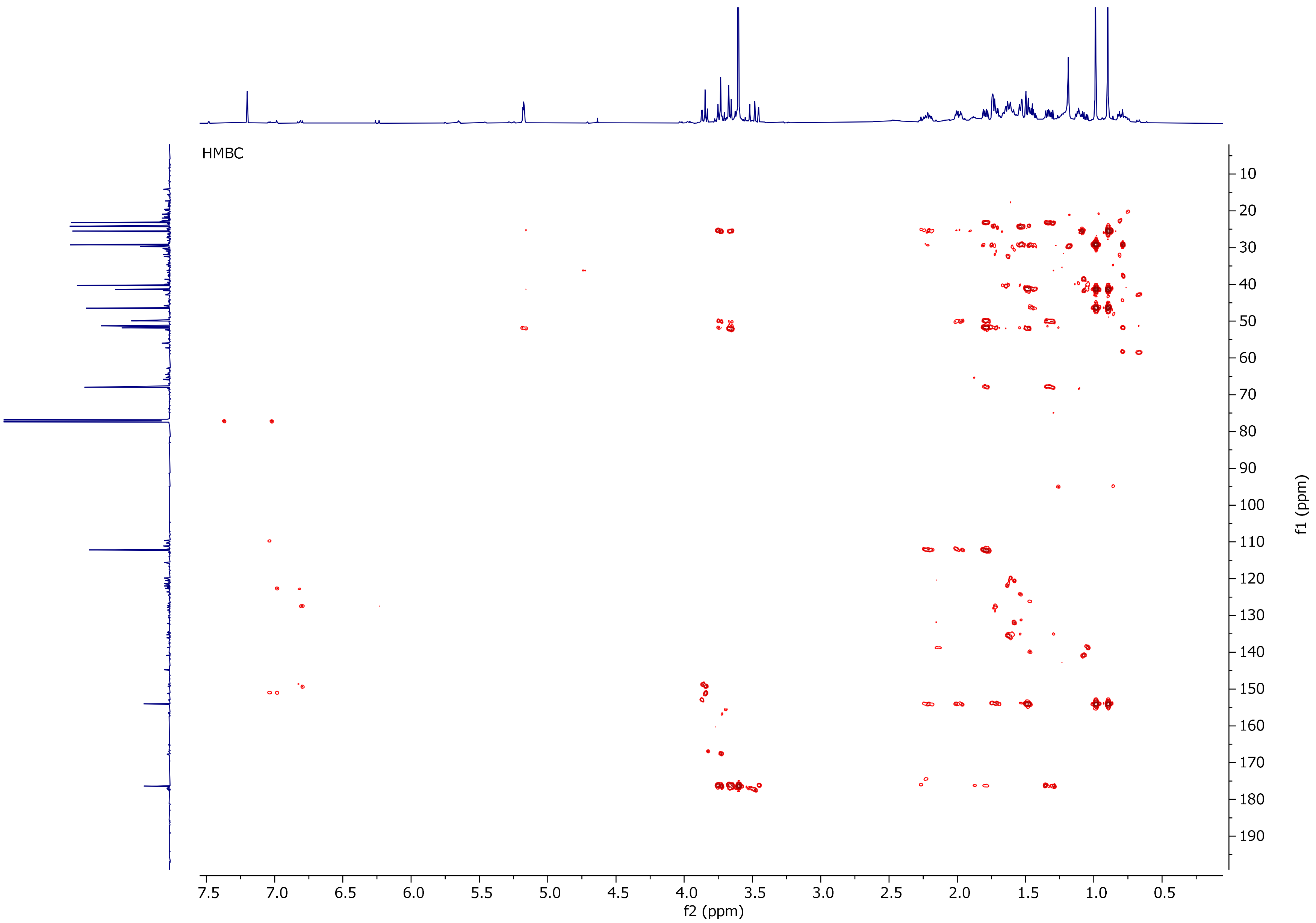
HMBC
f2 (ppm)
f1 (ppm)
7.5
7.0
6.5
6.0
5.5
5.0
4.5
4.0
3.5
3.0
2.5
2.0
1.5
1.0
0.5
10
20
30
40
50
60
70
80
90
100
110
120
130
140
150
160
170
180
190

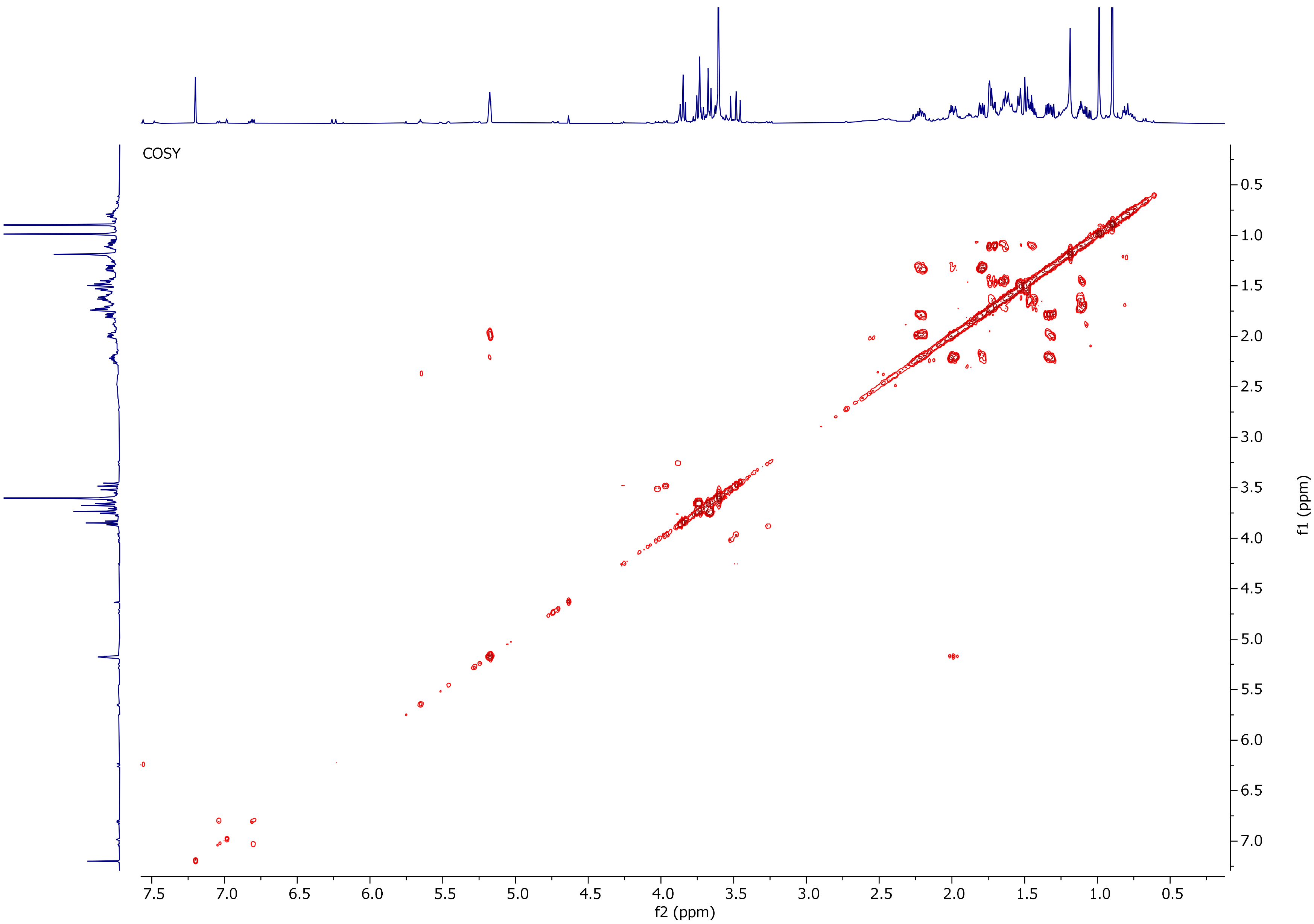
COSY
f2 (ppm)
f1 (ppm)
7.5
7.0
6.5
6.0
5.5
5.0
4.5
4.0
3.5
3.0
2.5
2.0
1.5
1.0
0.5

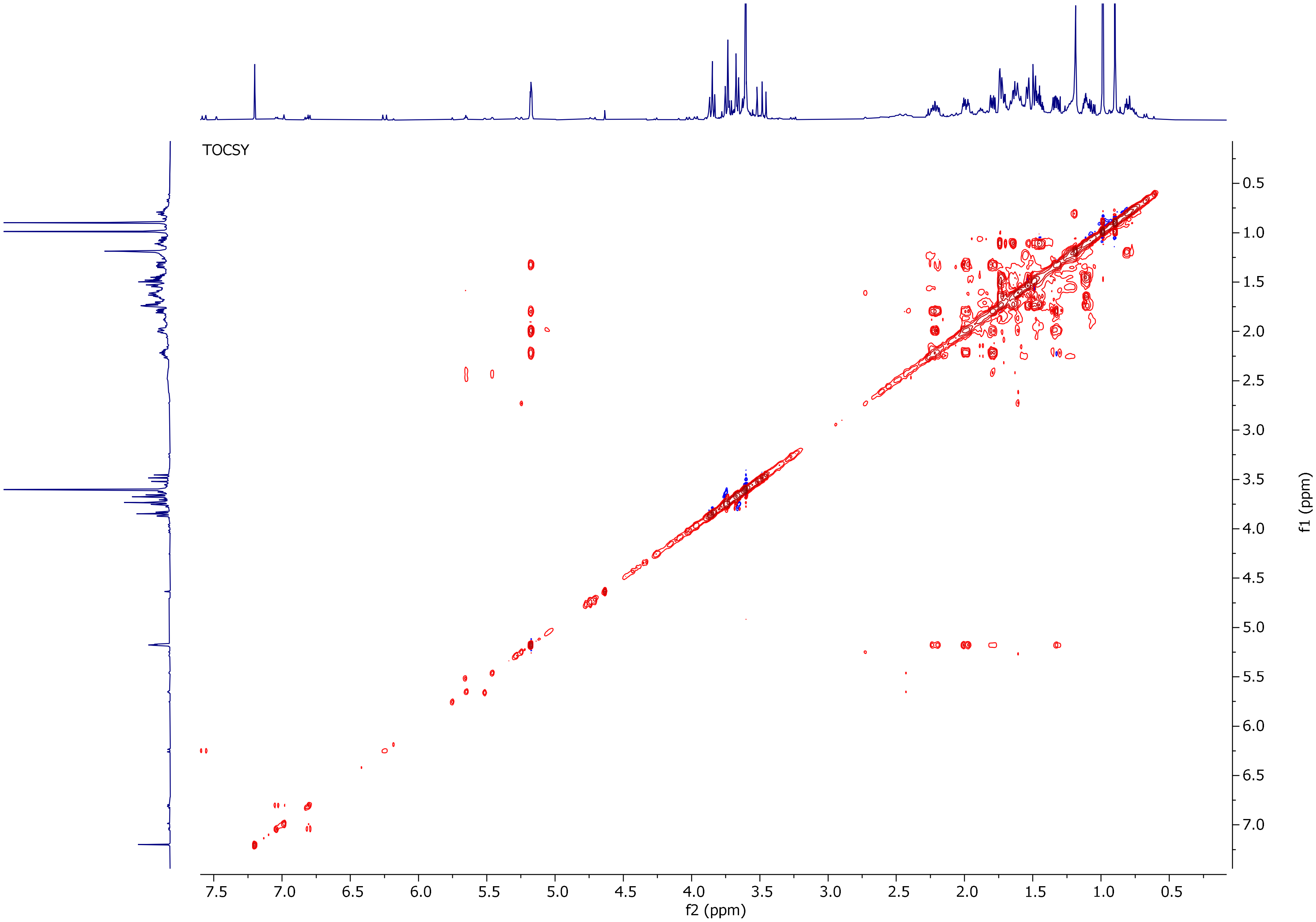
TOCSY
f2 (ppm)
f1 (ppm)
7.5
7.0
6.5
6.0
5.5
5.0
4.5
4.0
3.5
3.0
2.5
2.0
1.5
1.0
0.5

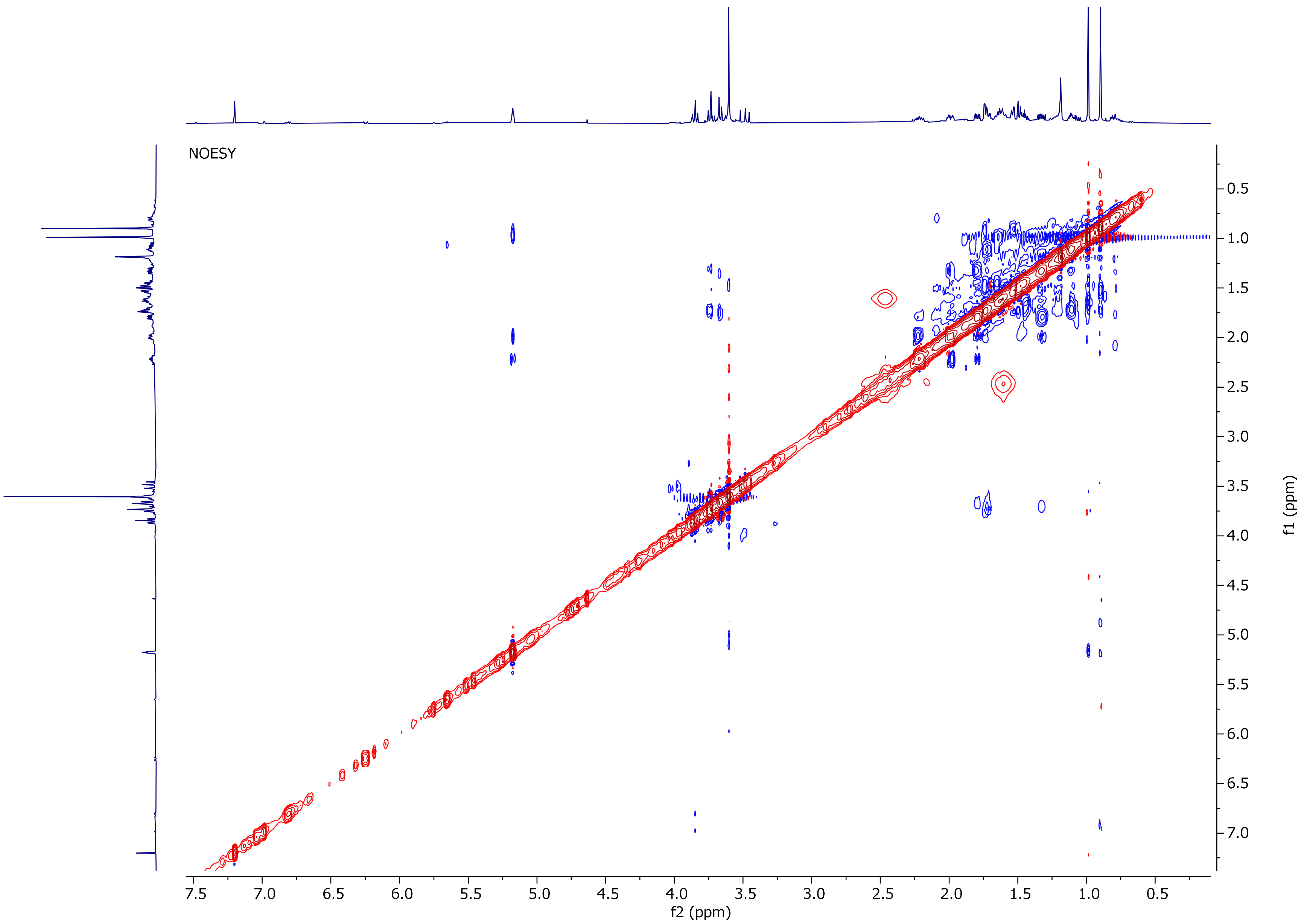
NOESY
f2 (ppm)
f1 (ppm)
7.5
7.0
6.5
6.0
5.5
5.0
4.5
4.0
3.5
3.0
2.5
2.0
1.5
1.0
0.5

^{1}H NMR DEPT 135, ^{13}C NMR, IR, HSQC, HMBC, COSY, TOCSY and NOESY of **12**

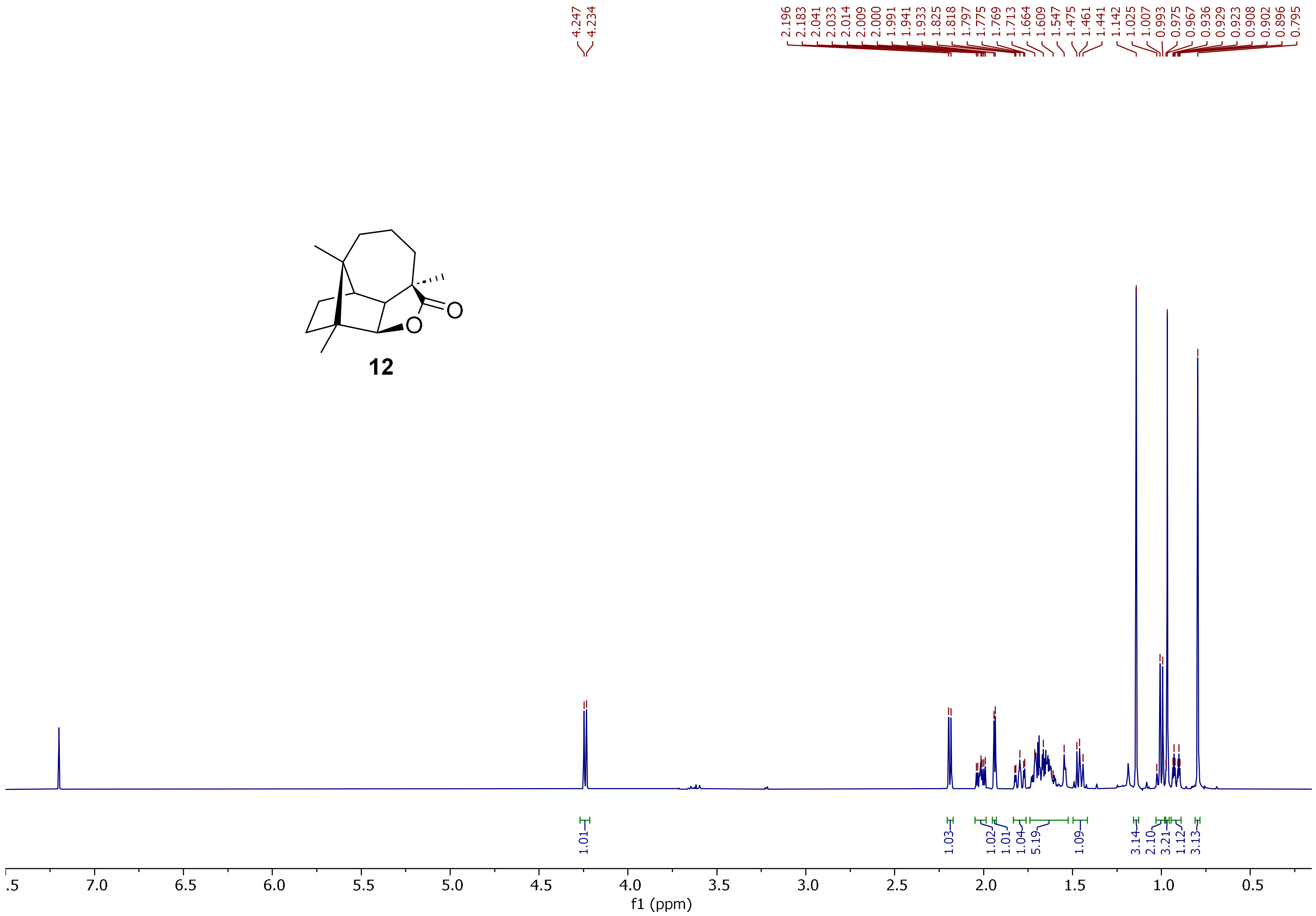

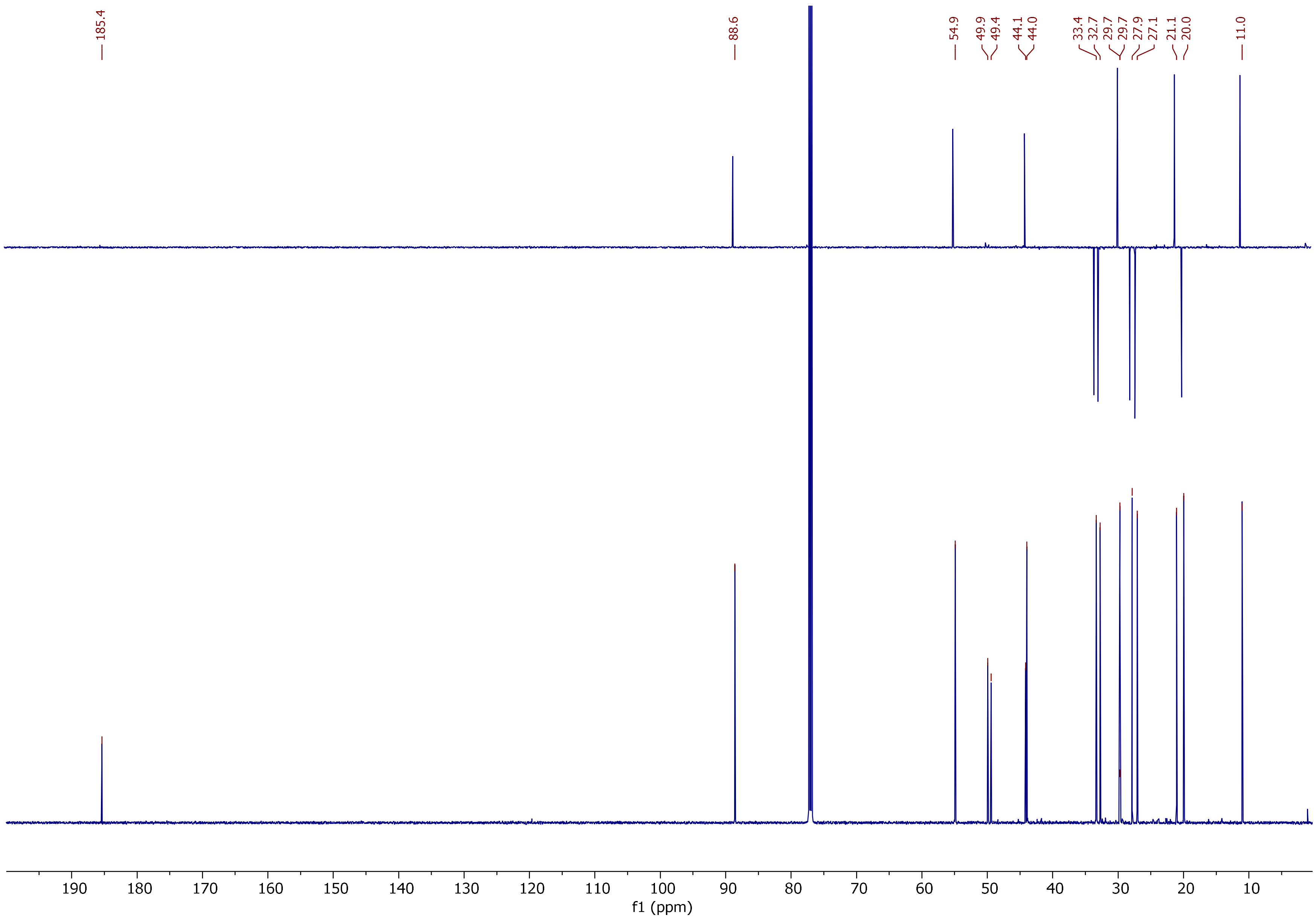
185.4
88.6
54.9
49.9
49.4
44.1
44.0
33.4
32.7
29.7
29.7
27.9
27.1
21.1
20.0
11.0
190 180 170 160 150 140 130 120 110 100 90 80 70 60 50 40 30 20 10
f1 (ppm)

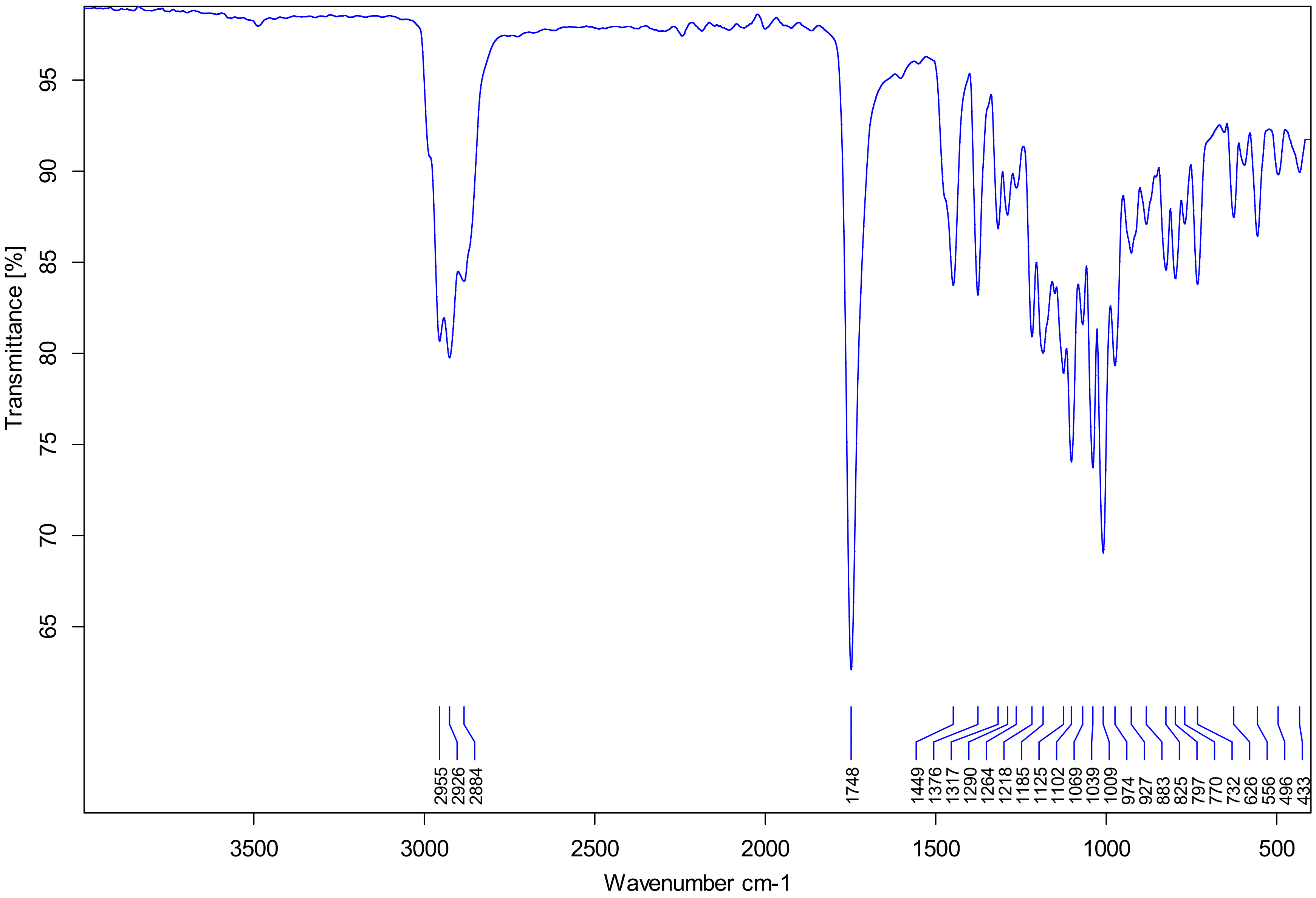
Transmittance [%]
65
70
75
80
85
90
95
Wavenumber cm-1
3500
3000
2500
2000
1500
1000
500
2955
2926
2884
1748
1449
1376
1317
1290
1264
1218
1185
1125
1102
1069
1039
1009
974
927
883
825
797
770
732
626
556
496
433

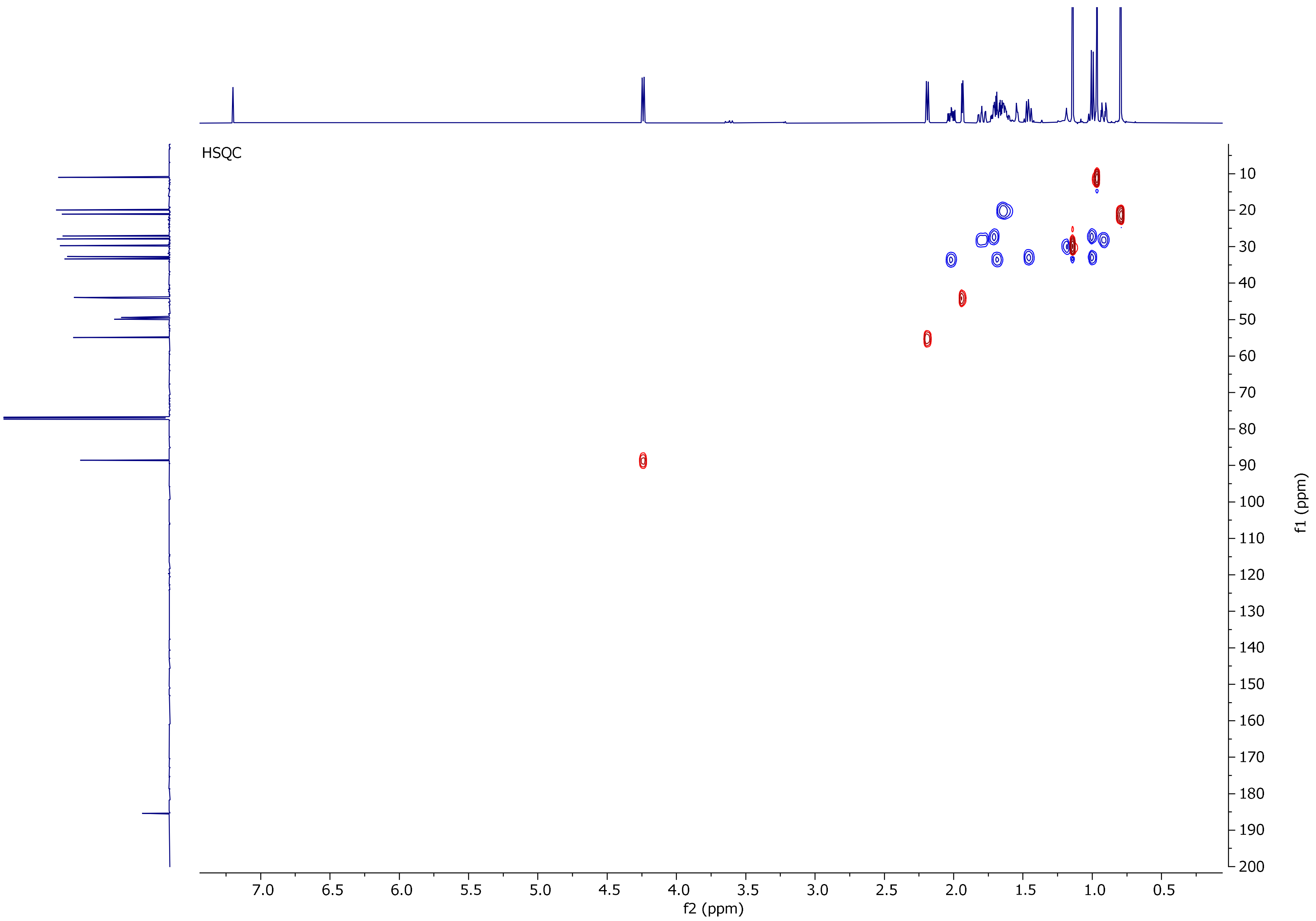
HSQC
7.0
6.5
6.0
5.5
5.0
4.5
4.0
3.5
3.0
2.5
2.0
1.5
1.0
0.5
f2 (ppm)
10
20
30
40
50
60
70
80
90
100
110
120
130
140
150
160
170
180
190
200
f1 (ppm)

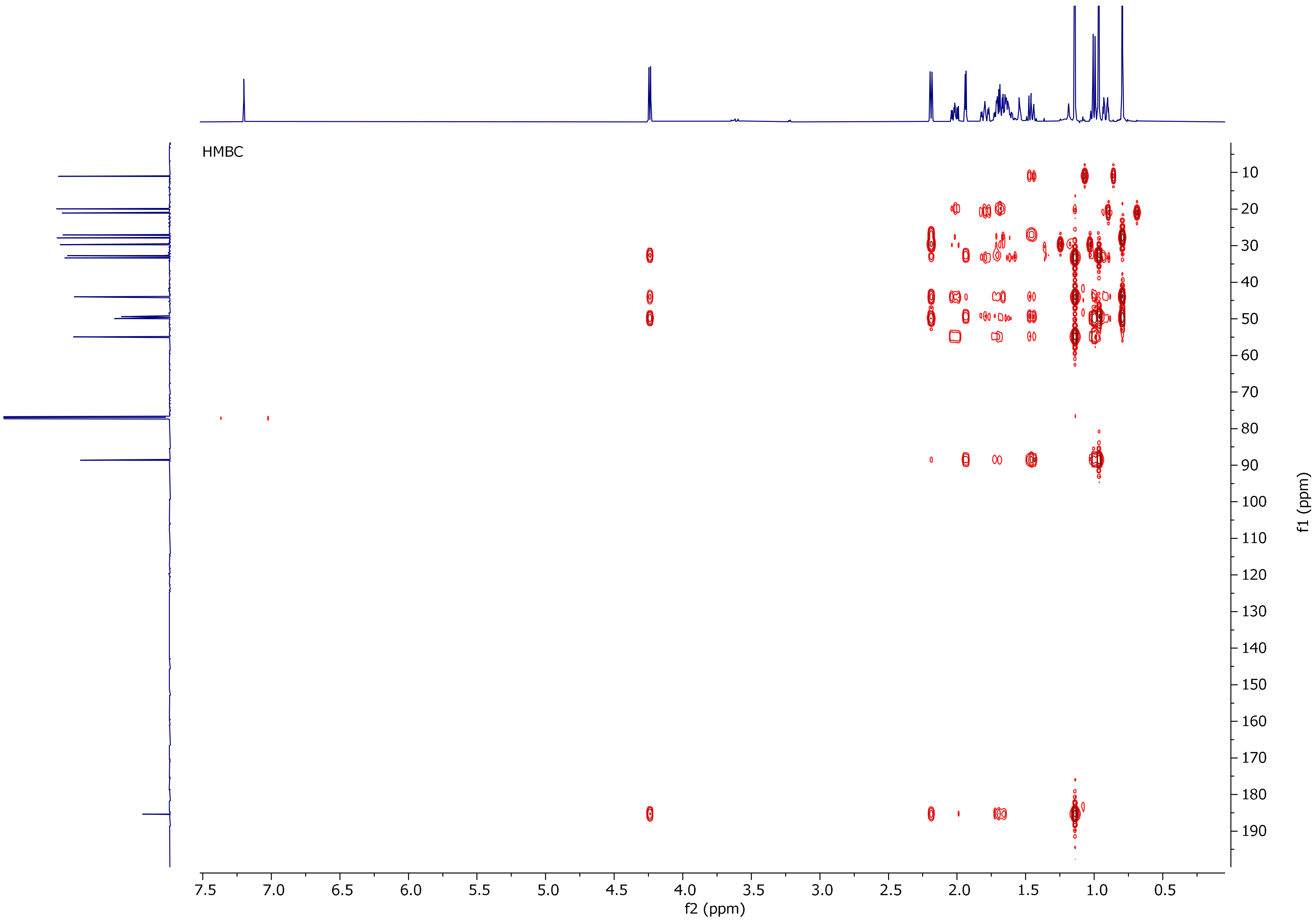
HMBC
f2 (ppm)
f1 (ppm)

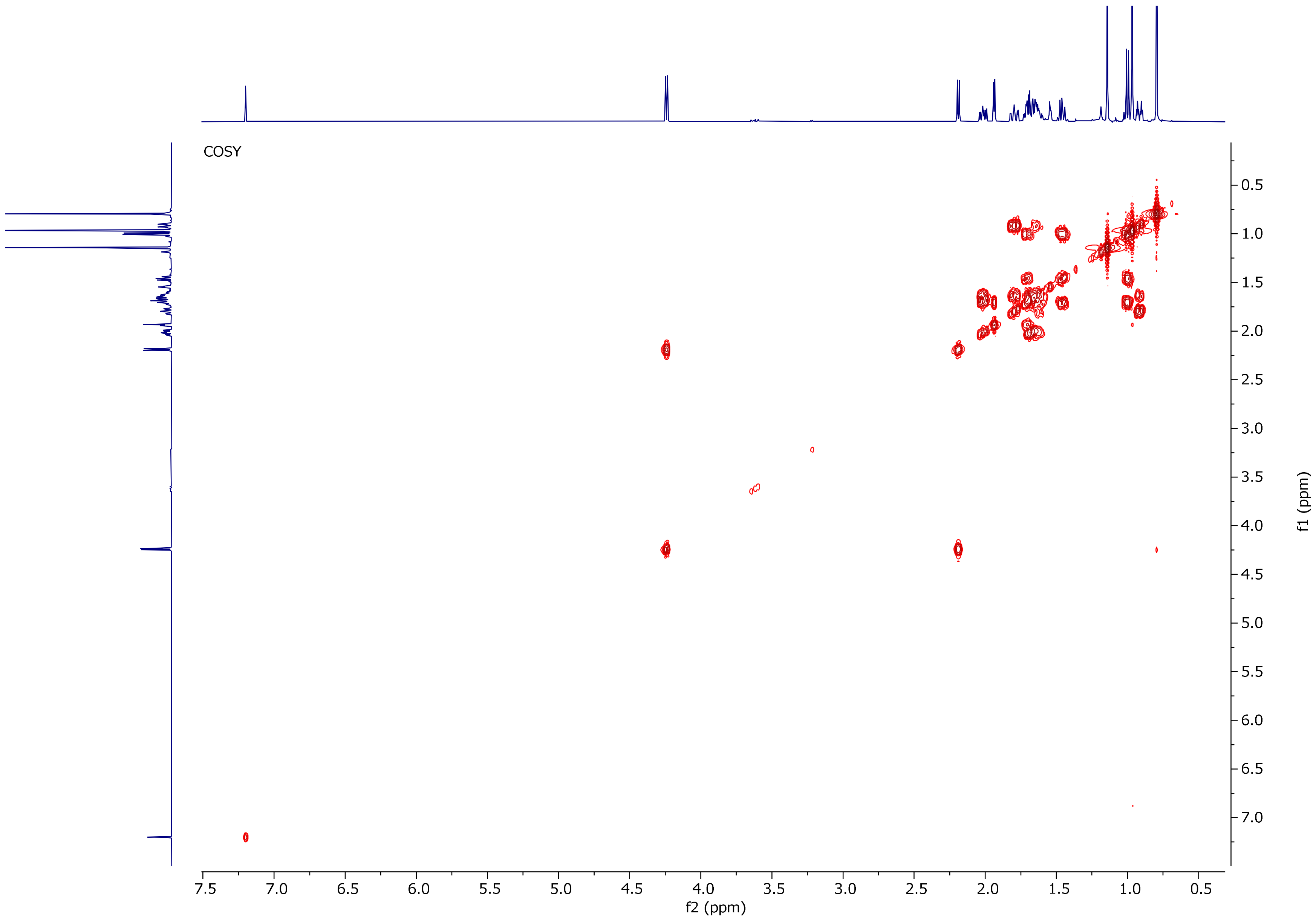
COSY
7.5 7.0 6.5 6.0 5.5 5.0 4.5 4.0 3.5 3.0 2.5 2.0 1.5 1.0 0.5
f2 (ppm)
0.5 1.0 1.5 2.0 2.5 3.0 3.5 4.0 4.5 5.0 5.5 6.0 6.5 7.0
f1 (ppm)

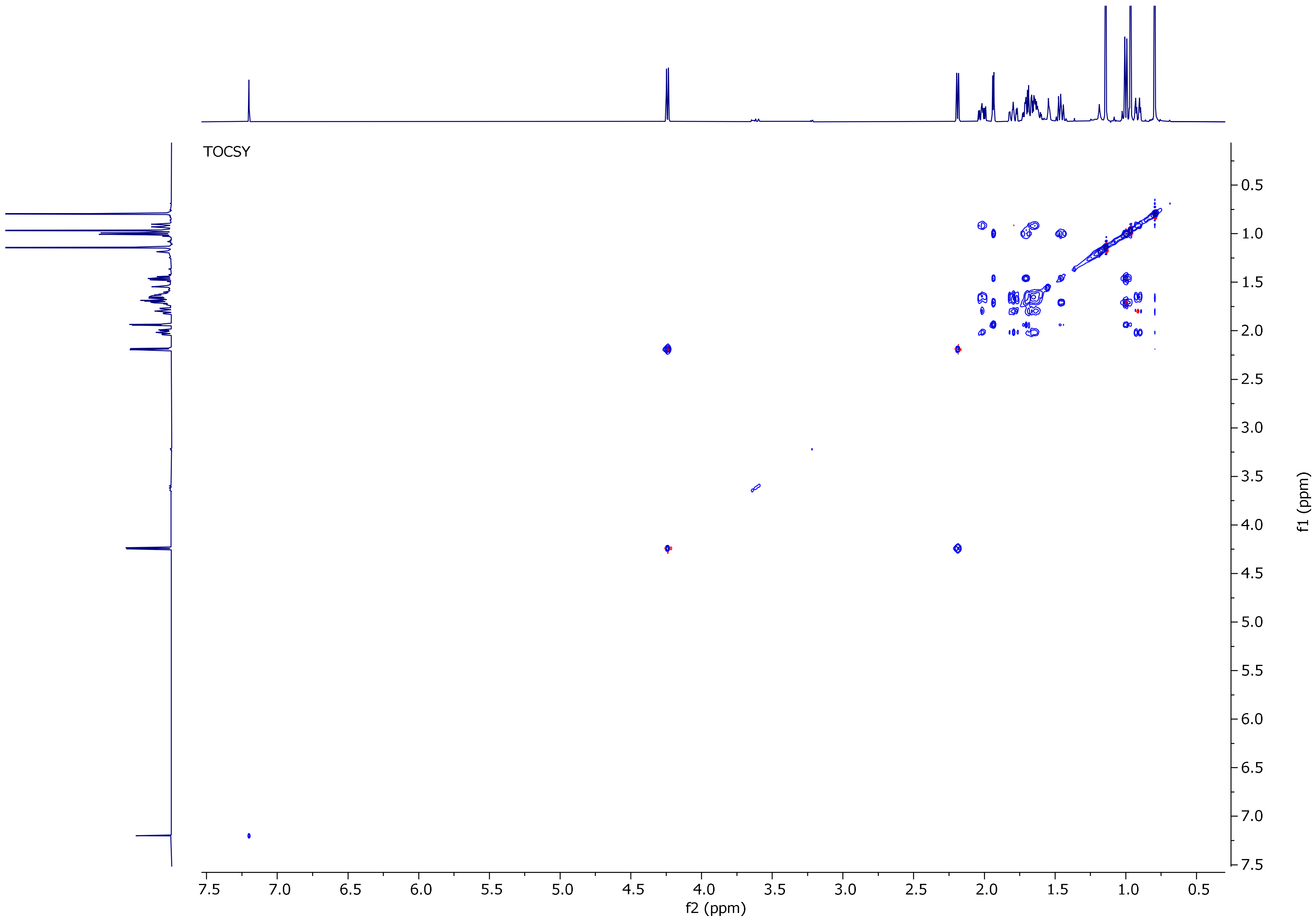
TOCSY
f2 (ppm)
f1 (ppm)

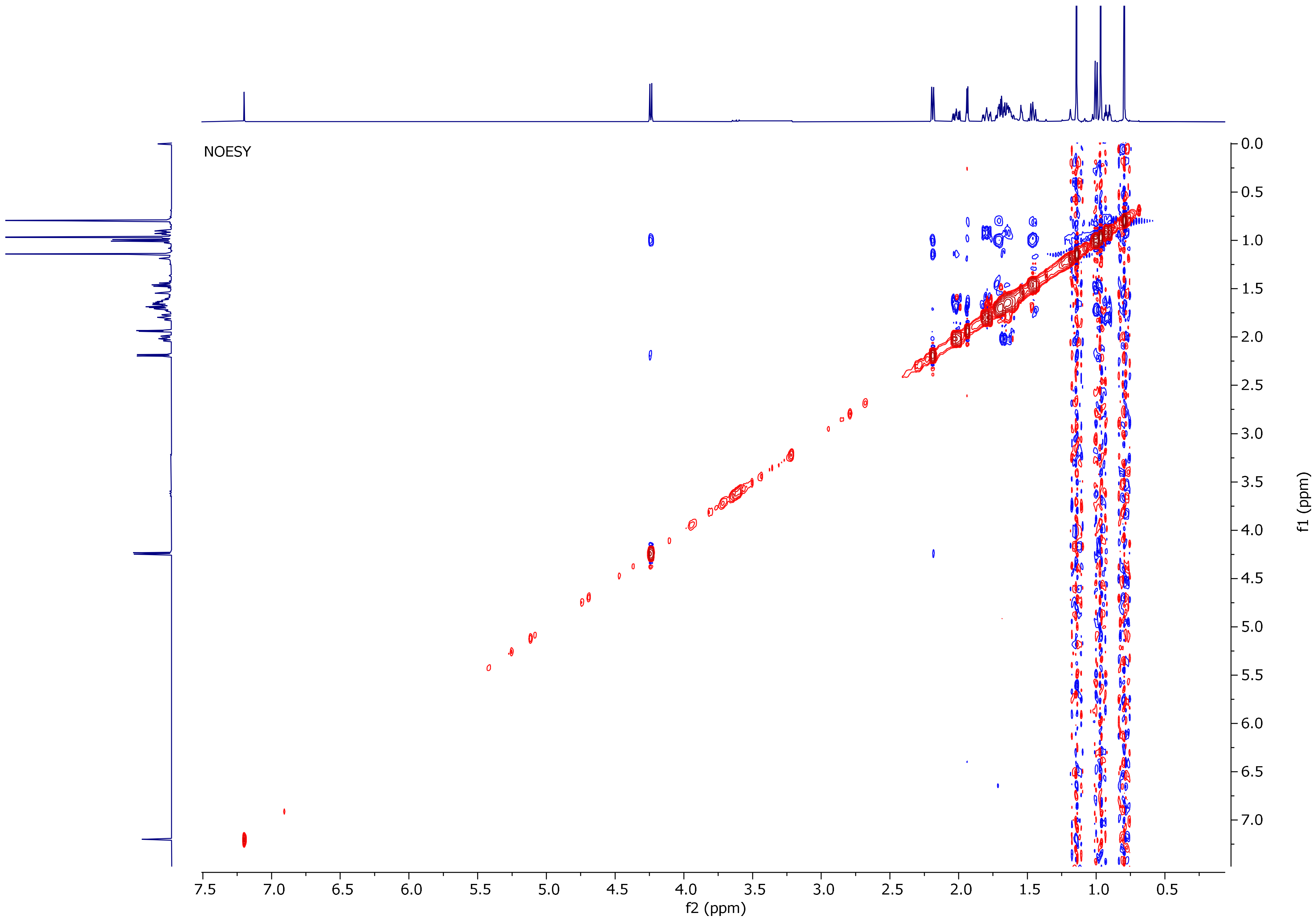
NOESY
f2 (ppm)
f1 (ppm)
7.5
7.0
6.5
6.0
5.5
5.0
4.5
4.0
3.5
3.0
2.5
2.0
1.5
1.0
0.5
0.0

^{1}H NMR, DEPT 135, ^{13}C NMR, IR, HSQC, HMBC, COSY, TOCSY and NOESY of **13**

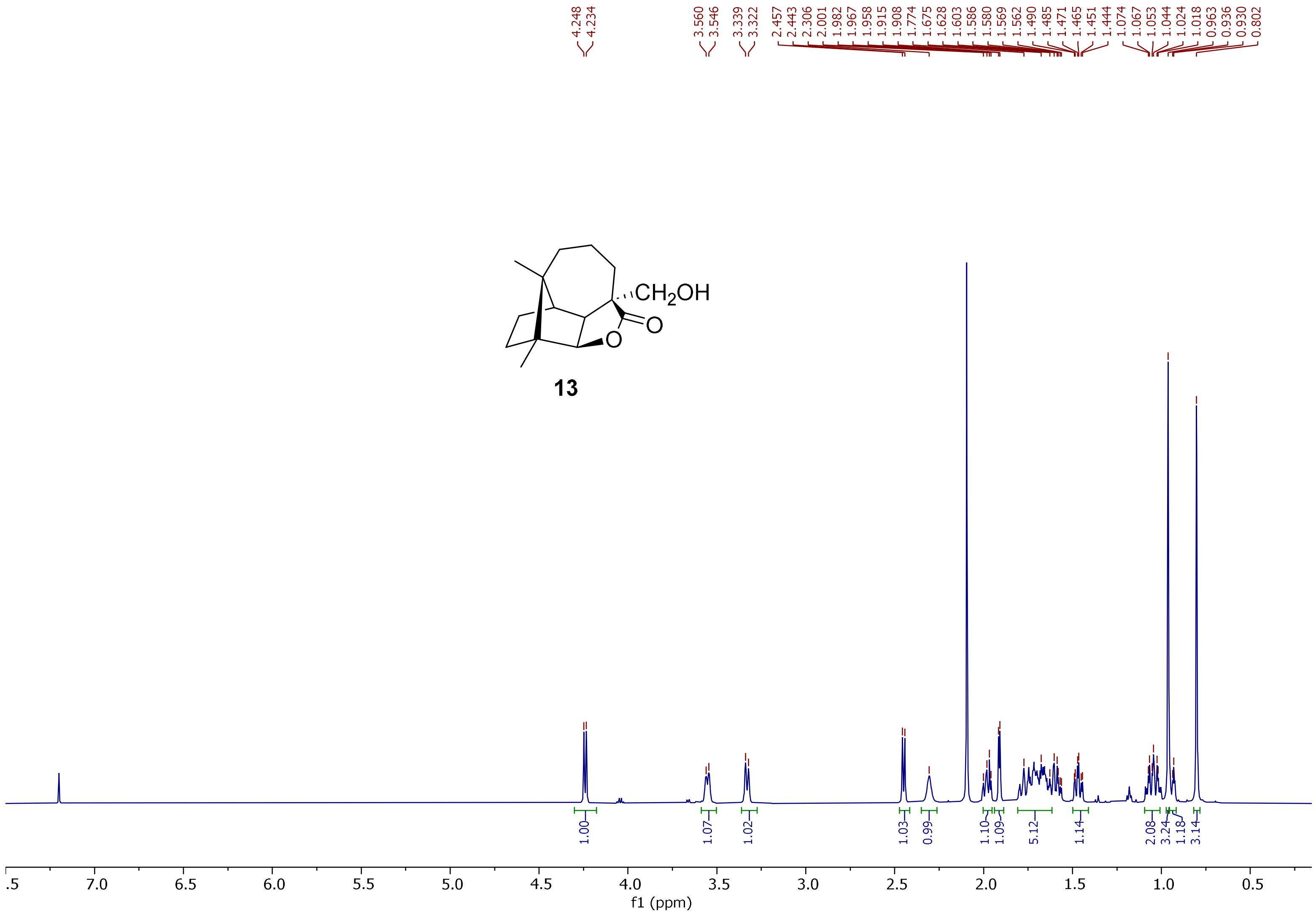

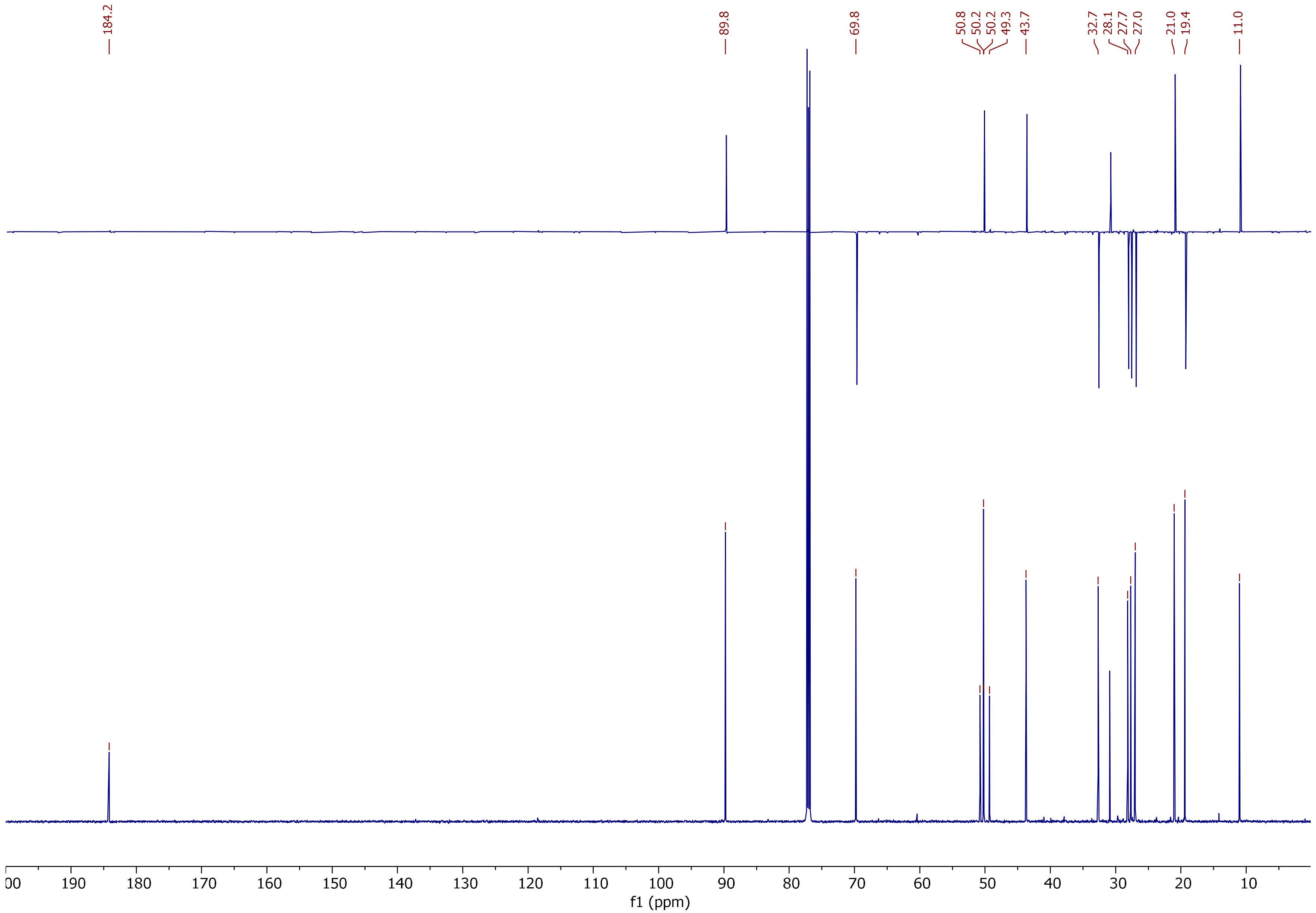
184.2
89.8
69.8
50.8
50.2
50.2
49.3
43.7
32.7
28.1
27.7
27.0
21.0
19.4
11.0
00
190
180
170
160
150
140
130
120
110
100
90
80
70
60
50
40
30
20
10
f1 (ppm)

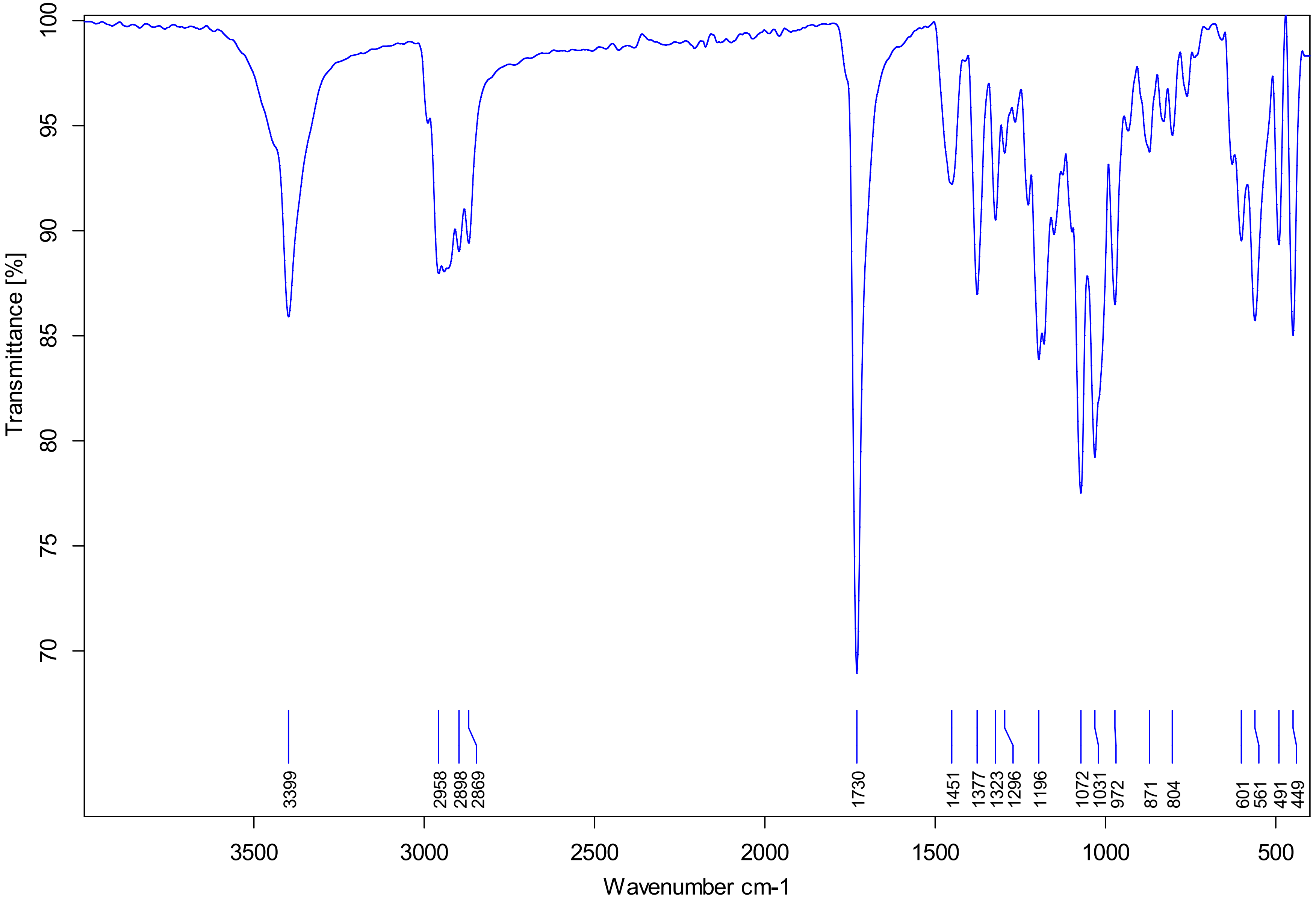
Transmittance [%]
100
95
90
85
80
75
70
3399
2958
2898
2869
1730
1451
1377
1323
1296
1196
1072
1031
972
871
804
601
561
491
449
3500
3000
2500
2000
1500
1000
500
Wavenumber cm-1

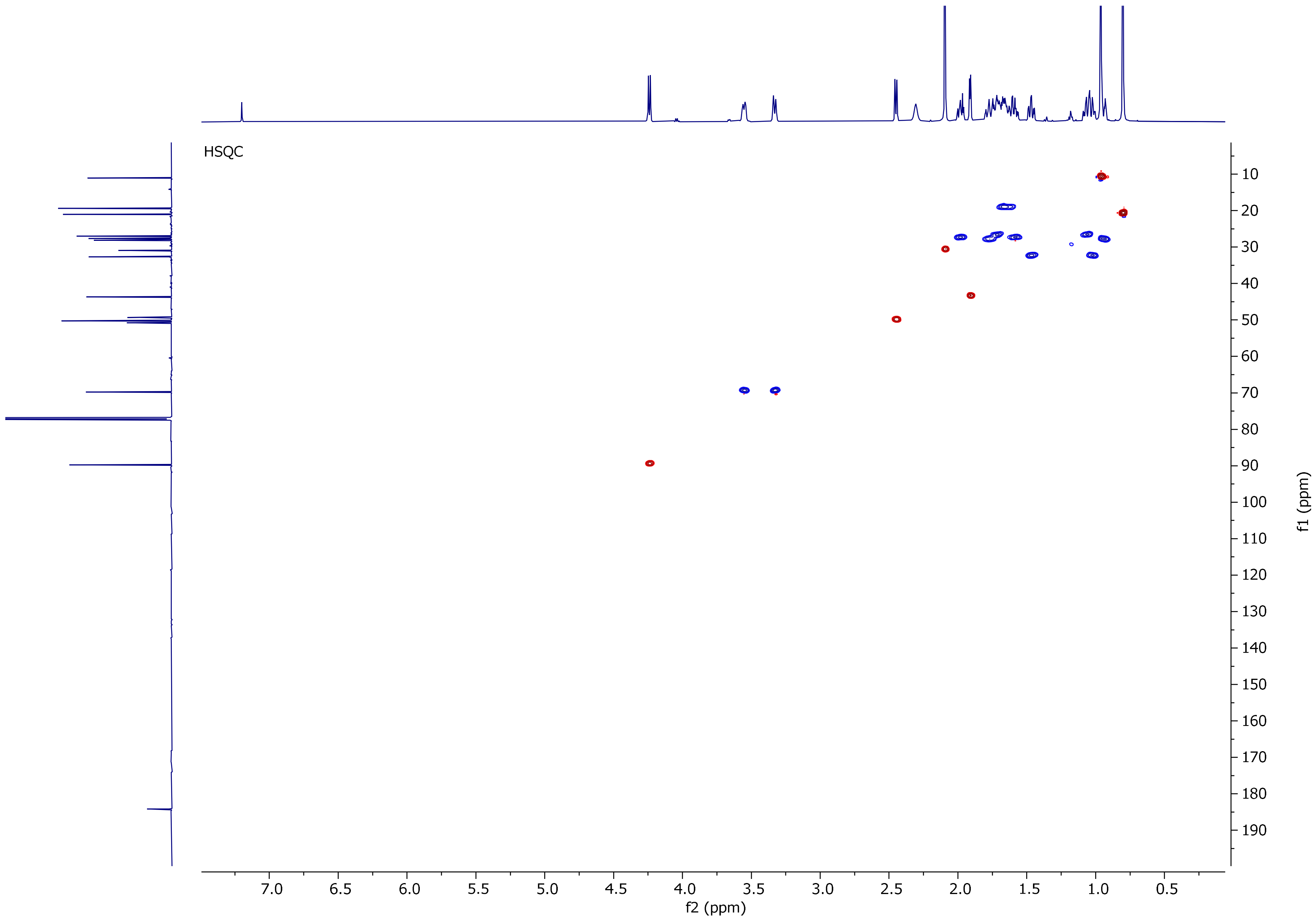
HSQC
f2 (ppm)
f1 (ppm)

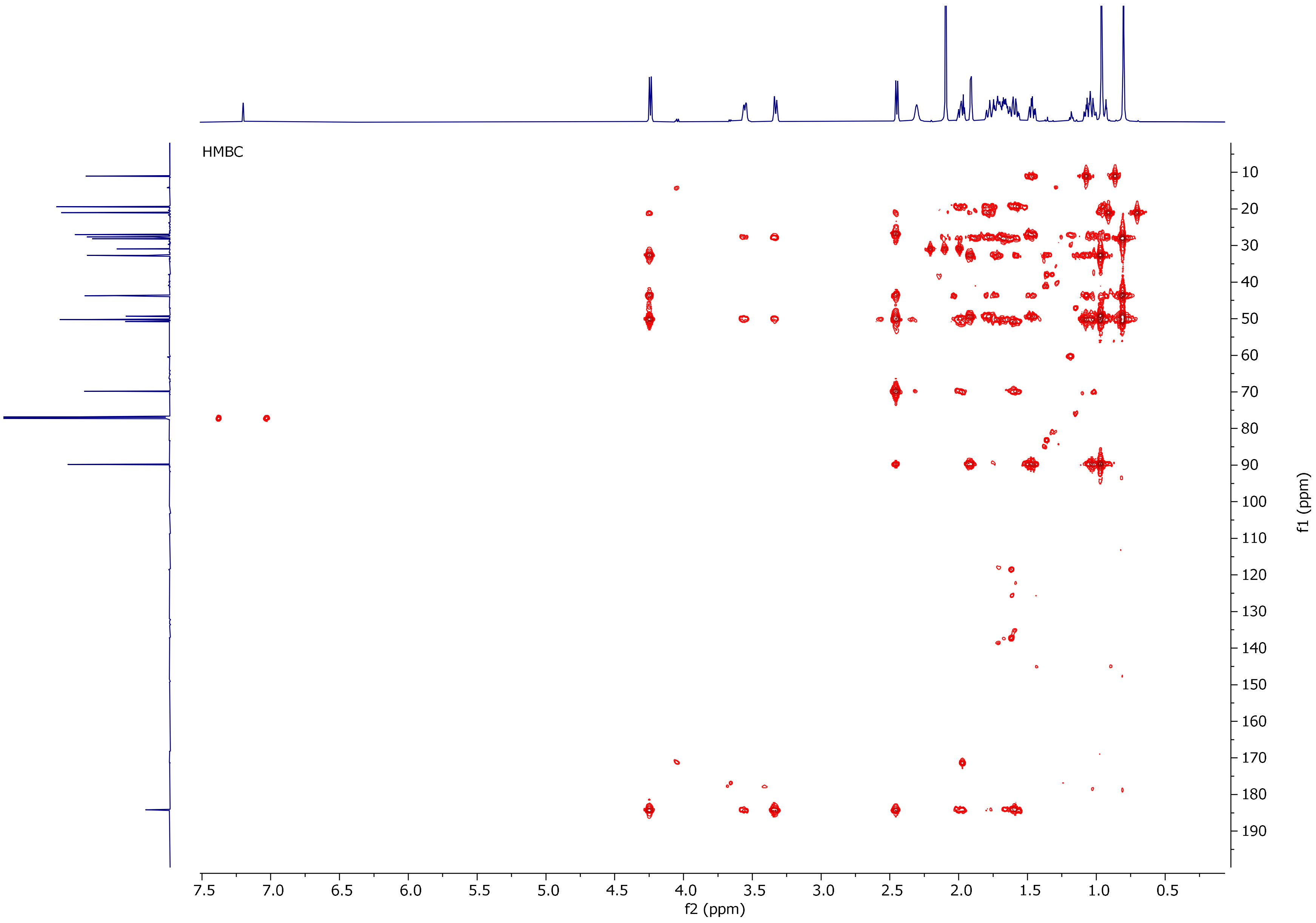
HMBC
f2 (ppm)
f1 (ppm)

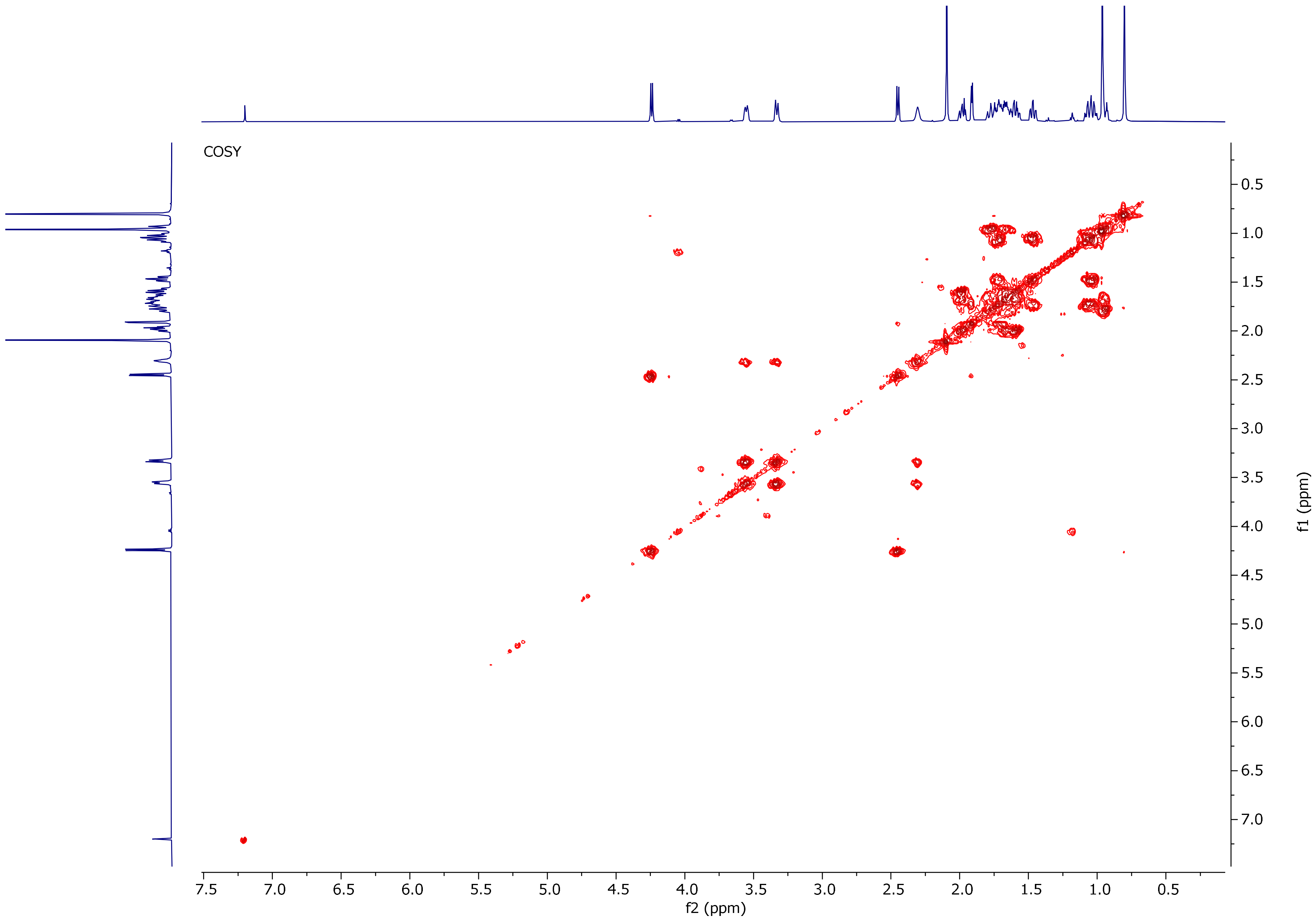
COSY
7.5
7.0
6.5
6.0
5.5
5.0
4.5
4.0
3.5
3.0
2.5
2.0
1.5
1.0
0.5
f2 (ppm)
f1 (ppm)

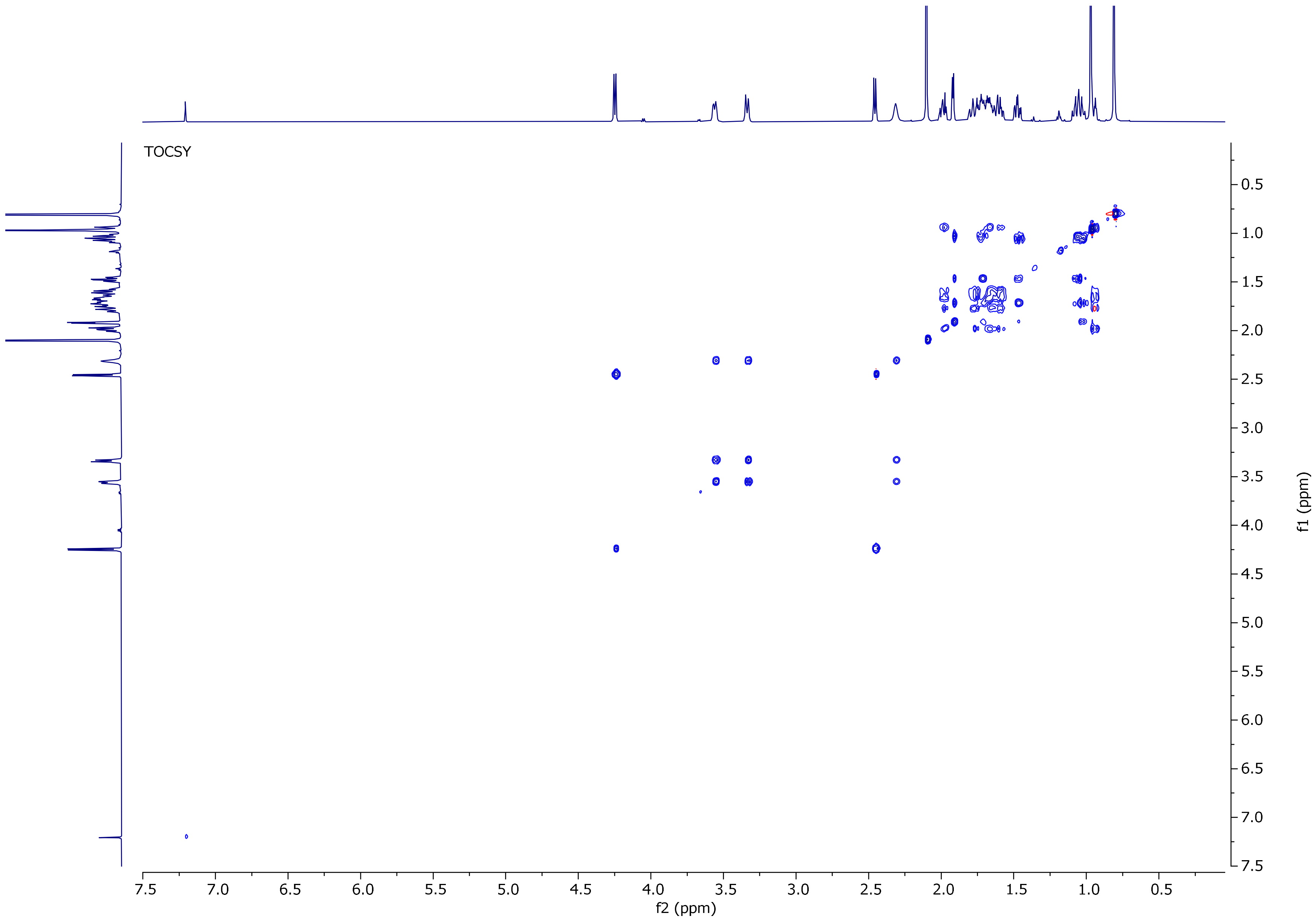
TOCSY
f2 (ppm)
f1 (ppm)
7.5
7.0
6.5
6.0
5.5
5.0
4.5
4.0
3.5
3.0
2.5
2.0
1.5
1.0
0.5

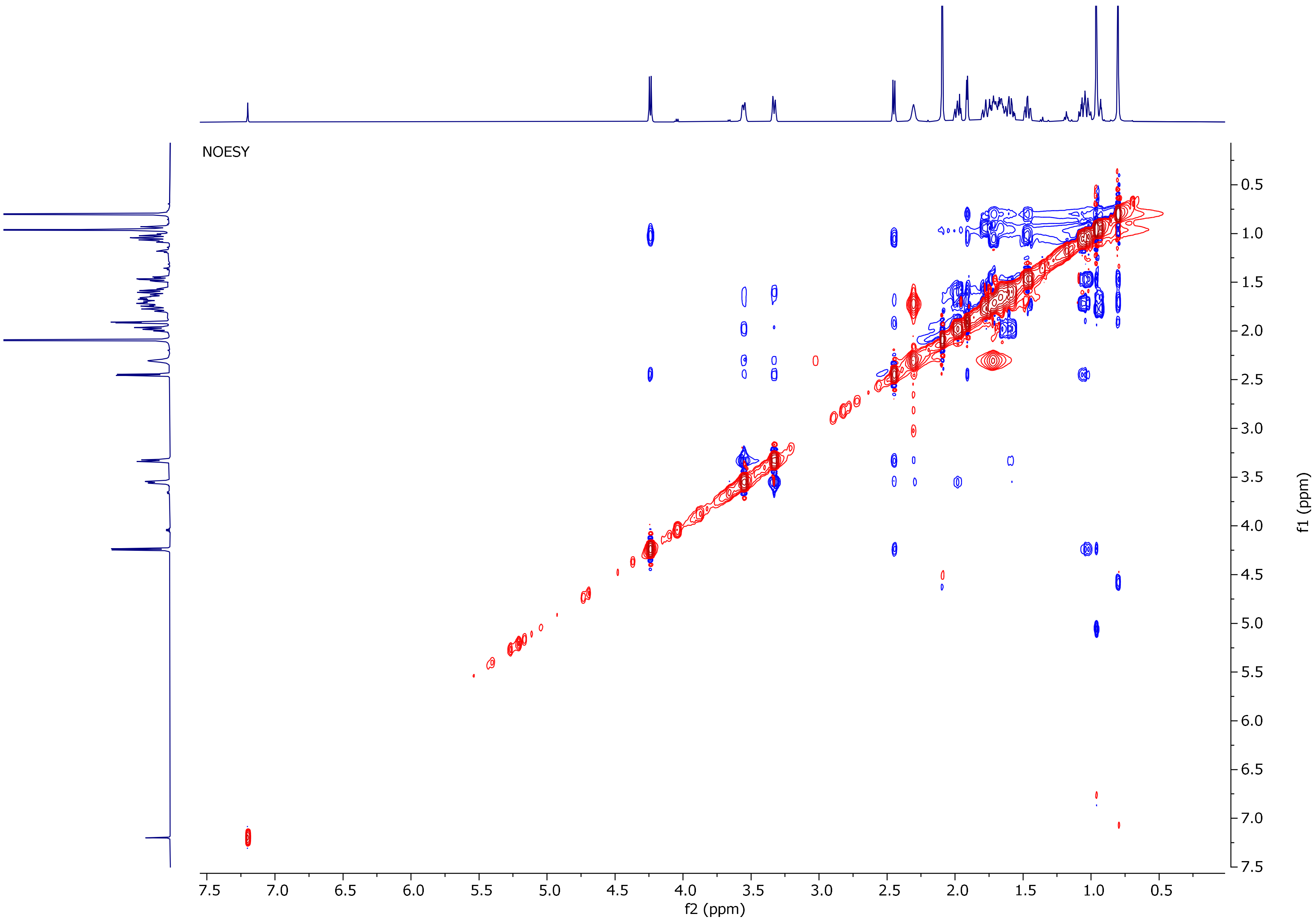
NOESY
f2 (ppm)
f1 (ppm)
7.5
7.0
6.5
6.0
5.5
5.0
4.5
4.0
3.5
3.0
2.5
2.0
1.5
1.0
0.5

¹H NMR, DEPT 135, ¹³C NMR, IR, HSQC, HMBC, COSY, TOCSY and NOESY of **14**

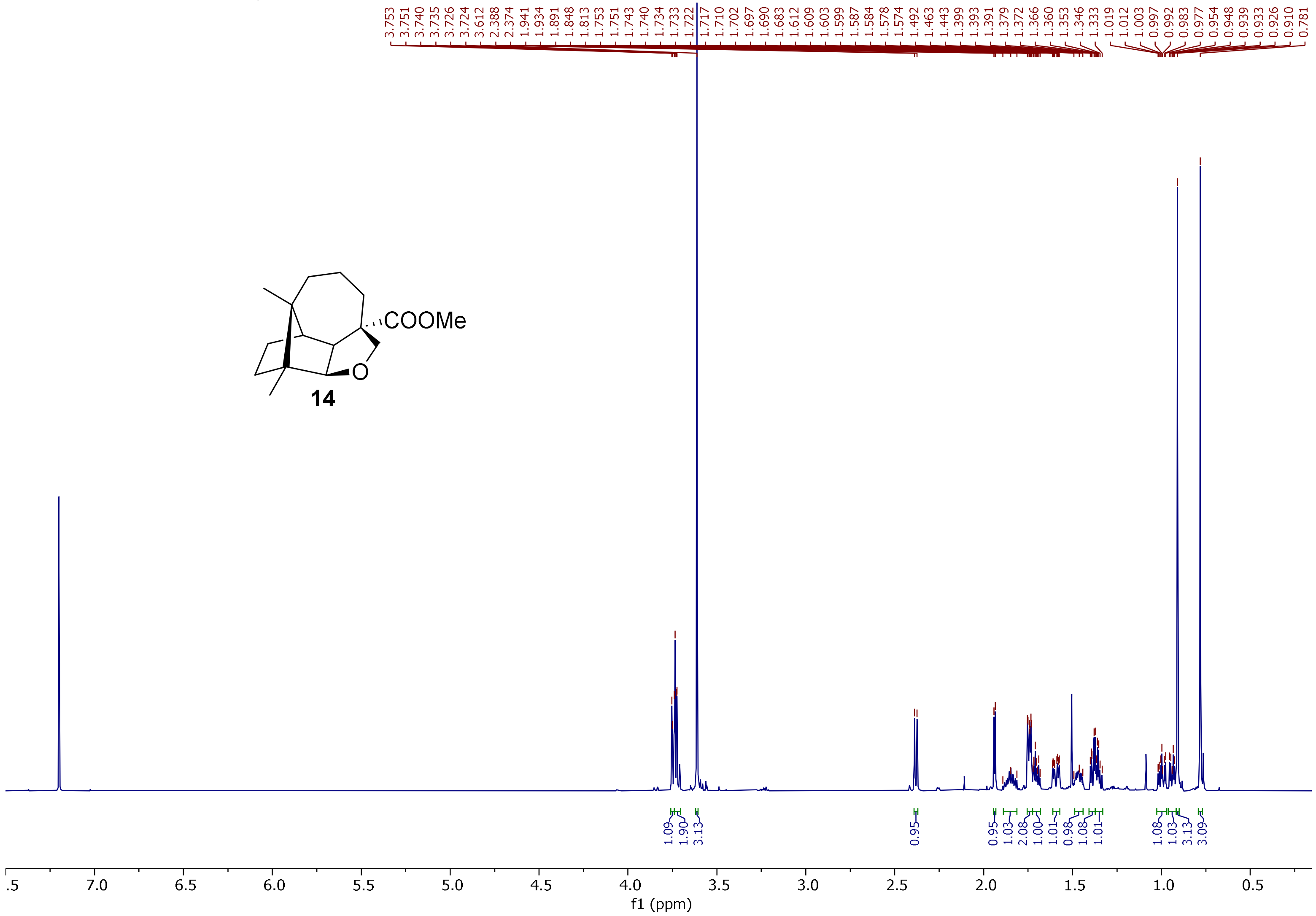

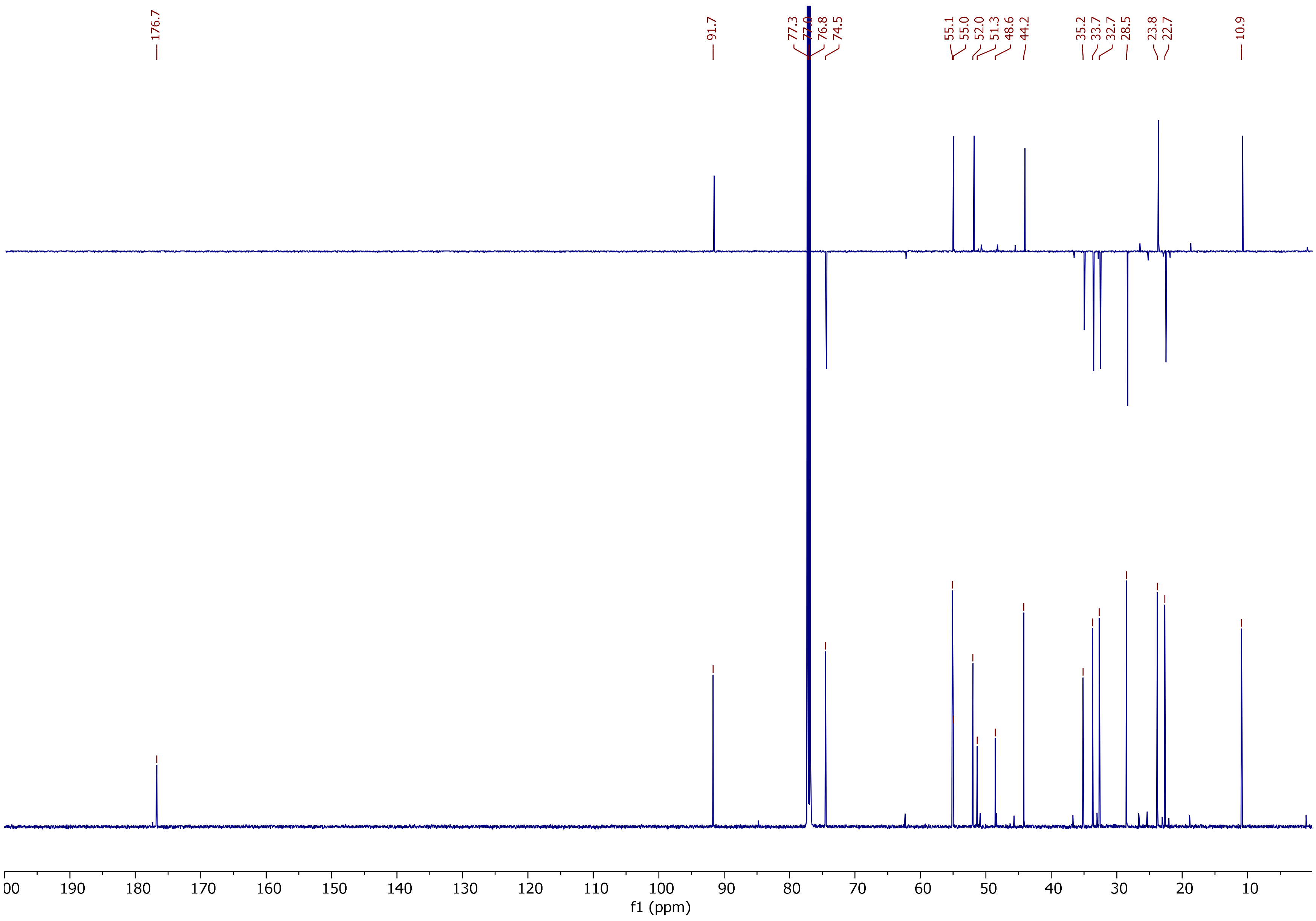
176.7
91.7
77.3
77.0
76.8
74.5
55.1
55.0
52.0
51.3
48.6
44.2
35.2
33.7
32.7
28.5
23.8
22.7
10.9
190 180 170 160 150 140 130 120 110 100 90 80 70 60 50 40 30 20 10
f1 (ppm)

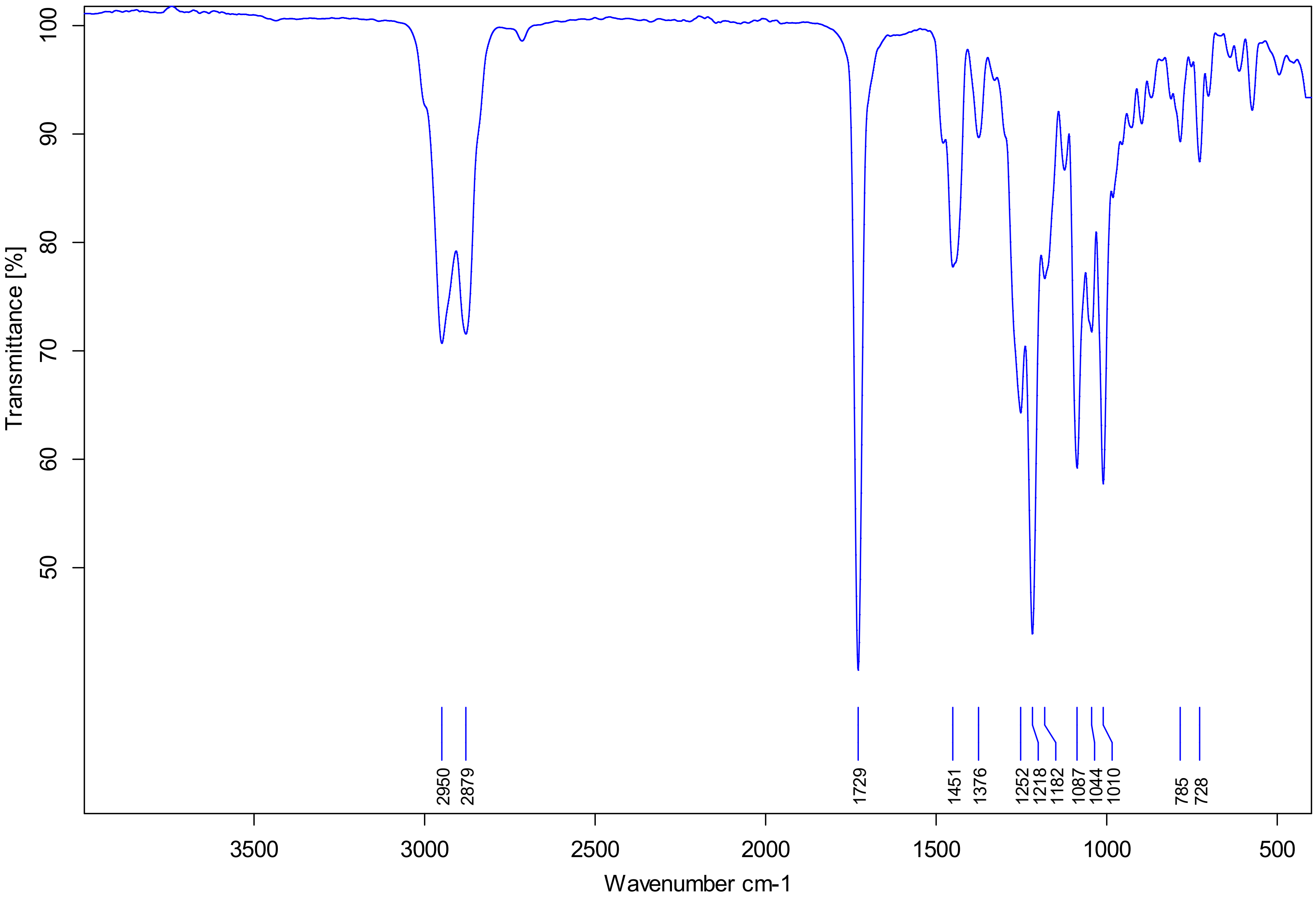
Transmittance [%]
100
90
80
70
60
50
2950
2879
1729
1451
1376
1252
1218
1182
1087
1044
1010
785
728
3500
3000
2500
2000
1500
1000
500
Wavenumber cm-1

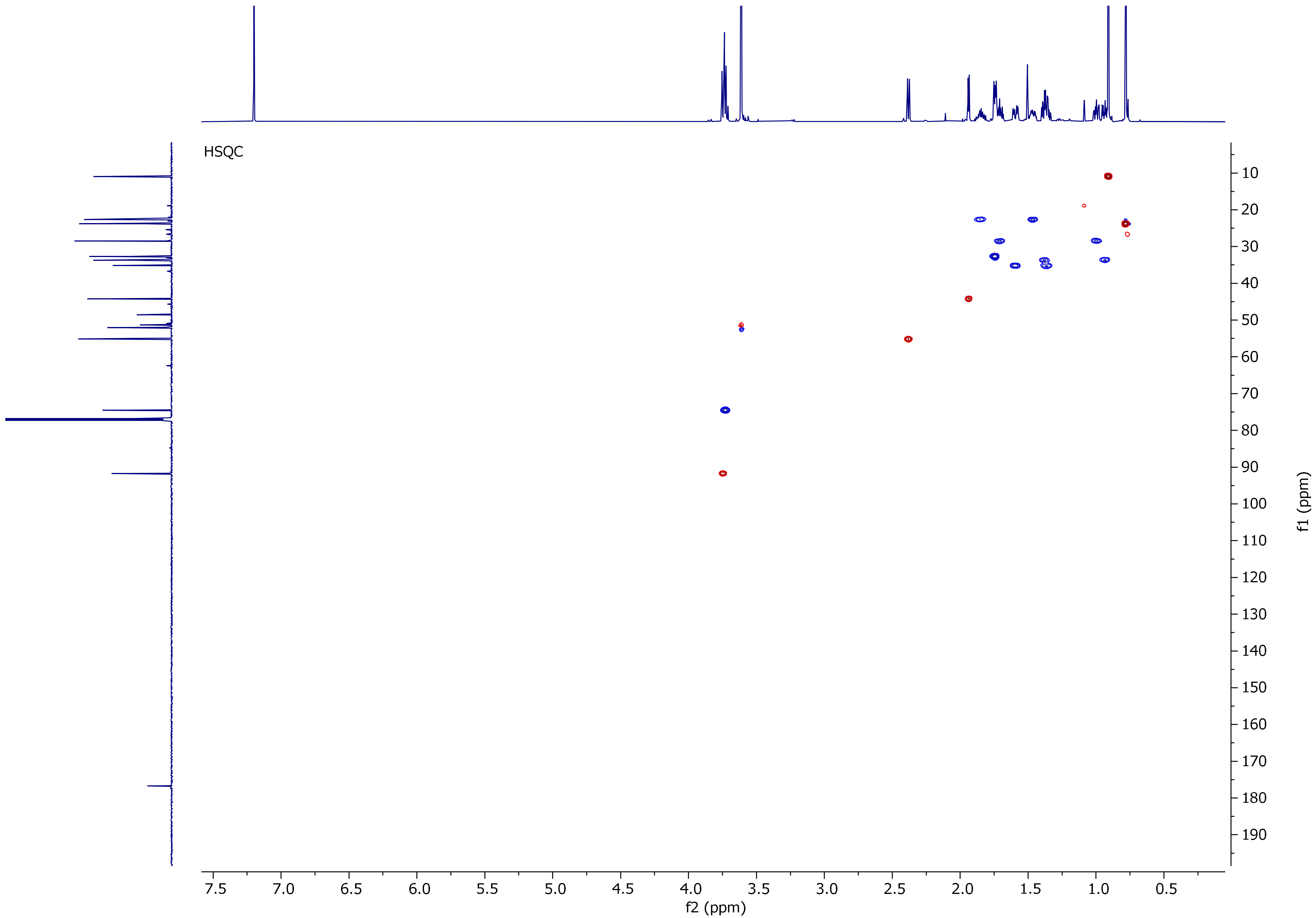
HSQC
7.5
7.0
6.5
6.0
5.5
5.0
4.5
4.0
3.5
3.0
2.5
2.0
1.5
1.0
0.5
f2 (ppm)
10
20
30
40
50
60
70
80
90
100
110
120
130
140
150
160
170
180
190
f1 (ppm)

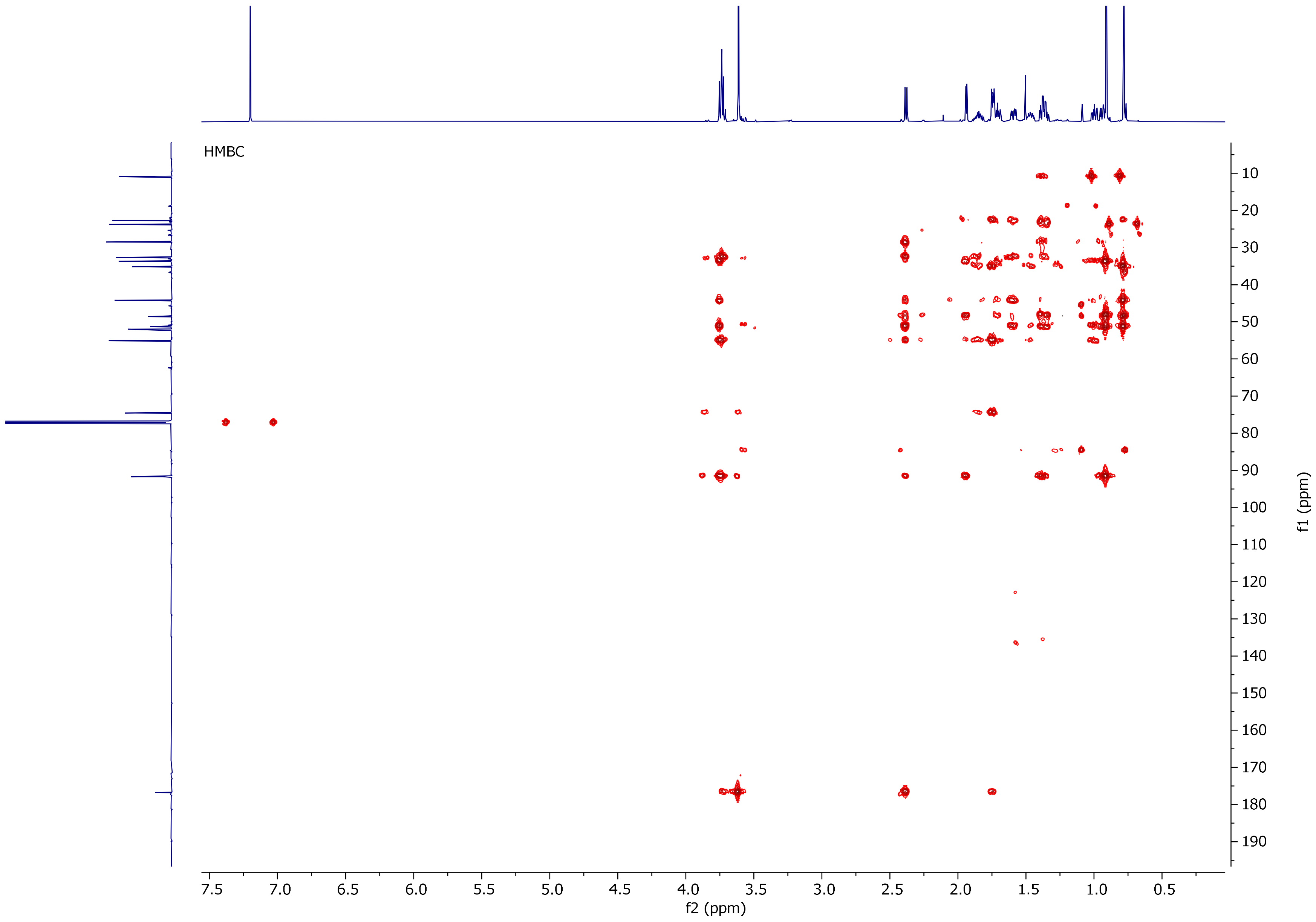
HMBC
f2 (ppm)
f1 (ppm)

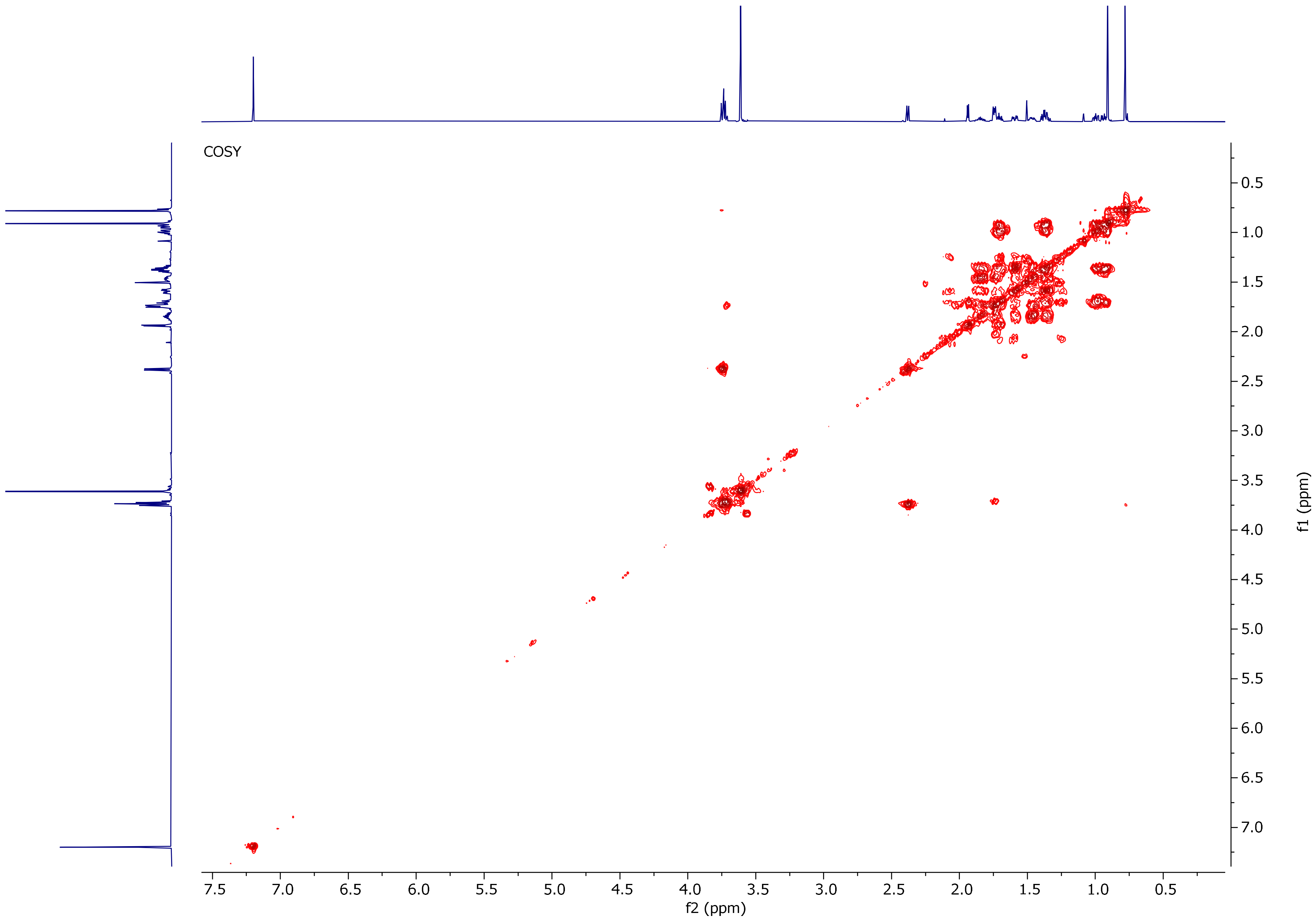
COSY
7.5
7.0
6.5
6.0
5.5
5.0
4.5
4.0
3.5
3.0
2.5
2.0
1.5
1.0
0.5
f2 (ppm)
f1 (ppm)

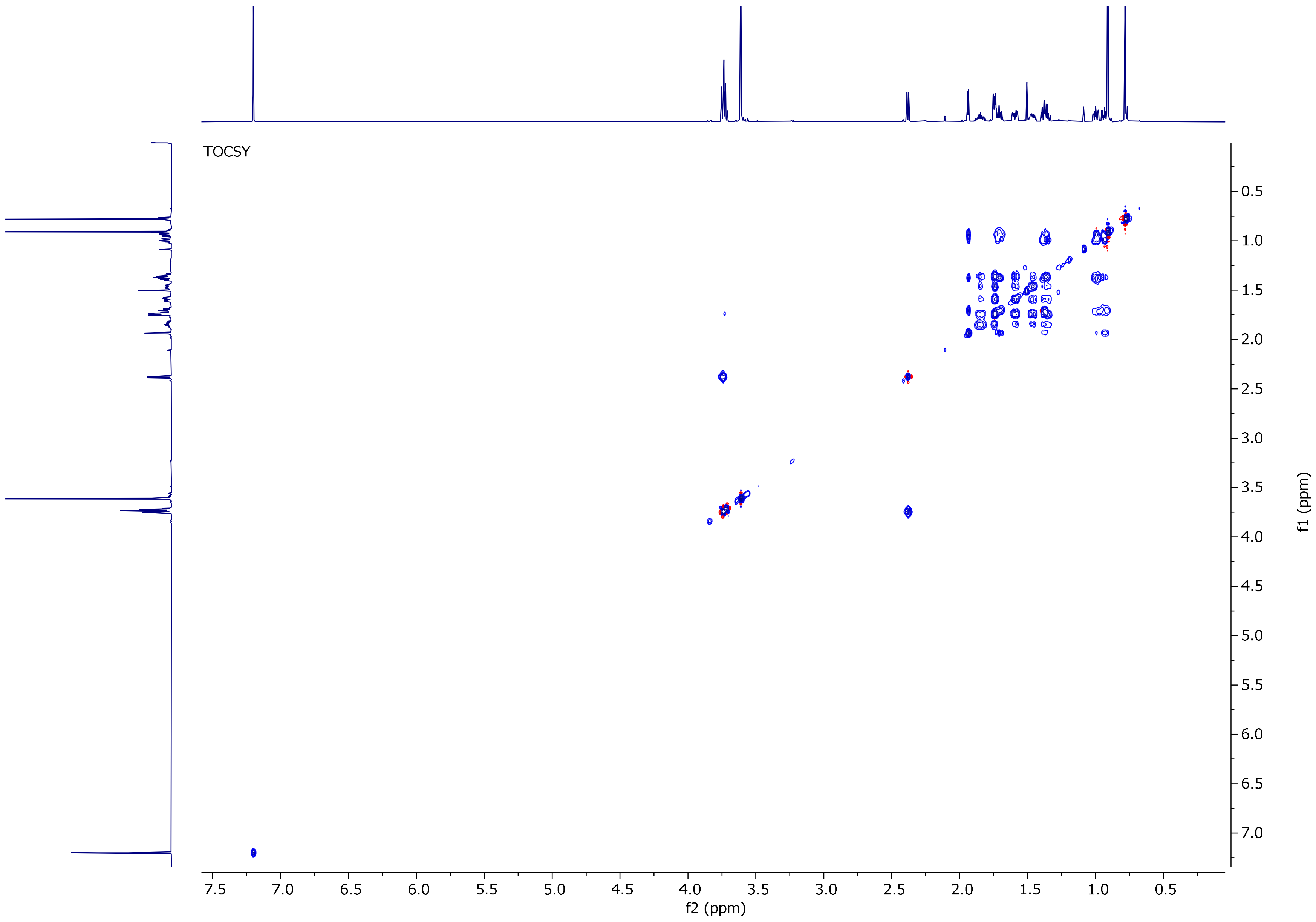
TOCSY
f2 (ppm)
f1 (ppm)

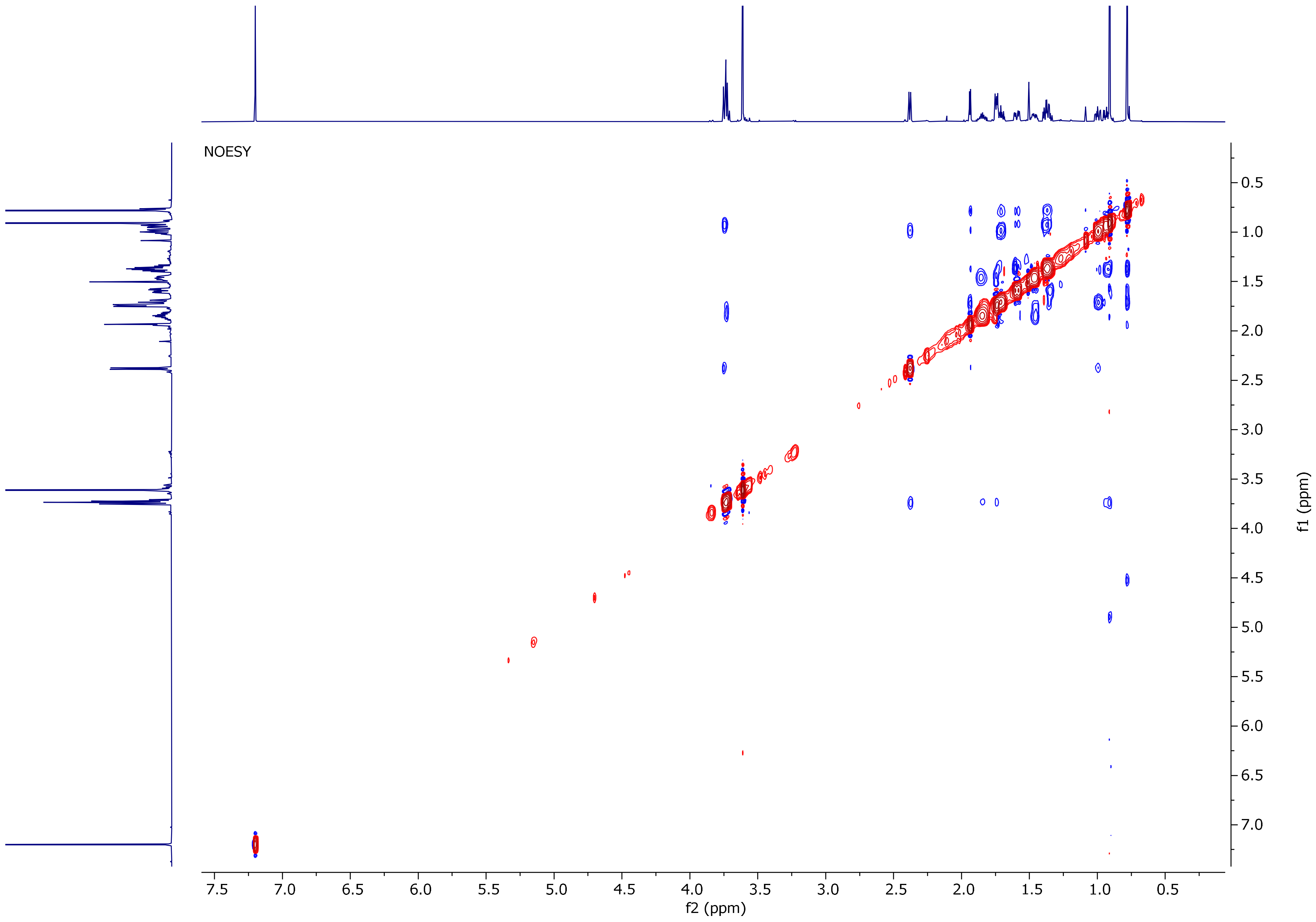
NOESY
7.5
7.0
6.5
6.0
5.5
5.0
4.5
4.0
3.5
3.0
2.5
2.0
1.5
1.0
0.5
f2 (ppm)
f1 (ppm)

^{1}H NMR, DEPT 135, ^{13}C NMR, IR, HSQC, HMBC, COSY, TOCSY and NOESY of **15**

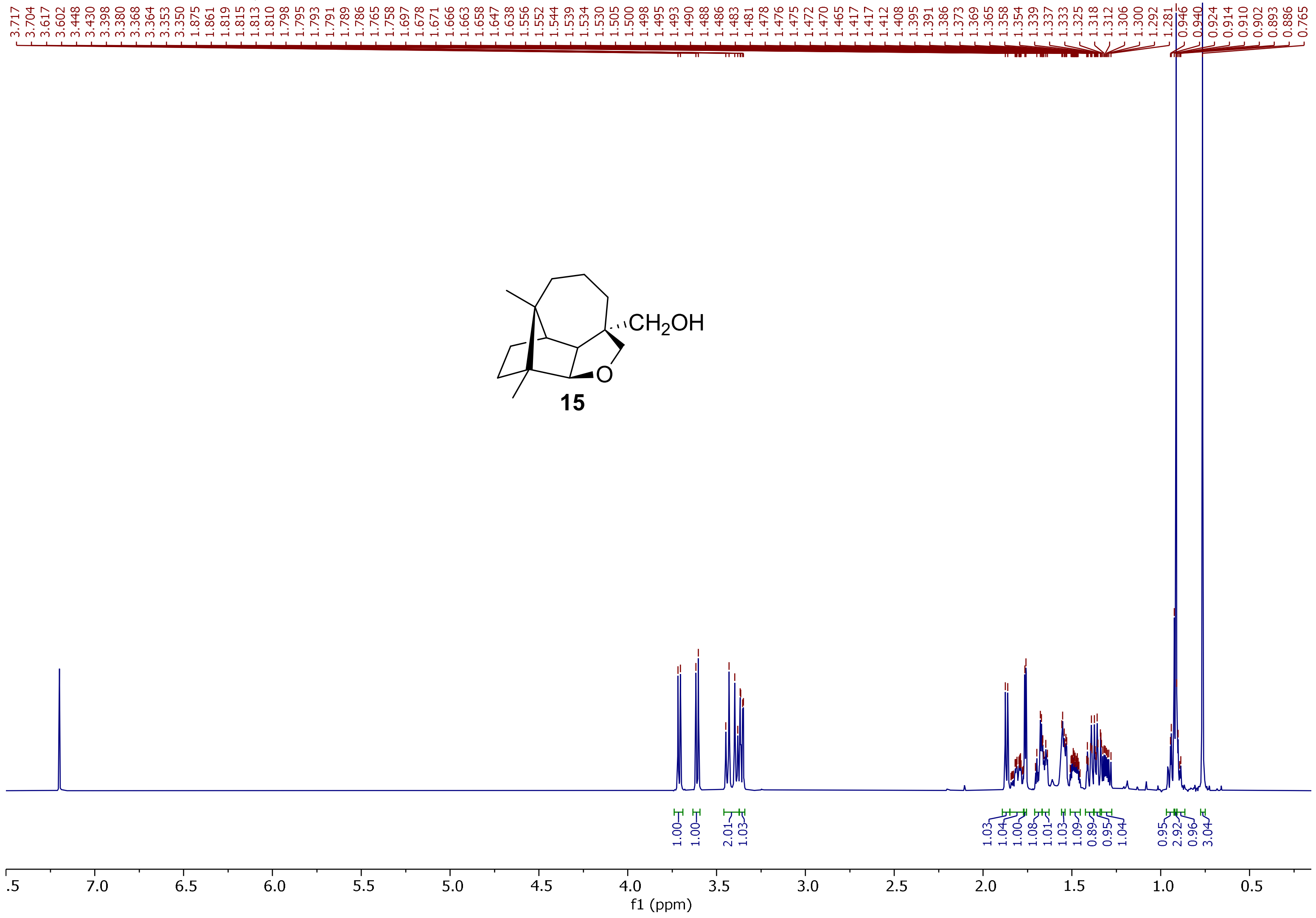

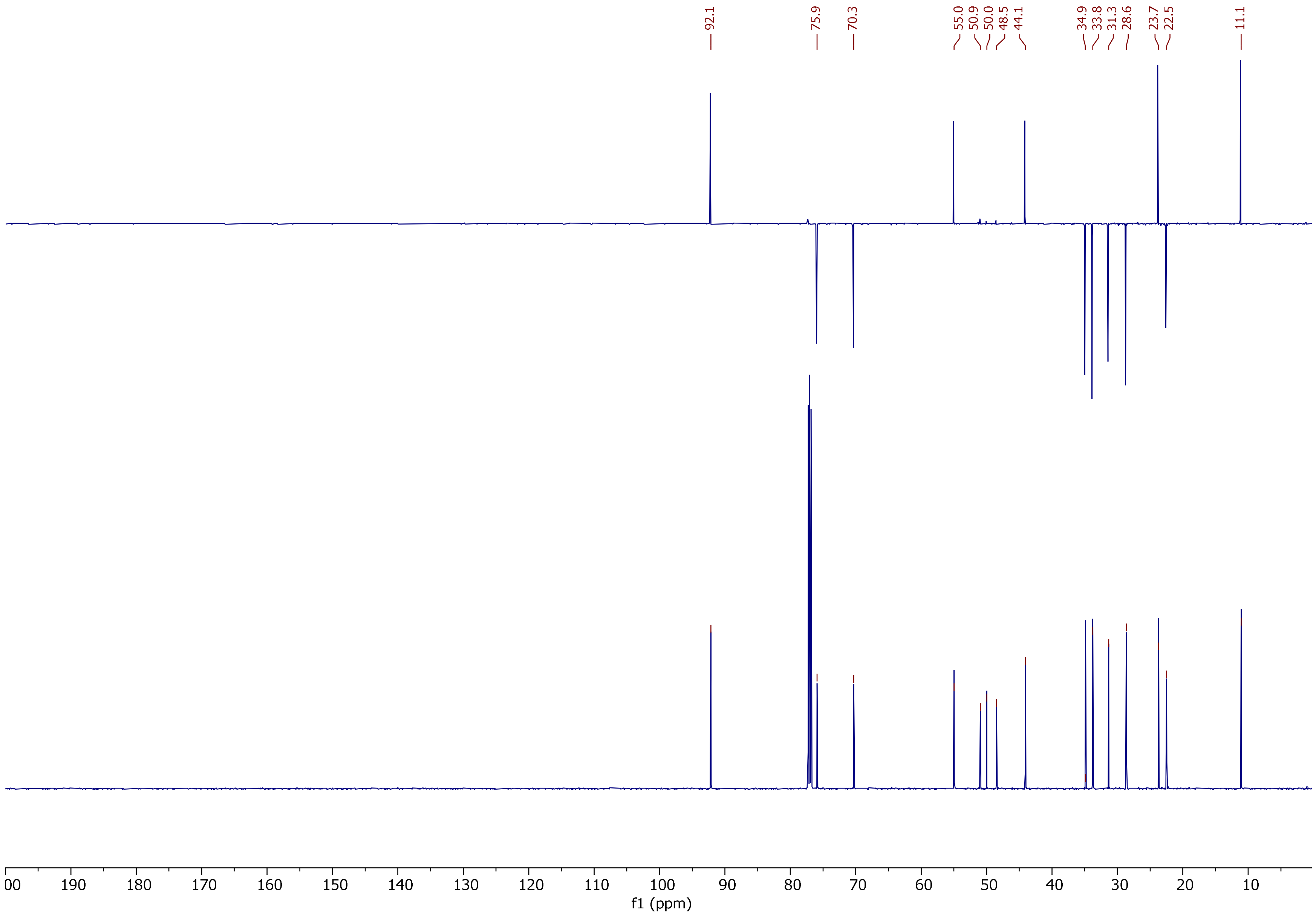
92.1
75.9
70.3
55.0
50.9
50.0
48.5
44.1
34.9
33.8
31.3
28.6
23.7
22.5
11.1
00
190
180
170
160
150
140
130
120
110
100
90
80
70
60
50
40
30
20
10
f1 (ppm)

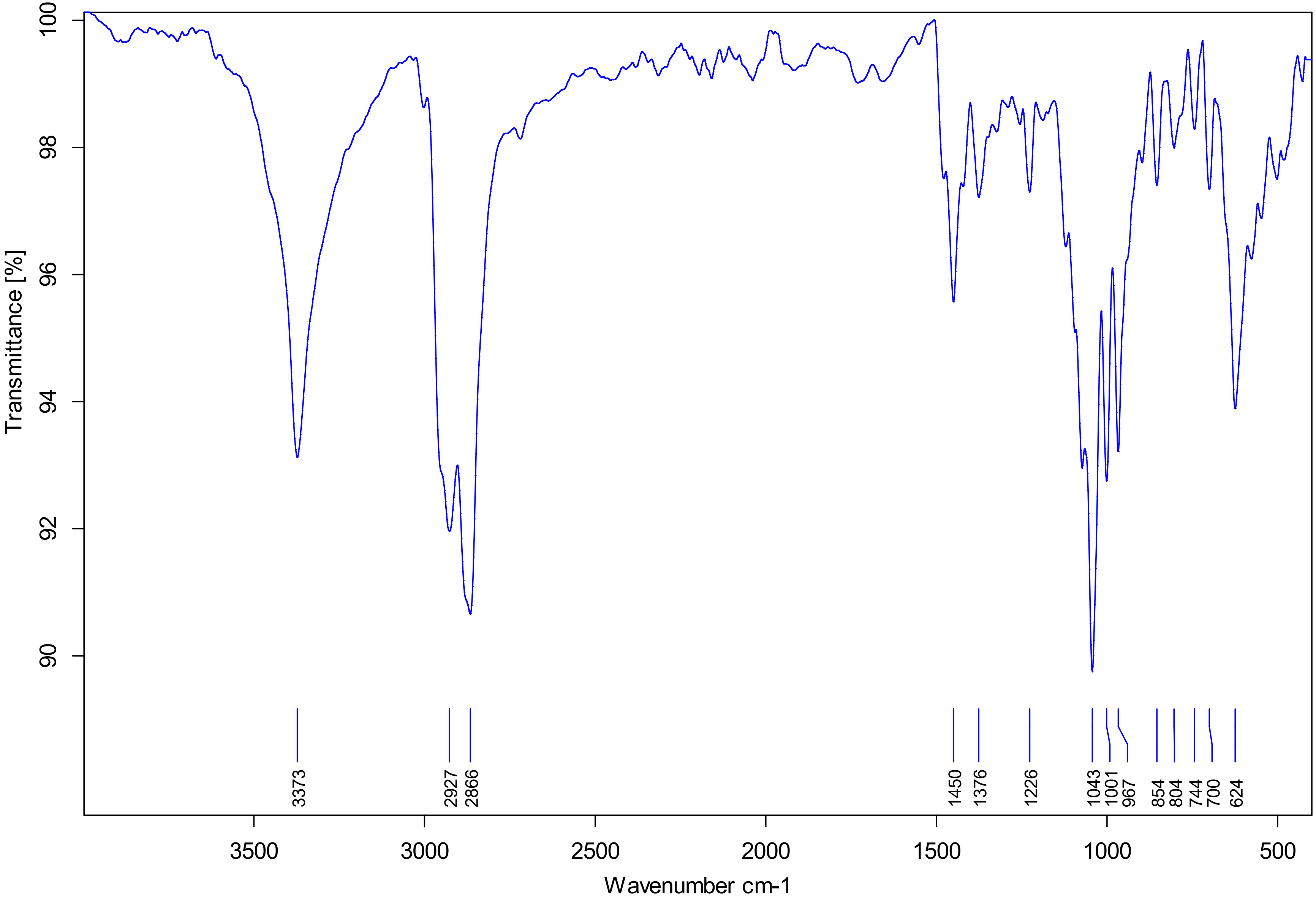

Transmittance [%]
100
98
96
94
92
90
3373
2927
2866
1450
1376
1226
1043
1001
967
854
804
744
700
624
3500
3000
2500
2000
1500
1000
500
Wavenumber cm-1

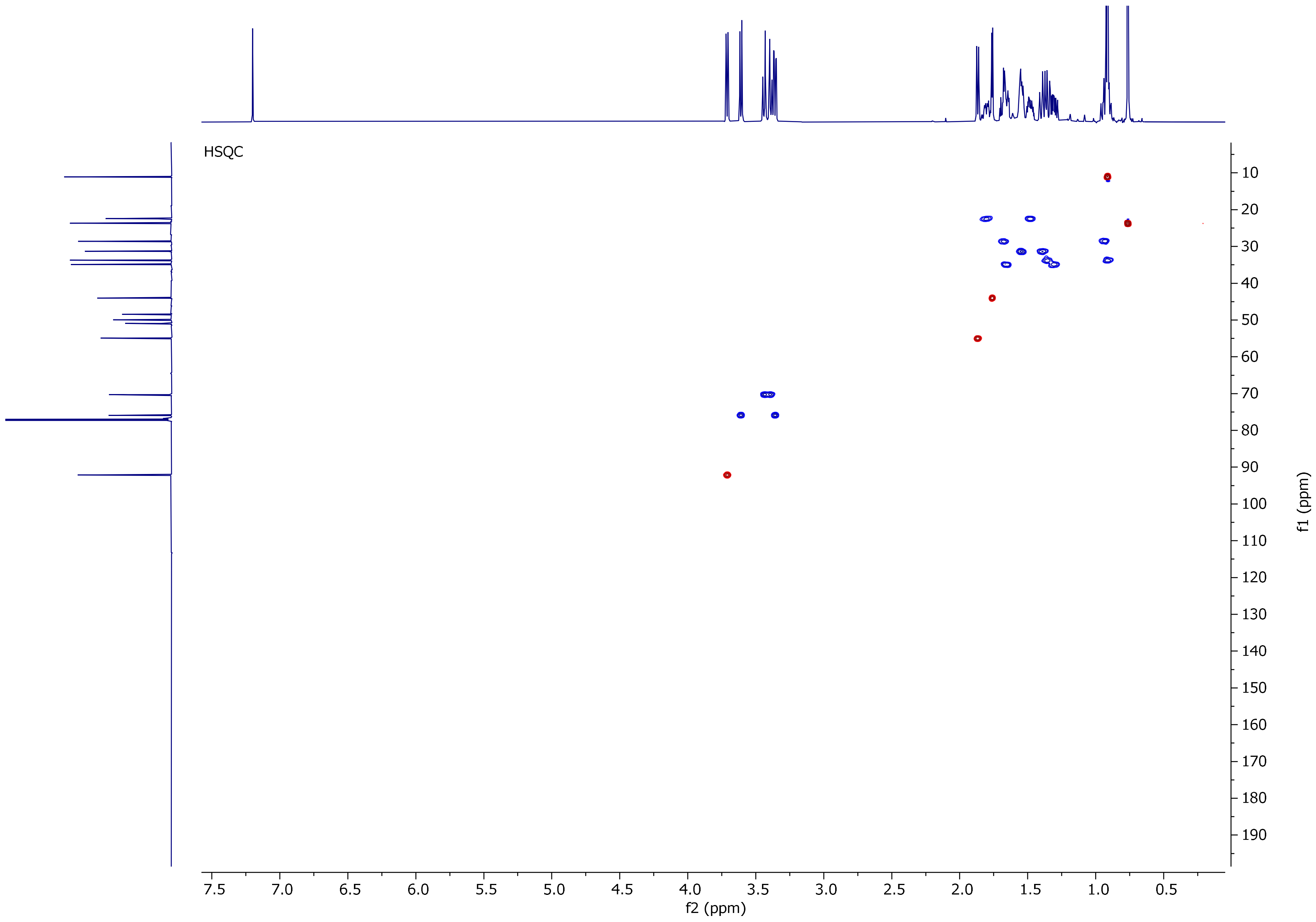

HSQC
f2 (ppm)
f1 (ppm)
7.5
7.0
6.5
6.0
5.5
5.0
4.5
4.0
3.5
3.0
2.5
2.0
1.5
1.0
0.5
10
20
30
40
50
60
70
80
90
100
110
120
130
140
150
160
170
180
190

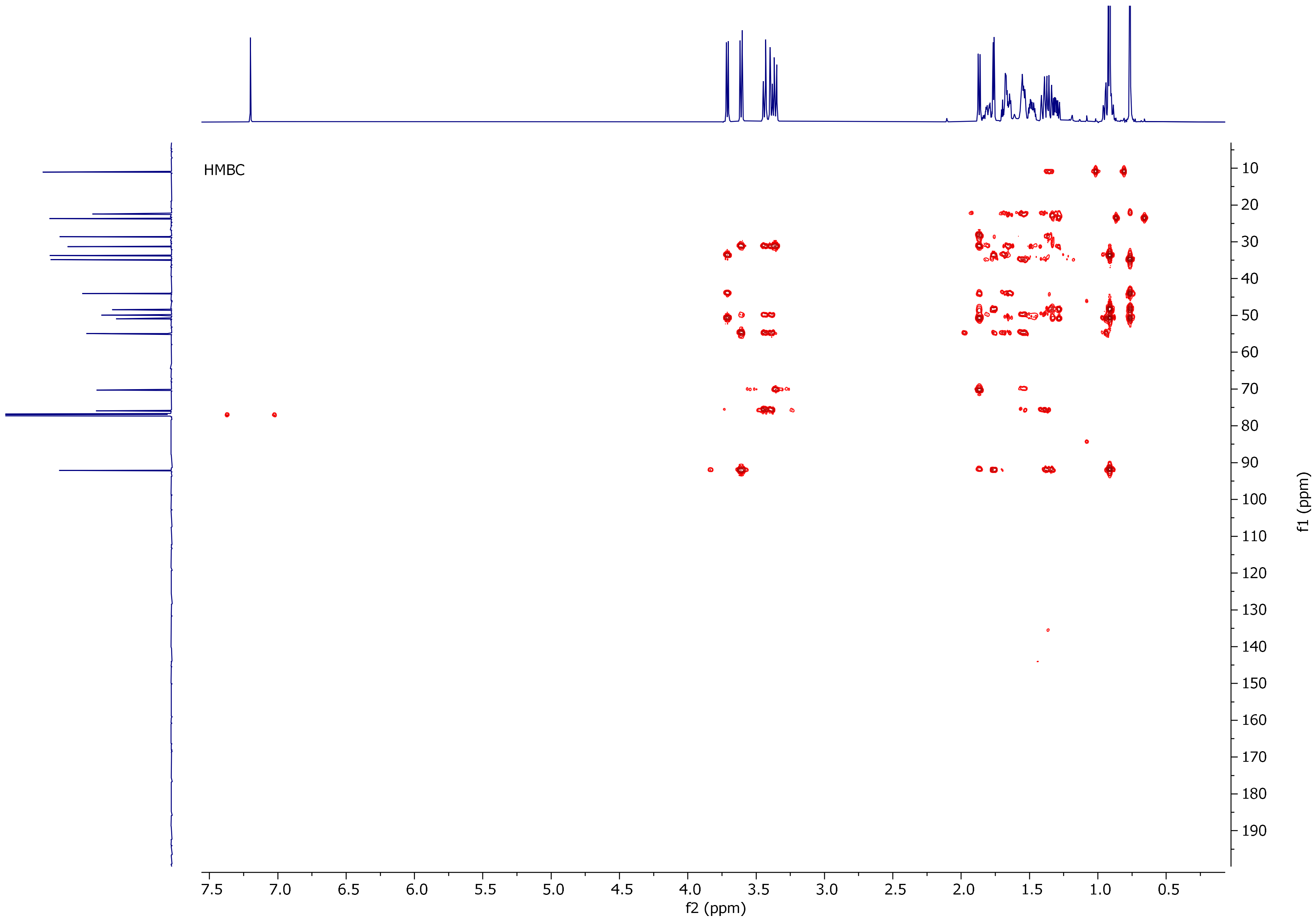
HMBC
7.5
7.0
6.5
6.0
5.5
5.0
4.5
4.0
3.5
3.0
2.5
2.0
1.5
1.0
0.5
f2 (ppm)
10
20
30
40
50
60
70
80
90
100
110
120
130
140
150
160
170
180
190
f1 (ppm)

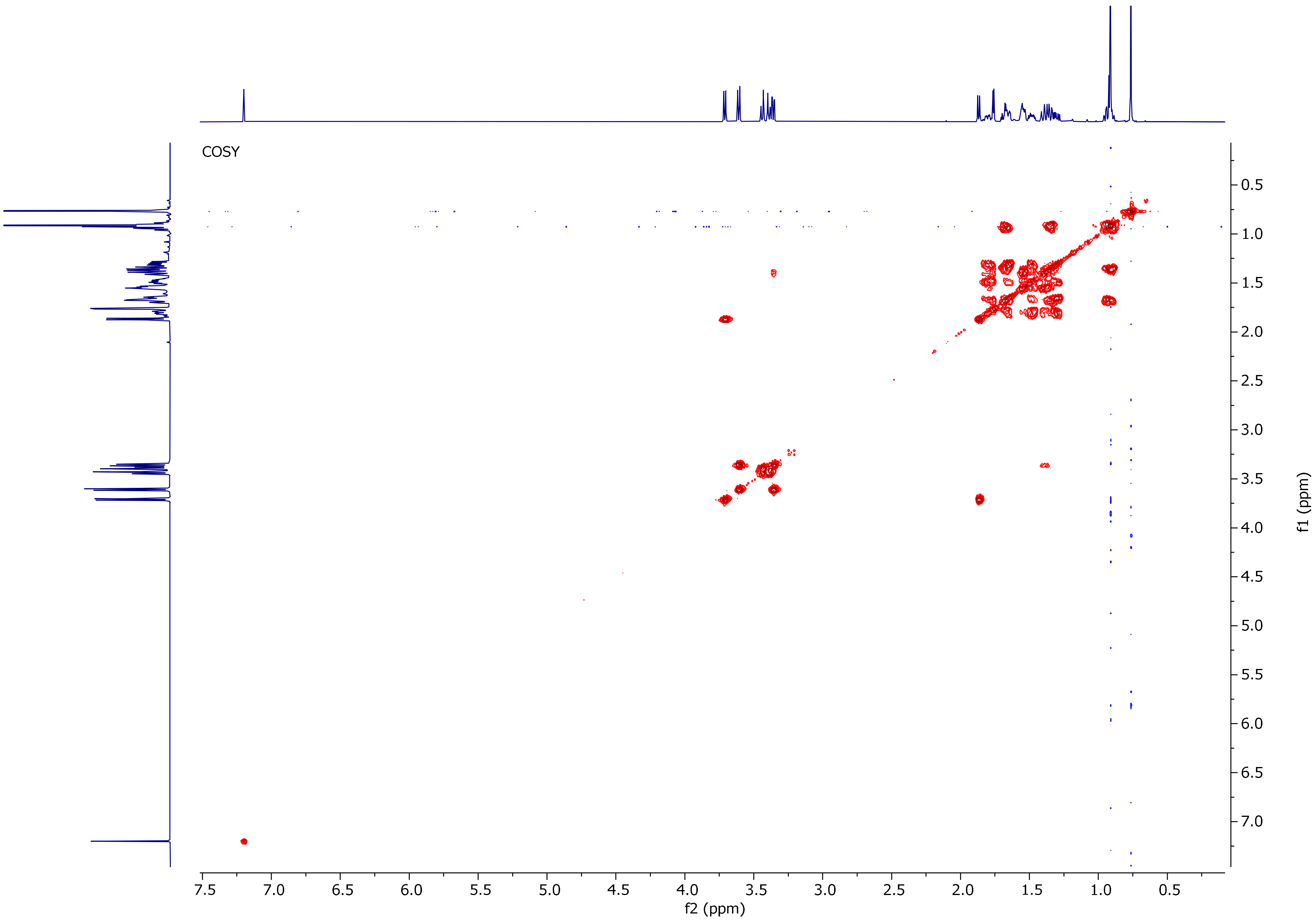

COSY
f2 (ppm)
f1 (ppm)

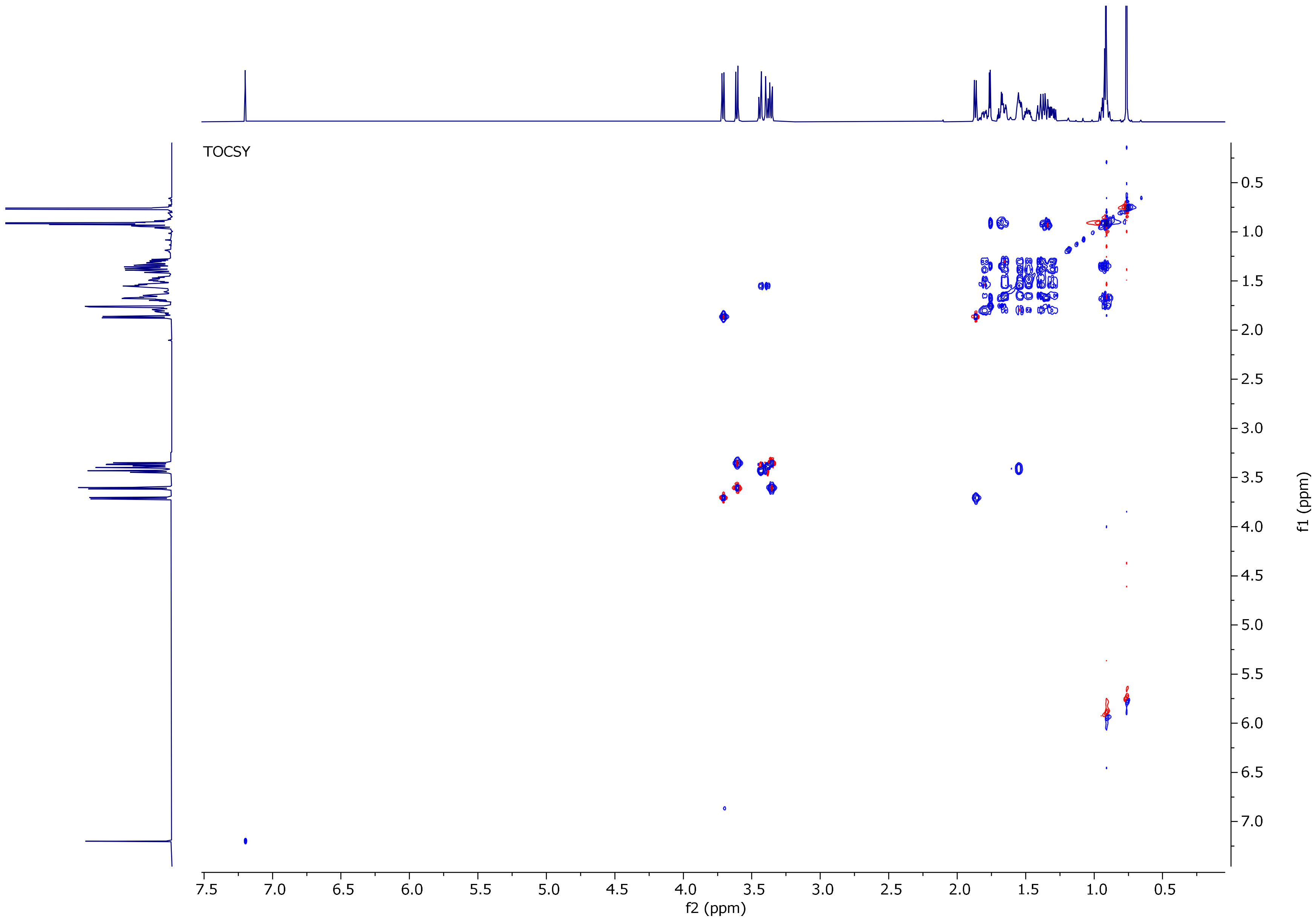
TOCSY
f2 (ppm)
f1 (ppm)

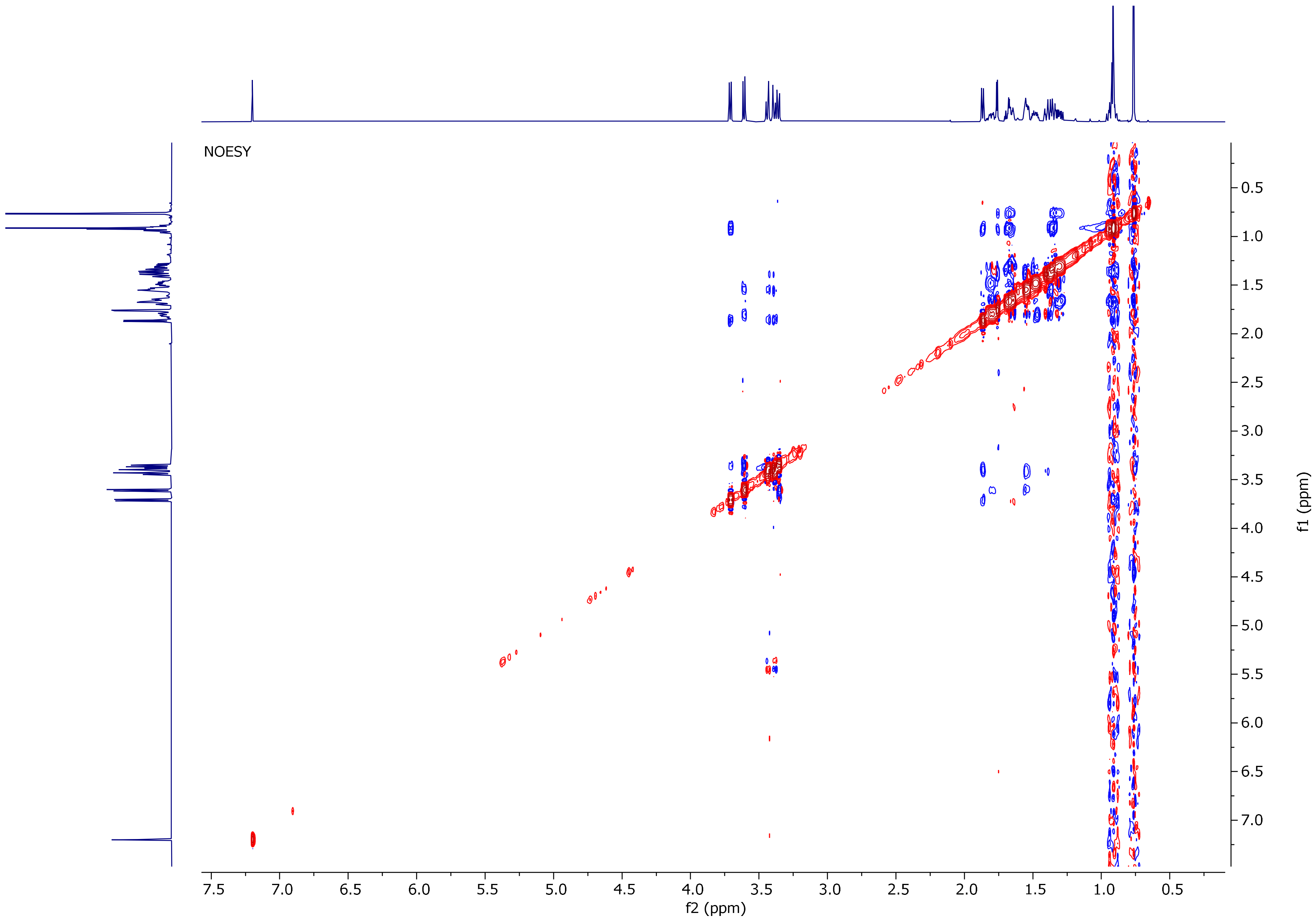
NOESY
7.5
7.0
6.5
6.0
5.5
5.0
4.5
4.0
3.5
3.0
2.5
2.0
1.5
1.0
0.5
f2 (ppm)
f1 (ppm)

^{1}H NMR, DEPT 135, ^{13}C NMR, IR, HSQC, HMBC, COSY, TOCSY and NOESY of **16**

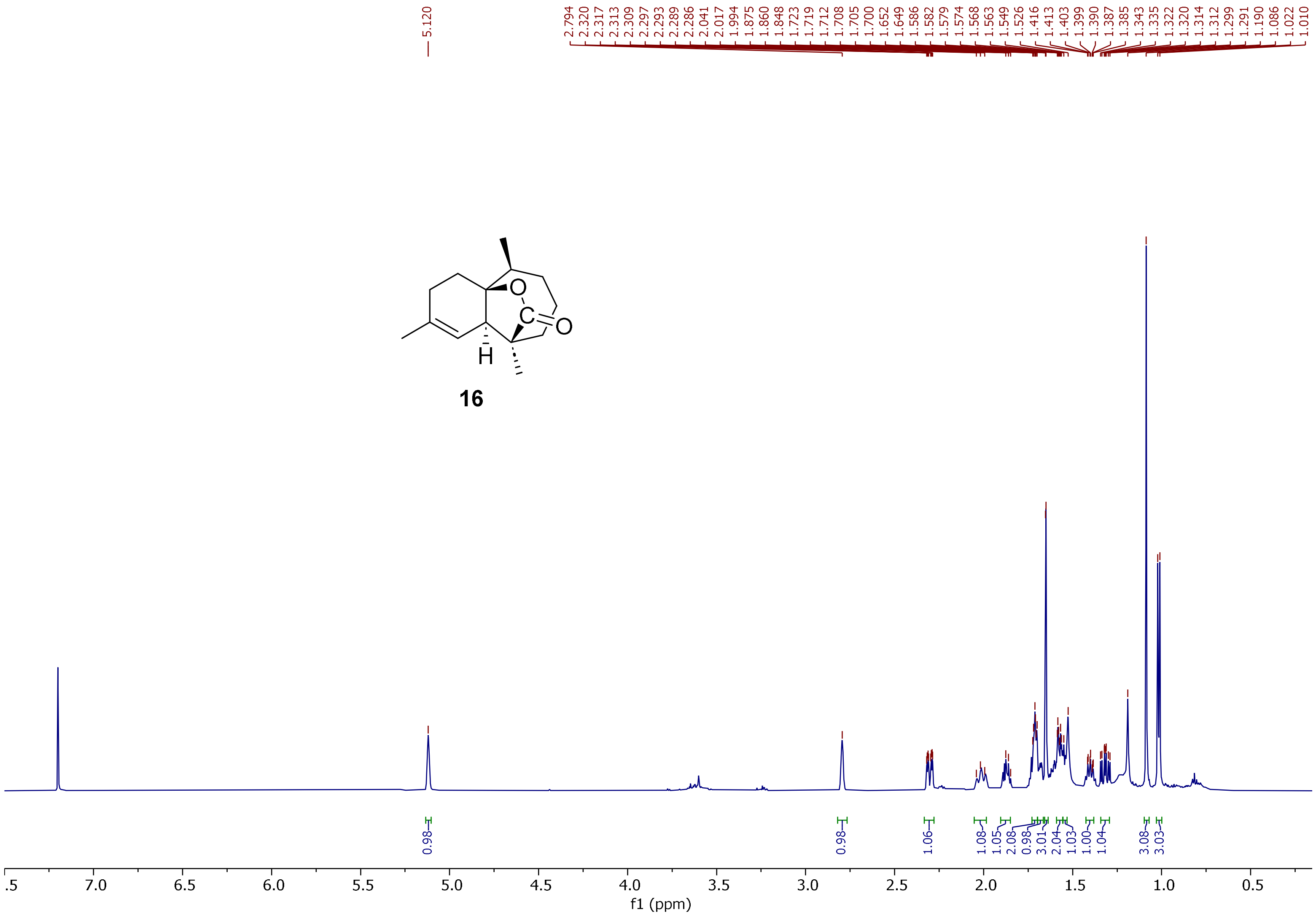

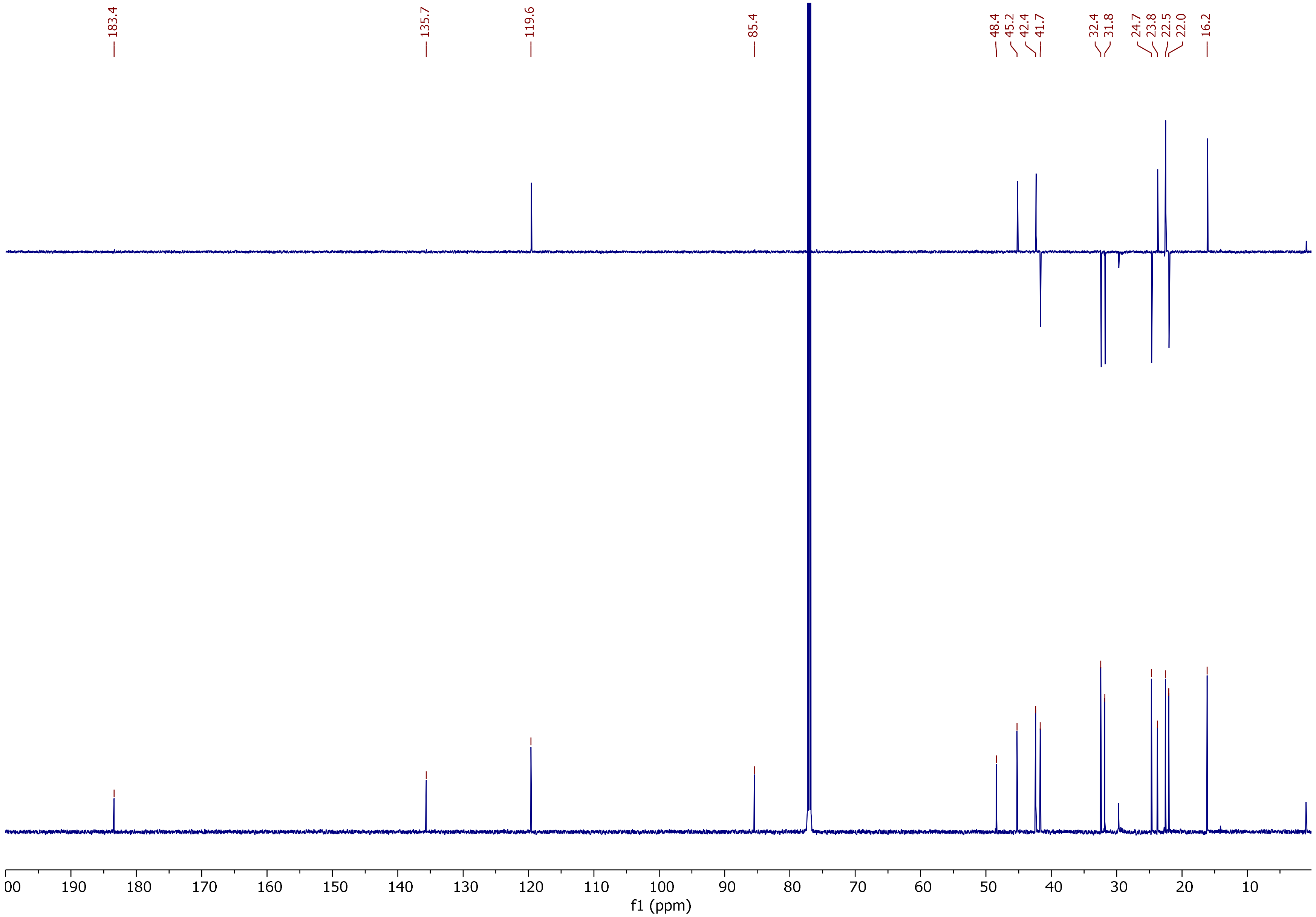
183.4
135.7
119.6
85.4
48.4
45.2
42.4
41.7
32.4
31.8
24.7
23.8
22.5
22.0
16.2
00
190
180
170
160
150
140
130
120
110
100
90
80
70
60
50
40
30
20
10
f1 (ppm)

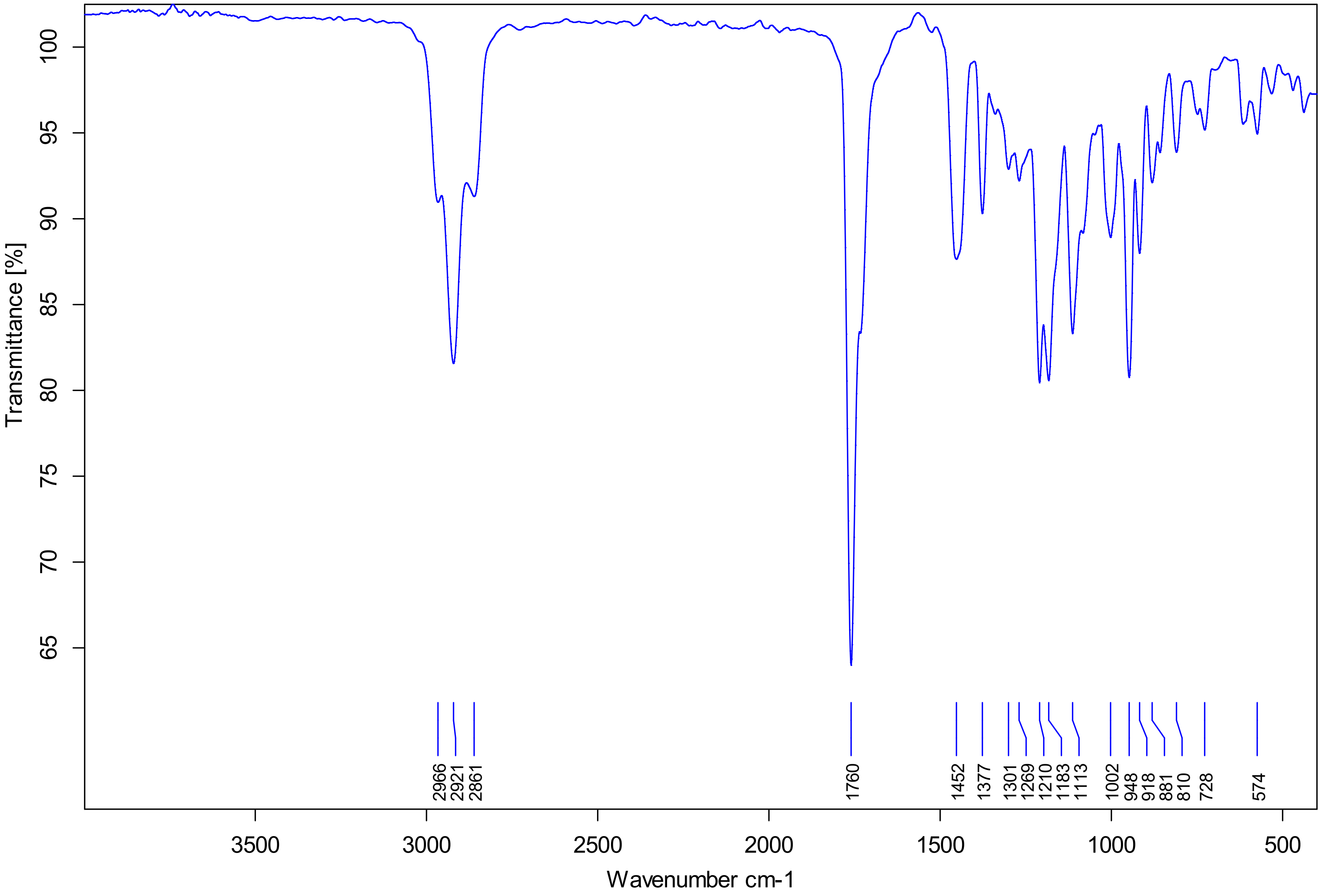

Transmittance [%]
100
95
90
85
80
75
70
65
2966
2921
2861
1760
1452
1377
1301
1269
1210
1183
1113
1002
948
918
881
810
728
574
3500
3000
2500
2000
1500
1000
500
Wavenumber cm-1

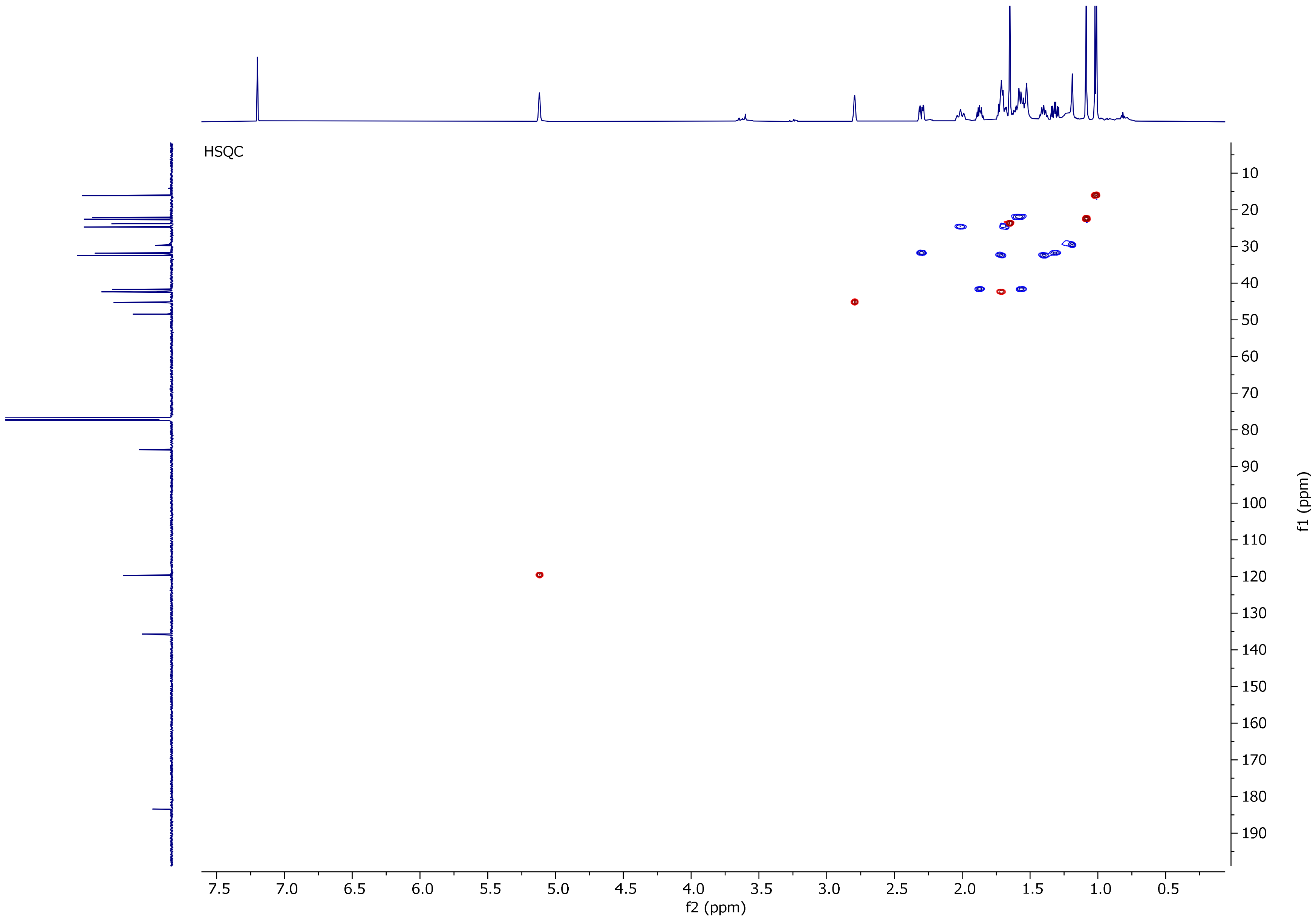
HSQC
f2 (ppm)
f1 (ppm)
7.5
7.0
6.5
6.0
5.5
5.0
4.5
4.0
3.5
3.0
2.5
2.0
1.5
1.0
0.5
10
20
30
40
50
60
70
80
90
100
110
120
130
140
150
160
170
180
190

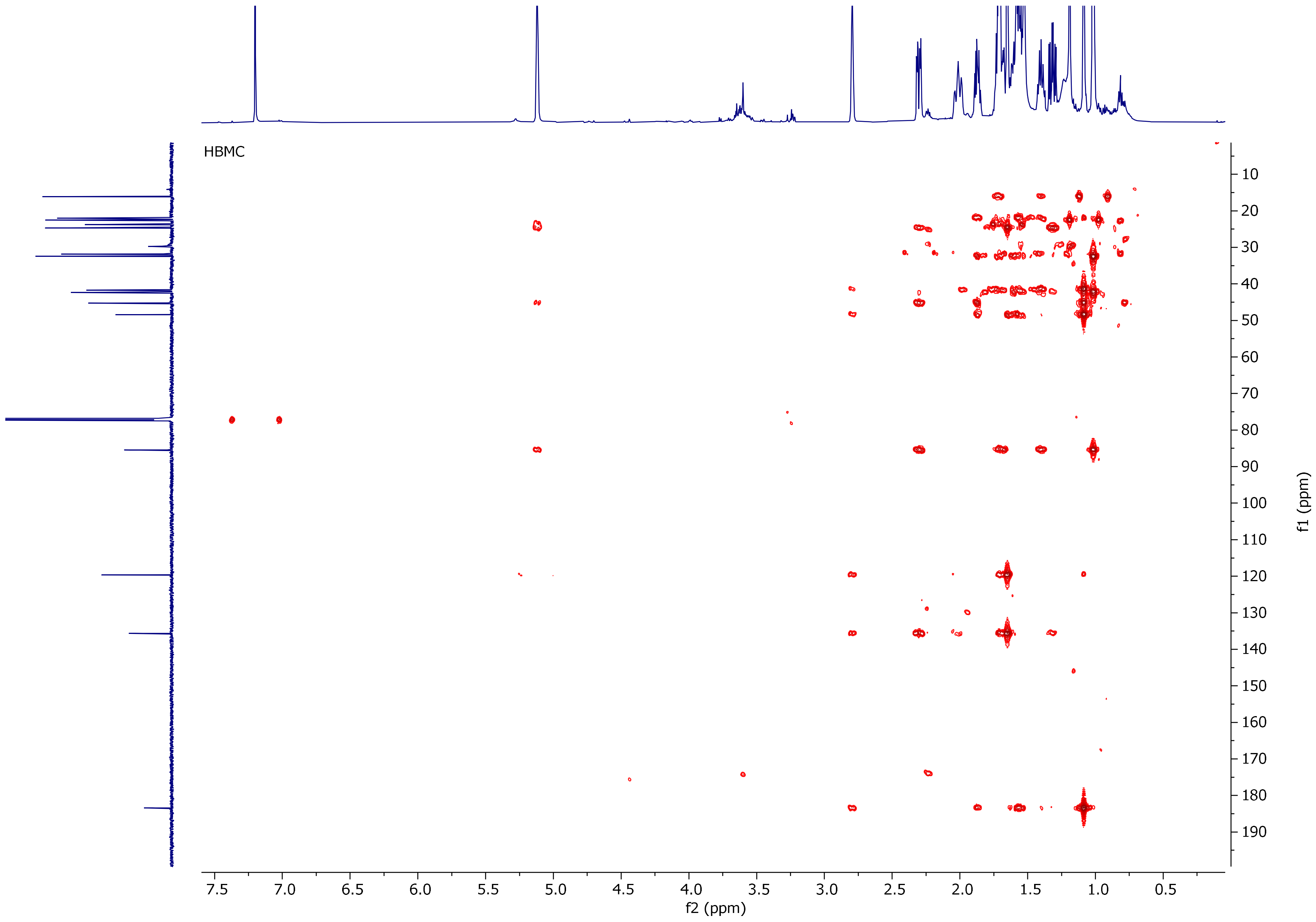
HBMC
7.5
7.0
6.5
6.0
5.5
5.0
4.5
4.0
3.5
3.0
2.5
2.0
1.5
1.0
0.5
f2 (ppm)
10
20
30
40
50
60
70
80
90
100
110
120
130
140
150
160
170
180
190
f1 (ppm)

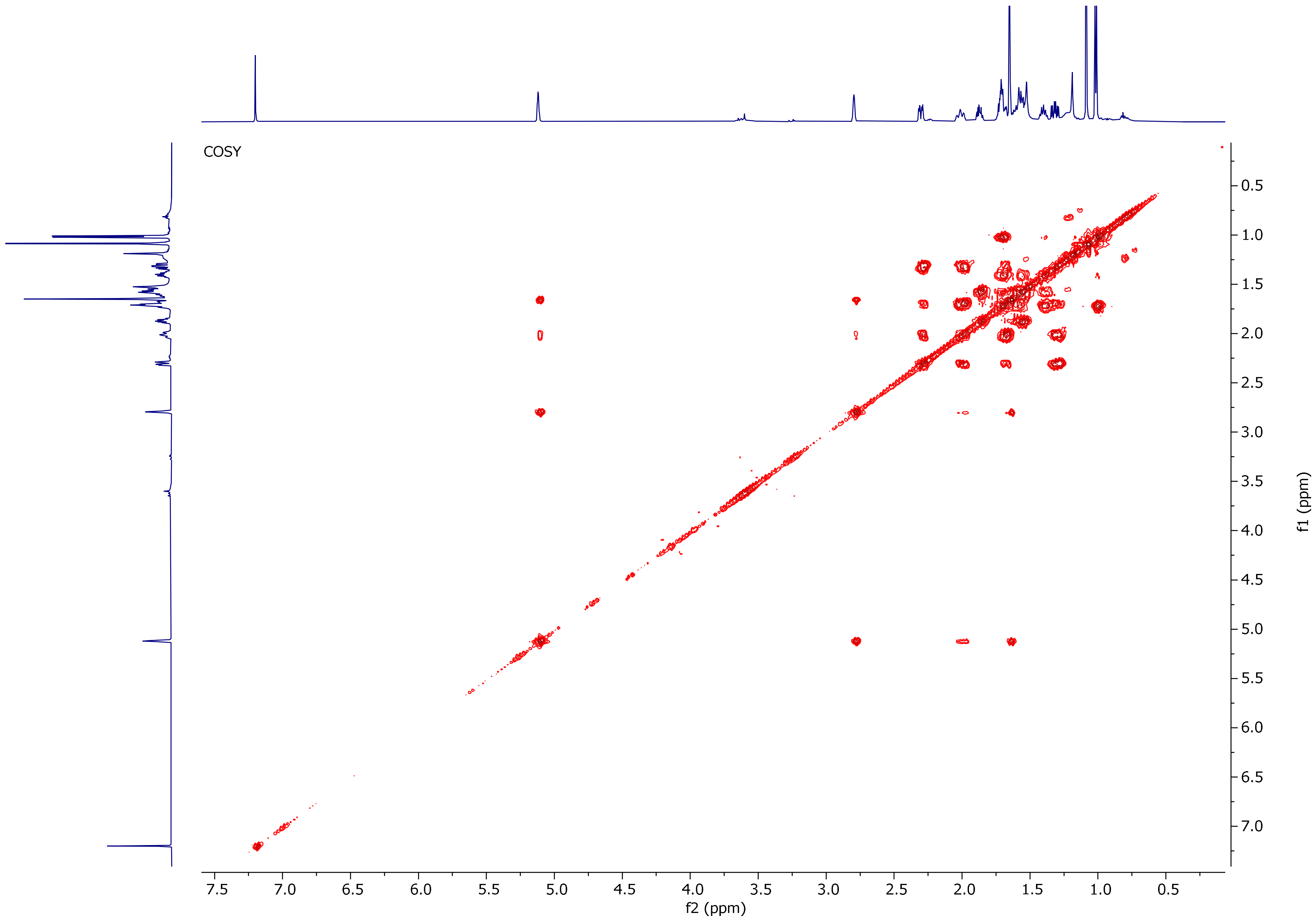
COSY
f2 (ppm)
f1 (ppm)
7.5
7.0
6.5
6.0
5.5
5.0
4.5
4.0
3.5
3.0
2.5
2.0
1.5
1.0
0.5

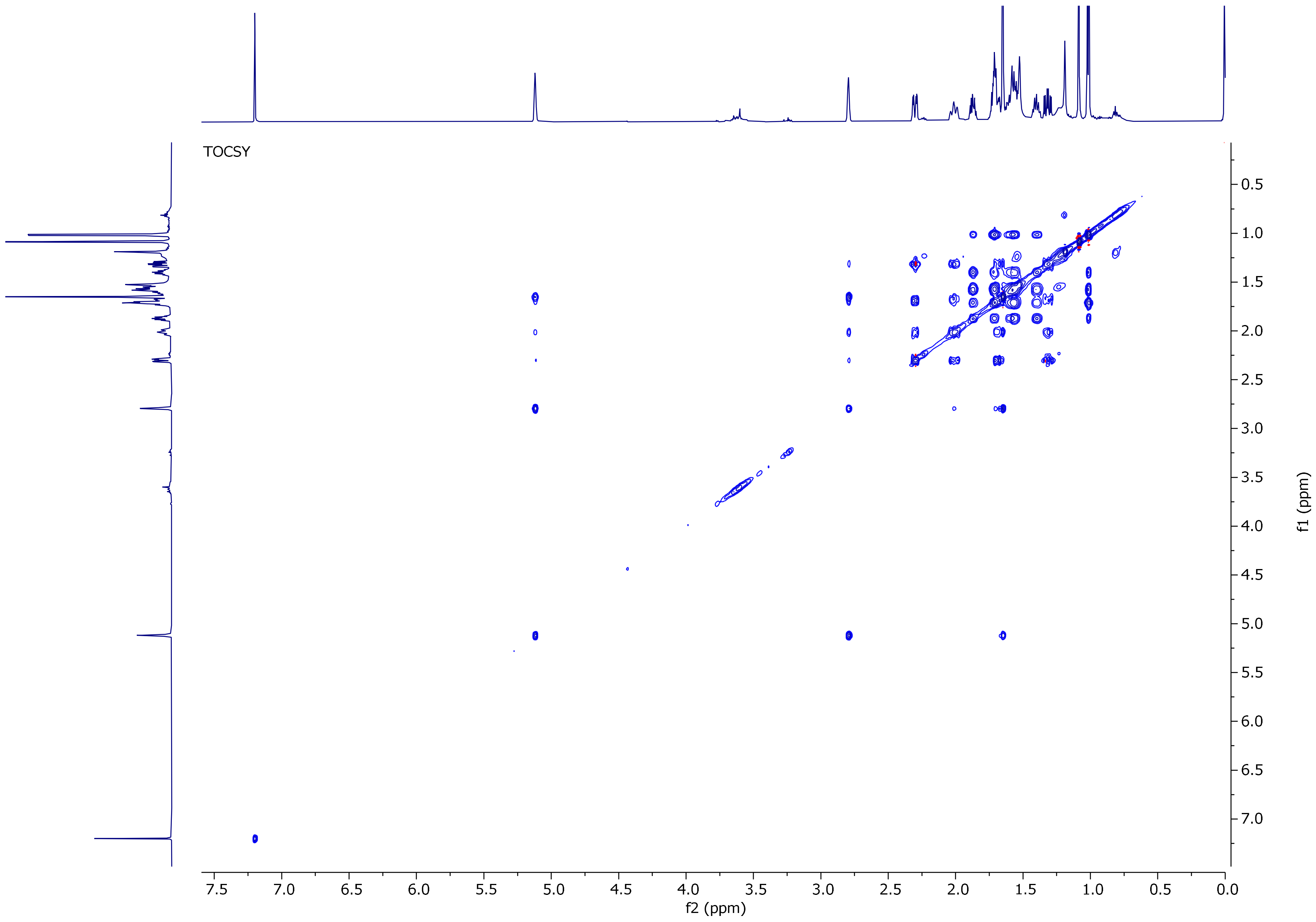
TOCSY
f2 (ppm)
f1 (ppm)
7.5
7.0
6.5
6.0
5.5
5.0
4.5
4.0
3.5
3.0
2.5
2.0
1.5
1.0
0.5
0.0

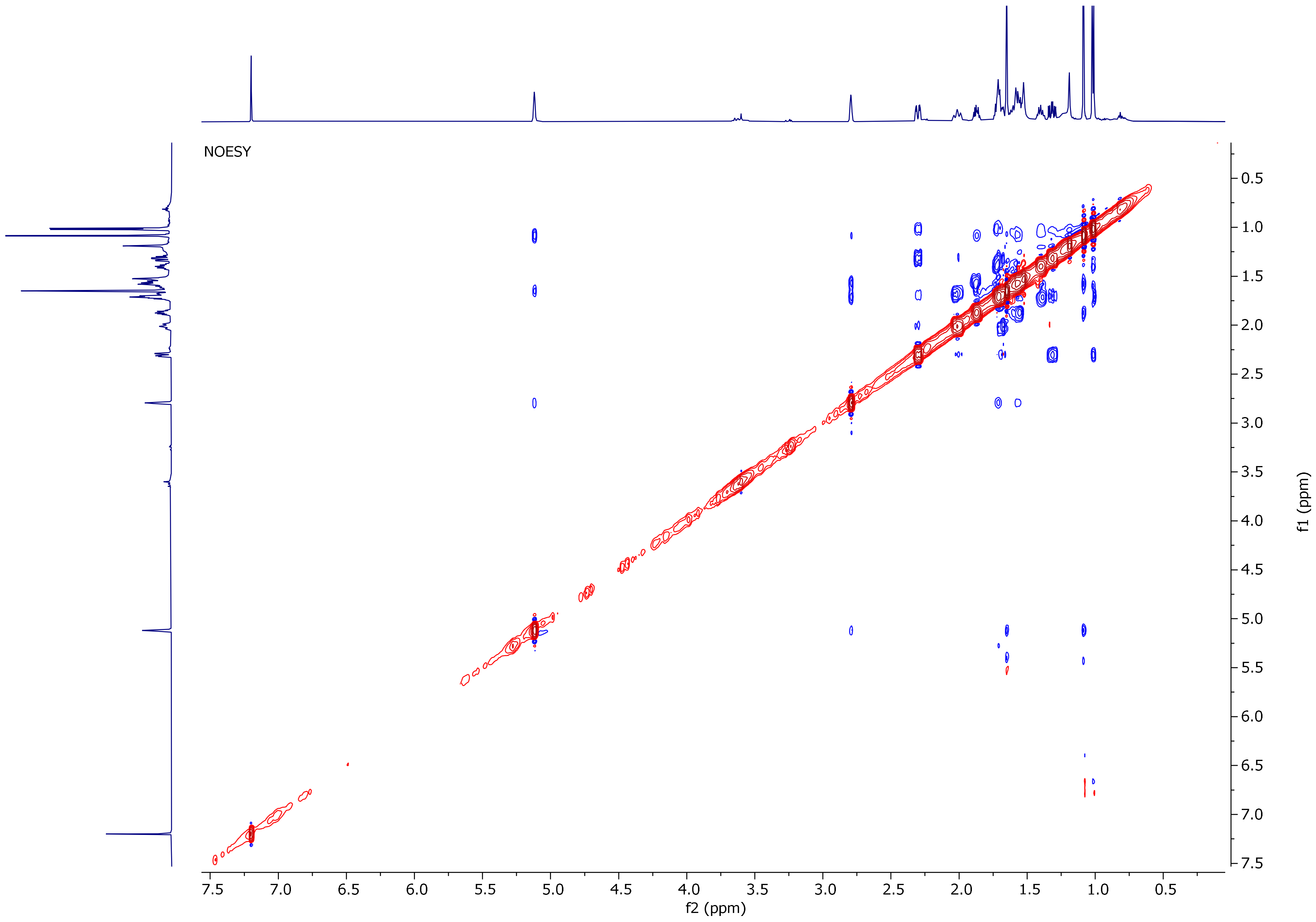
NOESY
f2 (ppm)
f1 (ppm)
7.5
7.0
6.5
6.0
5.5
5.0
4.5
4.0
3.5
3.0
2.5
2.0
1.5
1.0
0.5

^{1}H NMR, DEPT 135, ^{13}C NMR, IR, HSQC, HMBC, COSY, TOCSY and NOESY of **17**

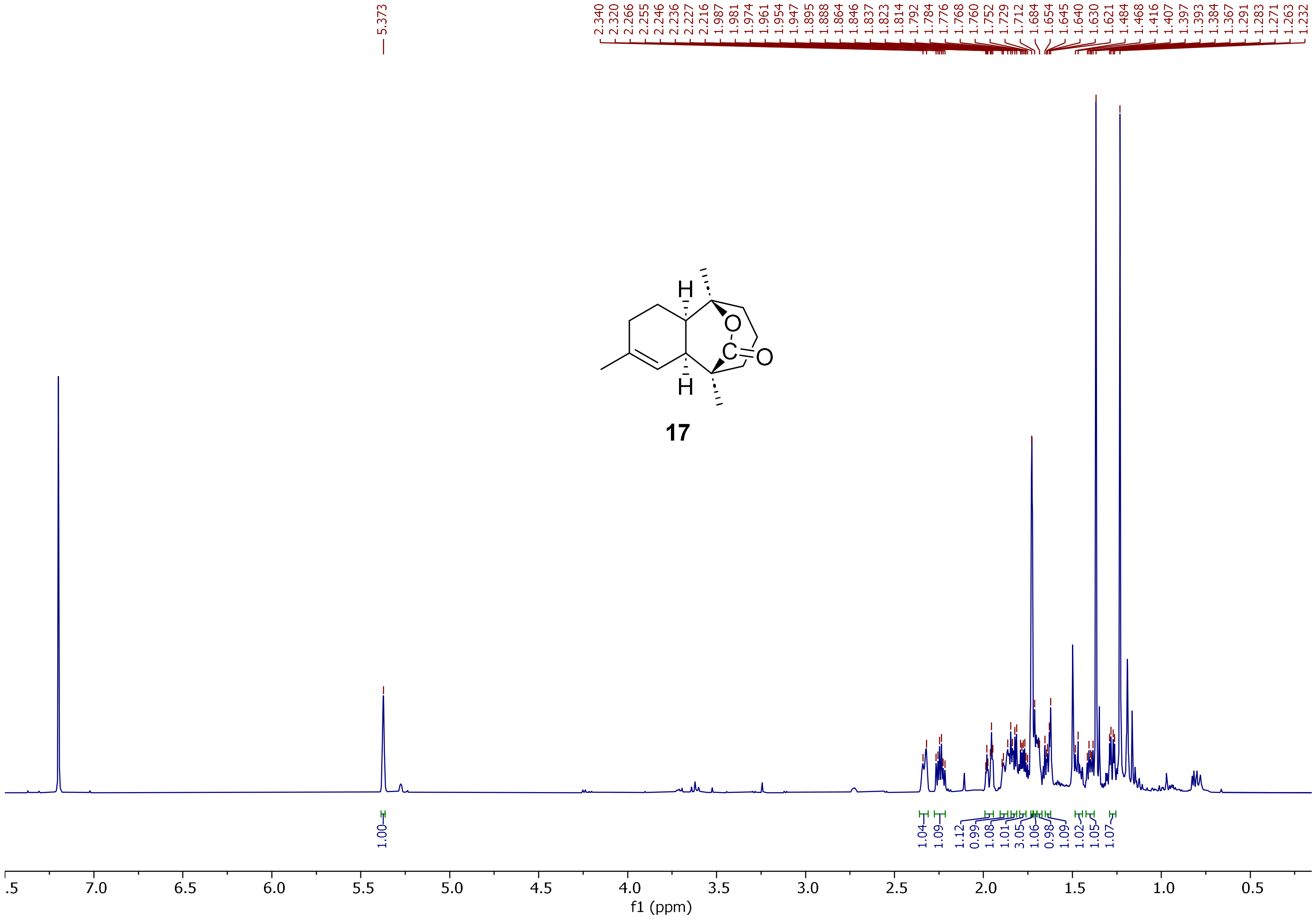

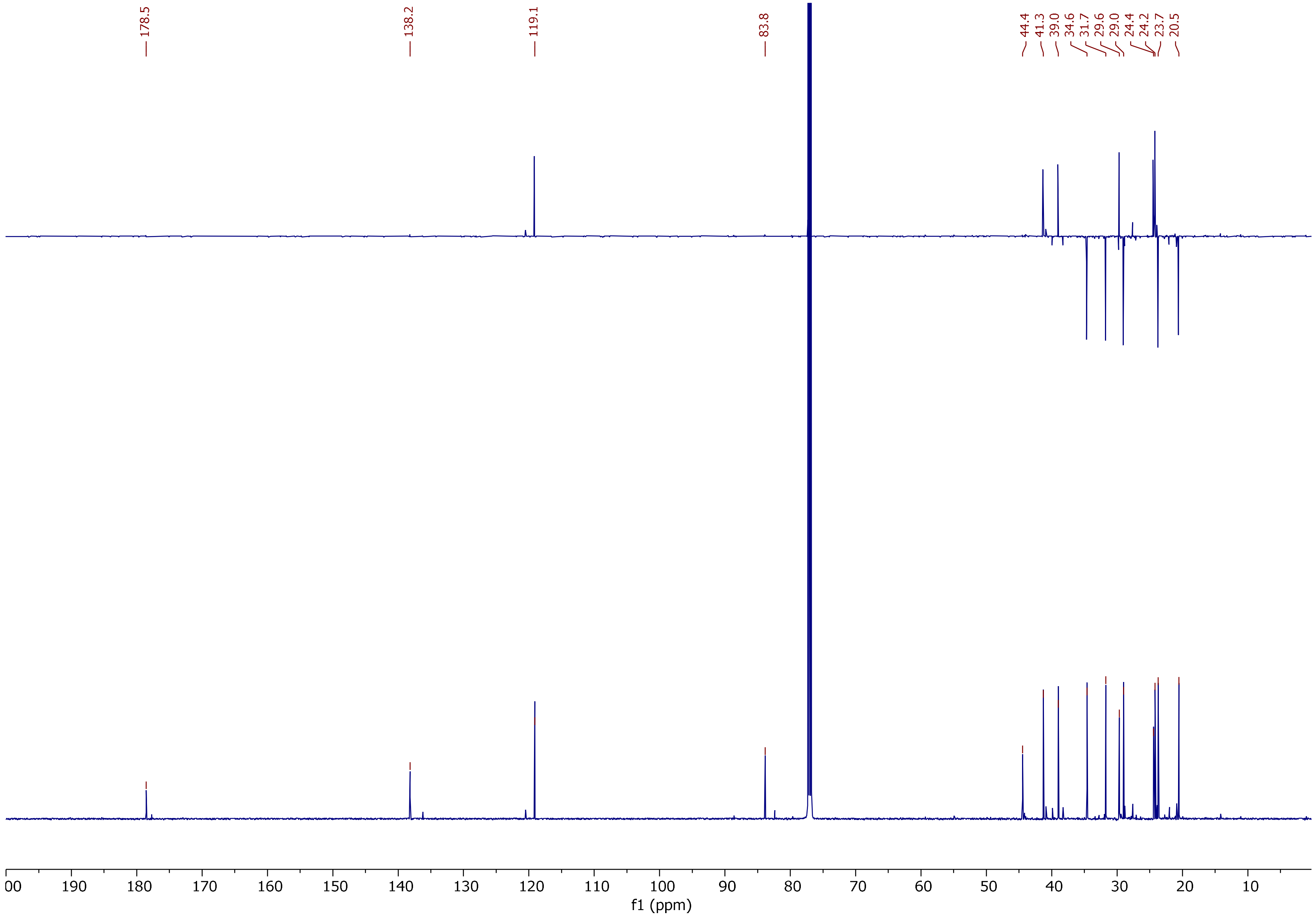
178.5
138.2
119.1
83.8
44.4
41.3
39.0
34.6
31.7
29.6
29.0
24.4
24.2
23.7
20.5
00
190
180
170
160
150
140
130
120
110
100
90
80
70
60
50
40
30
20
10
f1 (ppm)

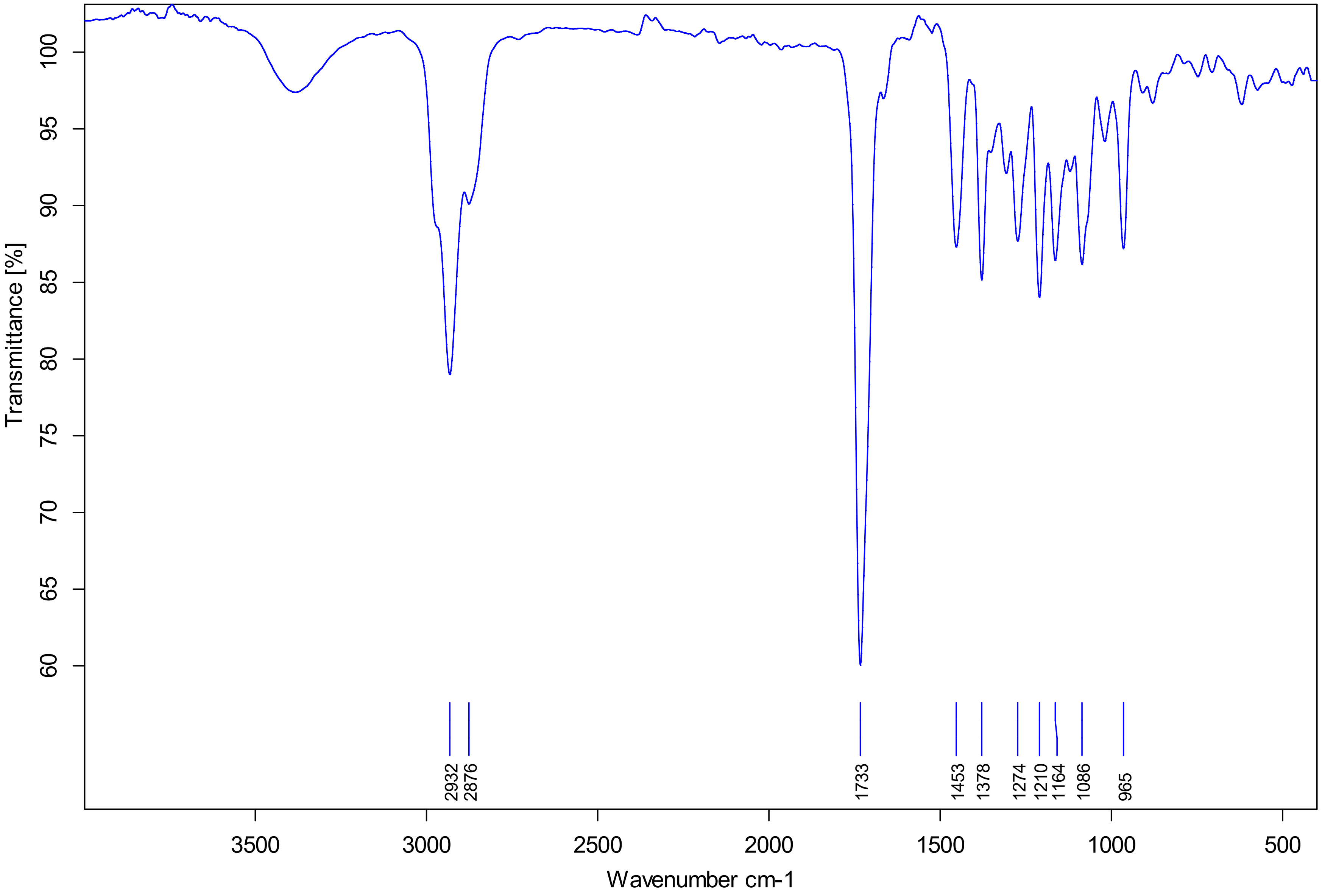

Transmittance [%]
100
95
90
85
80
75
70
65
60
2932
2876
1733
1453
1378
1274
1210
1164
1086
965
3500
3000
2500
2000
1500
1000
500
Wavenumber cm-1

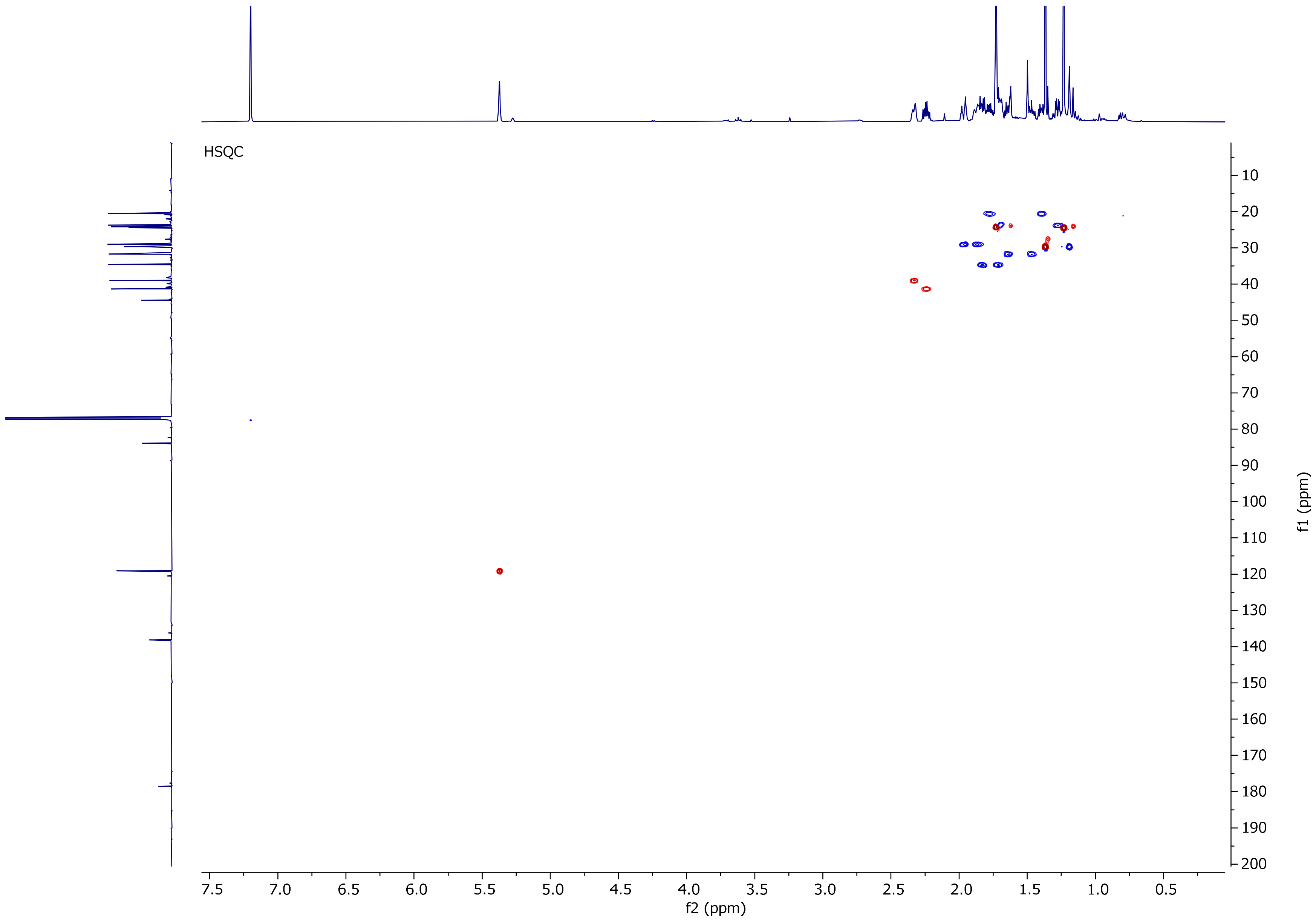
HSQC
7.5
7.0
6.5
6.0
5.5
5.0
4.5
4.0
3.5
3.0
2.5
2.0
1.5
1.0
0.5
f2 (ppm)
10
20
30
40
50
60
70
80
90
100
110
120
130
140
150
160
170
180
190
200
f1 (ppm)

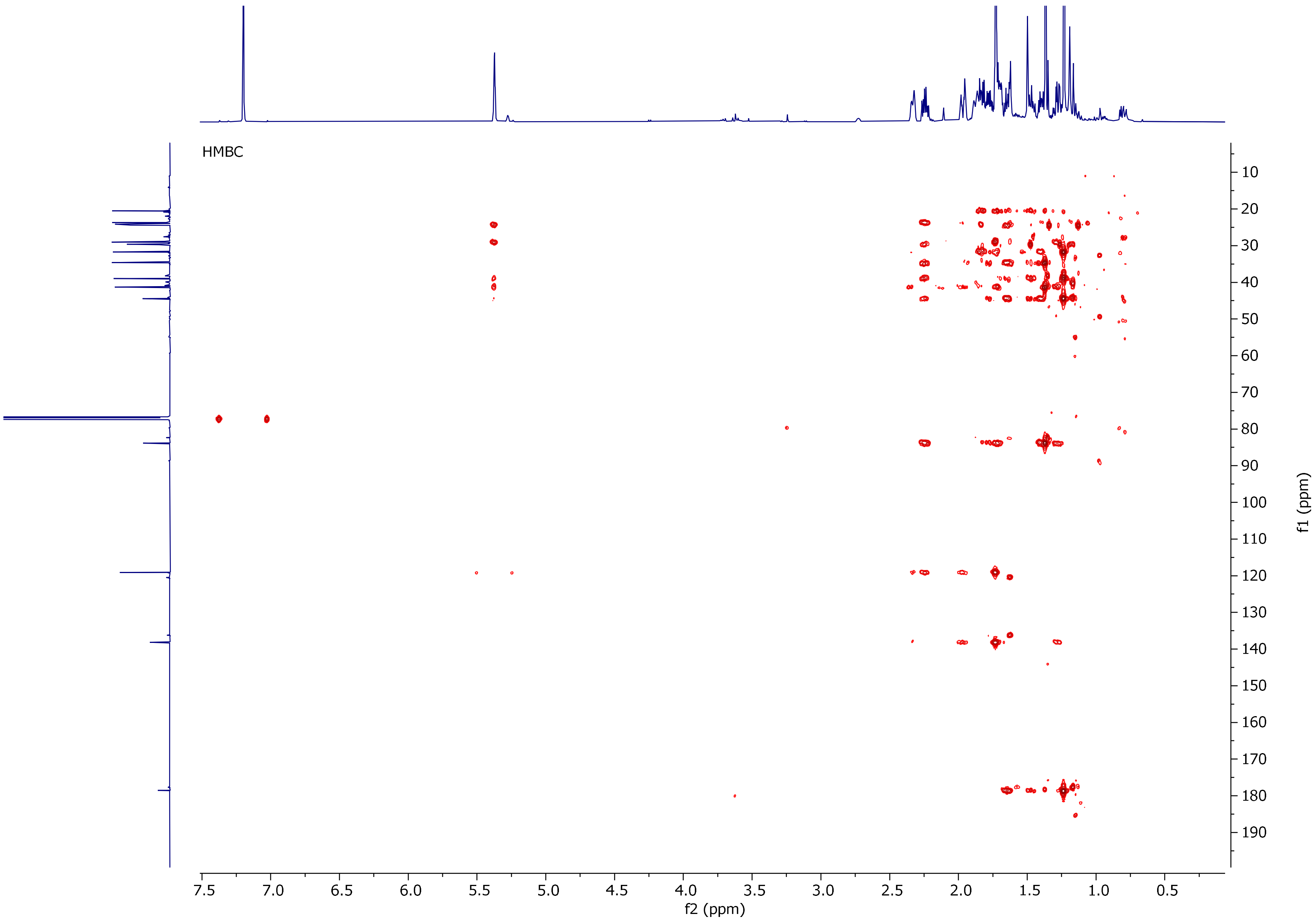
HMBC
7.5
7.0
6.5
6.0
5.5
5.0
4.5
4.0
3.5
3.0
2.5
2.0
1.5
1.0
0.5
f2 (ppm)
10
20
30
40
50
60
70
80
90
100
110
120
130
140
150
160
170
180
190
f1 (ppm)

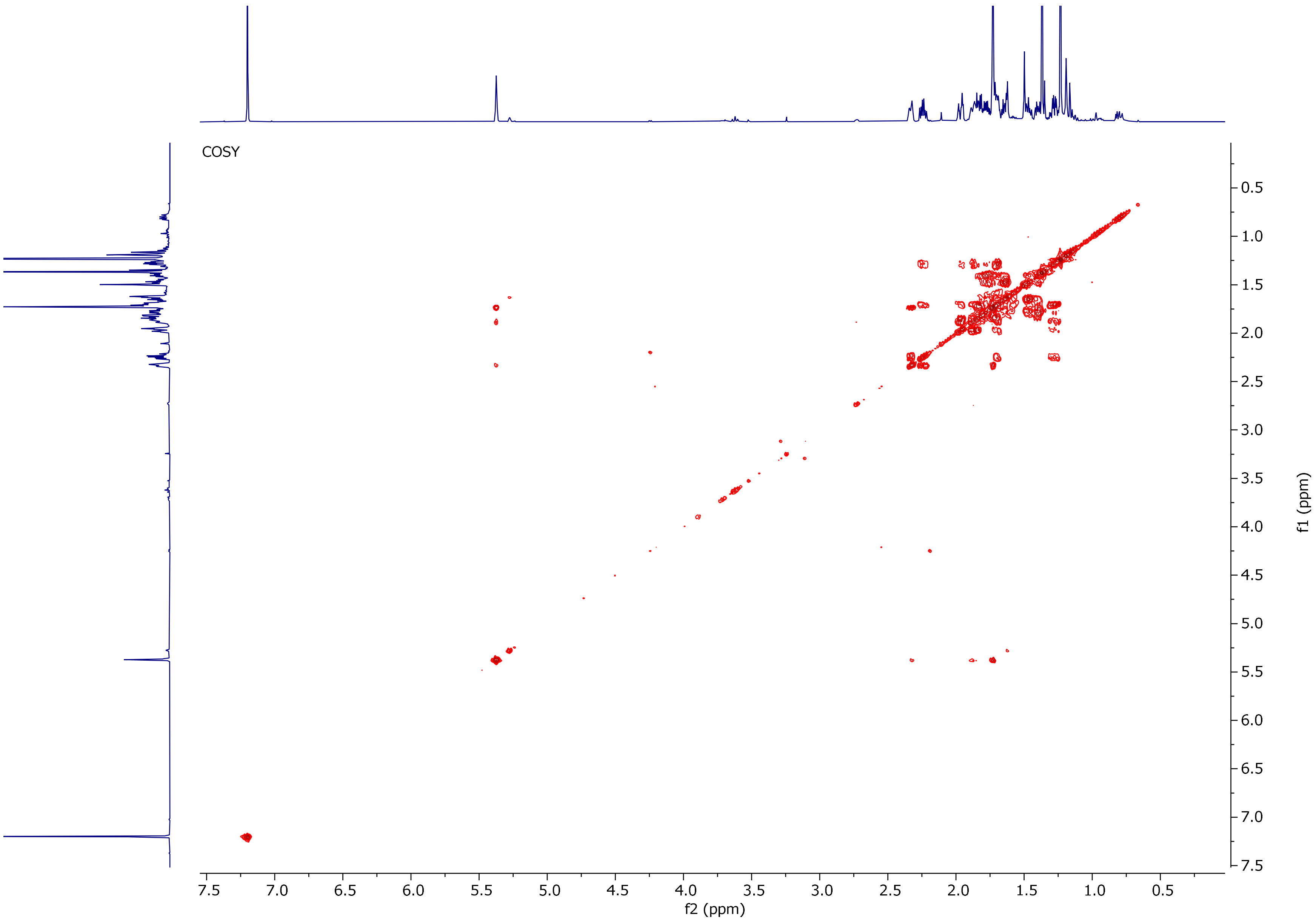
COSY
f2 (ppm)
f1 (ppm)
7.5
7.0
6.5
6.0
5.5
5.0
4.5
4.0
3.5
3.0
2.5
2.0
1.5
1.0
0.5

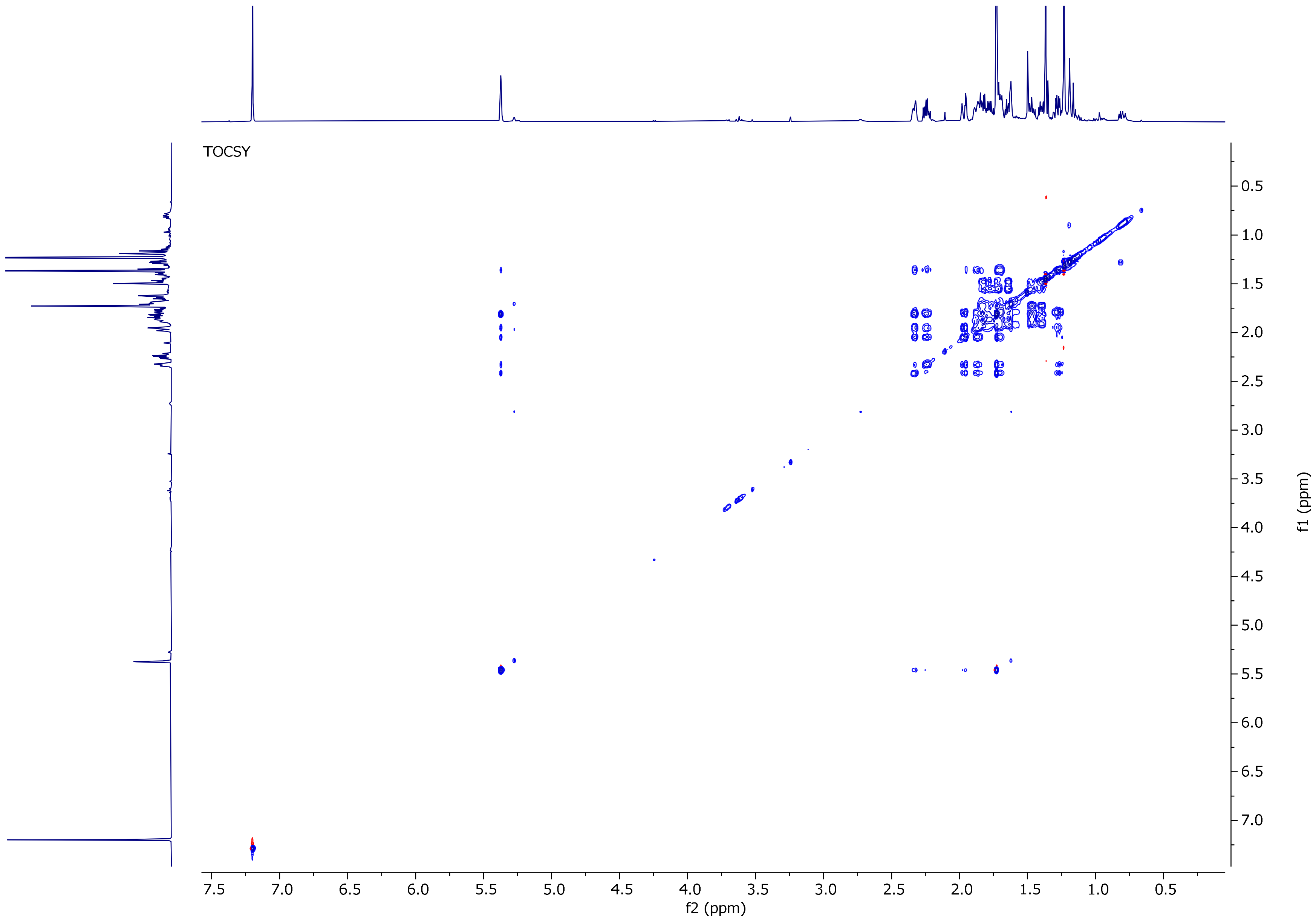

TOCSY
f2 (ppm)
f1 (ppm)
7.5
7.0
6.5
6.0
5.5
5.0
4.5
4.0
3.5
3.0
2.5
2.0
1.5
1.0
0.5

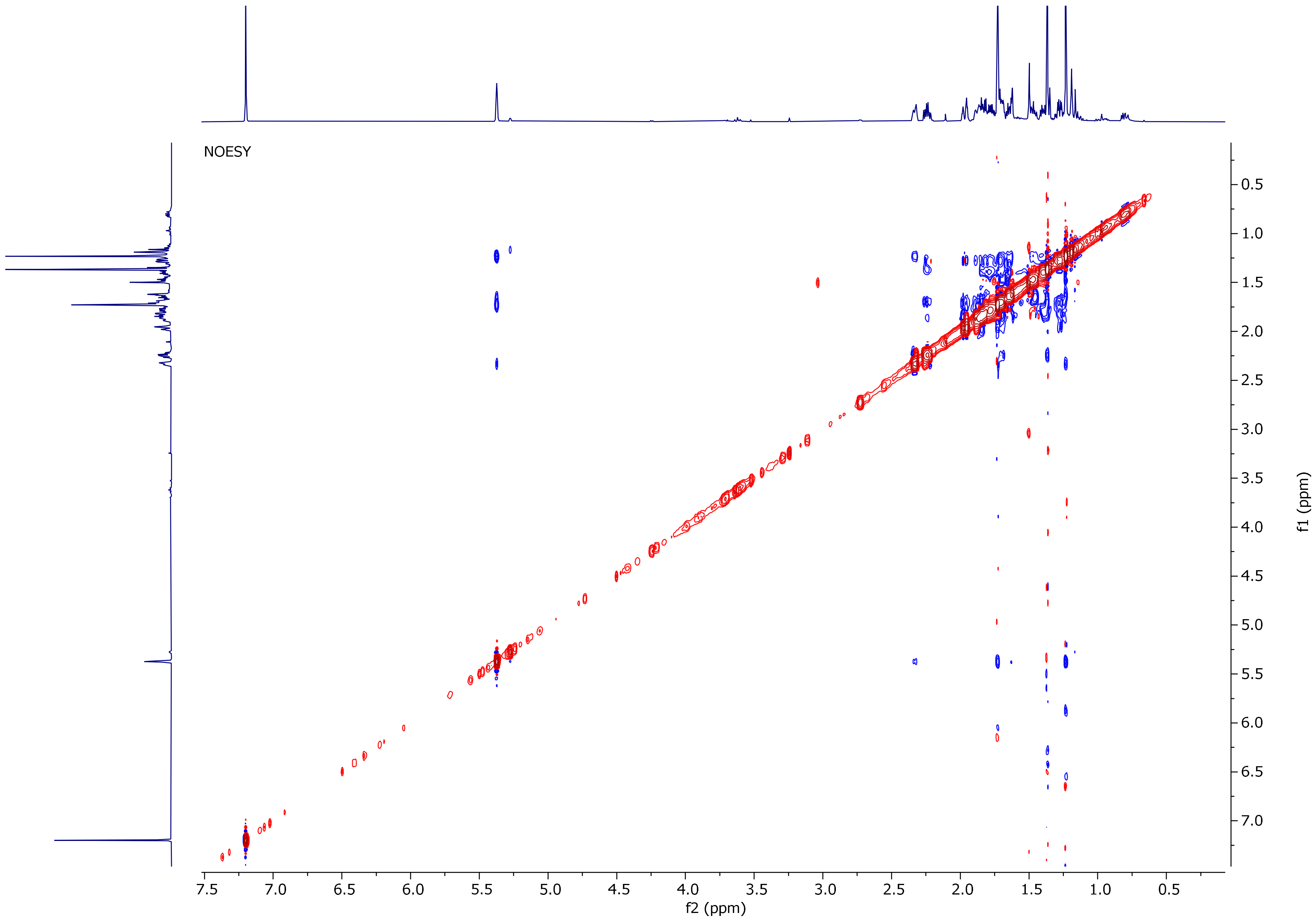

NOESY
7.5
7.0
6.5
6.0
5.5
5.0
4.5
4.0
3.5
3.0
2.5
2.0
1.5
1.0
0.5
f2 (ppm)
f1 (ppm)

^{1}H NMR, DEPT 135, ^{13}C NMR, IR, HSQC, HMBC, COSY, TOCSY and NOESY of **18**

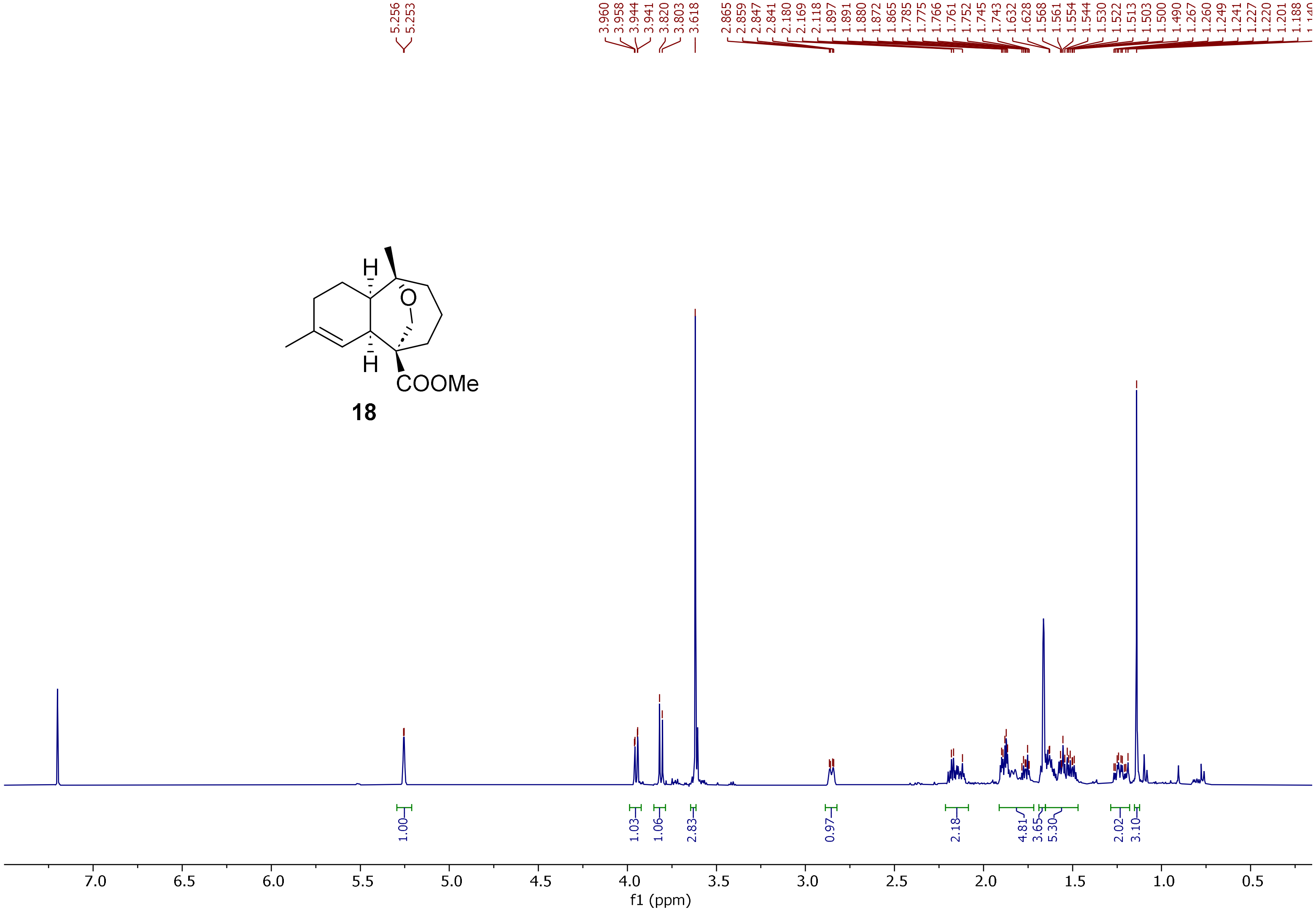

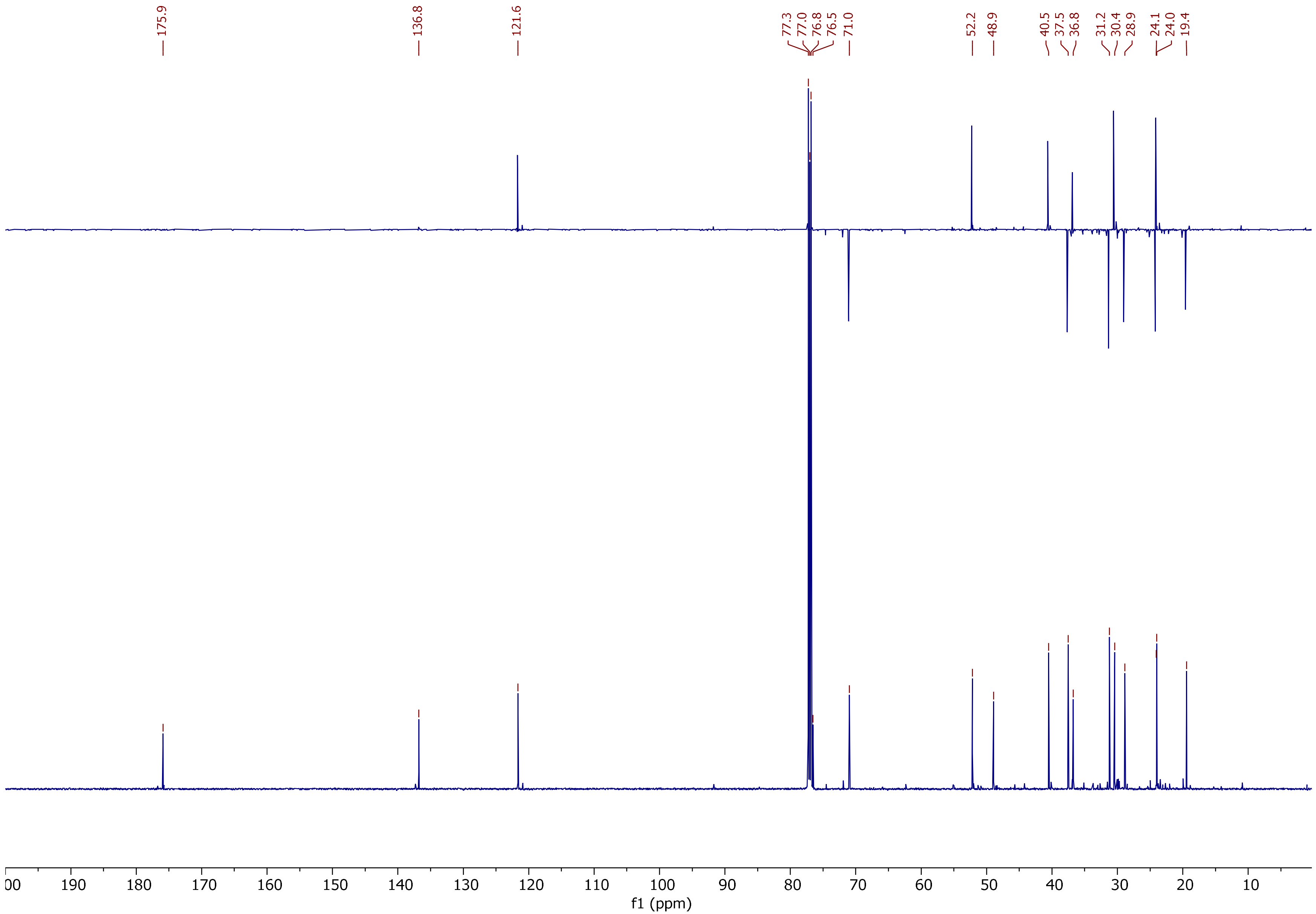
175.9
136.8
121.6
77.3
77.0
76.8
76.5
71.0
52.2
48.9
40.5
37.5
36.8
31.2
30.4
28.9
24.1
24.0
19.4
00
190
180
170
160
150
140
130
120
110
100
90
80
70
60
50
40
30
20
10
f1 (ppm)

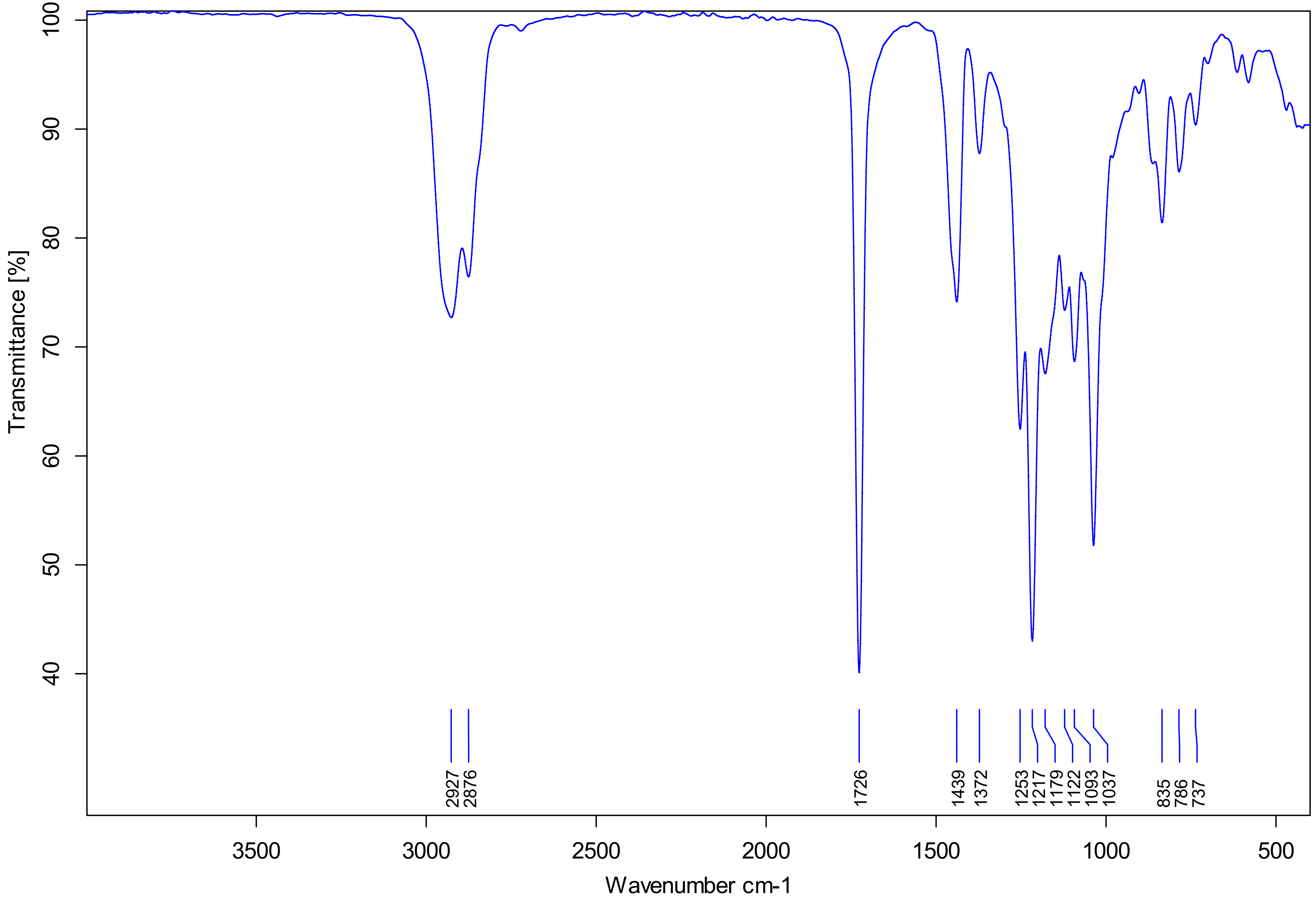

Transmittance [%]
100
90
80
70
60
50
40
2927
2876
1726
1439
1372
1253
1217
1179
1122
1093
1037
835
786
737
3500
3000
2500
2000
1500
1000
500
Wavenumber cm-1

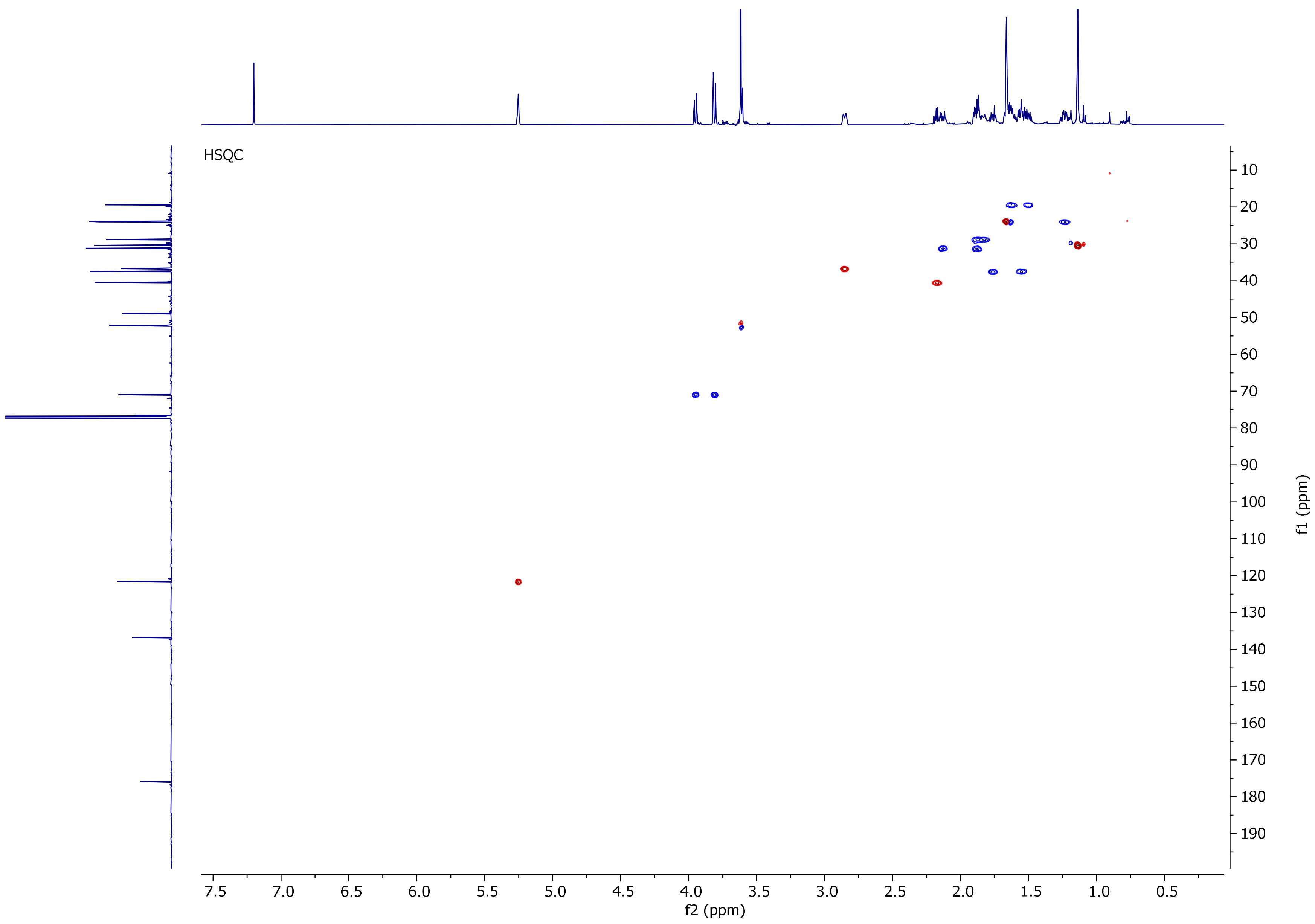
HSQC
f2 (ppm)
f1 (ppm)

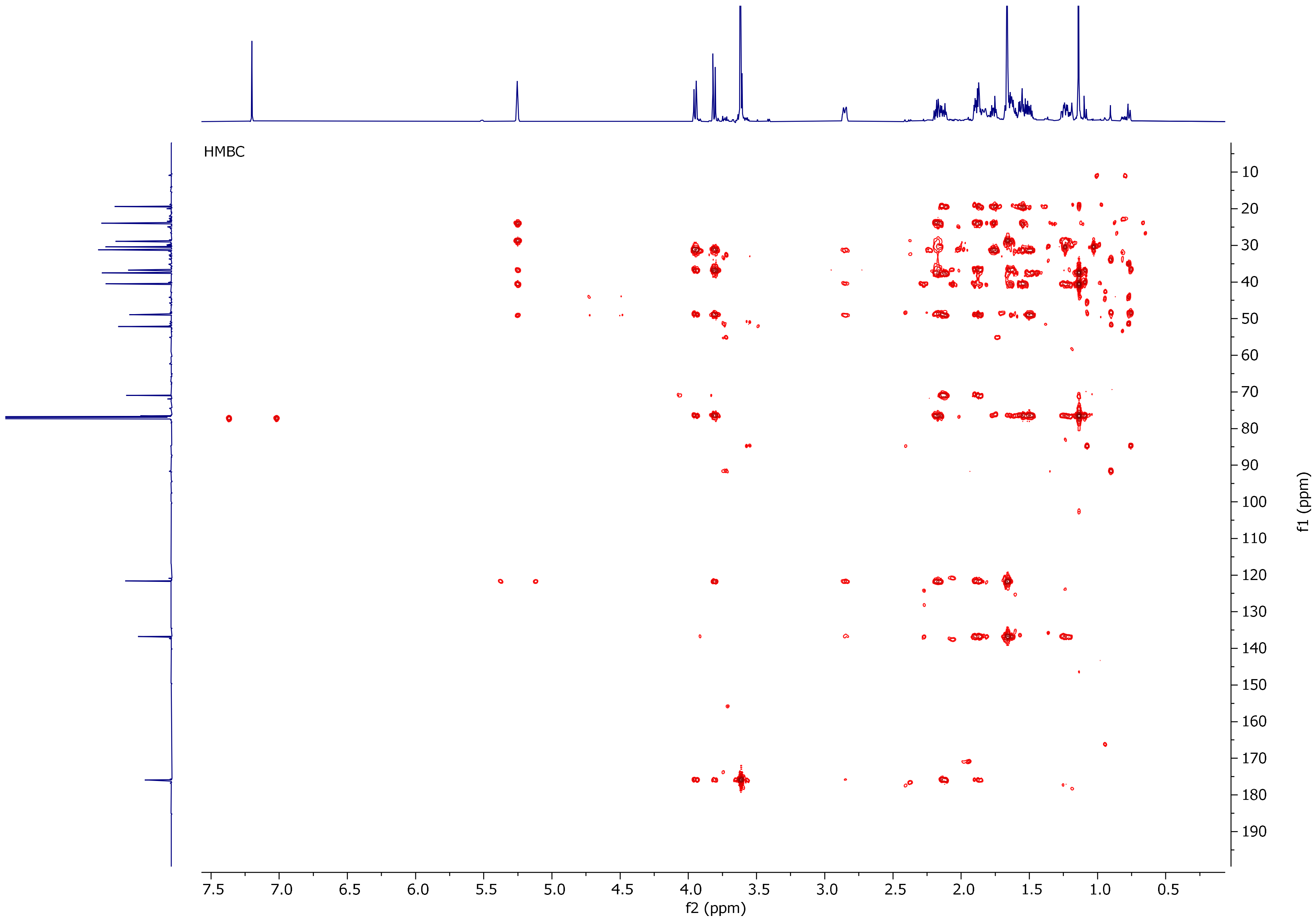
HMBC
f2 (ppm)
f1 (ppm)
7.5
7.0
6.5
6.0
5.5
5.0
4.5
4.0
3.5
3.0
2.5
2.0
1.5
1.0
0.5
10
20
30
40
50
60
70
80
90
100
110
120
130
140
150
160
170
180
190

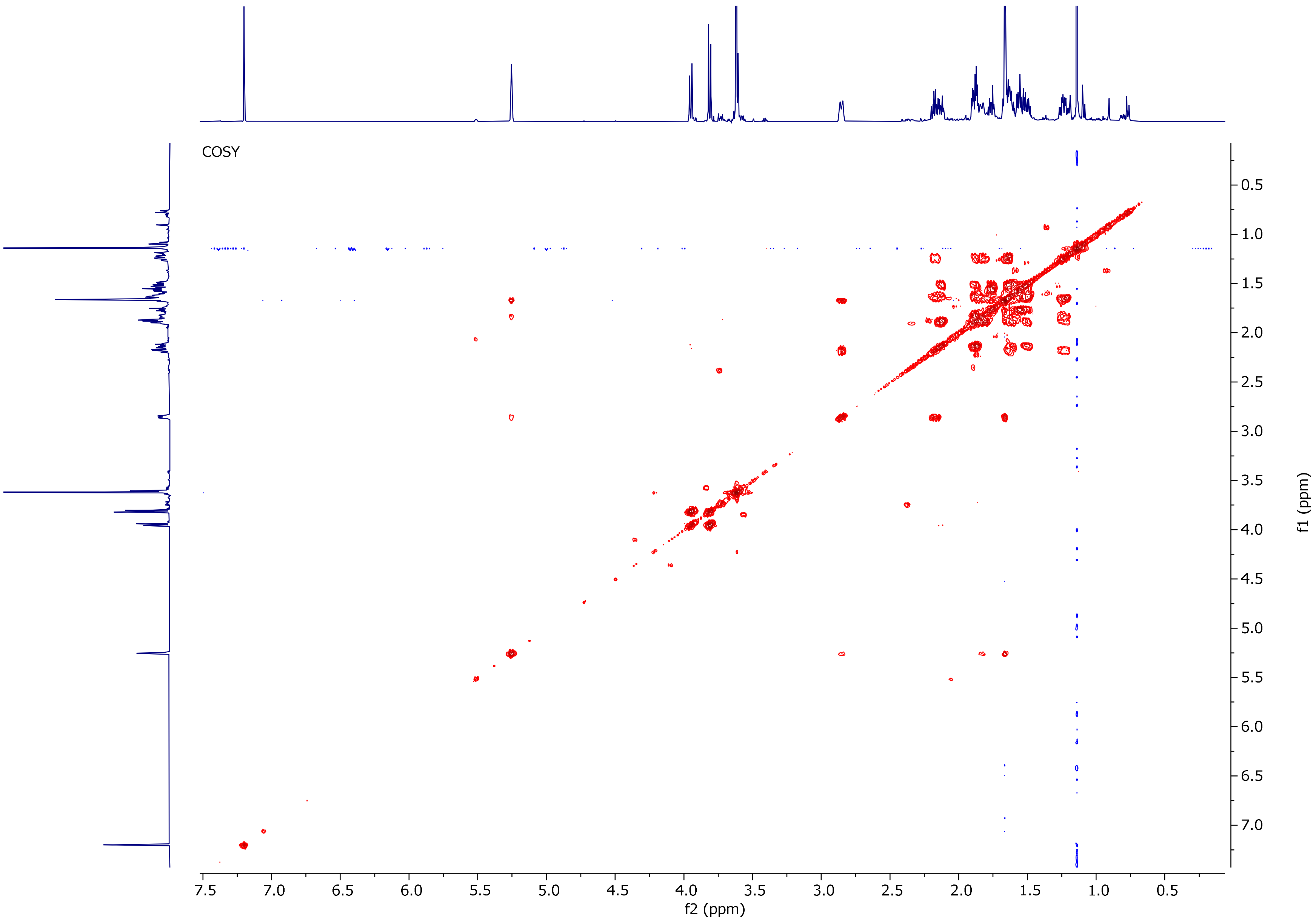
COSY
7.5
7.0
6.5
6.0
5.5
5.0
4.5
4.0
3.5
3.0
2.5
2.0
1.5
1.0
0.5
f2 (ppm)
f1 (ppm)

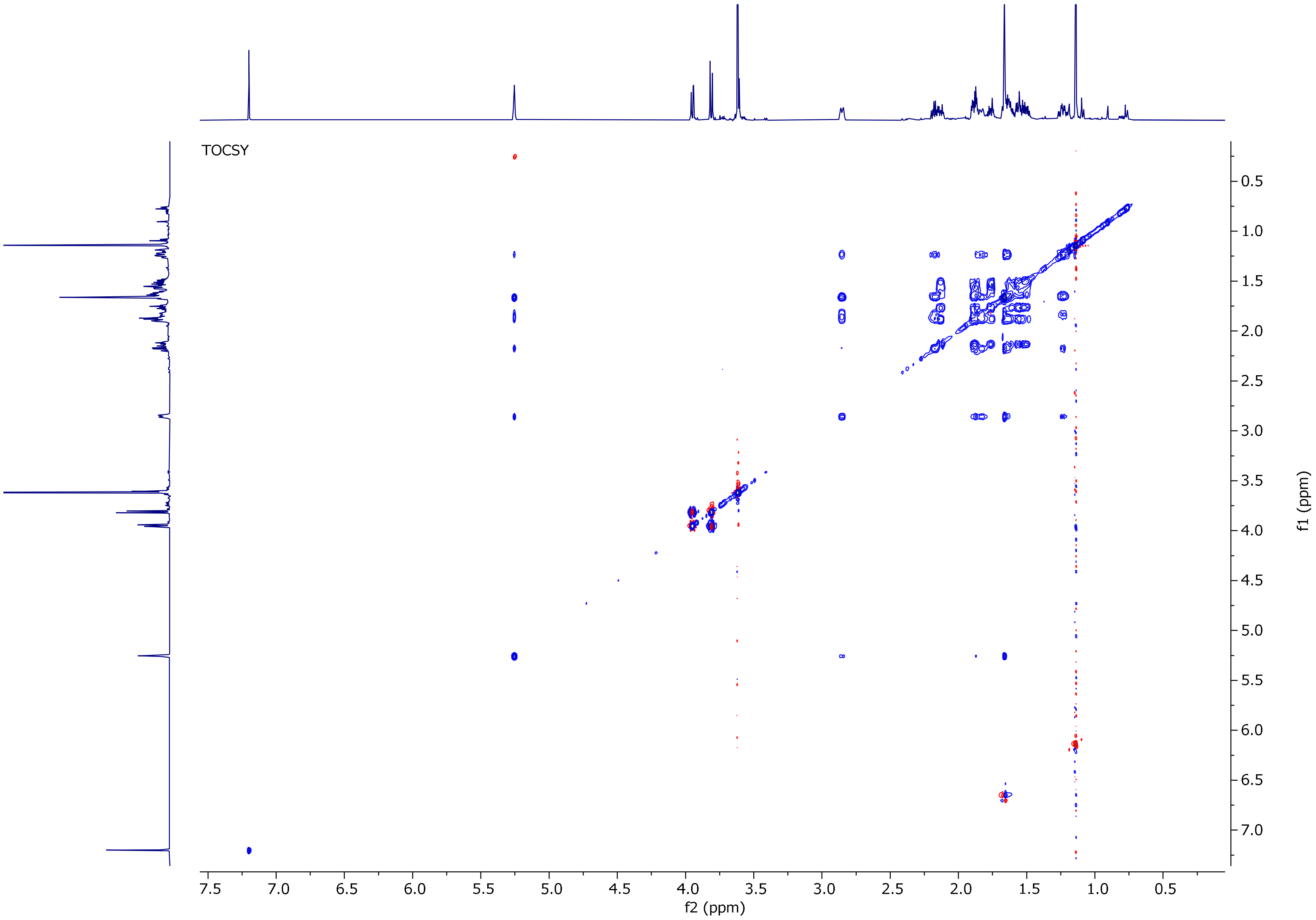

TOCSY
f2 (ppm)
f1 (ppm)

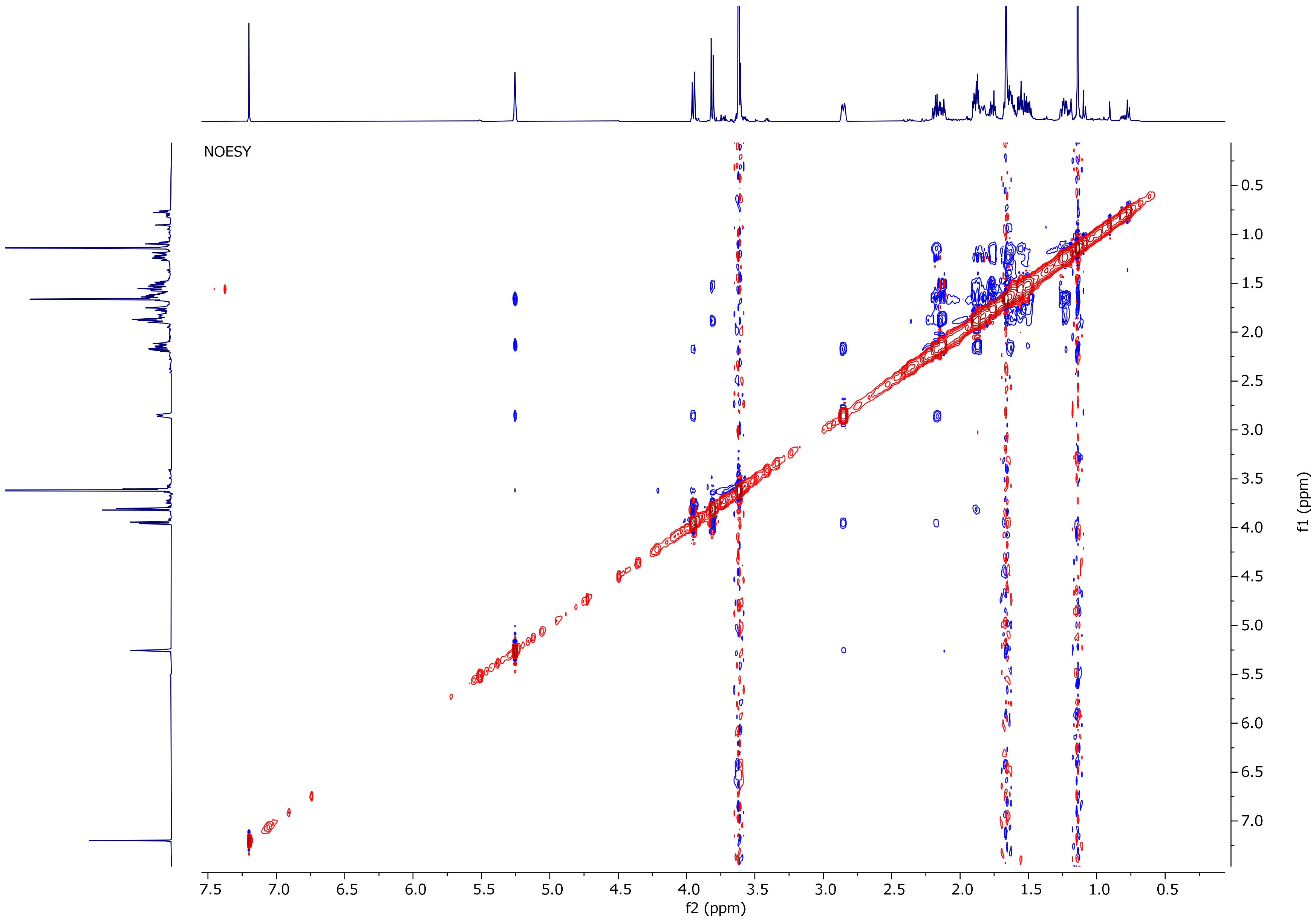
NOESY
7.5
7.0
6.5
6.0
5.5
5.0
4.5
4.0
3.5
3.0
2.5
2.0
1.5
1.0
0.5
f2 (ppm)
f1 (ppm)

^{1}H NMR, DEPT 135, ^{13}C NMR and IR of **19a/19b**

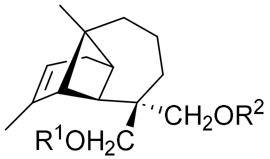

19a R^1= MOM R^2= H
19b R^1= H R^2= MOM

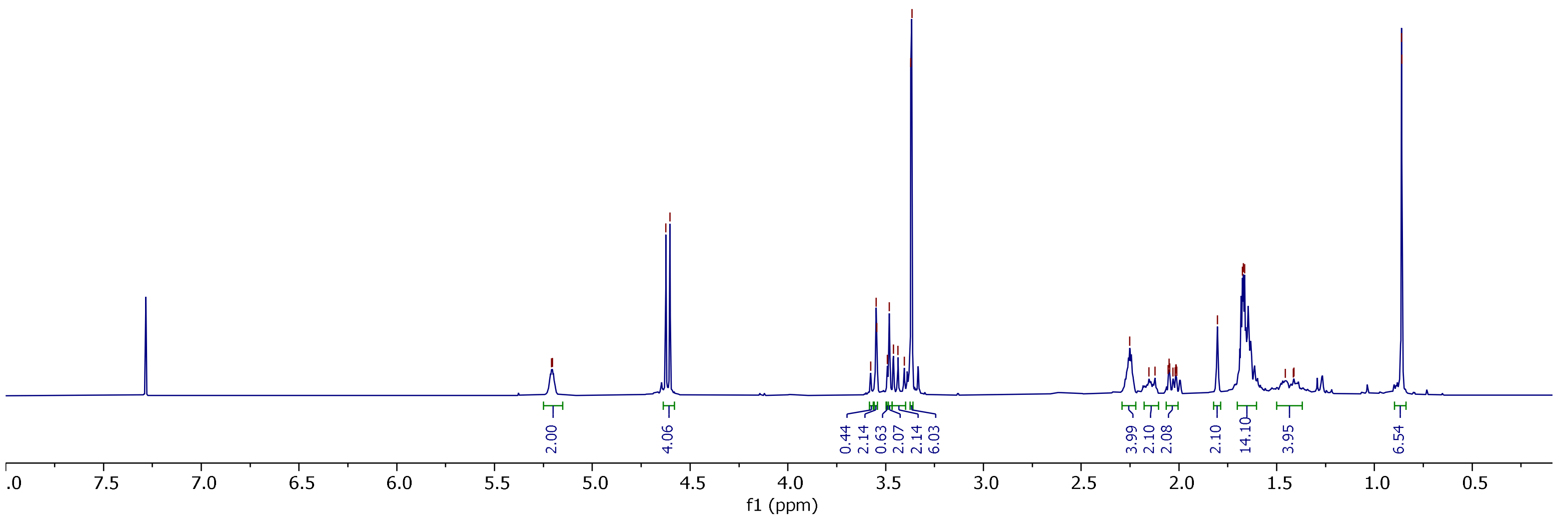

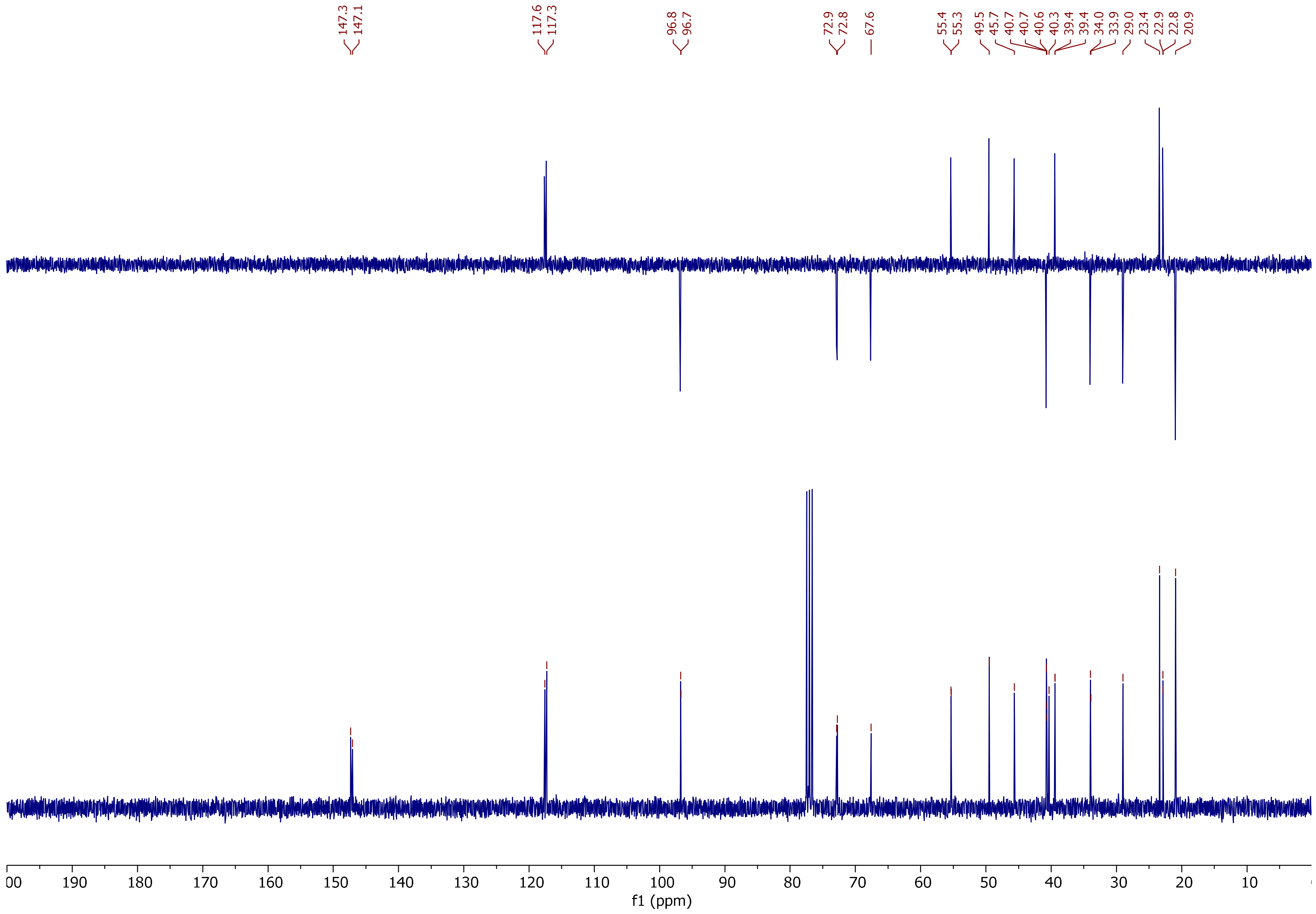
147.3
147.1
117.6
117.3
96.8
96.7
72.9
72.8
67.6
55.4
55.3
49.5
45.7
40.7
40.7
40.6
40.3
39.4
39.4
34.0
33.9
29.0
23.4
22.9
22.8
20.9
f1 (ppm)

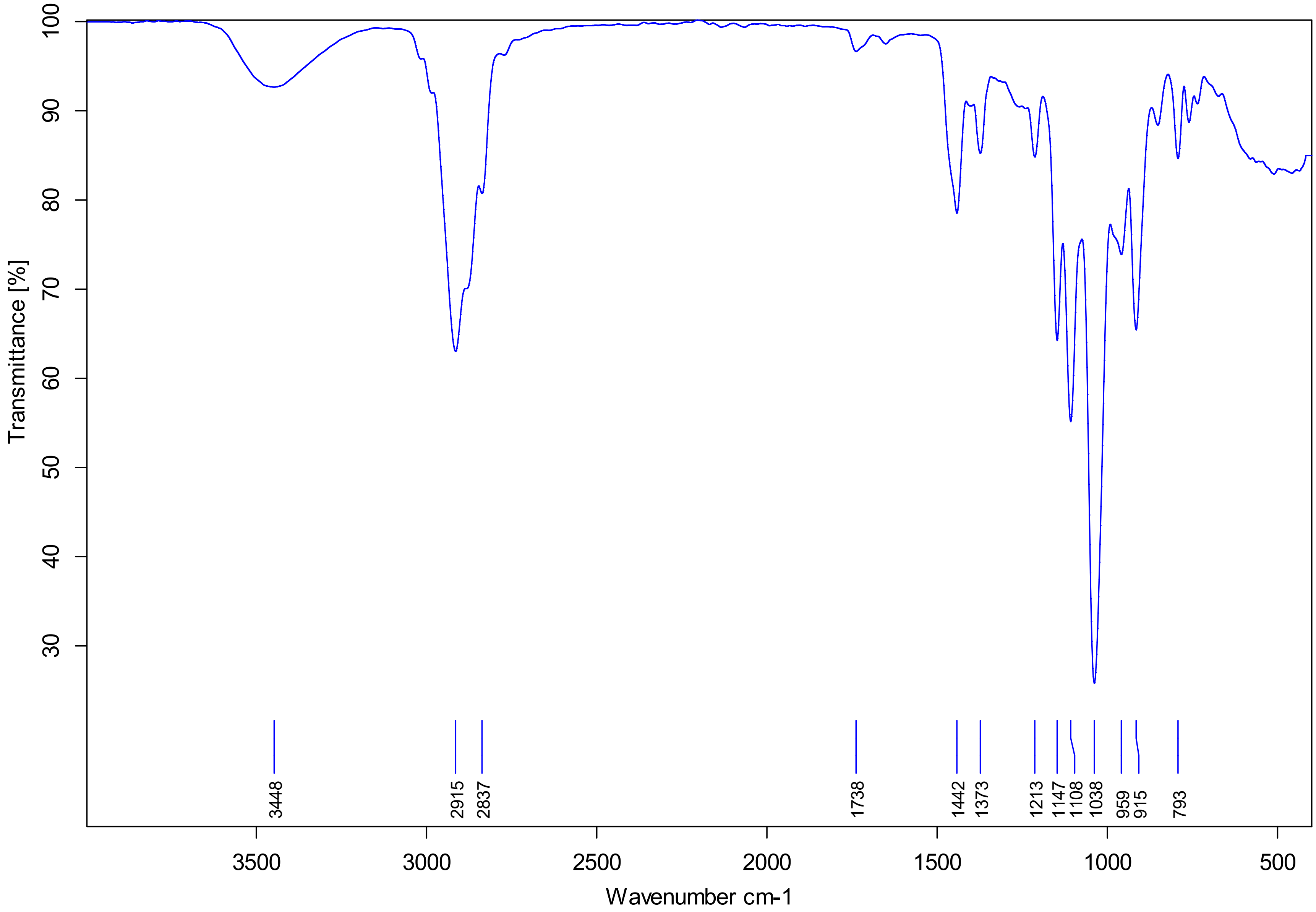
100
90
80
70
60
50
40
30
Transmittance [%]
3448
2915
2837
1738
1442
1373
1213
1147
1108
1038
959
915
793
3500
3000
2500
2000
1500
1000
500
Wavenumber cm-1

^{1}H NMR, DEPT 135, ^{13}C NMR, IR, HSQC, HMBC, COSY, TOCSY and NOESY of **20**

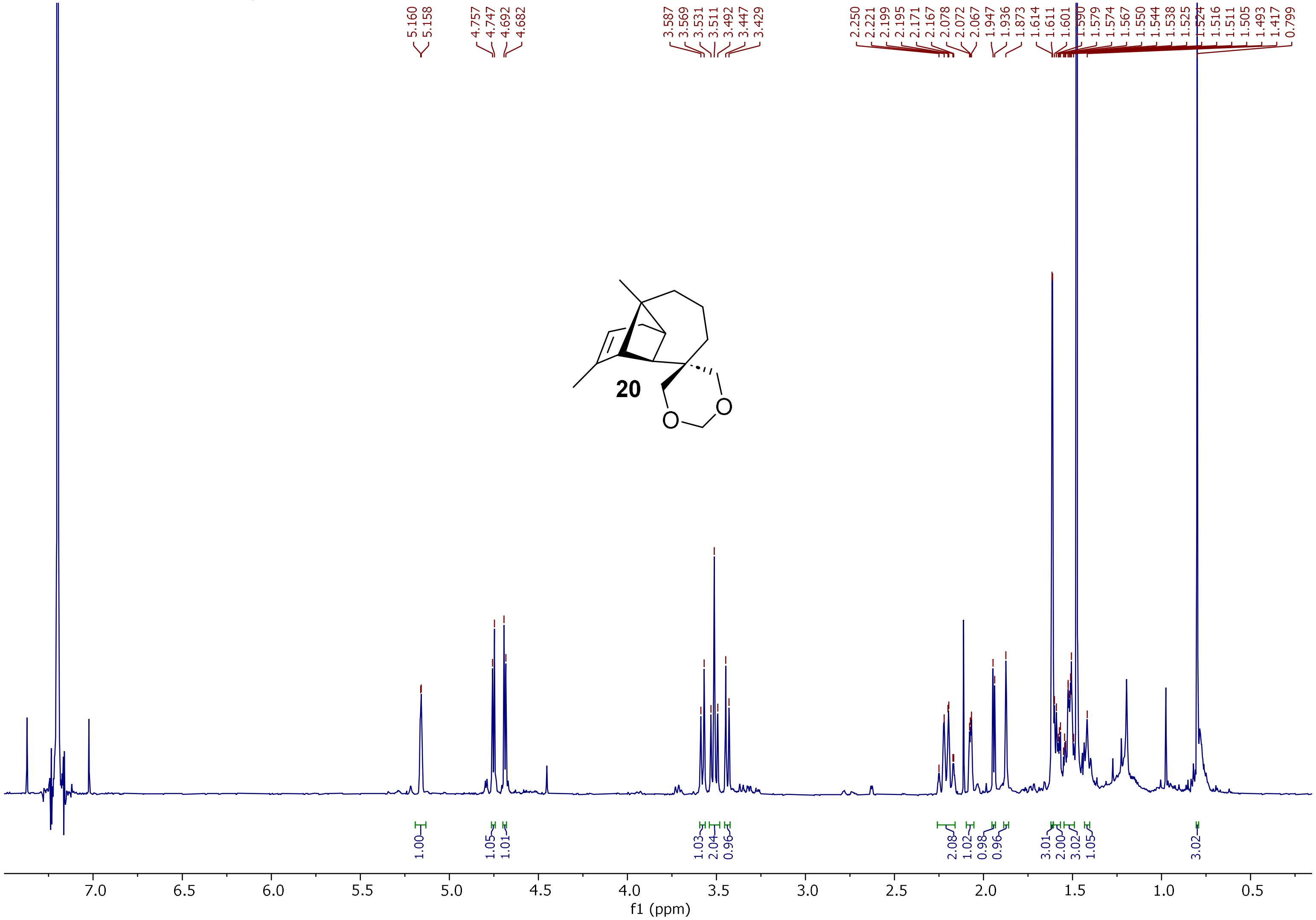

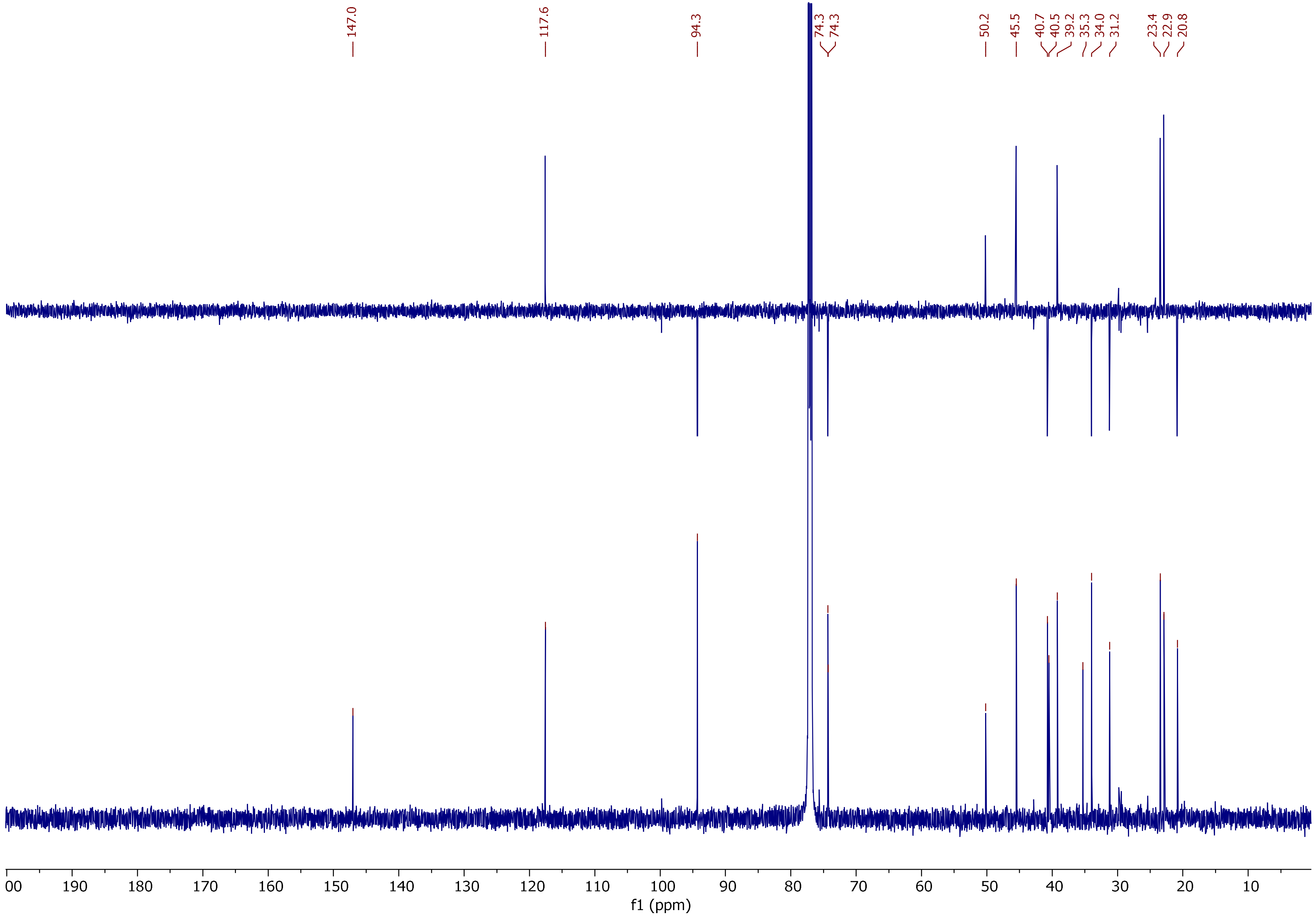
147.0
117.6
94.3
74.3
74.3
50.2
45.5
40.7
40.5
39.2
35.3
34.0
31.2
23.4
22.9
20.8
00
190
180
170
160
150
140
130
120
110
100
90
80
70
60
50
40
30
20
10
f1 (ppm)

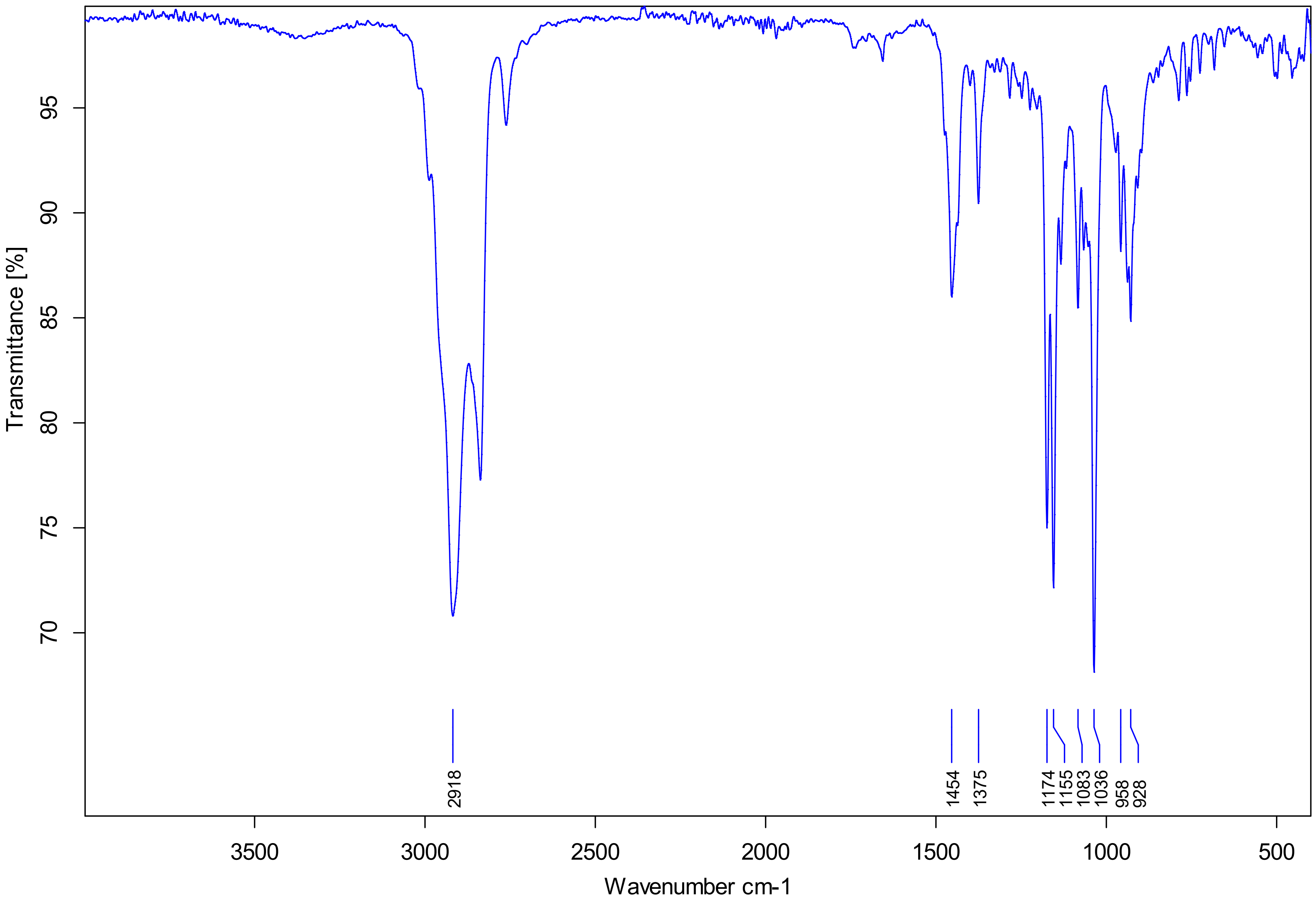
Transmittance [%]
95
90
85
80
75
70
2918
1454
1375
1174
1155
1083
1036
958
928
3500
3000
2500
2000
1500
1000
500
Wavenumber cm-1

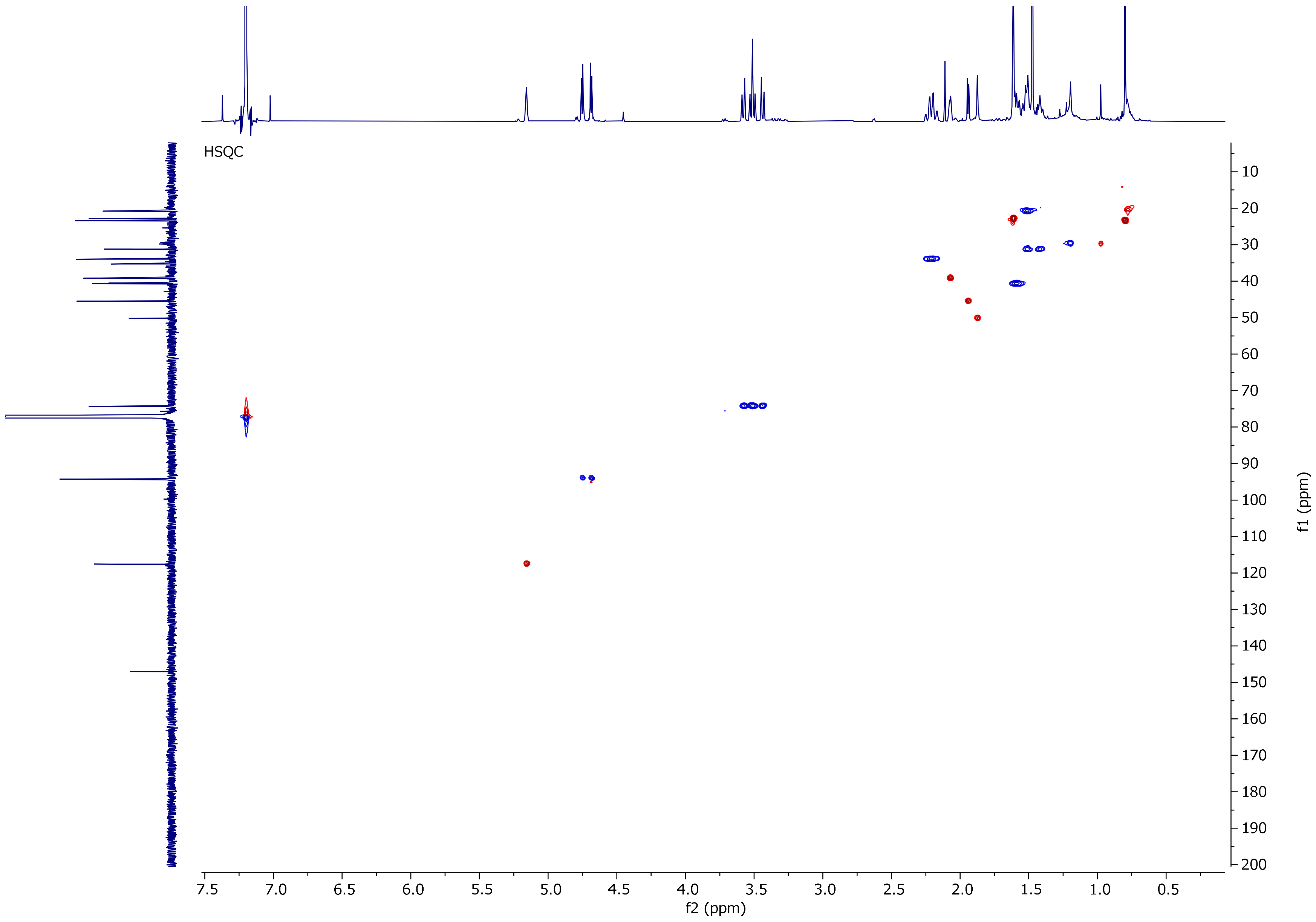
HSQC
f2 (ppm)
f1 (ppm)

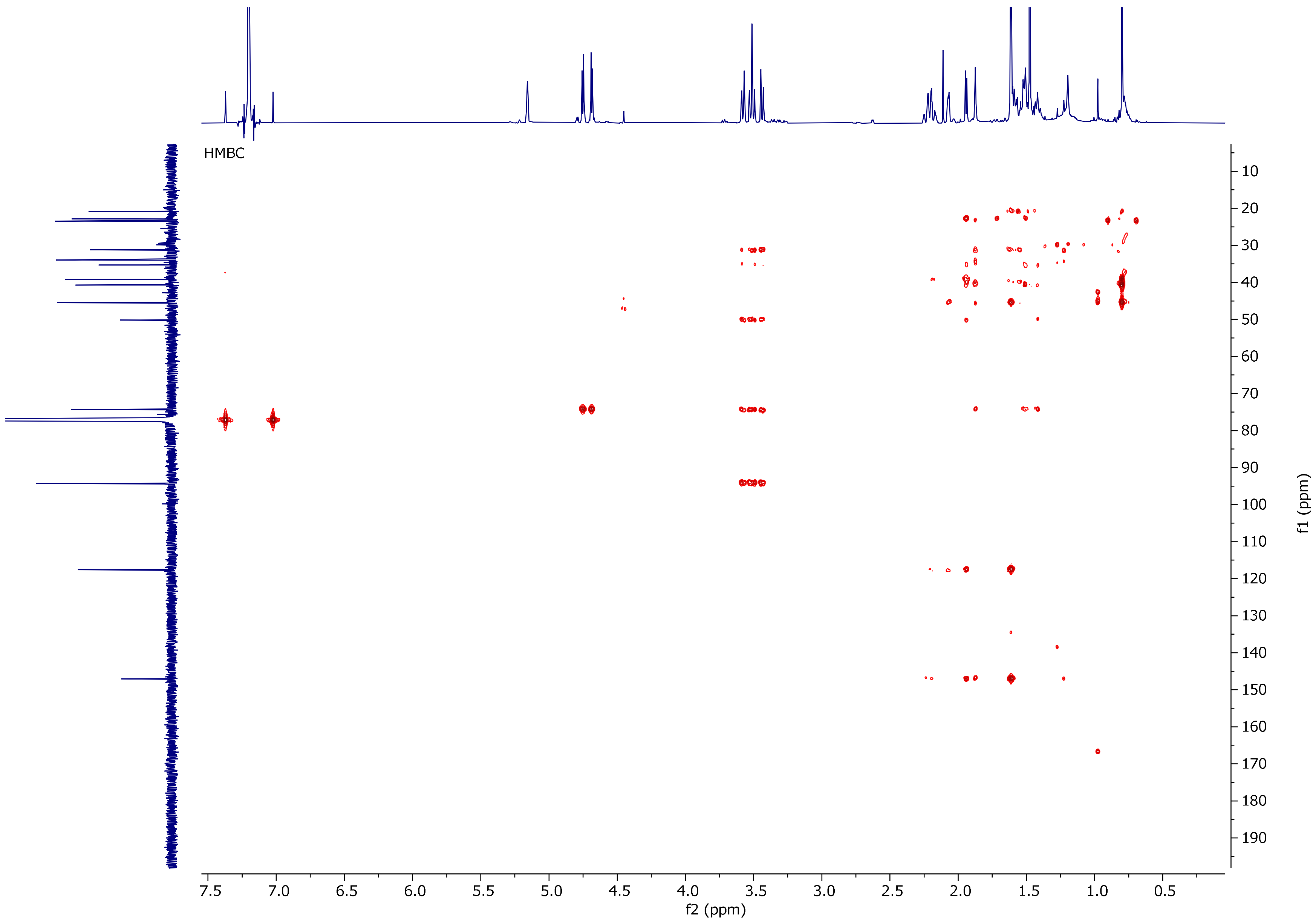
HMBC
f2 (ppm)
f1 (ppm)

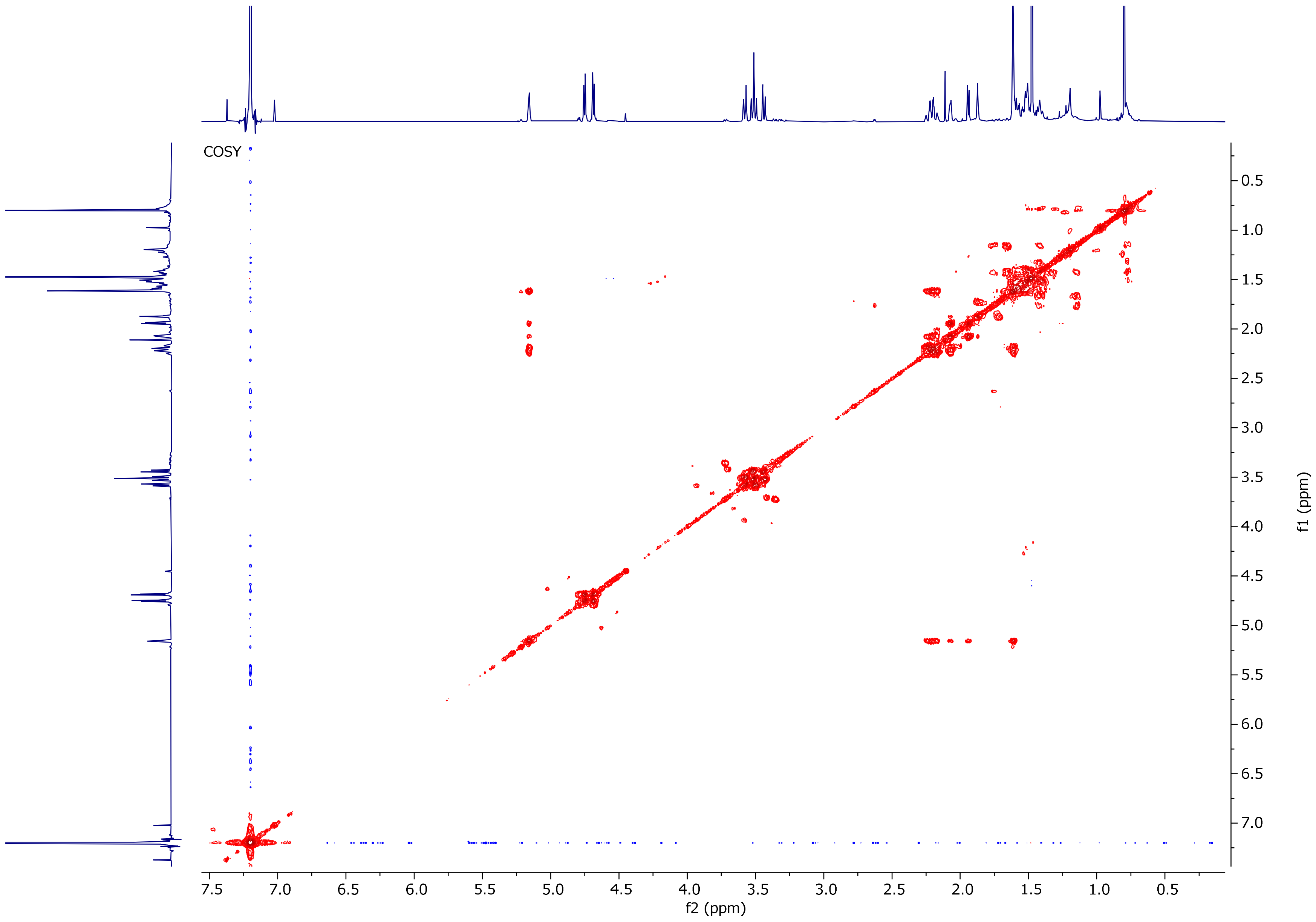
COSY
f2 (ppm)
f1 (ppm)

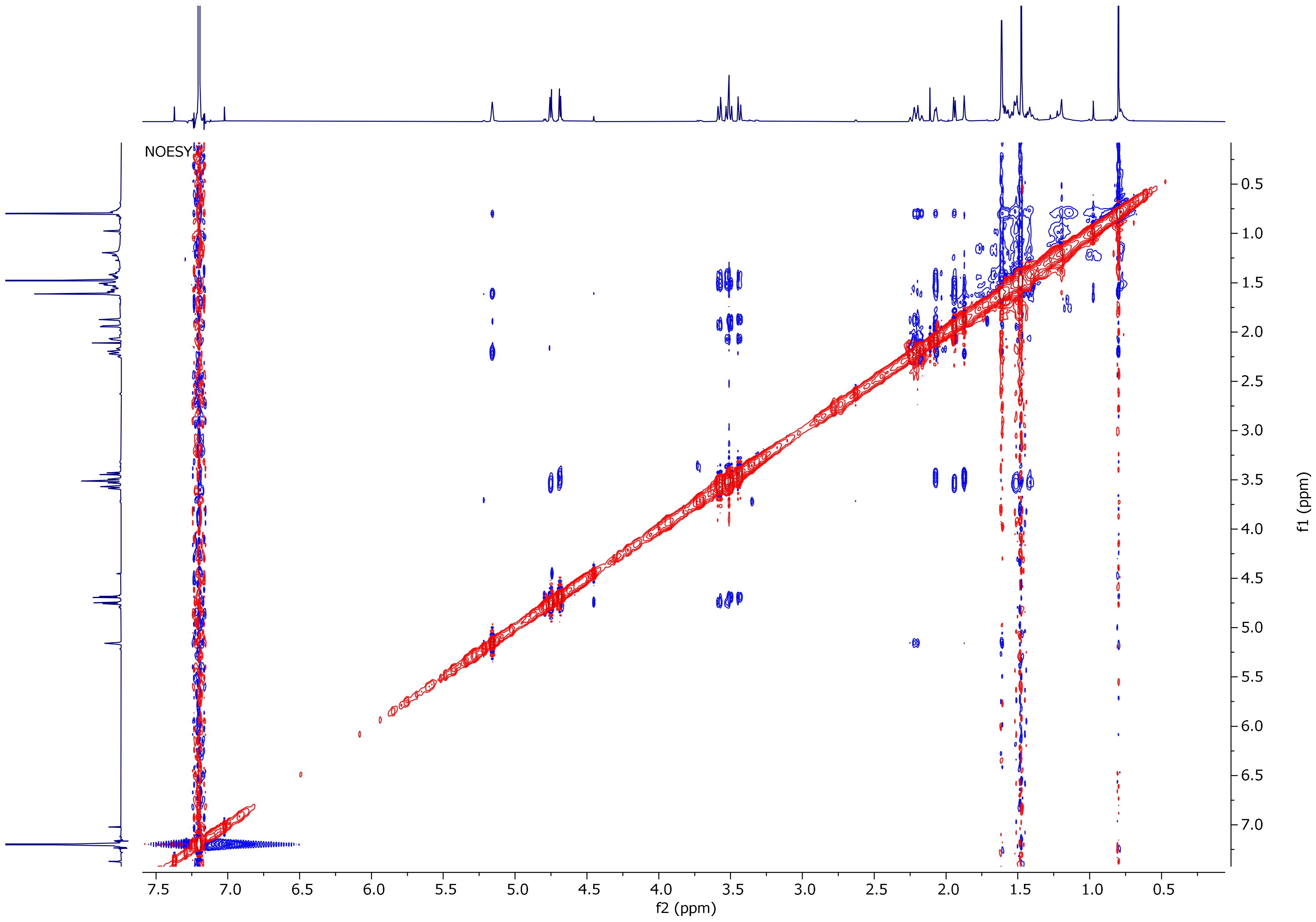
NOESY
7.5
7.0
6.5
6.0
5.5
5.0
4.5
4.0
3.5
3.0
2.5
2.0
1.5
1.0
0.5
f2 (ppm)
f1 (ppm)

^{1}H NMR, DEPT 135, ^{13}C NMR and IR of **21**

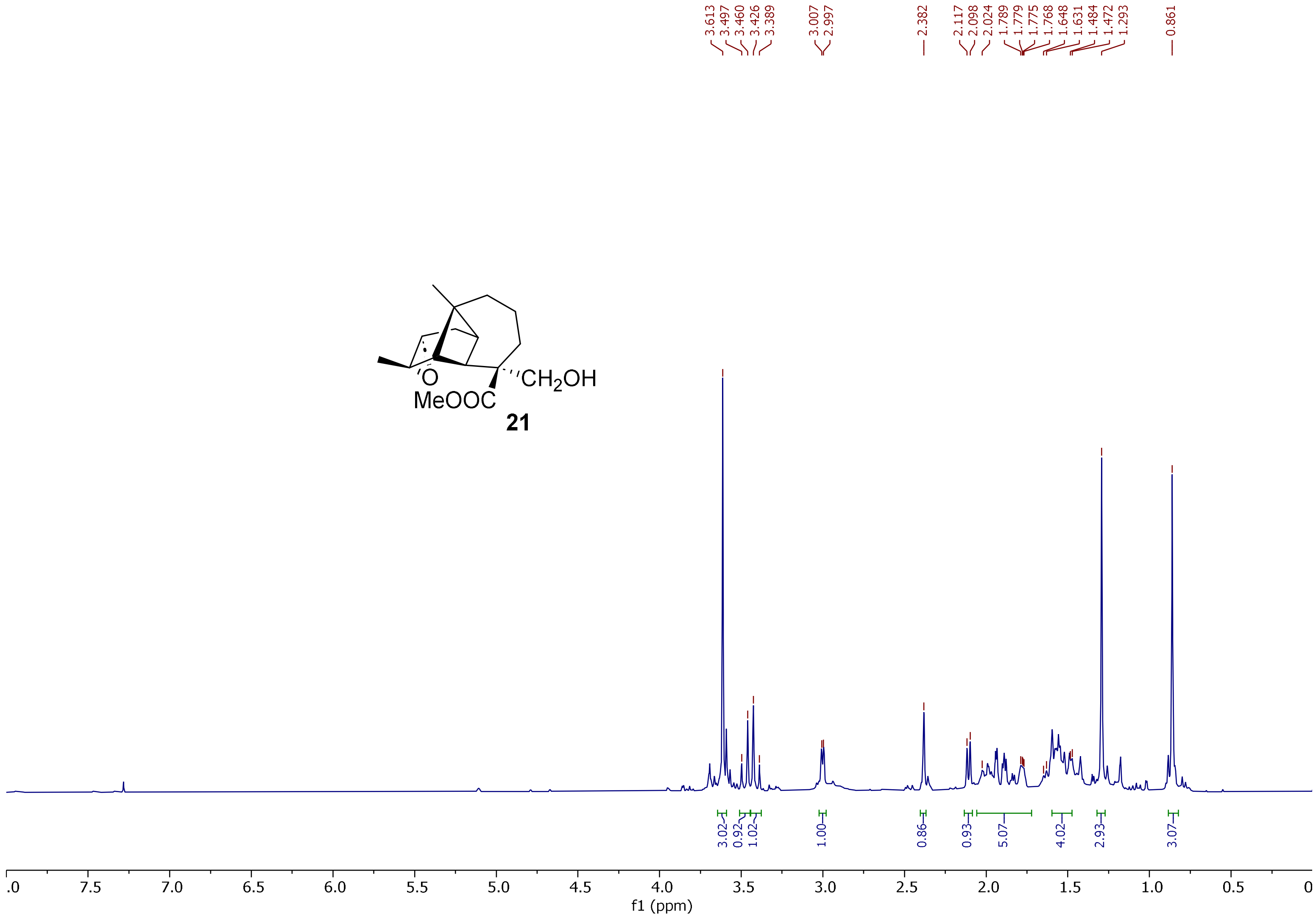

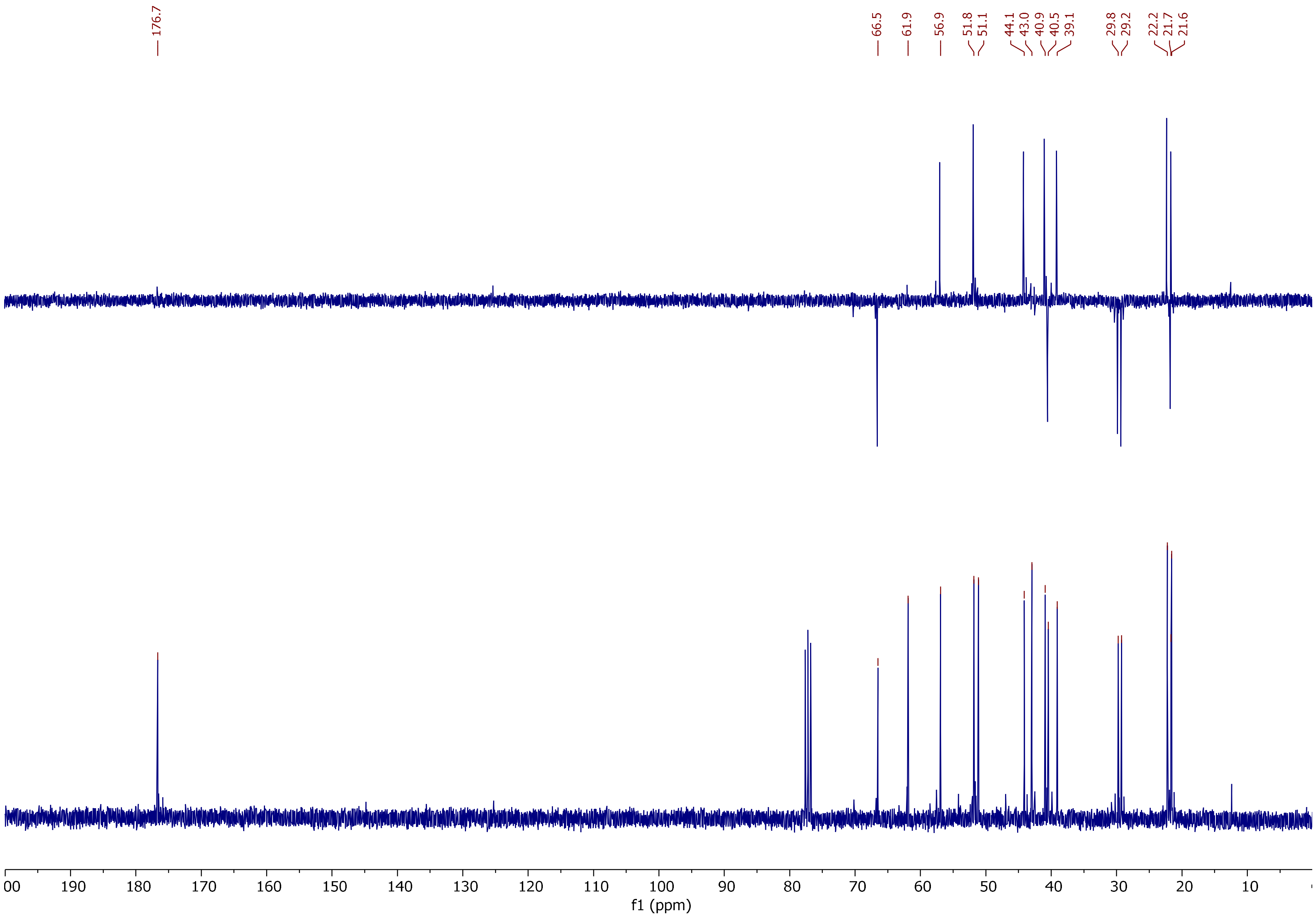

176.7
66.5
61.9
56.9
51.8
51.1
44.1
43.0
40.9
40.5
39.1
29.8
29.2
22.2
21.7
21.6
00
190
180
170
160
150
140
130
120
110
100
90
80
70
60
50
40
30
20
10
f1 (ppm)

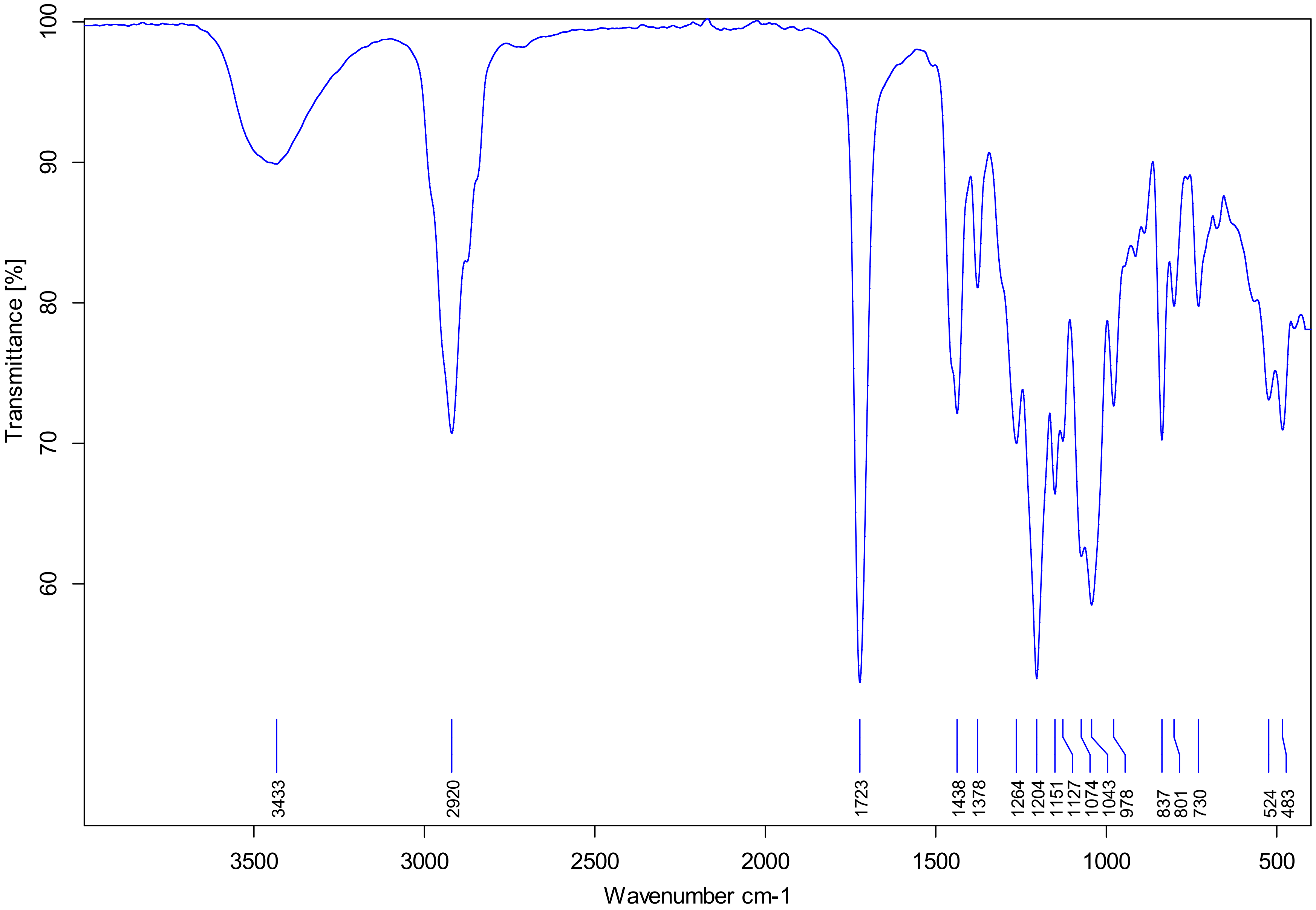

Transmittance [%]
100
90
80
70
60
3433
2920
1723
1438
1378
1264
1204
1151
1127
1074
1043
978
837
801
730
524
483
3500
3000
2500
2000
1500
1000
500
Wavenumber cm-1

^{1}H NMR, DEPT 135, ^{13}C NMR and IR of **22**

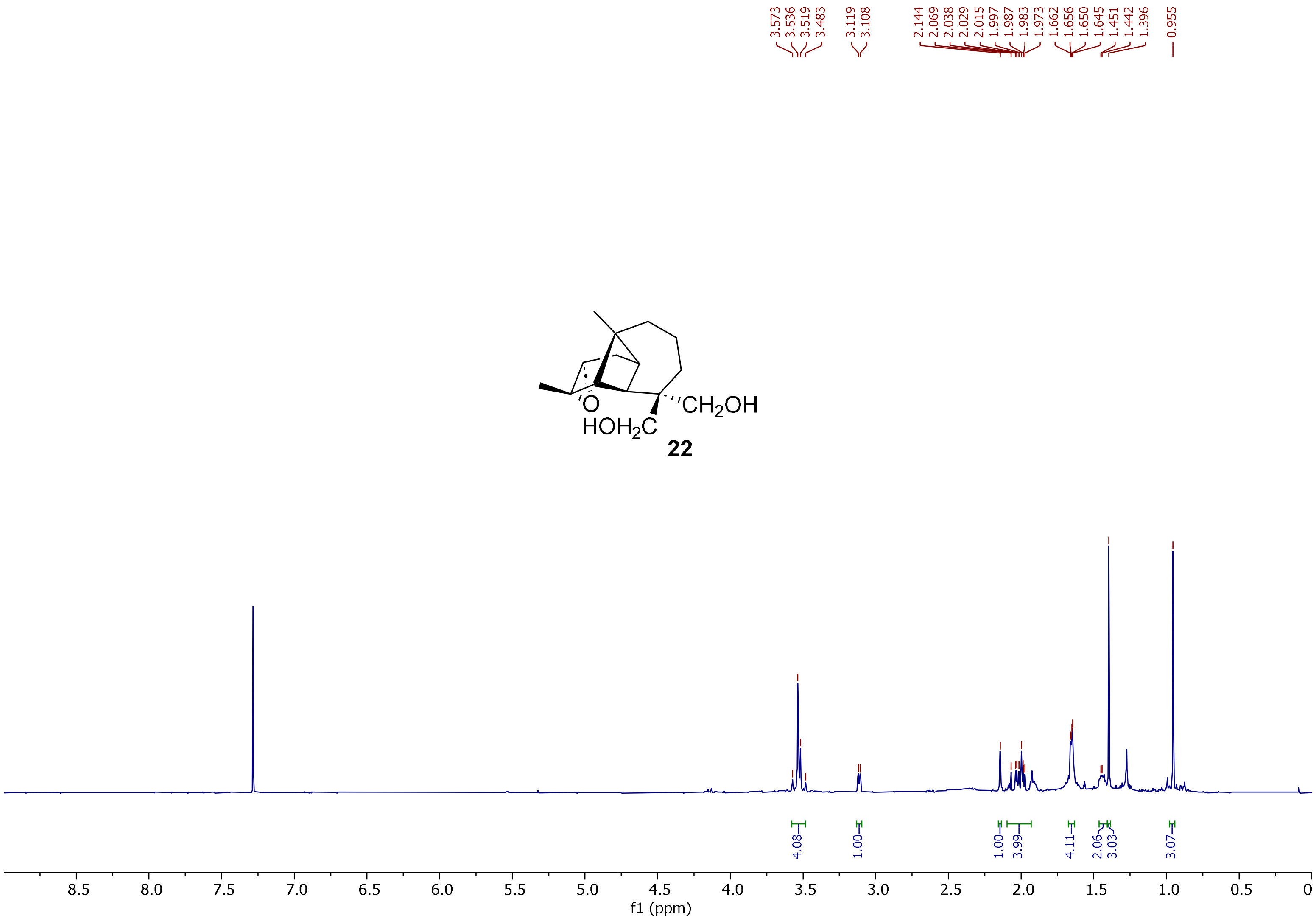

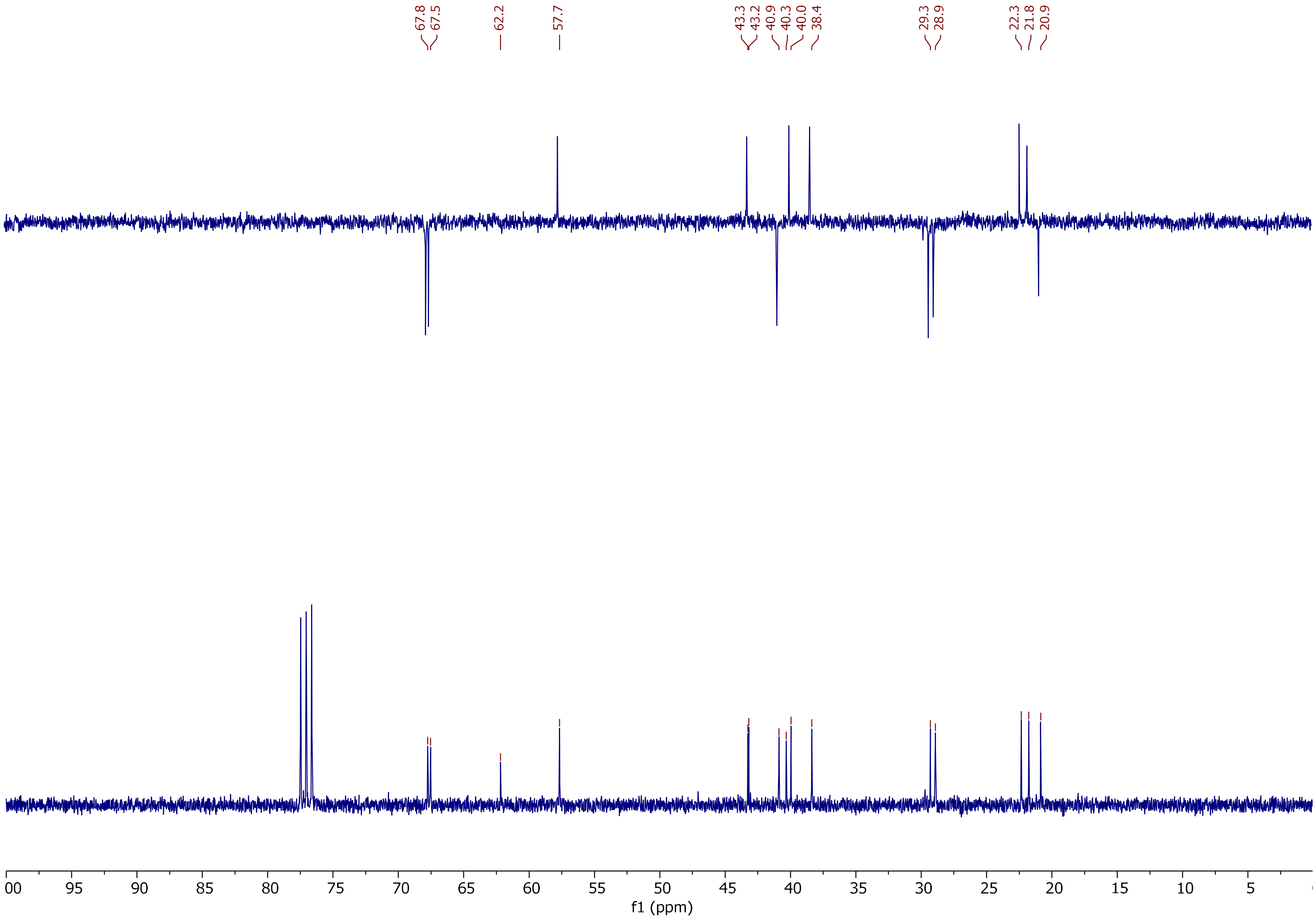
67.8
67.5
62.2
57.7
43.3
43.2
40.9
40.3
40.0
38.4
29.3
28.9
22.3
21.8
20.9
00
95
90
85
80
75
70
65
60
55
50
45
40
35
30
25
20
15
10
5
f1 (ppm)

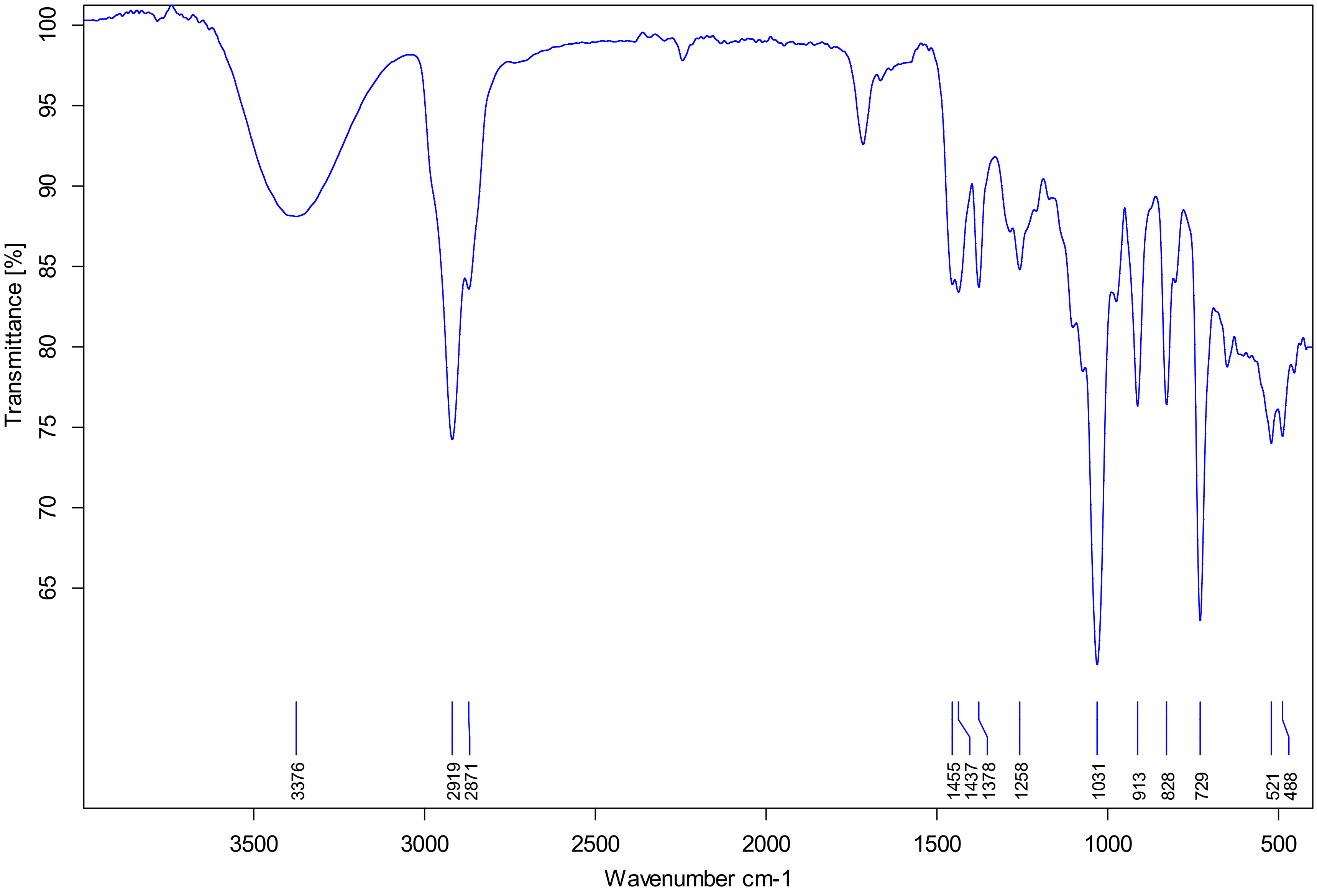

100
95
90
85
80
75
70
65
Transmittance [%]
3376
2919
2871
1455
1437
1378
1258
1031
913
828
729
521
488
3500
3000
2500
2000
1500
1000
500
Wavenumber cm-1

^{1}H NMR, DEPT 135, ^{13}C NMR, IR, HSQC, HMBC, COSY, TOCSY and NOESY of **23**

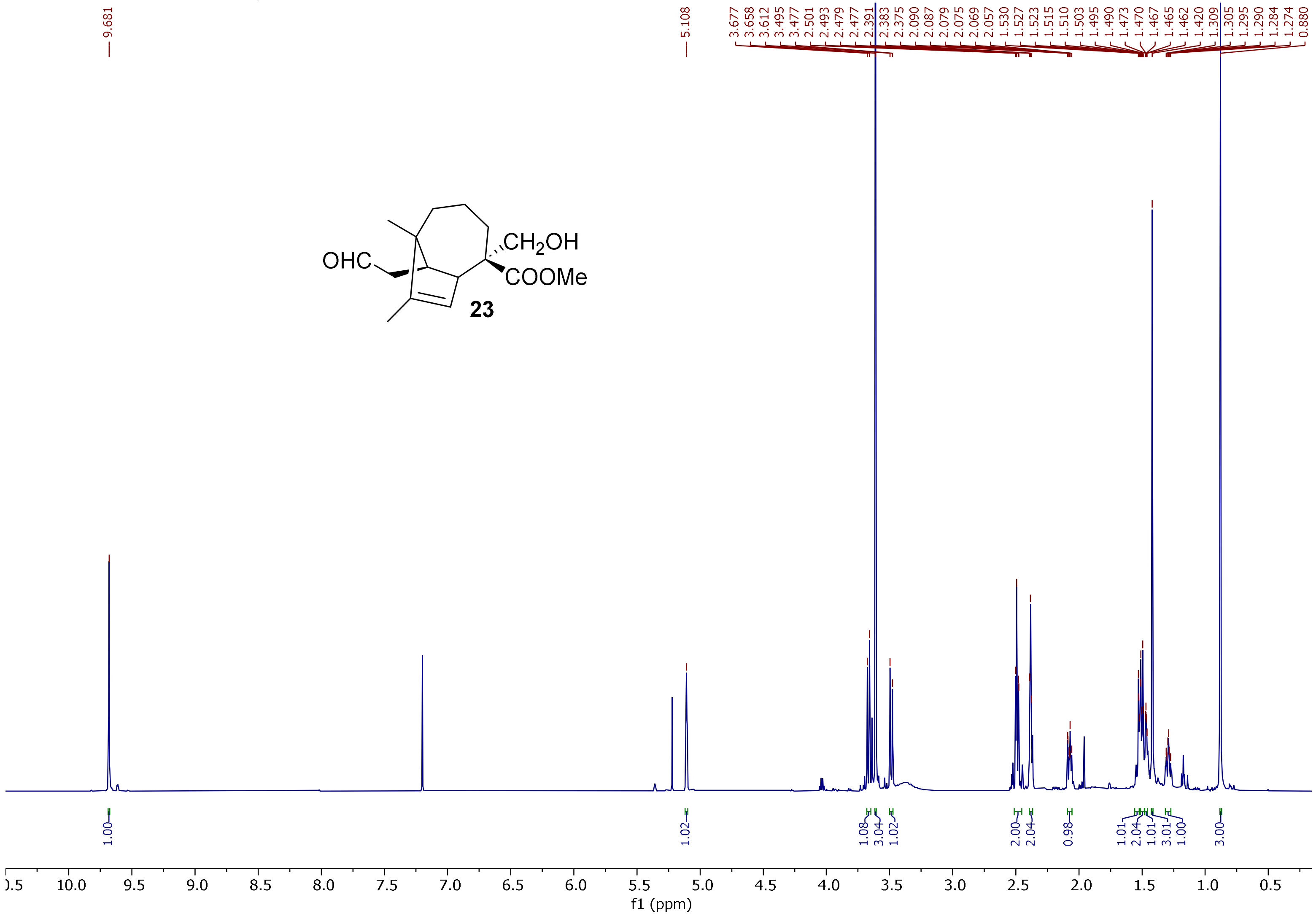

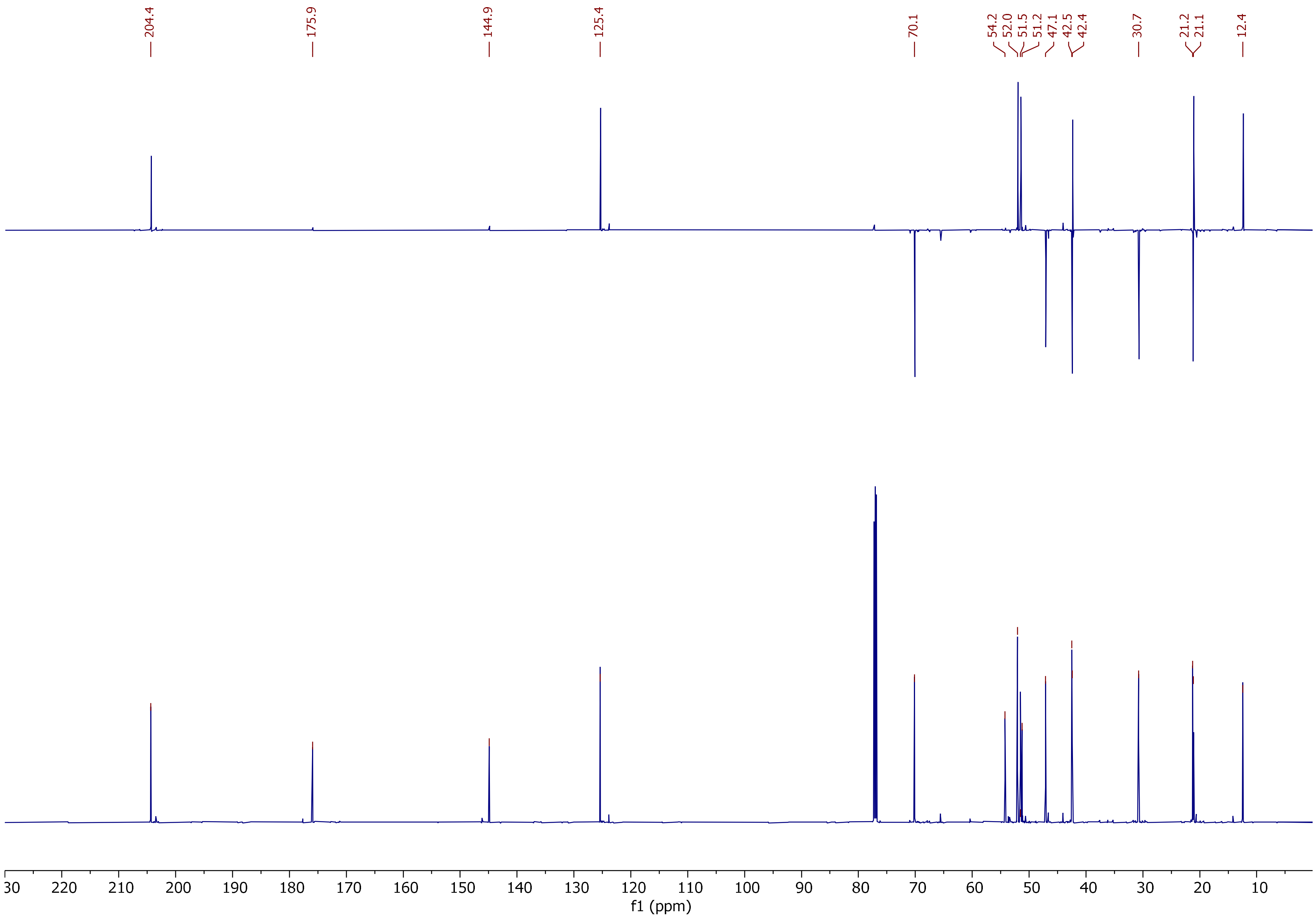
204.4
175.9
144.9
125.4
70.1
54.2
52.0
51.5
51.2
47.1
42.5
42.4
30.7
21.2
21.1
12.4
30
220
210
200
190
180
170
160
150
140
130
120
110
100
90
80
70
60
50
40
30
20
10
f1 (ppm)

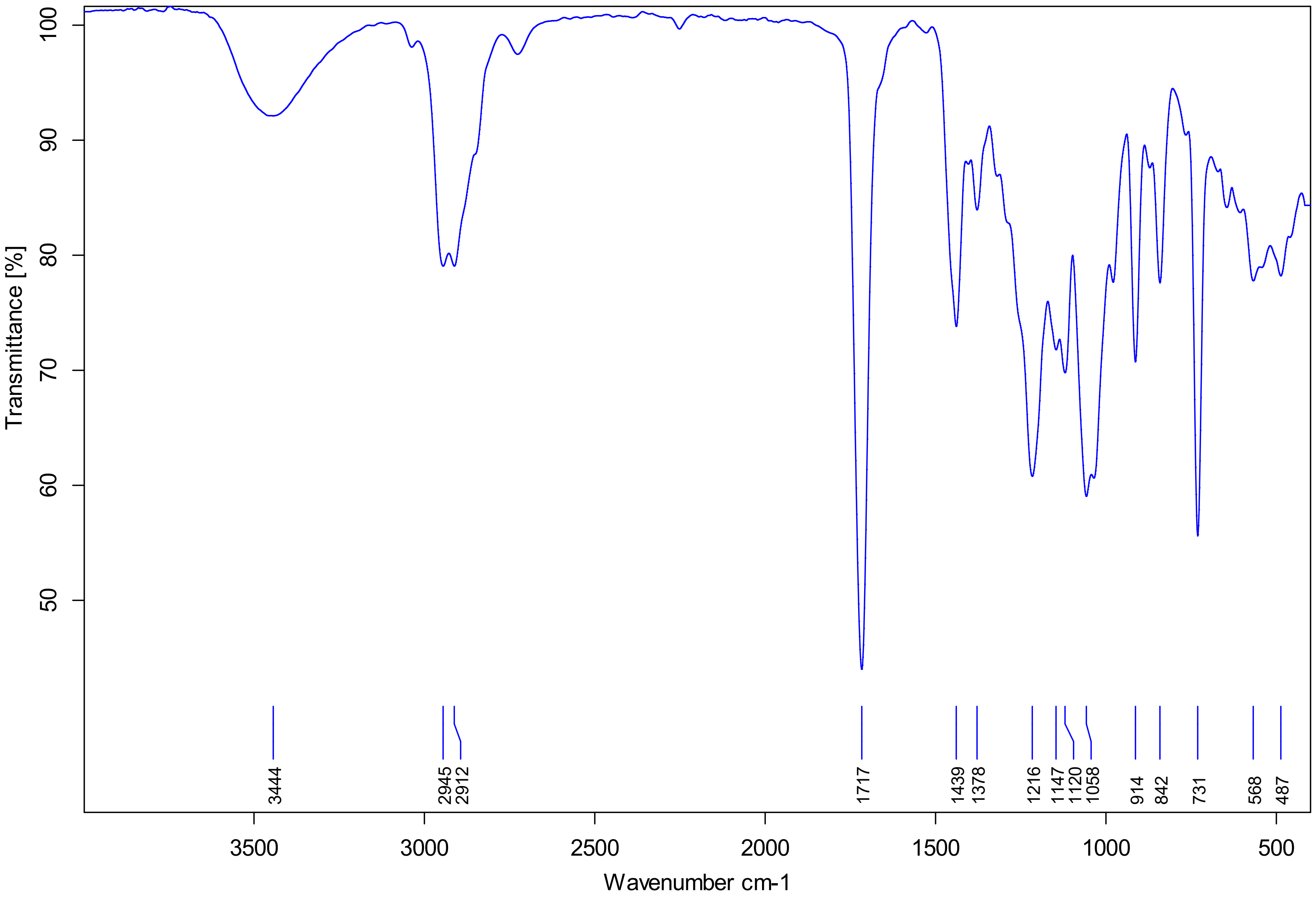

Transmittance [%]
100
90
80
70
60
50
3444
2945
2912
1717
1439
1378
1216
1147
1120
1058
914
842
731
568
487
3500
3000
2500
2000
1500
1000
500
Wavenumber cm-1

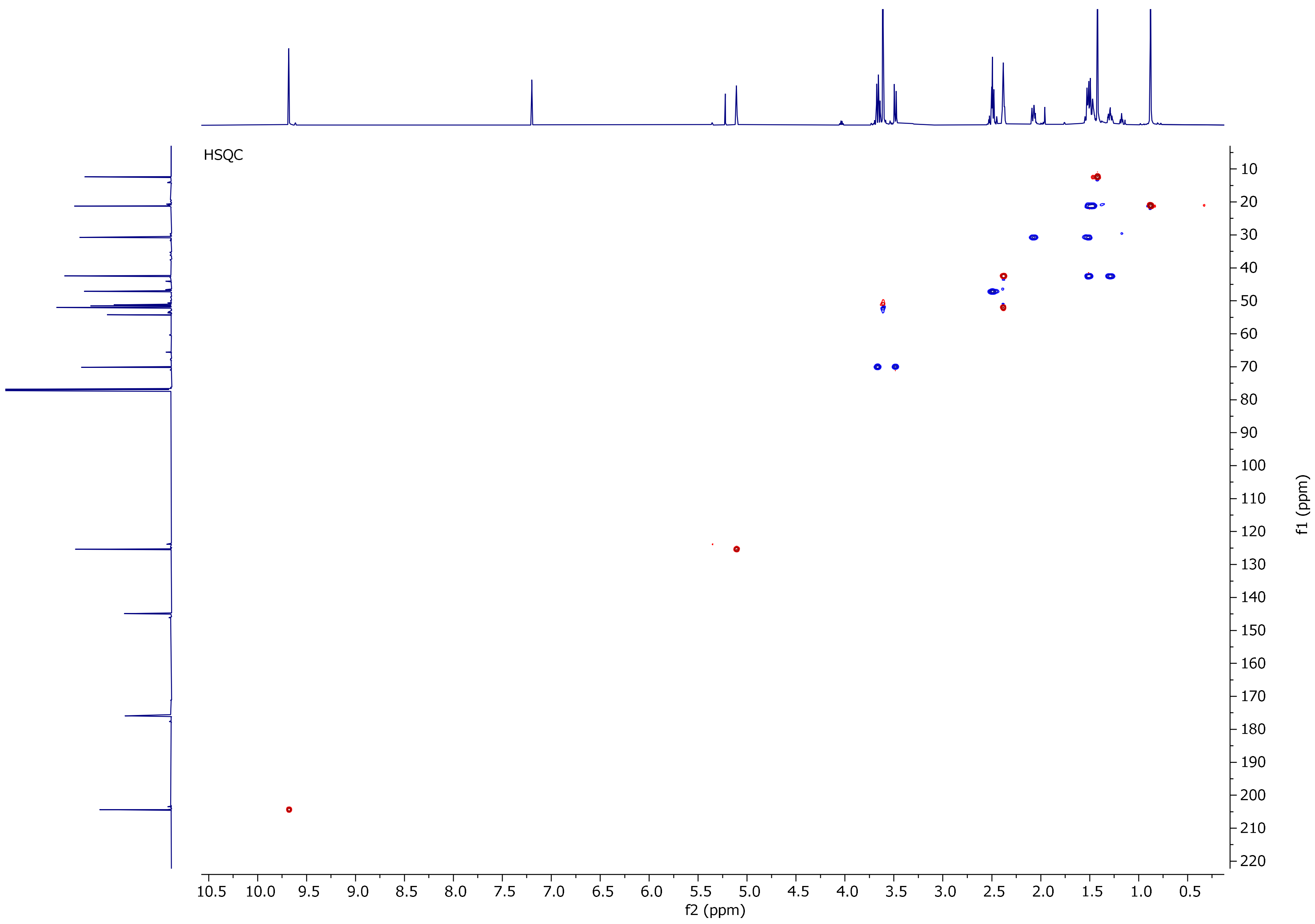

HSQC
f2 (ppm)
f1 (ppm)
10.5 10.0 9.5 9.0 8.5 8.0 7.5 7.0 6.5 6.0 5.5 5.0 4.5 4.0 3.5 3.0 2.5 2.0 1.5 1.0 0.5
10 20 30 40 50 60 70 80 90 100 110 120 130 140 150 160 170 180 190 200 210 220

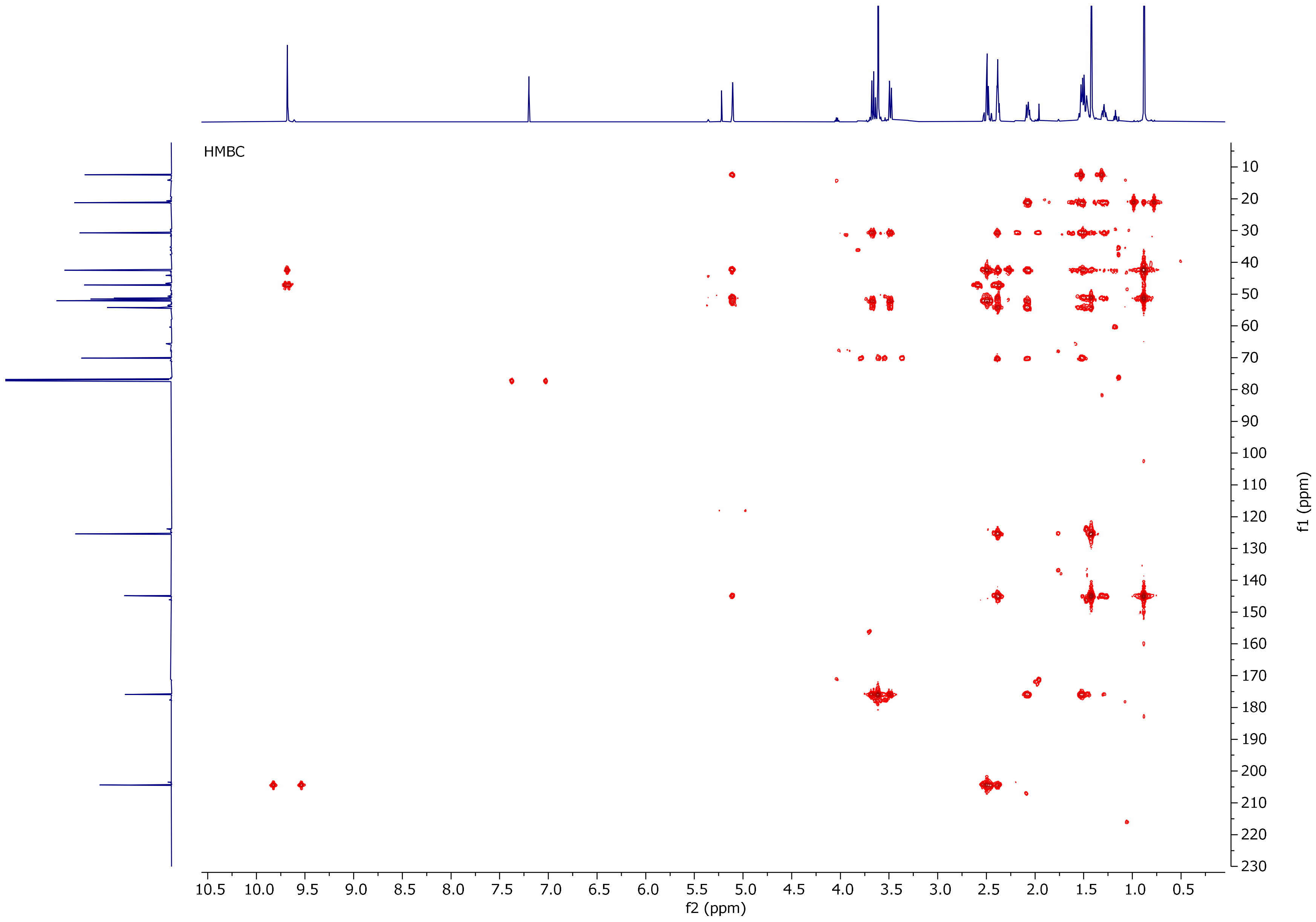

HMBC
f2 (ppm)
f1 (ppm)

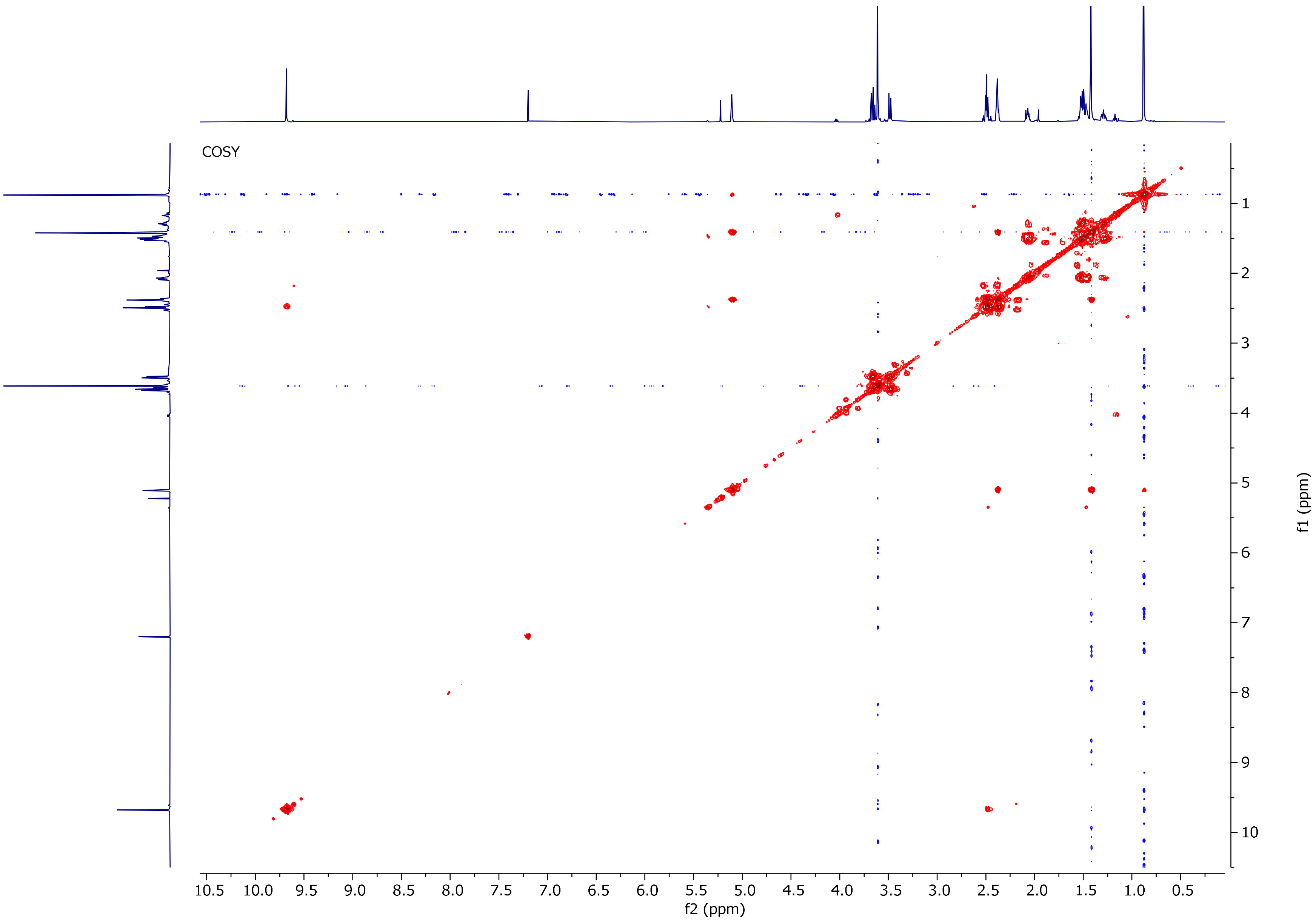
COSY
10.5
10.0
9.5
9.0
8.5
8.0
7.5
7.0
6.5
6.0
5.5
5.0
4.5
4.0
3.5
3.0
2.5
2.0
1.5
1.0
0.5
f2 (ppm)
1
2
3
4
5
6
7
8
9
10
f1 (ppm)

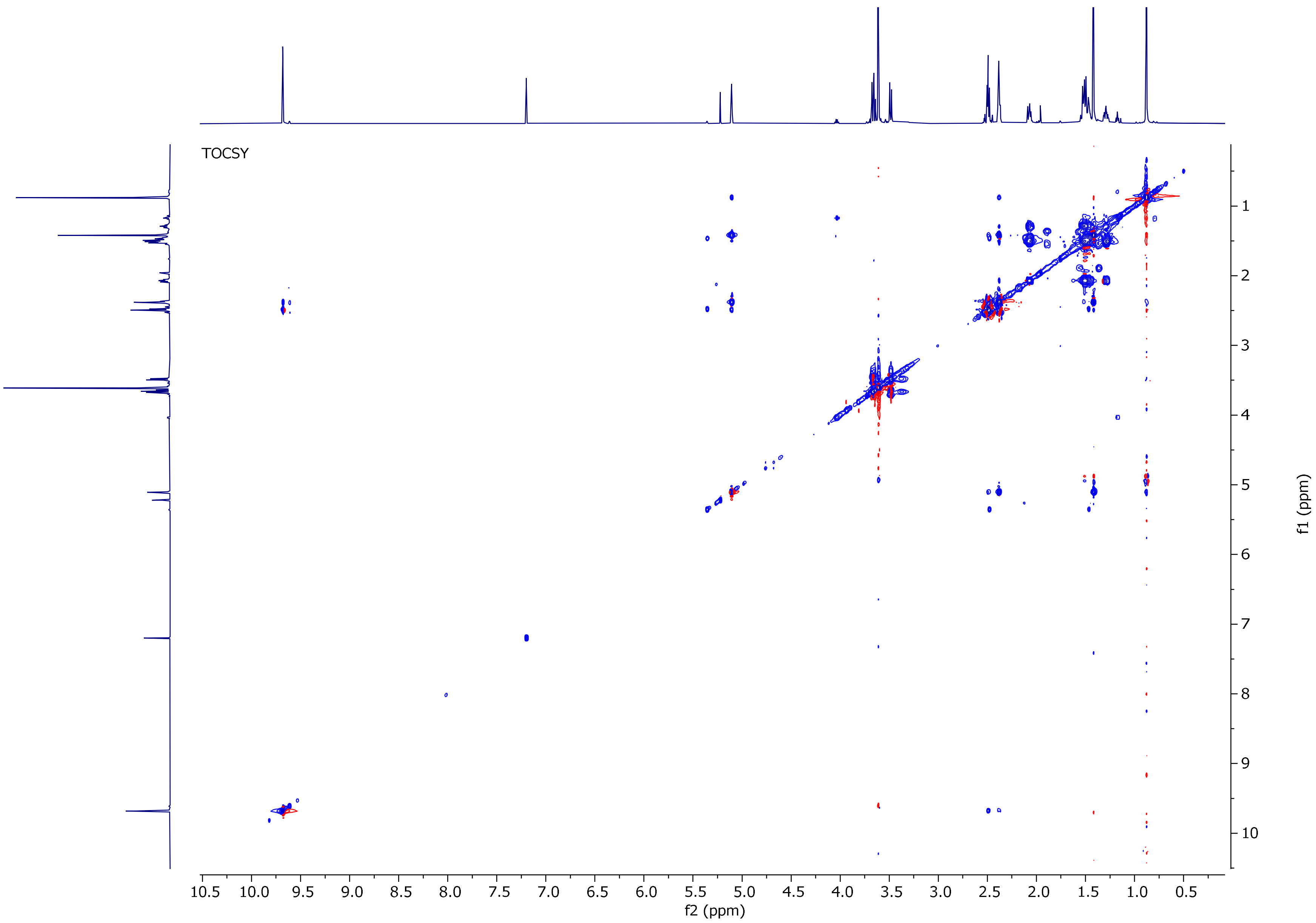

TOCSY
f2 (ppm)
f1 (ppm)
10.5 10.0 9.5 9.0 8.5 8.0 7.5 7.0 6.5 6.0 5.5 5.0 4.5 4.0 3.5 3.0 2.5 2.0 1.5 1.0 0.5
1 2 3 4 5 6 7 8 9 10

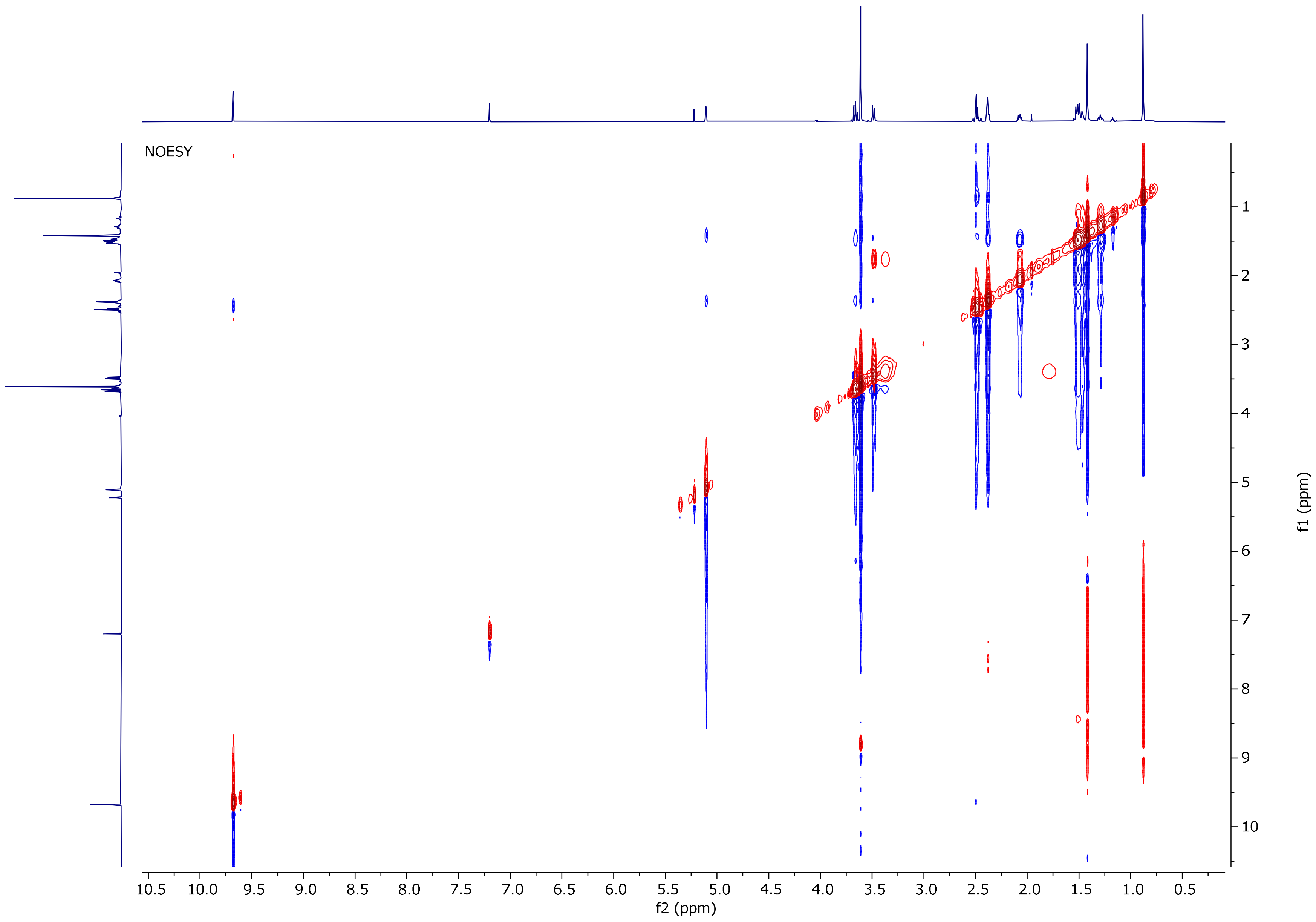
NOESY
10.5
10.0
9.5
9.0
8.5
8.0
7.5
7.0
6.5
6.0
5.5
5.0
4.5
4.0
3.5
3.0
2.5
2.0
1.5
1.0
0.5
f2 (ppm)
1
2
3
4
5
6
7
8
9
10
f1 (ppm)

^{1}H NMR, DEPT 135, ^{13}C NMR, IR, HSQC, HMBC, COSY, TOCSY and NOESY of **24**

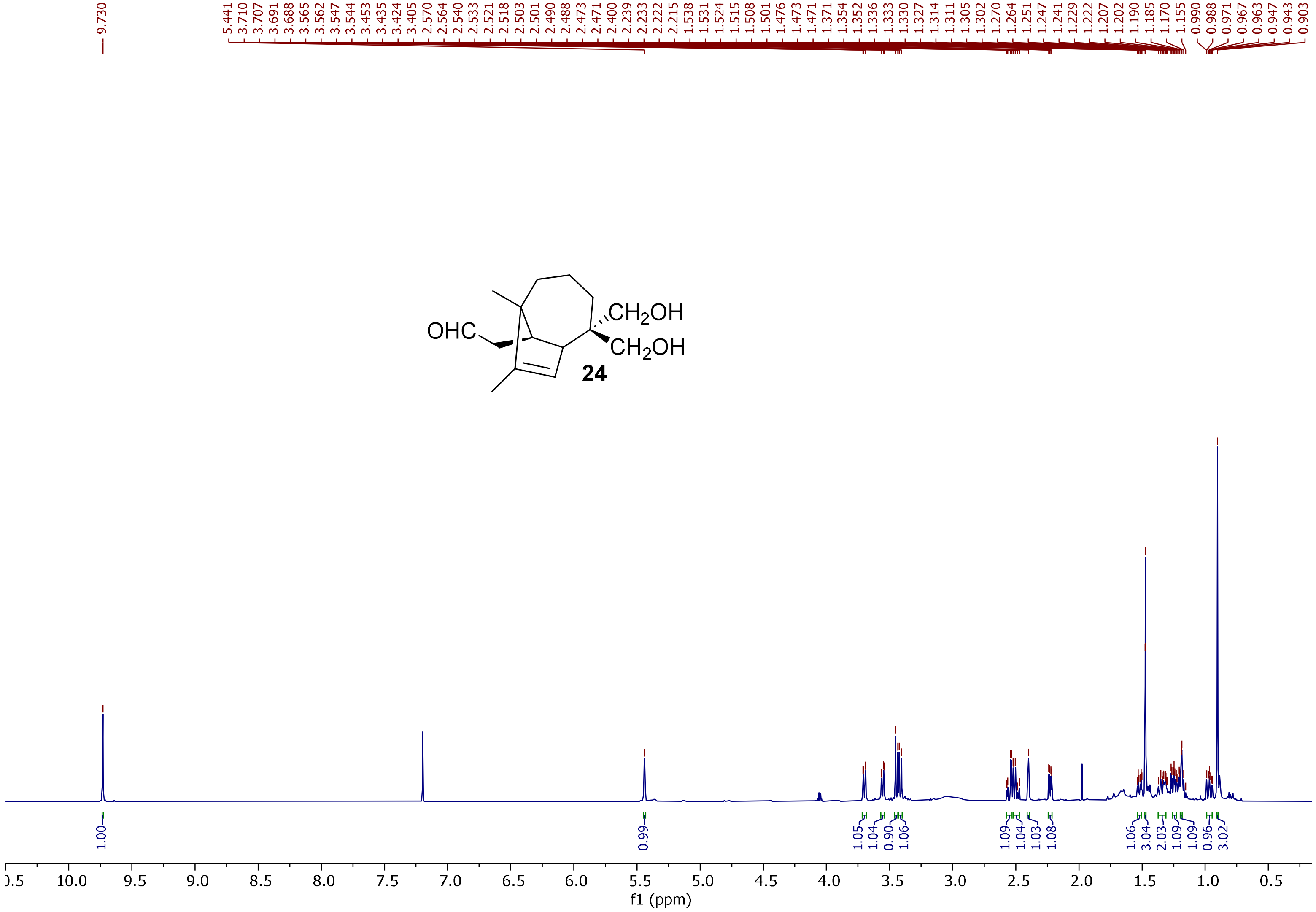

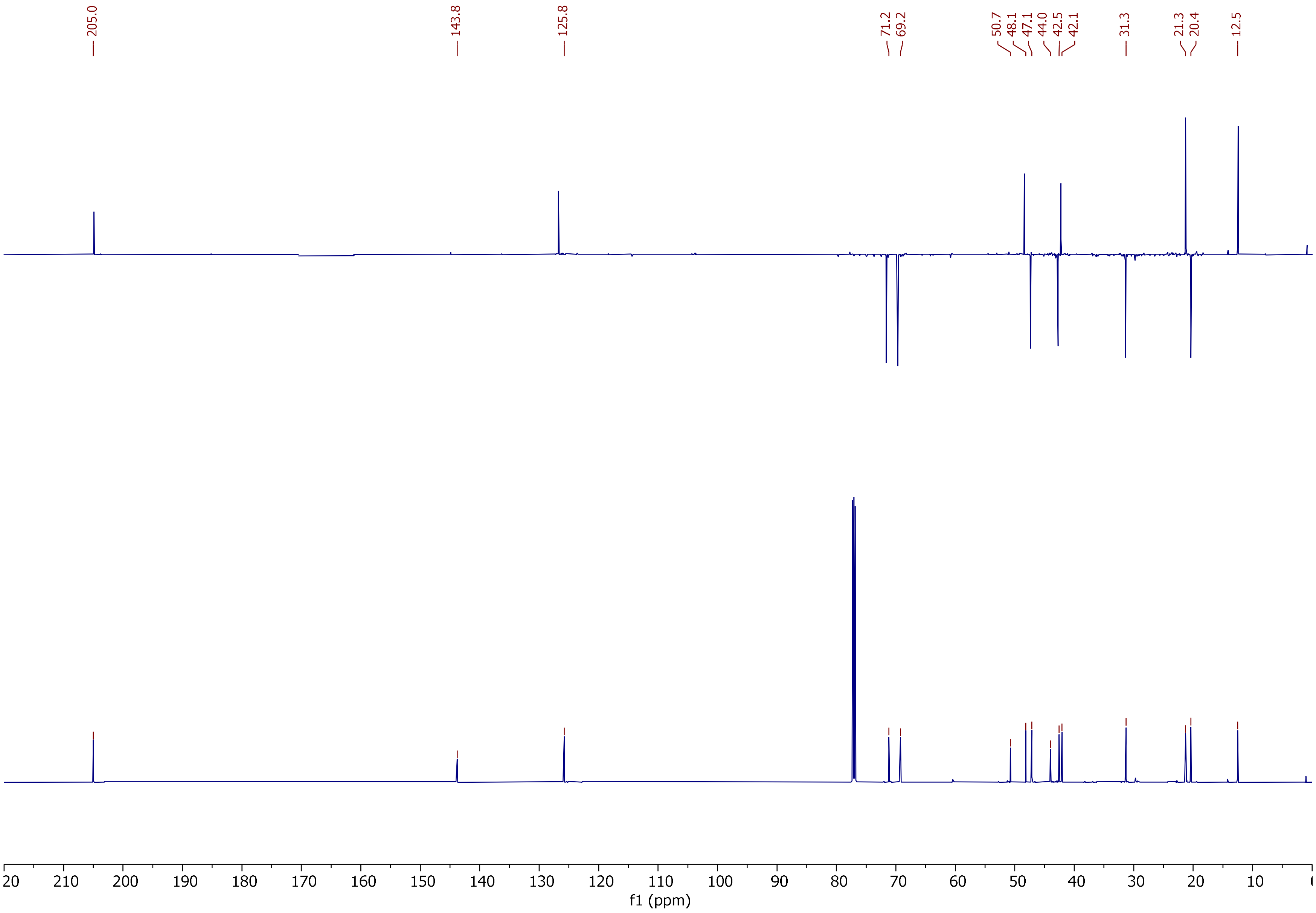
205.0
143.8
125.8
71.2
69.2
50.7
48.1
47.1
44.0
42.5
42.1
31.3
21.3
20.4
12.5
f1 (ppm)

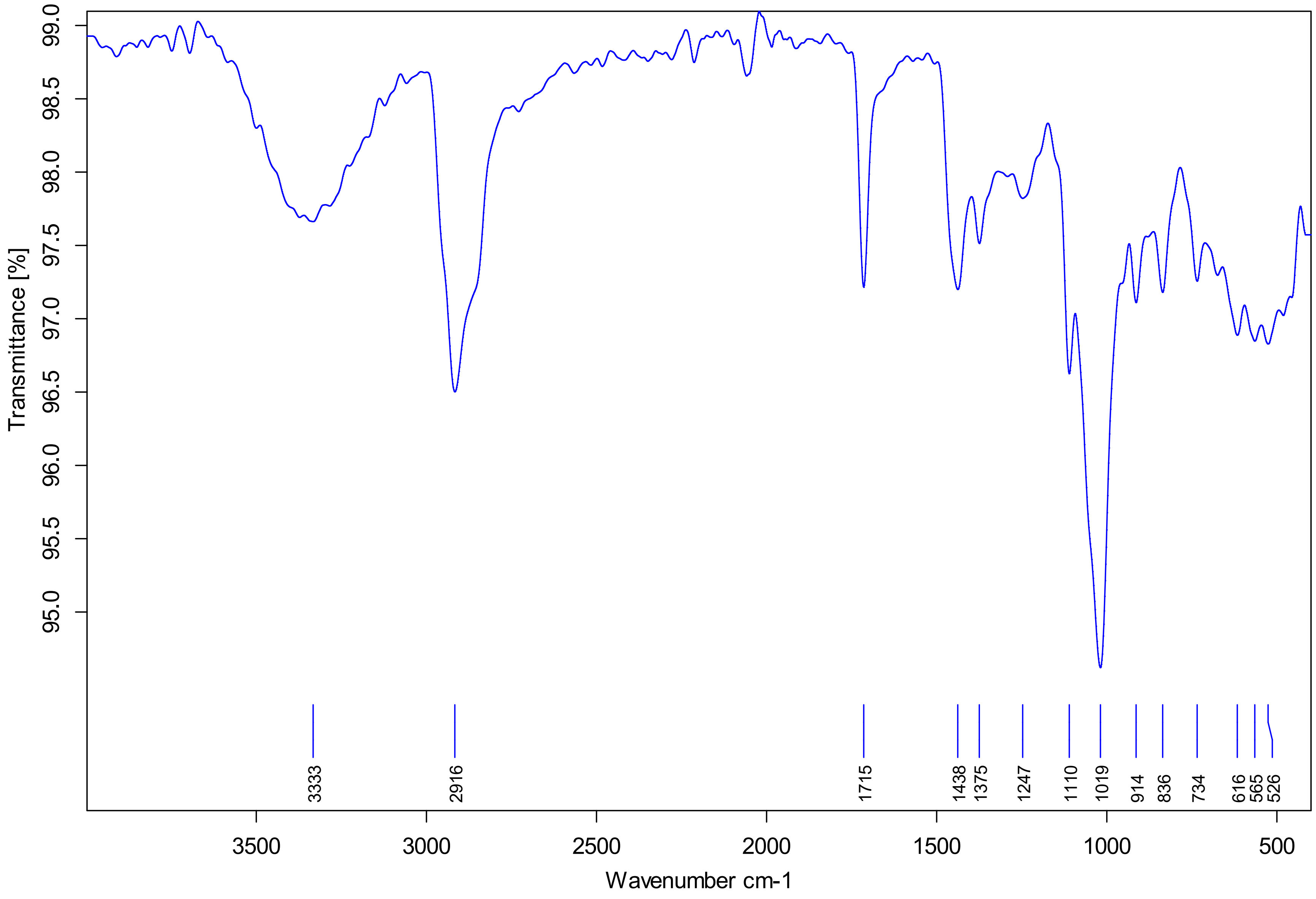
Transmittance [%]
99.0
98.5
98.0
97.5
97.0
96.5
96.0
95.5
95.0
3333
2916
1715
1438
1375
1247
1110
1019
914
836
734
616
565
526
3500
3000
2500
2000
1500
1000
500
Wavenumber cm-1

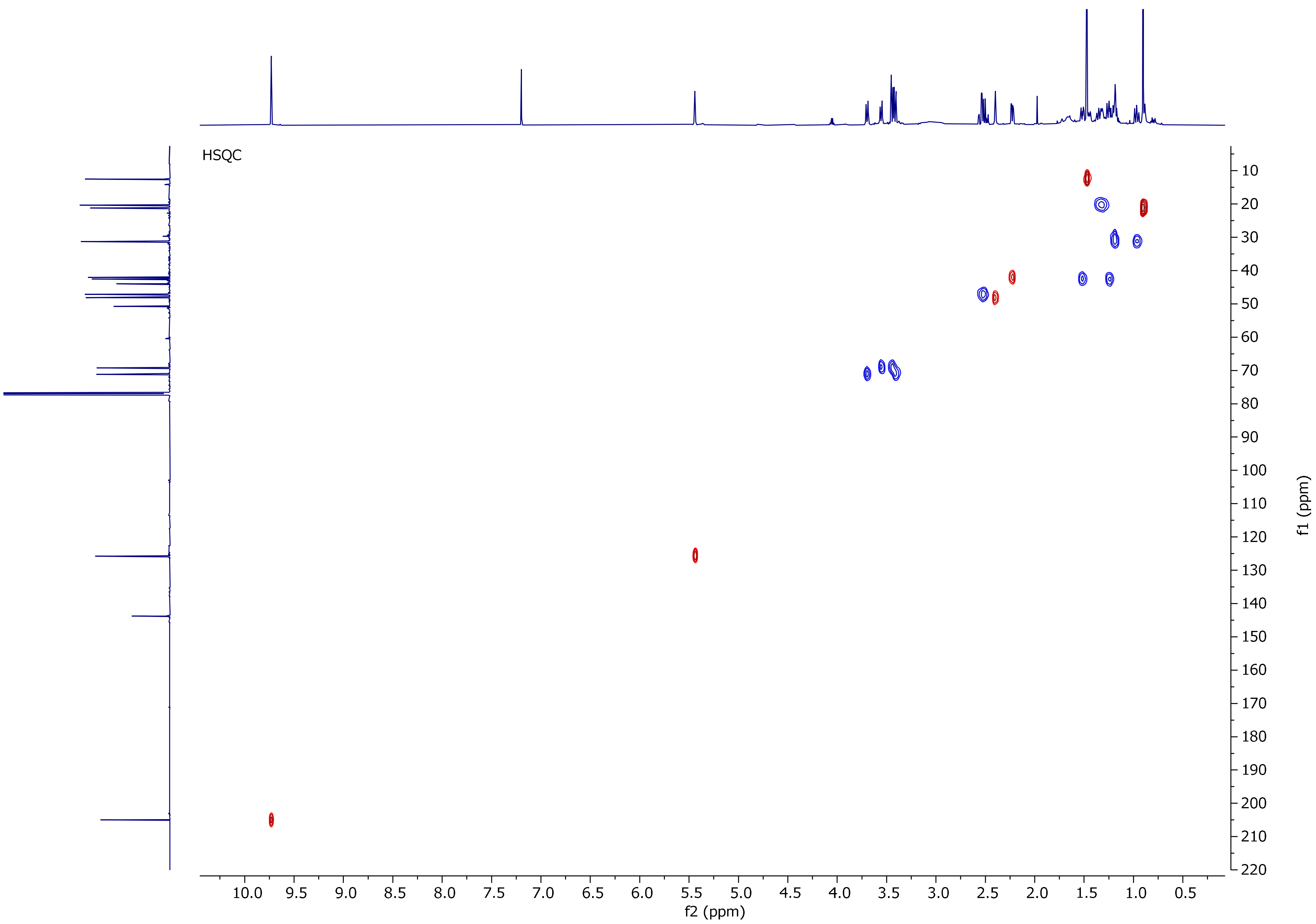
HSQC
10
20
30
40
50
60
70
80
90
100
110
120
130
140
150
160
170
180
190
200
210
220
f1 (ppm)
10.0
9.5
9.0
8.5
8.0
7.5
7.0
6.5
6.0
5.5
5.0
4.5
4.0
3.5
3.0
2.5
2.0
1.5
1.0
0.5
f2 (ppm)

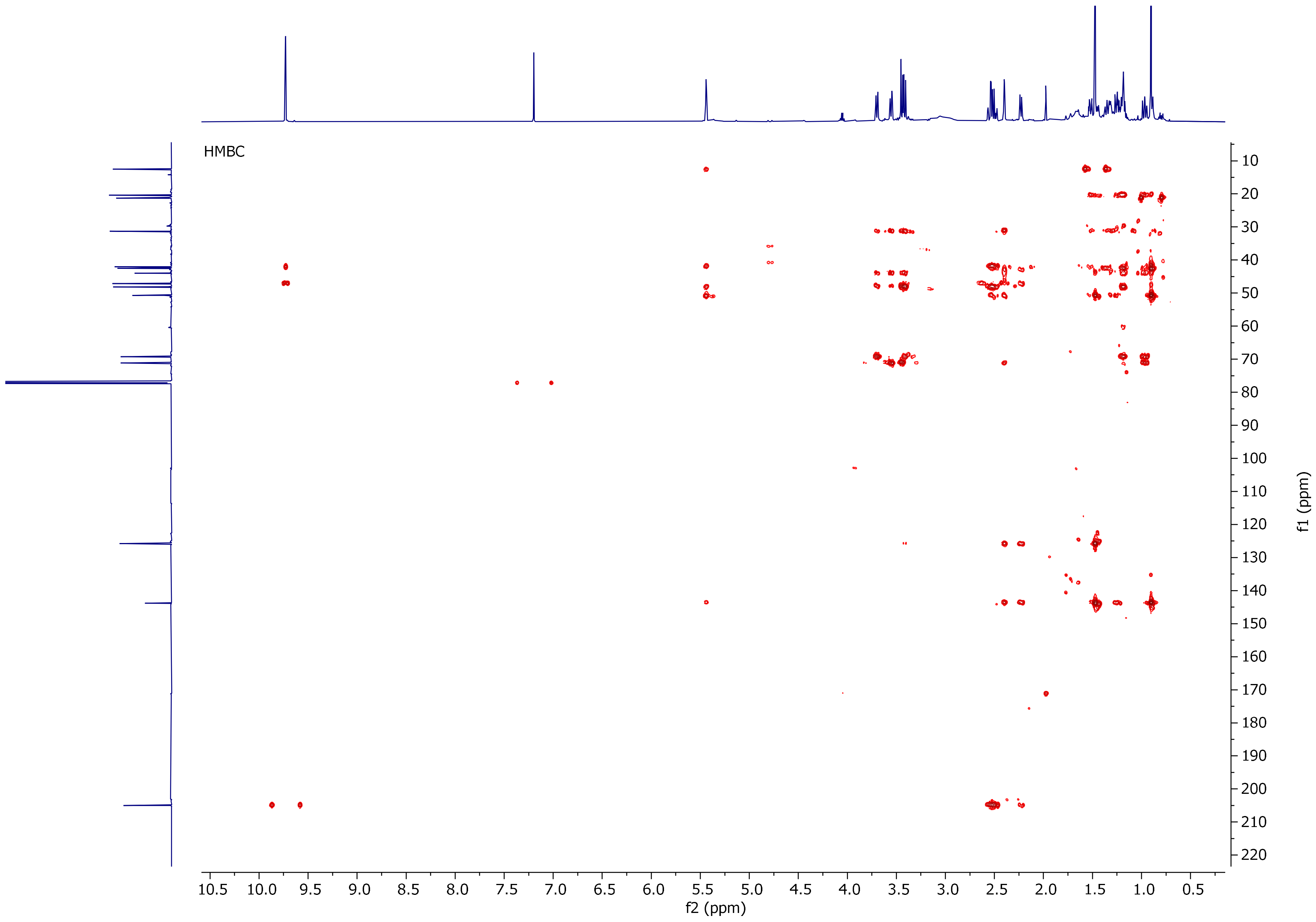
HMBC
f2 (ppm)
f1 (ppm)

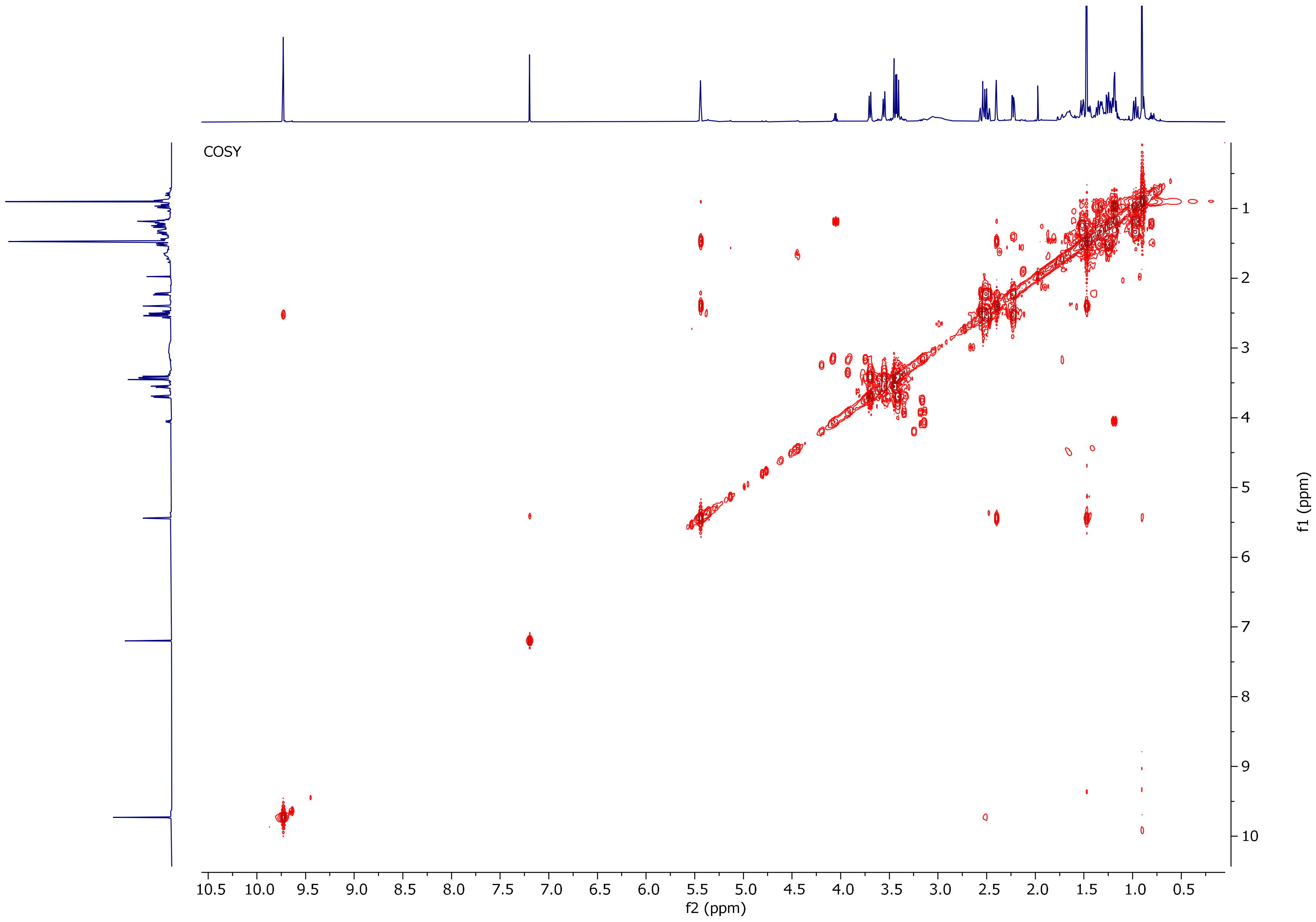
COSY
f2 (ppm)
f1 (ppm)

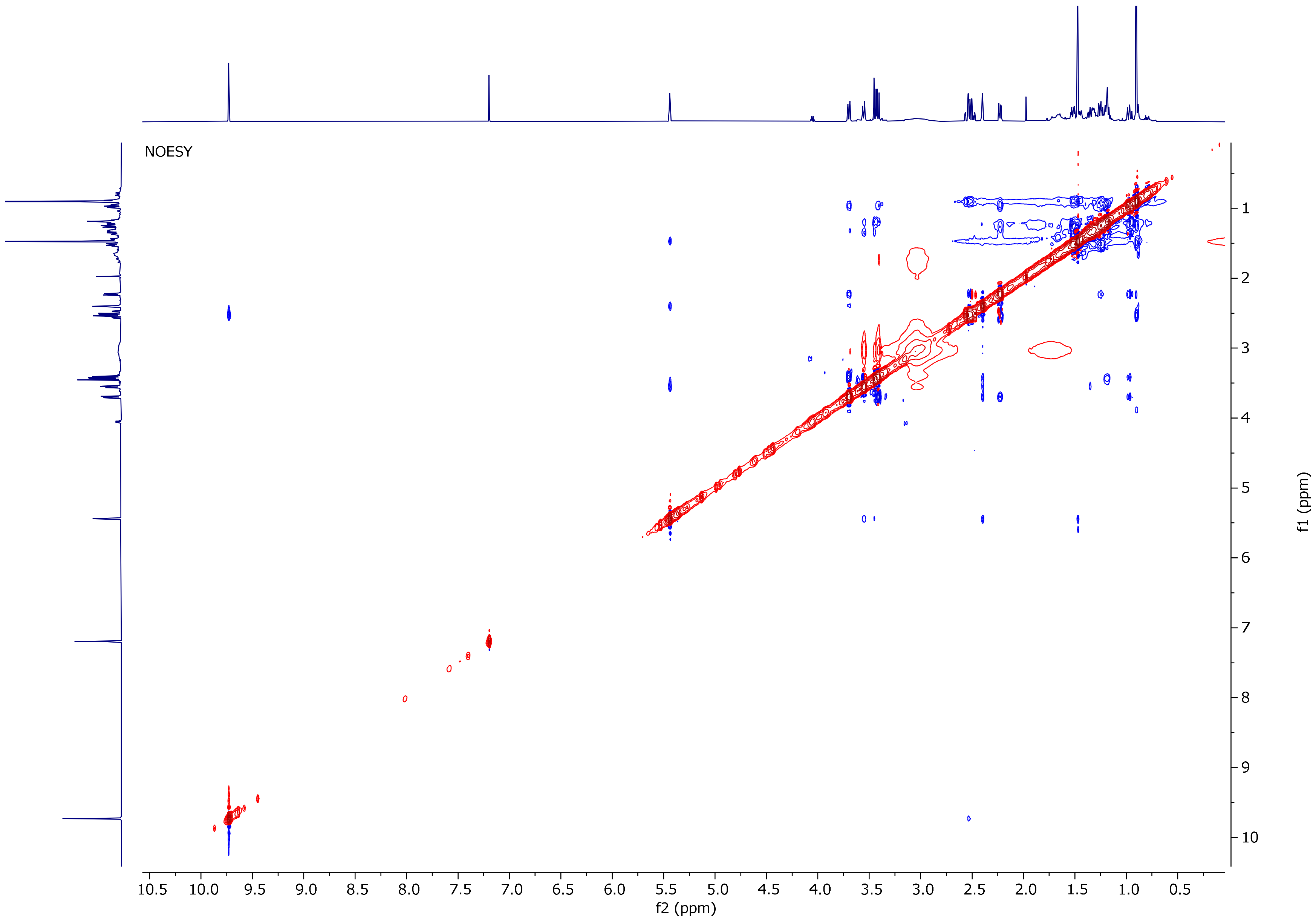
NOESY
f2 (ppm)
f1 (ppm)
10.5
10.0
9.5
9.0
8.5
8.0
7.5
7.0
6.5
6.0
5.5
5.0
4.5
4.0
3.5
3.0
2.5
2.0
1.5
1.0
0.5
1
2
3
4
5
6
7
8
9
10

CRYSTAL DATA AND STRUCTURE REFINEMENT FOR 13

Table 1. Crystal data and structure refinement for **13**.

	**longibornane 13**
empirical formula	$C_{15}H_{22}O_3$
molecular weight	250.32
temperatura (K)	155.70
crystal size (mm^3)	0.372 x 0.231 x 0.07
crystal system	orthorhombic
space group	$P2_12_12_1$
a (Å)	8.1459 (2)
b (Å)	12.0831 (4)
c (Å)	13.4903 (4)
α (deg)	90
β (deg)	90
γ (deg)	90
volume (Å^3)	1327.82 (7)
Z, ρ calculated (g/cm^3)	4, 1.252
μ (mm^{-1})	0.685
F (000)	544.0
radiation	CuKα (λ = 1.54178)
2Θ range for data collection (deg)	12.694 to 143.86
index ranges	$-10 \le h \le 9$, $-14 \le k \le 14$, $-16 \le 1 \le 16$
collected reflections	16026
independent reflections	2585 [R_{int}= 0.0402, R_{sigma}= 0.0282]
data/restraints/parameters	2585/0/168
goodness-of-fit on F^2	1.067
final R indexes [I>=2σ (I)]	R_1= 0.0341, wR_2= 0.0917
final R indexes [all data]	R_1= 0.0349, wR_2= 0.0927
largest diff. peak/hole (e Å^{-3})	0.17/-0.15
Flack parameters	0.09 (5)

Table 2. Fractional atomic coordinates (×10⁴) and equivalent isotropic displacement parameters (Å²×10³) for **13**. U_{eq} is defined as 1/3 of the trace of the orthogonalised U_{IJ} tensor.

Atom	*x*	*y*	*z*	U(eq)
O3	7852(2)	7807.7(13)	4941.0(13)	60.0(4)
O1	7757.0(17)	5096.9(12)	4684.3(9)	42.7(3)
O2	10314.4(18)	5701.4(14)	4760.8(12)	55.9(4)
C13	6077(3)	3130.4(18)	7444.4(19)	59.1(6)
C2	6651(2)	4037.1(14)	6729.9(13)	37.6(4)
C1	6025(2)	5204.0(14)	7016.4(12)	34.3(4)
C7	6546(2)	5926.3(12)	6136.5(12)	31.9(3)
C6	8367(2)	6257.6(13)	6052.1(13)	34.0(4)
C14	8551(3)	7511.8(16)	5859.3(17)	47.6(5)
C8	6236(2)	5150.6(15)	5247.7(12)	35.4(4)
C15	8952(2)	5676.2(15)	5115.6(14)	38.5(4)
C9	5730(2)	4036.6(14)	5715.4(13)	38.7(4)
C12	5940(3)	3045.0(18)	5038(2)	60.9(6)
C3	8549(2)	3930.3(15)	6650.2(15)	42.6(4)
C4	9503(3)	4752.6(19)	7281.6(16)	50.4(5)
C5	9456(2)	5967.4(18)	6944.6(15)	45.8(4)
C11	4145(2)	5078.2(17)	6929.5(16)	45.7(4)
C10	3942(2)	4262.3(17)	6051.9(17)	48.1(5)

Table 3. Anisotropic displacement parameters (Å^2×10^3) for **13**. The anisotropic displacement factor exponent takes the form: $-2\pi^2[h^2a^{*2}U_{11}+2hka^*b^*U_{12}+\ldots]$.

**Atom**	**U11**	**U22**	**U33**	**U23**	**U13**	**U12**
O3	62.9(9)	48.5(9)	68.6(10)	17.9(8)	5.9(8)	7.1(7)
O1	46.5(7)	50.5(7)	31.0(6)	-5.2(6)	4.0(5)	-5.9(6)
O2	42.4(8)	66.6(9)	58.5(9)	-2.0(8)	16.4(6)	-3.2(7)
C13	73.5(15)	45.1(10)	58.7(12)	16.5(9)	10.7(12)	-4.2(11)
C2	44.2(9)	31.6(8)	37.1(9)	2.8(7)	3.7(8)	1.5(7)
C1	35.1(8)	35.9(8)	32.0(7)	-3.4(6)	4.5(6)	-2.0(7)
C7	32.1(8)	28.1(7)	35.4(8)	-1.7(6)	-0.3(7)	1.4(6)
C6	32.3(8)	32.9(8)	36.8(8)	-1.5(6)	-0.5(7)	-1.2(6)
C14	44.8(10)	35.6(9)	62.4(12)	-0.7(8)	3.3(9)	-7.2(8)
C8	34.8(8)	39.1(8)	32.4(8)	-0.7(7)	-3.6(6)	-1.4(7)
C15	37.7(9)	40.1(8)	37.6(8)	4.6(7)	5.2(7)	0.4(7)
C9	43.0(9)	32.5(8)	40.5(9)	-6.9(7)	0.4(8)	-4.2(7)
C12	73.0(14)	45.2(10)	64.5(13)	-23.5(10)	3.3(12)	-9.2(10)
C3	45.9(10)	37.9(8)	44.1(9)	6.9(7)	1.0(9)	12.6(8)
C4	41.4(10)	65.2(13)	44.7(10)	9.7(9)	-10.8(8)	7.1(9)
C5	38.3(9)	55.4(11)	43.8(10)	-3.2(8)	-8.1(8)	-3.9(8)
C11	34.9(9)	44.3(9)	57.8(11)	-7.2(8)	10.4(8)	-4.8(8)
C10	37.0(9)	45.8(10)	61.6(12)	-6.9(9)	0.0(9)	-9.9(8)

Table 4. Bond lengths for **13**.

Atom	Atom	Length/Å	Atom	Atom	Length/Å
O3	C14	1.410(3)	C7	C8	1.543(2)
O1	C8	1.455(2)	C6	C14	1.545(2)
O1	C15	1.332(2)	C6	C15	1.522(2)
O2	C15	1.209(2)	C6	C5	1.536(3)
C13	C2	1.532(3)	C8	C9	1.543(2)
C2	C1	1.548(2)	C9	C12	1.516(3)
C2	C9	1.560(3)	C9	C10	1.550(3)
C2	C3	1.556(3)	C3	C4	1.522(3)
C1	C7	1.533(2)	C4	C5	1.537(3)
C1	C11	1.544(2)	C11	C10	1.550(3)
C7	C6	1.540(2)			

Table 5. Bond angles for **13**.

Atom	Atom	Atom	Angle/°	Atom	Atom	Atom	Angle/°
C15	O1	C8	111.73(13)	O3	C14	C6	110.95(16)
C13	C2	C1	113.19(16)	O1	C8	C7	107.09(13)
C13	C2	C9	113.90(17)	O1	C8	C9	113.72(15)
C13	C2	C3	106.70(16)	C7	C8	C9	104.83(13)
C1	C2	C9	93.50(13)	O1	C15	C6	112.10(14)
C1	C2	C3	114.83(15)	O2	C15	O1	120.72(17)
C3	C2	C9	114.64(15)	O2	C15	C6	127.18(18)
C7	C1	C2	103.53(13)	C8	C9	C2	103.30(13)
C7	C1	C11	105.79(15)	C8	C9	C10	102.56(15)
C11	C1	C2	102.59(14)	C12	C9	C2	118.32(18)
C1	C7	C6	118.16(14)	C12	C9	C8	114.40(17)
C1	C7	C8	102.16(12)	C12	C9	C10	114.90(17)
C6	C7	C8	104.95(13)	C10	C9	C2	101.23(15)
C7	C6	C14	111.16(14)	C4	C3	C2	114.50(16)
C15	C6	C7	104.05(14)	C3	C4	C5	116.44(16)
C15	C6	C14	106.41(15)	C6	C5	C4	117.65(16)
C15	C6	C5	111.36(15)	C1	C11	C10	103.10(15)
C5	C6	C7	116.04(15)	C11	C10	C9	103.60(15)
C5	C6	C14	107.43(15)				

Table 6. Torsion angles for **13**.

A	B	C	D	Angle/°	A	B	C	D	Angle/°
O1	C8	C9	C2	87.22(16)	C7	C8	C9	C10	75.50(16)
O1	C8	C9	C12	-42.8(2)	C6	C7	C8	O1	-2.43(17)
O1	C8	C9	C10	-167.86(14)	C6	C7	C8	C9	118.68(14)
C13	C2	C1	C7	-172.64(17)	C14	C6	C15	O1	-117.21(17)
C13	C2	C1	C11	-62.7(2)	C14	C6	C15	O2	62.6(2)
C13	C2	C9	C8	167.74(16)	C14	C6	C5	C4	-179.36(18)
C13	C2	C9	C12	-64.7(2)	C8	O1	C15	O2	178.19(17)
C13	C2	C9	C10	61.79(19)	C8	O1	C15	C6	-1.9(2)
C13	C2	C3	C4	-99.99(19)	C8	C7	C6	C14	115.50(16)
C2	C1	C7	C6	-75.89(17)	C8	C7	C6	C15	1.34(16)
C2	C1	C7	C8	38.61(16)	C8	C7	C6	C5	-121.36(16)
C2	C1	C11	C10	-34.40(19)	C8	C9	C10	C11	-70.14(18)
C2	C9	C10	C11	36.39(19)	C15	O1	C8	C7	2.78(19)
C2	C3	C4	C5	-70.8(2)	C15	O1	C8	C9	-112.53(16)
C1	C2	C9	C8	50.44(15)	C15	C6	C14	O3	49.1(2)
C1	C2	C9	C12	178.01(18)	C15	C6	C5	C4	-63.2(2)
C1	C2	C9	C10	-55.51(15)	C9	C2	C1	C7	-54.75(15)
C1	C2	C3	C4	26.3(2)	C9	C2	C1	C11	55.16(16)
C1	C7	C6	C14	-131.54(16)	C9	C2	C3	C4	132.93(17)
C1	C7	C6	C15	114.31(16)	C12	C9	C10	C11	165.10(18)
C1	C7	C6	C5	-8.4(2)	C3	C2	C1	C7	64.50(18)
C1	C7	C8	O1	-126.29(14)	C3	C2	C1	C11	174.41(15)
C1	C7	C8	C9	-5.18(17)	C3	C2	C9	C8	-68.97(18)
C1	C11	C10	C9	-1.5(2)	C3	C2	C9	C12	58.6(2)
C7	C1	C11	C10	73.80(18)	C3	C2	C9	C10	-174.91(14)
C7	C6	C14	O3	-63.6(2)	C3	C4	C5	C6	4.1(3)
C7	C6	C15	O1	0.28(19)	C5	C6	C14	O3	168.43(17)
C7	C6	C15	O2	-179.87(19)	C5	C6	C15	O1	126.00(16)
C7	C6	C5	C4	55.6(2)	C5	C6	C15	O2	-54.1(3)
C7	C8	C9	C2	-29.42(17)	C11	C1	C7	C6	176.60(14)
C7	C8	C9	C12	-159.41(18)	C11	C1	C7	C8	-68.91(16)

Table 7. Hydrogen atom coordinates (Å×10^4) and isotropic displacement parameters (Å^2×10^3) for **13**.

Atom	*x*	*y*	*z*	U(eq)
H3	7050(50)	8260(30)	5020(30)	90
H13A	4894.6	3205.67	7559.79	89
H13B	6664.12	3203.2	8075.14	89
H13C	6305.84	2402.55	7155.25	89
H1	6415.2	5474.37	7676.18	41
H7	5829.39	6596.01	6090.08	38
H14A	8003.75	7929.53	6397.19	57
H14B	9729.74	7711.64	5862.02	57
H8	5328.62	5446.3	4824.77	43
H12A	5290.3	3154.09	4434.9	91
H12B	5563.87	2375.82	5380.44	91
H12C	7100.98	2964.05	4862.55	91
H3A	8868.63	3171.31	6848.24	51
H3B	8871.49	4030.96	5948.44	51
H4A	10664.39	4512.29	7302.19	61
H4B	9073.36	4714.55	7967.27	61
H5A	9089.9	6421.5	7513.94	55
H5B	10593.47	6195.88	6785.72	55
H11A	3615.5	5797.59	6784.12	55
H11B	3670.37	4769.69	7546.08	55
H10A	3291.54	4599.6	5510.38	58
H10B	3397.21	3570.86	6268.69	58

Refinement model description

Number of restraints - 0, number of constraints - unknown.

Details:

1. Fixed Uiso
 At 1.2 times of:
  All C(H) groups, All C(H,H) groups
 At 1.5 times of:
  All C(H,H,H) groups, All O(H) groups
2.a Ternary CH refined with riding coordinates:
 C3(H3), C4(H4), C7(H7)
2.b Secondary CH2 refined with riding coordinates:
 C6(H6A,H6B), C11(H11A,H11B), C12(H12A,H12B), C13(H13A,H13B), C14(H14A,H14B), C15(H15A,H15B)
2.c Idealised Me refined as rotating group:
 C1(H1A,H1B,H1C), C10(H10A,H10B,H10C)
